# Solvents’ and Reagents’ Noninnocent Roles in the Groebke–Blackburn–Bienaymé (GBB) Multicomponent Reaction: Experimental and Computational Evidence

**DOI:** 10.1021/acsorginorgau.5c00049

**Published:** 2025-06-12

**Authors:** Marcelo H. R. Carvalho, Pedro P. De Castro, Pedro Beck, Hélio F. Dos Santos, Fabricio Machado, José R. Correa, Brenno A. D. Neto, Giovanni W. Amarante

**Affiliations:** † Chemistry Department, 28113 Federal University of Juiz de Fora, Campus Martelos, Juiz de Fora, Minas Gerais 36036-900, Brazil; ‡ Pharmacy Department, Federal University of Juiz de Fora, Campus Governador Valadares, Governador Valadares, Minas Gerais 35010-180, Brazil; § Laboratory of Medicinal and Technological Chemistry, Chemistry Institute (IQ-UnB), University of Brasília, Campus Universitário Darcy Ribeiro, Brasília, Distrito Federal 70910-900, Brazil

**Keywords:** multicomponent reaction, catalysis, imidazo[1,2-a]heterocycles, solvent effect, mechanism, DFT, bioimaging experiments

## Abstract

Despite the frequent use of alcohols as solvents in GBB (Groebke–Blackburn–Bienaymé) protocols, the mechanistic reasons for their preference remain poorly understood. In this work, we combined experimental and theoretical investigations to elucidate the roles of solvents and reagents in the GBB reaction, revealing their noninnocent behavior. Kinetic experiments, high-resolution ESI­(+)-MS­(/MS), and DFT calculations demonstrated that methanol not only acts as a solvent but also as a cocatalyst, significantly influencing the reaction mechanism and accelerating key steps. We proposed both uncatalyzed and PTSA-catalyzed pathways, including alternative mechanisms involving solvent-participating intermediates. The reaction scope confirmed the method’s robustness, and selected fluorescent products were successfully applied as bioimaging probes in live cells. These findings contribute to a deeper understanding of MCR mechanisms and highlight the critical impact of solvent and reagent effects on their efficiency.

## Graphical abstract

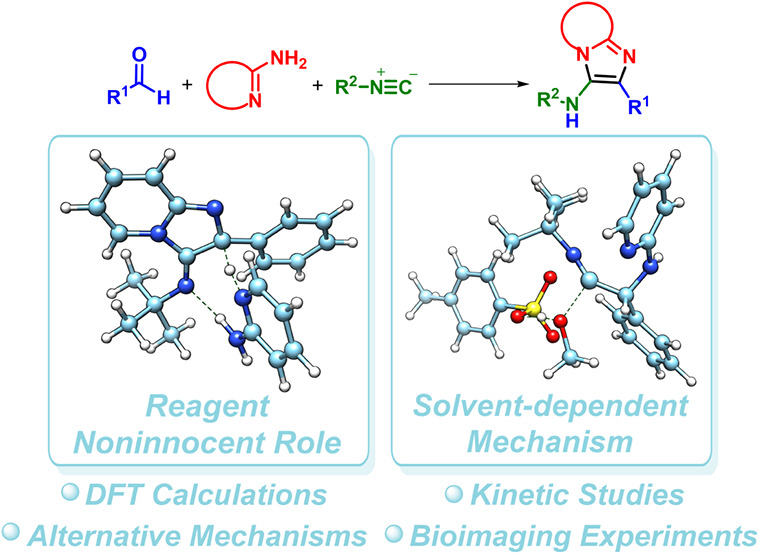

## Introduction

Multicomponent reactions (MCRs) involve transformations in which three or more reagents are added to a single flask for a one-pot reaction, reacting under a wide range of conditions to yield the desired product.^1,2^ Due to advantages such as atom economy, rapid diversification of molecular structures and complexity, waste minimization, and reduced purification requirements, these transformations have attracted growing interest within the chemistry community.^3–5^ These reactions also have significant applications in the preparation of compound libraries for use in high throughput studies in Medicinal Chemistry.^6–9^ Isocyanide-based reactions are among the most important MCRs, encompassing classic transformations such as the Passerini, Ugi, and Groebke–Blackburn–Bienaymé (GBB) reactions.^10,11^ For example, the GBB reaction involves the use of an aldehyde, an isocyanide, and a cyclic amidine (e.g., 2-aminopyridine) to produce imidazo­[1,2-*a*]­heterocycles.^12–16^ These heterocycles exhibit diverse activities, including antileishmanial,^17^ antibacterial,^18^ anticancer,^19^ and have also been noted for their fluorescent properties.^20^ Therefore, the scientific community is demonstrating a growing interest in the study and application of the GBB MCR.

Most protocols for the GBB reaction utilize alcohols as solvents. For instance, Dömling’s group reported a protocol for the synthesis of imidazo-fused heterocycle dimers via consecutive GBB reactions in methanol catalyzed by Scandium­(III) triflate under microwave irradiation (Scheme 1a).^20^ Poole and colleagues described a continuous flow protocol for the classic GBB reaction in ethanol, catalyzed by hydrochloric acid at 130 °C (Scheme 1b).^21^ Although the use of alcohols is widespread for this reaction, an in-depth analysis of the reasons for its use remains a gap in the literature.

**Scheme 1:**
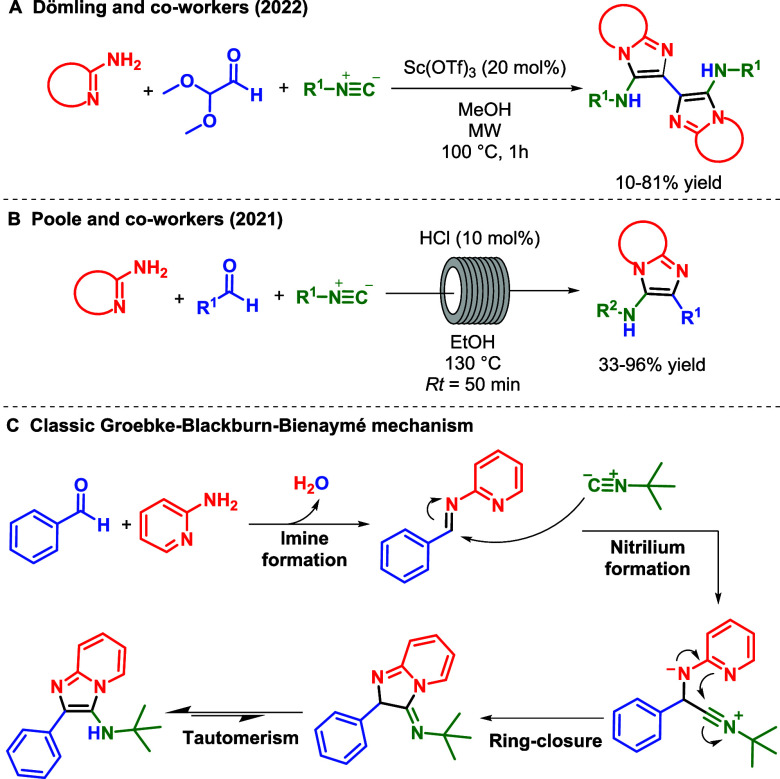
(a, b) Synthetic Approaches to the Groebke–Blackburn–Bienaymé Reaction and (c) the Most Accepted Simplified Mechanism

Reagents are also not always “innocent” in MCRs, may occasionally contribute to the stabilization of intermediates and catalysts, and facilitate product formation. For example, some of us have demonstrated that reagents can lead to the formation of active metal complexes responsible for catalyzing the Biginelli MCR.^22^ Studies of this nature, however, remain scarce in the multicomponent transformation literature.

The currently most accepted mechanism for the GBB reaction is illustrated in Scheme 1c. The reaction begins with imine formation through the condensation between the aldehyde and the cyclic amidine. Subsequently, isocyanide attacks the imine to form a nitrilium intermediate, followed by intramolecular cyclization and a tautomerism/aromatization step. According to this proposal, the solvent effect or the role of any of the reagents is not evident in the mechanism. Our group recently demonstrated that alcohols, especially methanol, can play a crucial role in the Ugi four-component reaction.^23^ In this case, the solvent exhibited an additional, yet unexplored, role in the multicomponent transformation—that of a cocatalyst.

The neglected aspect of solvent effects and their influence on mechanistic pathways in MCRs has been recently highlighted.^24^ While solvent effects have been explored extensively to enhance yields, selectivities, and favor specific mechanisms in various fields,^25^ their significance in MCRs has been relatively overlooked. Despite their paramount importance, only a few studies have systematically accounted for their vital influence in the realm of MCRs,^26–29^ indicating a gap in in-depth studies on this topic.

In this work, we reveal that alcohols may exhibit noninnocent behavior in the GBB reaction, as well as one of the reagents. Our findings contribute to the understanding of solvent effects and the role of reagents in this important MCR by proposing a few alternatives, yet energetically plausible, mechanisms for this multicomponent transformation. By combining theoretical (DFT) calculations and experimental evidence, the results of an in-depth mechanistic investigation are disclosed herein. A series of GBB adducts was efficiently synthesized, and the fluorescent derivatives were applied as fluorescent cell-imaging probes.

## Results and Discussion

We began our investigation by analyzing the GBB reaction in the absence of catalysts. For this purpose, we evaluated various solvents with diverse dielectric constants^30^ in the three-component reaction involving benzaldehyde, 2-amino pyridine, and *tert*-butyl isocyanide (Table 1). The reaction did not occur in toluene (ε = 2.4) and dichloromethane (ε = 9.1) (Table 1, Entries 1 and 2), which is consistent with most synthetic protocols, as they primarily rely on polar and protic solvents. Alcohols bearing sterically hindered groups, such as isopropanol (ε = 18.3) and n-butanol (ε = 17.1) (Table 1, entries 3 and 4), also failed to produce the GBB adduct **1a**. In contrast, ethanol (ε = 24.3) resulted in a small conversion (8% conversion by ^1^H NMR—Table 1, Entry 5), and methanol (ε = 32.6) yielded the best overall result (40% conversion—Table 1, Entry 6). Since the reaction failed in water (ε = 78.5), the dielectric constant of the solvent alone cannot explain the increase in conversions, suggesting that additional effects—and possibly an alternative mechanism—might be involved, as recently described for the Ugi four-component reaction.^23^ Increasing the reaction time in methanol from 6 to 18 h was also effective in increasing the conversion to 67% (Table 1, entry 8).

**Table 1:**
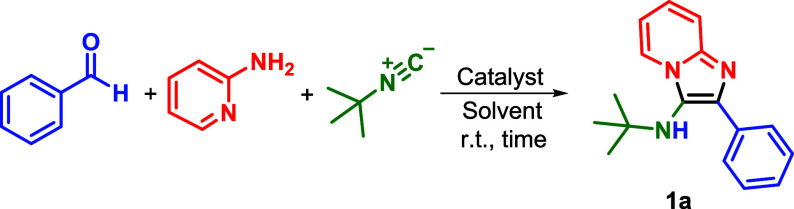
Optimization of the Groebke–Blackburn–Bienaymé Reaction

Entry	Time (h)	Catalyst	Solvent	Conversion^a^ [%]
1	6	-	MePh	-
2	6	-	DCM	-
3	6	-	*i*-PrOH	-
4	6	-	*n*-BuOH	-
5	6	-	EtOH	8
6	6	-	MeOH	40
7	6	-	Water	-
8	18	-	MeOH	67
9	18	AcOH (10 mol %)	MeOH	77
10	18	(±)-CSA (10 mol %)	MeOH	76
11	18	4-Phenolsulfonic acid (10 mol %)	MeOH	77
12	18	PTSA (10 mol %)	MeOH	94 (92)^b^
13	2	PTSA (10 mol %)	MeOH	79
14	4	PTSA (10 mol %)	MeOH	85
15	6	PTSA (10 mol %)	MeOH	94 (90)^b^
16	6	PTSA (5 mol %)	MeOH	61

a Conversion was calculated through the ^1^H NMR analysis of the crude reaction mixture.

b Isolated yield.

Next, we tested the use of a Brønsted acid catalyst aiming at enhancing product formation. Employing 10 mol % of acetic acid and other common sulfonic acids, such as (±)-camphorsulfonic acid and 4-phenolsulfonic acid, resulted in a slight increase in conversions (ranging between 76% and 77%—Table 1, Entries 9–11). As shown in Entry 12 (Table 1), the use of 10 mol % of monohydrated *p*-toluenesulfonic acid (PTSA) efficiently promoted the formation of product **1a**, affording it in excellent conversion (94%) and 92% isolated yield. Further optimization of the reaction time and catalyst loading (Table 1, entries 13–16) revealed that a similar conversion could be achieved using 10 mol % of PTSA in only 6 h. Additionally, other acids with diverse p*K*a values were evaluated for this transformation (e.g., hydrochloric acid, sulfuric acid, phosphotungstic acid), but none yielded better results than PTSA (for full details, see Table S1).

We then focused on preparing a representative reaction scope using the optimized reaction conditions (Scheme 2). Remarkably, the reaction tolerated a variety of substituted benzaldehydes; for instance, chlorine at the para, meta, and even sterically hindered ortho positions yielded products **1b**–**1d** in up to 87% yield. Fluorine and bromine at the para-position were also well tolerated, giving products **1e** and **1f** in 81% and 63% yield, respectively. Both strong electron-donating substituents, such as para-methoxy, and strong electron-withdrawing substituents, such as meta-methoxy and para-trifluoromethyl, provided products **1g**–**1i** in good yields, ranging from 62 to 86%. The use of aldehydes bearing heteroaromatic substituents was also explored, resulting in products **1j**–**1l** in yields of up to 93%. Additionally, using an aliphatic aldehyde, product **1m** was isolated in 87% yield. The formation of other heterocycles was investigated by altering the amine substrate. Pyrimidin-2-amine and benzo­[*d*]­thiazol-2-amine (both less reactive) were employed for this purpose, yielding products **1n**–**1p** in 21–30% yield. Finally, benzyl isocyanide afforded derivative **1q** in 73% yield.

**Scheme 2:**
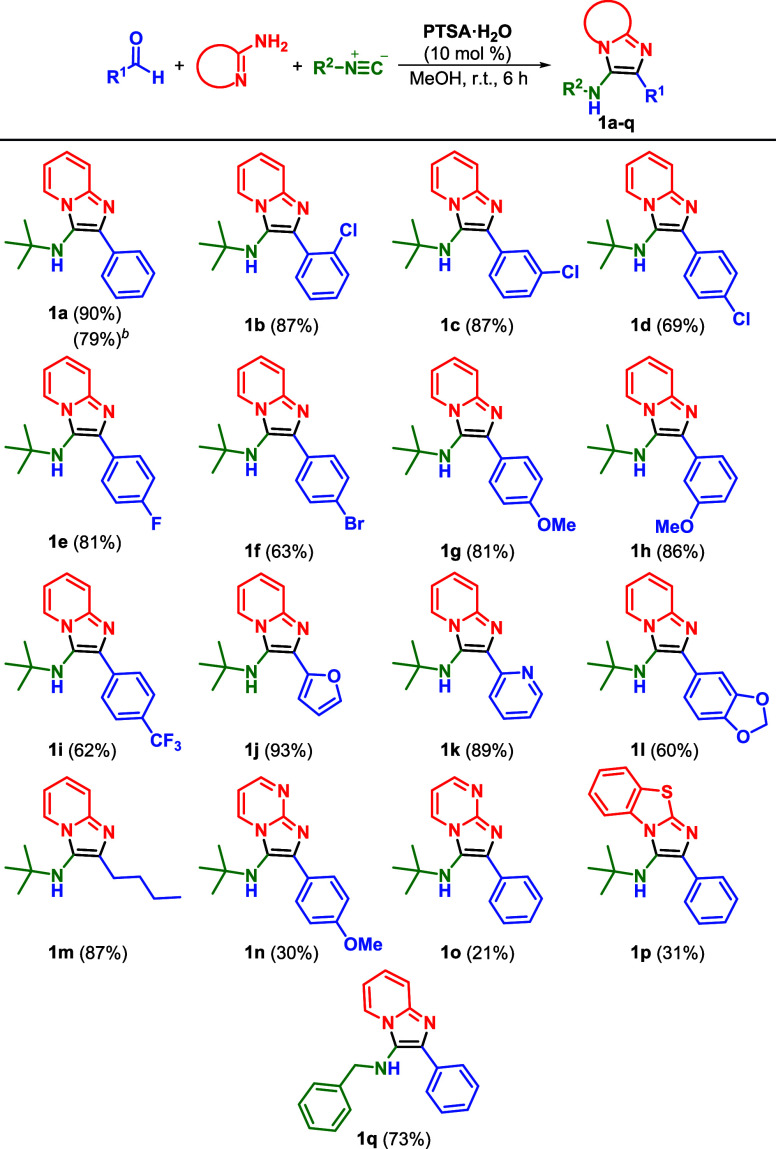
Scope of the Groebke–Blackburn–Bienaymé reaction^a^ ^b^

a Reaction conditions: aldehyde (0.25 mmol, 1 equiv), amidine (1 equiv), isocyanide (1 equiv), PTSA·H_2_O (10 mol %), MeOH (0.5 mL), r.t., and 6 h.
b Reaction carried out in a 2 mmol scale.

As it is evident that the solvent plays a critical role in the reaction, as previously indicated in Table 1, further attempts were made to investigate its impact on the reaction catalyzed by PTSA (Table 2). Although the reaction still failed to yield the product in toluene (Table 2, Entry 1), a small conversion (15%) was observed in dichloromethane (Table 2, Entry 2). Other polar protic and aprotic solvents, such as chloroform, acetonitrile, and water, also failed to increase product formation (Table 2, Entries 3–5). Although protocols employing water as solvent have been reported in the literature,^31–33^ they usually require heating, catalysts, and/or longer reaction times, which can explain the reason that only traces were obtained in our investigation, probably due to imine equilibrium with the starting materials. Once again, alcohols were found to be the best solvents for the GBB reaction. The use of sterically hindered alcohols resulted in lower conversions compared to alcohols bearing small substituents. For example, *tert*-butanol (17% conversion—Table 2, entry 6) and isopropanol (45% conversion—Table 2, entry 7) were considerably less efficient than *n*-butanol (76% conversion—Table 2, entry 8), ethanol (85% conversion—Table 2, entry 9), and trifluoroethanol (88% conversion—Table 2, entry 10). Methanol was the best solvent for this transformation, allowing a conversion of 94% to the desired product.

**Table 2:**
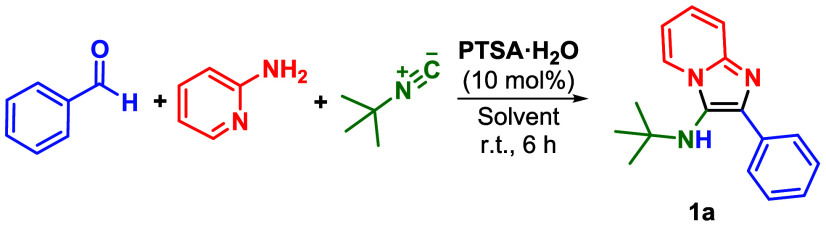
Solvent Influence in the Catalyzed Groebke–Blackburn–Bienaymé Reaction

Entry	Solvent	Conversion^a^ [%]
1	PhMe	Traces
2	DCM	15
3	CHCl3	18
4	MeCN	8
5	Water	Traces
6	*t*-BuOH	17
7	*i*-PrOH	45
8	*n*-BuOH	76
9	EtOH	85
10	TFE	88
11	MeOH	94

a Conversion was calculated through the ^1^H NMR analysis of the crude reaction mixture.

These results suggest that the use of alcohols as solvents has a direct influence on the GBB yields, highlighting the need to investigate the origin of these effects. Initially, to elucidate and quantify the major effect of methanol, the catalyzed reaction of **1a** in deuterated chloroform was monitored by ^1^H NMR for 12 h (Figure 1). The effect of increasing loadings of methanol during the reaction course was investigated. Thus, 1, 5, 15, and 20 equiv of methanol were added at the beginning of the reaction (for full details, see the Experimental Section—Supporting Information).

**Figure 1:**
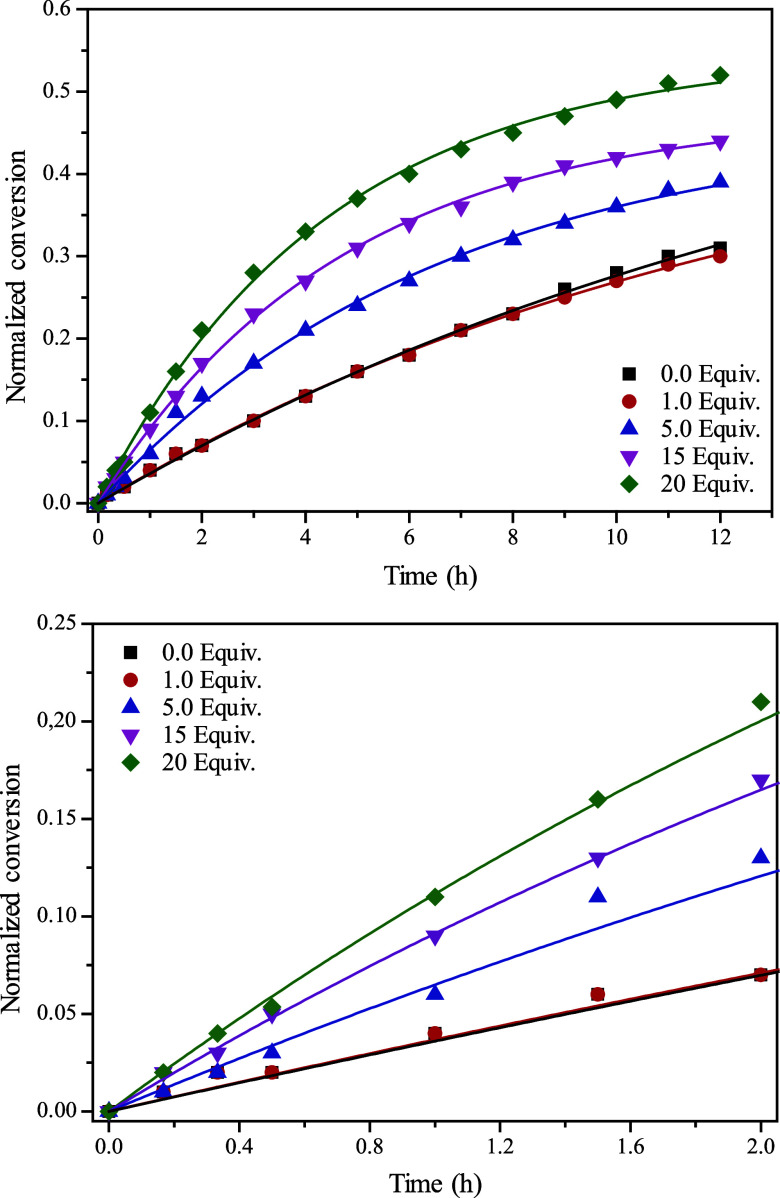
Impact of increasing methanol loadings on the catalyzed GBB reaction as depicted by ^1^H NMR. The lines represent model predictions, while the symbols denote experimentally obtained conversions at specific times. (Top) Full reaction monitoring over 12 h. (Bottom) Initial reaction period monitoring over 2 h.

The addition of 1 equiv of methanol did not increase product formation, in contrast to the effect observed with higher amounts. For example, after 6 h, the 18% conversion in the absence of methanol was lower than the 27%, 34%, and 40% conversion observed with 5, 15, and 20 equiv of the alcohol, respectively. Increasing the methanol loading further enhanced the conversions, indicating that methanol significantly affects the GBB transformation.

To quantitatively access the methanol influence over conversions (and yields), a kinetic approach has been performed based on the data plotted in Figure 1. The kinetic constant *k* and model parameter A (Table 3) were estimated using a nonlinear least-squares approach with the Levenberg–Marquardt (LM) optimization algorithm. A 95% confidence level (significance level, *p* = 0.05) was applied to assess the statistical significance of the estimated parameters for all cases.

**Table 3:** Kinetic Parameters as a Function of Methanol Concentration in the GBB MCR^a^

	Constants	
Equiv. (MeOH)	A	*k* (h^–1^)
0	0.60 ± 0.04	0.062 ± 0.01
5	0.51 ± 0.02	0.075 ± 0.01
10	0.46 ± 0.01	0.152 ± 0.01
15	0.48 ± 0.01	0.213 ± 0.01
20	0.55 ± 0.01	0.228 ± 0.01

a Conversion = *A*(1 – *e*–*^k^* ^*t*^), where *k* is the constant and *t* is the time (h).

The data in Table 3 indicate that the GBB reaction proceeds nearly four times faster in the presence of 20 equiv of methanol than in the absence of this polar and protic solvent, strongly highlighting the positive effect of methanol on the GBB transformation. Given the quantification of this effect (Table 3), it is essential to understand which solvent properties influence the reaction and how these effects combine to favor this MCR transformation.

In this context, the results from Table 2 were therefore plotted using the three different KAT (Kamlet–Abboud–Taft) descriptors: α (hydrogen bond-accepting ability), β (hydrogen bond-donating ability), and π* (dipolarity/polarizability property).^34^ This approach allows for the quantification of key solvent properties and identifies which of them have the most pronounced effect in promoting the reaction. When we considered all solvents without separating them into classes—polar and protic, polar and aprotic, and nonpolar—no significant statistical correlation was observed (data not shown). However, when focusing exclusively on the class of alcohols (polar and protic solvents), interesting correlations emerged (Figure 2). The parameter P (reaction productivity) was expressed in its natural logarithmic form^26^ and is directly related to the yields obtained (from Table 2) of the GBB MCR (for details, see the Experimental Section in Supporting Information).

**Figure 2:**
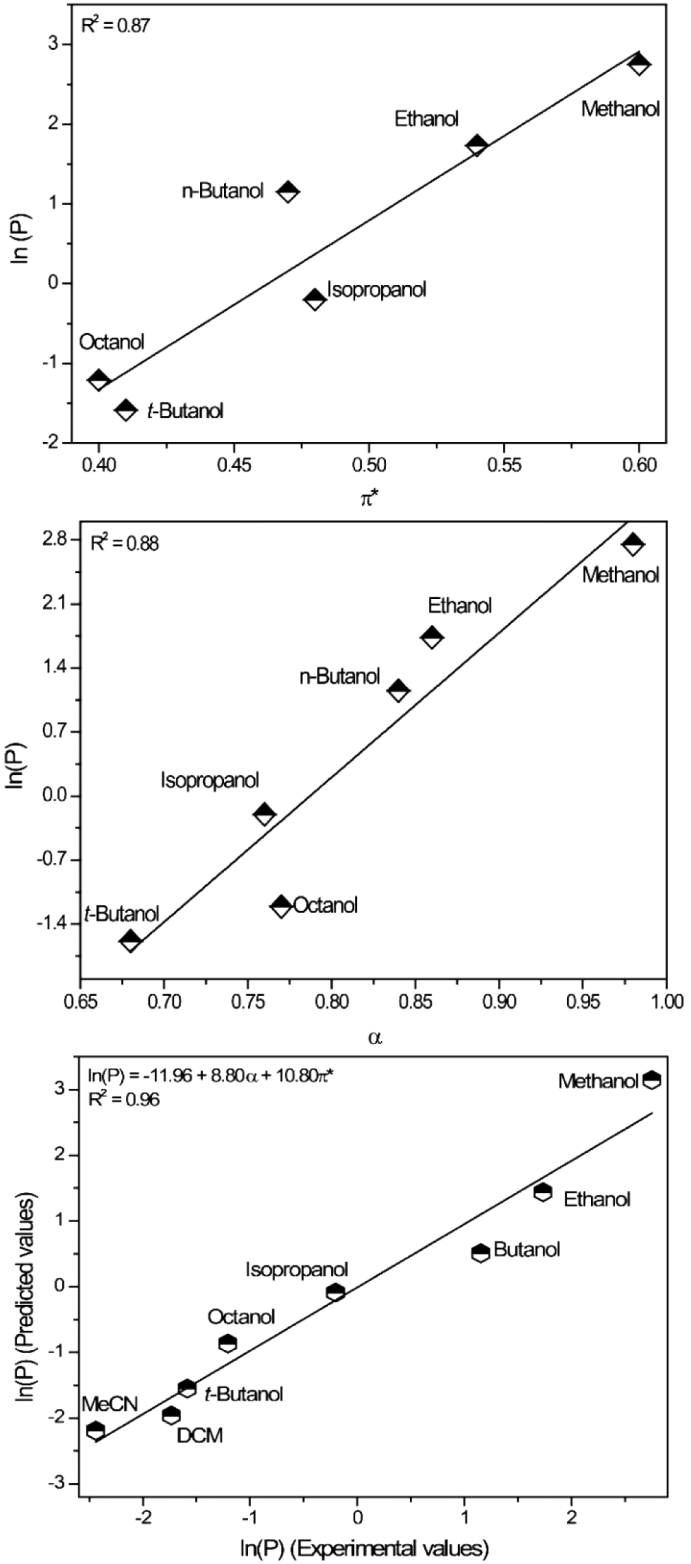
Reaction productivity (direct correlated with yields) as a function of π* (top) and α (center). Multivariate analysis indicates a strong correlation between the α and π* KAT parameters in enhancing reaction productivity (bottom).

In this analysis, trifluoroethanol was excluded due to its known deviation from the KAT model. This deviation, still debated in the literature,^35,36^ is in general attributed to its unique molecular characteristics, particularly a strong hydrogen-bond donating ability due to the electronegative trifluoromethyl group. The result is an unusual polarity profile that diverges from typical alcohols. Additionally, trifluoroethanol exhibits the so-called “hyperpolarity” when mixed with specific components,^35^ such as charged (and polar) species, as proposed in the GBB MCR mechanism (see Scheme 1c), where complex solvent–solvent and solute–solvent interactions further distort expected KAT scales. These interactions lead to enhanced local dipolarity/polarizability values, creating a solvation environment that deviates from ideal interaction predictions.^36^

The analysis of the β descriptor returned no significant results, indicating that this parameter is not essential in governing the transformation. However, considering α and π*, important insights emerge from these plots (Figure 2). The formation of charged and polar intermediates, with multiple heteroatoms and the potential for hydrogen bond formation under acidic conditions, already suggests that these two parameters can influence this MCR. Additionally, the results presented here align well with previous findings indicating the efficiency of PTSA as a Brønsted acid catalyst in high-polarity solvents (e.g., methanol).^37^

The GBB MCR’s nature underscores its pronounced sensitivity to polarity, as potential transition states throughout different reaction stages display significantly higher polarity than the initial reagents. This property makes the reaction kinetics highly responsive to the polarity of the surrounding environment, aligning with the observations and conclusion depicted from Figure 1 (Table 3). In this context, the π* parameter, in a certain way, expresses a transition from “neutral” to “dipolar” states. For instance, studies on Menshutkin (S_N_2-type) reactions across varied substrates in different media—such as organic solvents, ionic liquids, and gases—have shown that π* is a key descriptor for understanding this transformation.^38^ Consequently, the results presented in Figure 2 are well-aligned with previous findings in the literature and with the complex nature of the GBB transformation.

Based on the results presented, it is unsurprising that multivariate analysis identified α and π* as the key KAT descriptors for improving the GBB transformation (Figure 2—bottom), including all types of polar solvents—protic and aprotic. However, additional experimental and theoretical evidence is still needed to clarify the role of the solvent, as well as that of any reagent, throughout the various steps of the GBB MCR and to better understand the nature of the transformation. The KAT descriptors highlight the significance of methanol in advancing the reaction and enhancing its productivity, though the mechanistic steps and intermediates still demand a more detailed elucidation.

In this context, we decided to monitor the reaction in methanol under optimized conditions to gain further insights into the reaction mechanism using electrospray (tandem) mass spectrometry—ESI-MS­(/MS). This technique has been shown to be highly effective for investigating MCRs, as reviewed in prior studies.^39^ We opted for a charge-tagged aldehyde derivative to monitor the reaction (Figures 3 andS60–S66) and prevent the escape of any neutral intermediate, as this strategy has been efficiently applied and recommended in mechanistic evaluations.^40–42^

**Figure 3:**
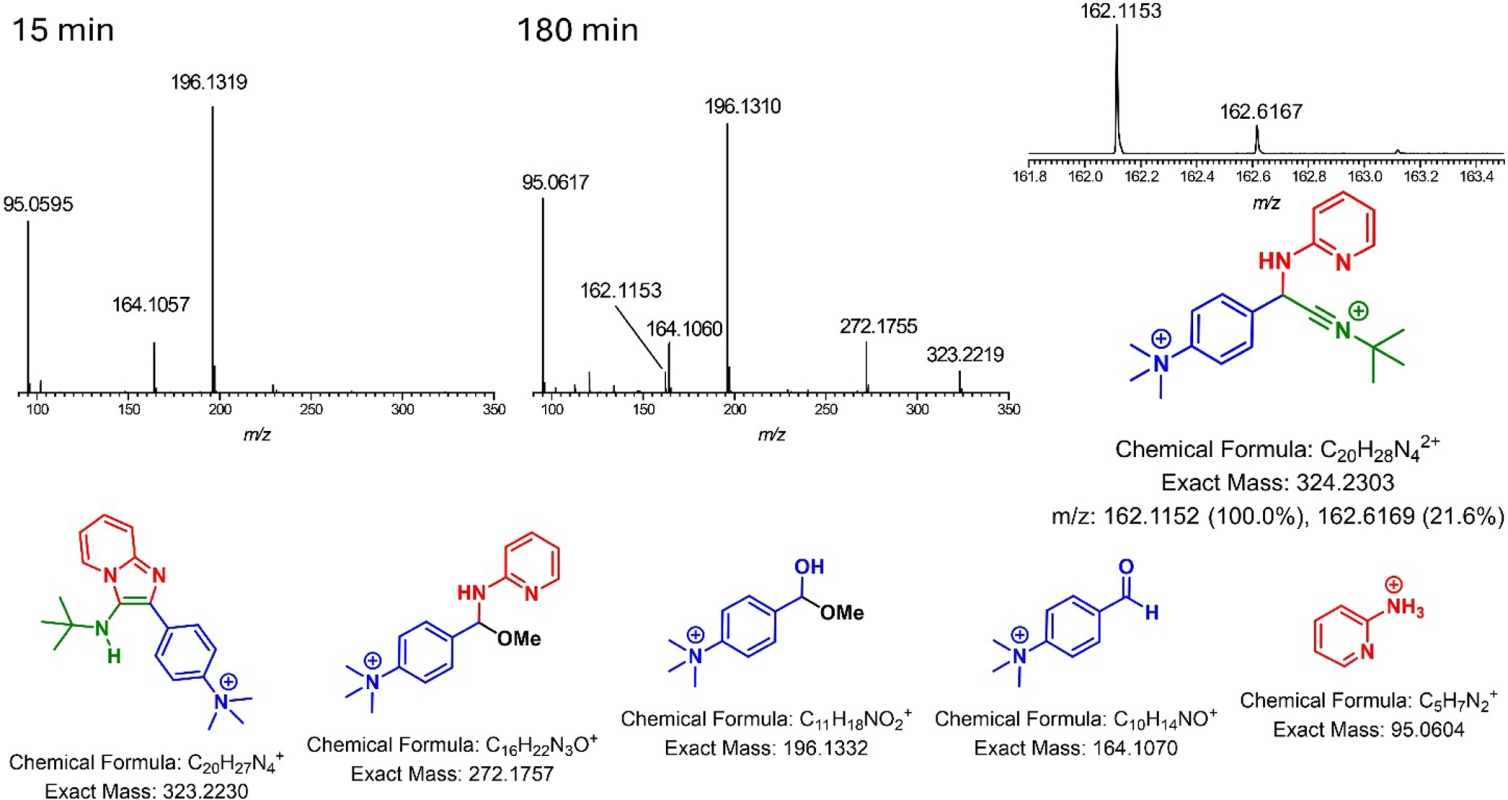
Time ESI­(+)-MS­(/MS) monitoring of the catalyzed (PTSA) GBB reaction using a charge-tagged aldehyde derivative. Drawn structures are shown with their respective theoretical exact masses. All ions were detected with high accuracy and characterized via their tandem MS/MS experiments.

In the initial stage of the reaction monitoring (after 15 min), a rapid addition of the solvent to the charge-tagged aldehyde (*m*/*z* 164) is observed, affording the ion of *m*/*z* 196. This ion is likely a dead end in the reaction and reversibly dissociates back into the reagent (aldehyde) and solvent molecules, representing a side equilibrium with the main reaction pathway. A similar side reaction with methanol is noted after the addition of 2-aminopyridine and the subsequent water elimination, affording the charge-tagged imine intermediate (*m*/*z* 240), which is considered the first step of the reaction mechanism. This ion appears at very low intensities and also undergoes a fast and reversible methanol addition, forming the ion of *m*/*z* 272 with considerable intensity—another dead end—which could be isolated and characterized by MS/MS.

The doubly charged advanced intermediate (*m*/*z* 162), a key species in the reaction, was detected and characterized, especially after 30 min of reaction, and persisted nearly until the end of the monitoring. The final adduct (*m*/*z* 323), formed from the cyclization of this advanced intermediate, was also detected and efficiently dissociated via MS/MS experiments. The use of the charge-tagged reagent proved to be a crucial strategy, especially considering that, without the tag, the advanced intermediate (prior to the intramolecular cyclization step) and the final GBB adduct would display the same nominal *m*/*z* ratio (both singly charged by protonation), preventing unequivocal structure determination via MS/MS and discrimination between these two key species. The presence of the ions of *m*/*z* 196 and 272 should also be noted, as they could potentially lead to the formation of the advanced doubly charged intermediate (*m*/*z* 162) through a direct S_N_2 reaction with the isocyanide reagent, although this hypothesis still requires further investigation and additional experimental evidence. For instance, octanol could undergo this type of reaction, especially considering it is less nucleophilic than methanol; however, as stated, this possibility requires further investigation. All the experimental findings, therefore, call for an in-depth theoretical investigation to verify the energetics and plausibility of the operating GBB mechanism.

To comprehend the role of methanol in the GBB reaction, theoretical calculations using the Density Functional Theory (DFT) were carried out (for full details, see the Experimental Section). Initially, we turned our attention to the reaction in the absence of Brønsted acids, since, as shown in Table 1 (entries 1–8), this reaction occurred only in methanol and ethanol. The classical reaction mechanism (Scheme 1c) was first investigated in methanol, dichloromethane, and toluene (Figure S57). In this traditional proposal, the energy associated with the formation of TS3 (transition state 3 in Figure S57) already indicates the implausibility of this classical mechanistic view.

To identify a pathway in which the barrier of the final step was plausible, we evaluated whether the reagent 2-aminopyridine could act as a proton shuttle in this step. This approach led to a significant decrease in the energy barrier, shifting the rate-limiting step to the nitrilium formation. However, the energy barriers for this initial step are 24.14 kcal·mol^–1^ in methanol, 25.91 kcal·mol^–1^ in dichloromethane, and 26.71 kcal·mol^–1^ in toluene, which—although indicating methanol as the most favorable solvent—till represent relatively high barriers.

To investigate the effect of methanol, its potential role as both solvent and reagent/cocatalyst was evaluated, as already indicated and quantified by the kinetic experiment (see Figure 1). Following proposals previously made for the Ugi and Ugi–Smiles reactions,^23,43^ it was assumed that the reaction could proceed through an imidate intermediate via direct addition of methanol to the nitrilium (Figures S59 and S57). However, this led to a significant increase in the relative barrier of the second step, which is inconsistent and therefore not a suitable pathway. In light of this, we explored the possibility of methanol acting as a proton shuttle (Figures S61 and S62) instead of 2-aminopyridine, resulting in a relative barrier of 28.9 kcal·mol^–1^. We also evaluated the participation of methanol as a hydrogen bond donor, facilitating imine activation for isocyanide addition (Figure S61). This approach led to a decrease of 3.4 kcal·mol^–1^ in the nitrilium formation step, although the final step remained energetically unfavorable.

Bringing together the most relevant insights, we first propose and analyze the uncatalyzed mechanism for the GBB reaction (Figure 4). Initially, methanol acts as a hydrogen bond donor to the preformed imine (**I**), facilitating isocyanide addition and leading to the formation of the nitrilium species (**II**), with methanol stabilizing the negative charge on the nitrogen atom. This is followed by an intramolecular ring closure, forming intermediate (**III**). Finally, a second 2-aminopyridine molecule mediates the proton transfer, yielding the desired product (**IV**).

**Figure 4:**
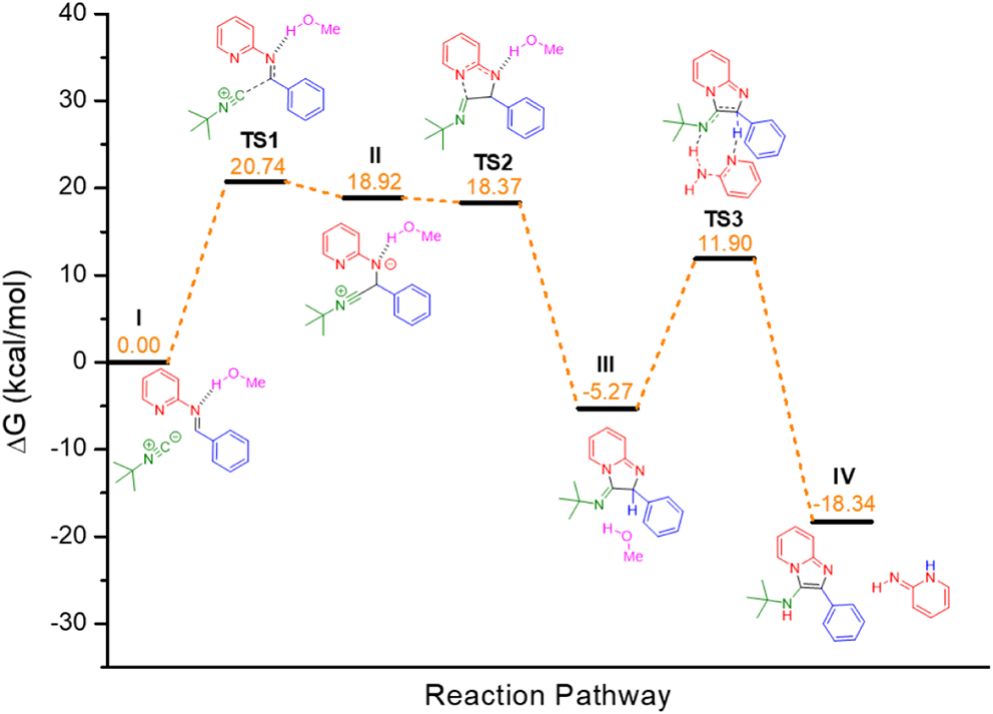
Energetic profile calculated for the noncatalyzed GBB reaction in methanol.

Since there are no reports in the literature on the participation of 2-aminopyridine in the proposed tautomerism step, we investigated the influence of its concentration on the reaction to support this proposition. For this purpose, based on the standard conditions of the noncatalyzed reaction (Table 1, entry 6), an excess of amine was employed (Scheme 3). As predicted, there was a relative and significant increase in product conversion (from 40% to 62%), consistent with the computational results.

**Scheme 3:**
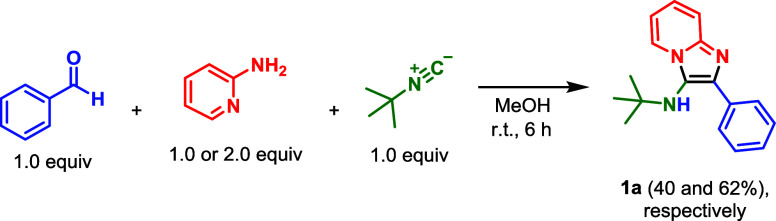
Evaluation of the Influence of 2-Aminopyridine Loading: Amine Excess Led to a Significant Improvement in Yield

Next, we turned our attention to the possible reaction mechanism in the presence of PTSA (Figure 5). As shown in Table 2, although various solvents promoted product formation, alcohols—particularly those with less steric hindrance, such as methanol, trifluoroethanol, and ethanol—led to better conversions. Based on all experimental and theoretically predicted results, a new mechanism can be proposed (Scheme 4) and evaluated. First, the imine (or iminium, in the presence of a Brønsted acid) intermediate (**I**) is generated by the reaction between the amine and the aldehyde. As shown by MS experiments, this intermediate can undergo reversible methanol addition, affording the intermediate (**VII**). This intermediate can undergo an S_N_2 pathway by isocyanide attack, furnishing the intermediate (**II**). However, this mechanistic proposal needs further computational and experimental investigations.

**Figure 5:**
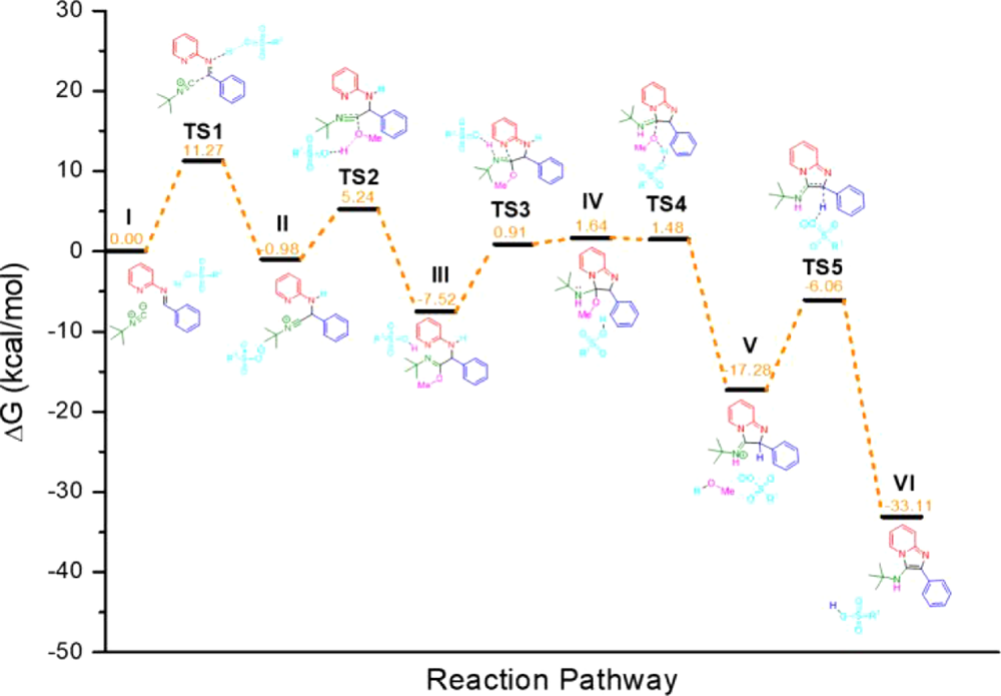
Energetic profile computed for the solvent-dependent alternative reaction pathway for GBB MCR in methanol.

**Scheme 4:**
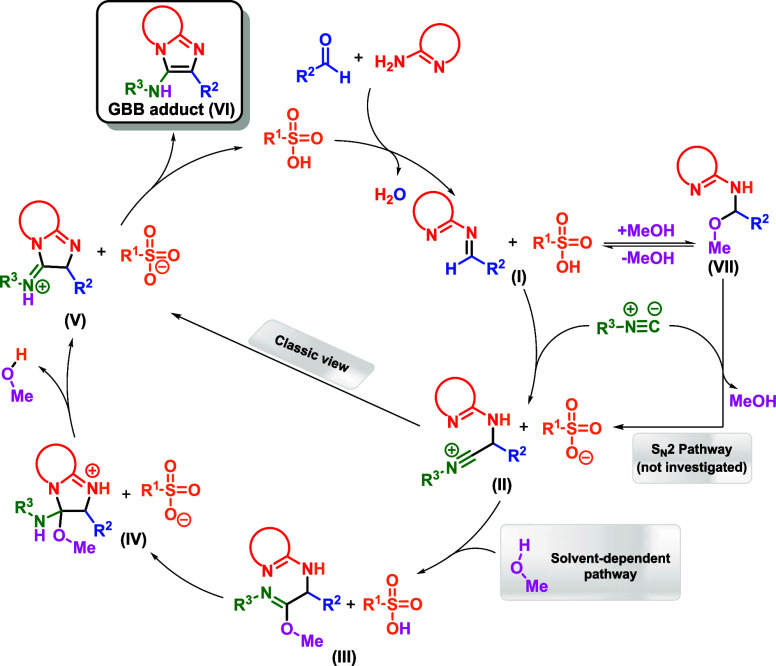
Investigated Mechanisms for the Catalyzed GBB Reaction: Classic and Solvent-Dependent (Alternative) Pathways

Since the iminium is more electrophilic than the imine, the addition of isocyanide to form the nitrilium intermediate (**II**) is likely to proceed with a lower barrier than in the absence of a catalyst. A 5-*exo*-*dig* cyclization then occurs affording intermediate (**V**), mediated by the tosylate, followed by a proton transfer that yields the final GBB adduct (**VI**). Although the energetic profile of the initially investigated mechanism is plausible (Figure S64), the small energy differences observed for methanol, dichloromethane, and toluene do not fully explain the experimental observation that alcohols outperform other solvents when applied to carry out the MCR.

The reaction monitoring by high-resolution ESI (+)-MS­(/MS) allowed the detection of an imidate intermediate, resulting from methanol addition to the imine (see Figure 3—ion of *m*/*z* of 272 related to Scheme 4—intermediate **VII**). DFT calculations indicate that the barrier for this addition is only 5.33 kcal·mol^–1^ (Figure S67), justifying its detection during the experiments. The reverse reaction, releasing methanol and the GBB intermediate, was also favored, in accordance with the hypothesis of a dead-end intermediate.

Two of the GBB adducts (**1n** and **1o**) exhibited visible fluorescence under daylight and were detectable by the naked eye. Therefore, we performed a photophysical characterization of these compounds with the aim of exploring their potential as cell markers, given that functional chromophores synthesized via MCRs represent a prominent new class of heterocycles used as bioimaging probes. Table 4 summarizes the obtained results, and Figures S54 and S55 illustrate the corresponding data.

**Table 4:**
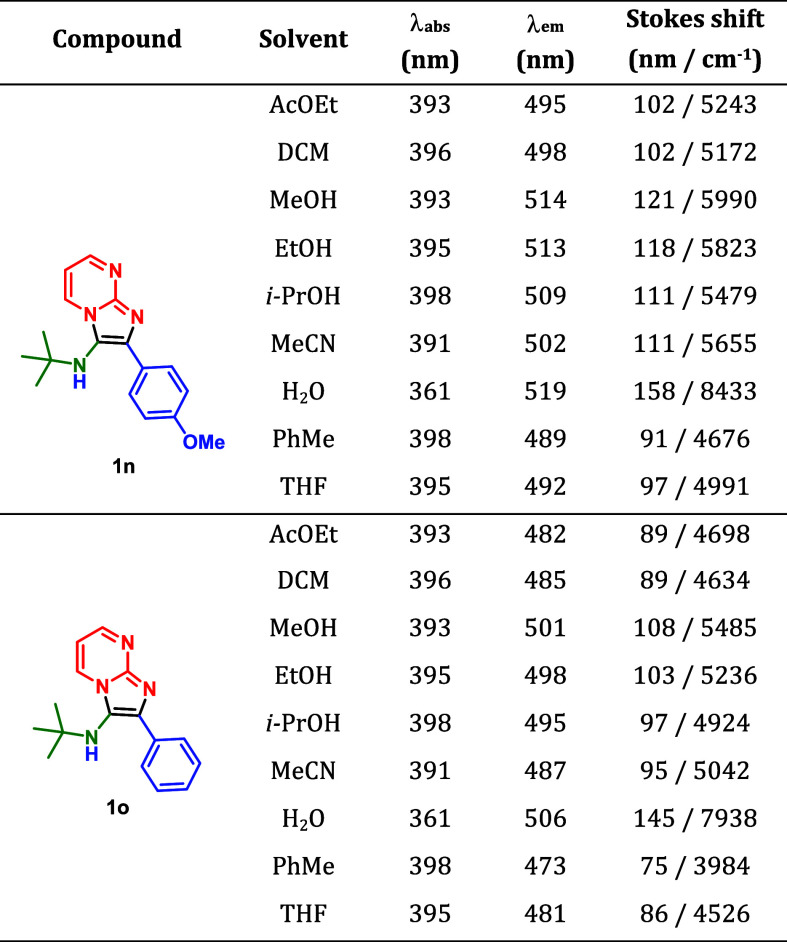
Photophysical Data for Compounds **1n** (70 μM) and **1o** (70 μM)

In general, a large Stokes shift was observed for both synthesized compounds, especially for **1n**, indicating a more efficient intramolecular charge transfer (ICT) process from the first excited state. This effect is likely due to the presence of the methoxy donating group, which enhances the electron-donating ability of the substituent in the D–A architecture observed in both derivatives. The largest Stokes shift for both derivatives was observed in water, indicating their potential for efficient bioimaging applications as bioprobes. Both probes were evaluated for photostability under constant light irradiation (256 nm), and the results indicated good stability (Figure S56), further supporting their suitability for bioimaging applications.

Both compounds, **1n** and **1o**, were then subjected to bioimaging experiments and could be excited with a visible light wavelength of 488 nm. Their fluorescence emissions were recorded in the green range (520–560 nm) and red range (600–680 nm), as shown in Figures 6 and 7. The fluorescence signal was distributed throughout the cytoplasm of all cells, excluding the nuclei, which appeared as black voids in all images, identified by the letter “N.”

**Figure 6:**
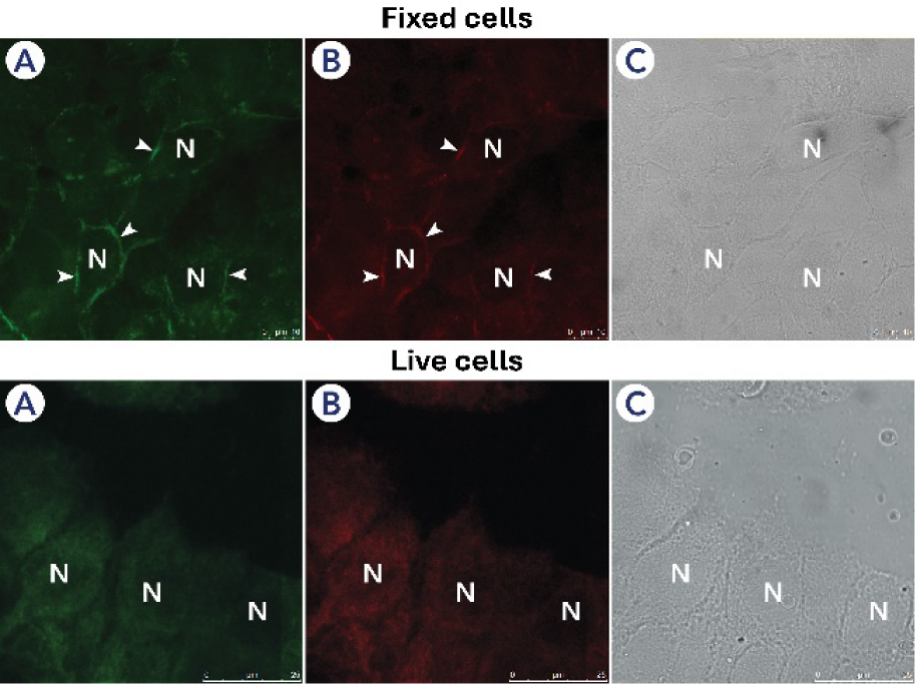
MCF-7 cells, fixed and live samples, incubated with compound **1n** (50 μM). Images A and B show the green and red fluorescent signals dispersed throughout the cytoplasm. The nuclei are indicated by the letter ″N.″ Images C show the normal cell morphology by phase-contrast microscopy. Reference scale bar = 25 μm.

**Figure 7:**
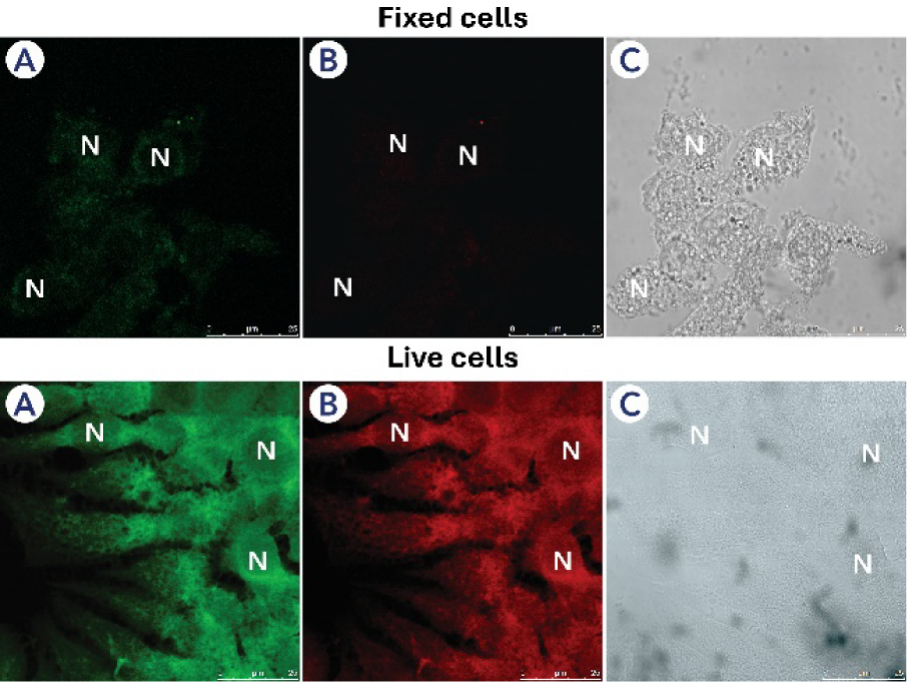
MCF-7 cells, fixed and live samples, incubated with compound **1o** (50 μM). Images A and B show the green and red fluorescent signals dispersed throughout the cytoplasm. The nuclei are indicated by the letter ″N.″ Image C shows the normal cell morphology by phase-contrast microscopy. Reference scale bar = 25 μm.

Samples that were fixed and incubated with compound **1n** exhibited more pronounced fluorescent labeling in the contact region between cells compared to live-cell samples incubated with the same compound, as indicated by the white arrows. Additionally, a significant difference in fluorescence intensity was observed between live and fixed samples incubated with compound **1o**. Live cells displayed a much stronger emission than fixed samples, as shown in Figure 8.

**Figure 8:**
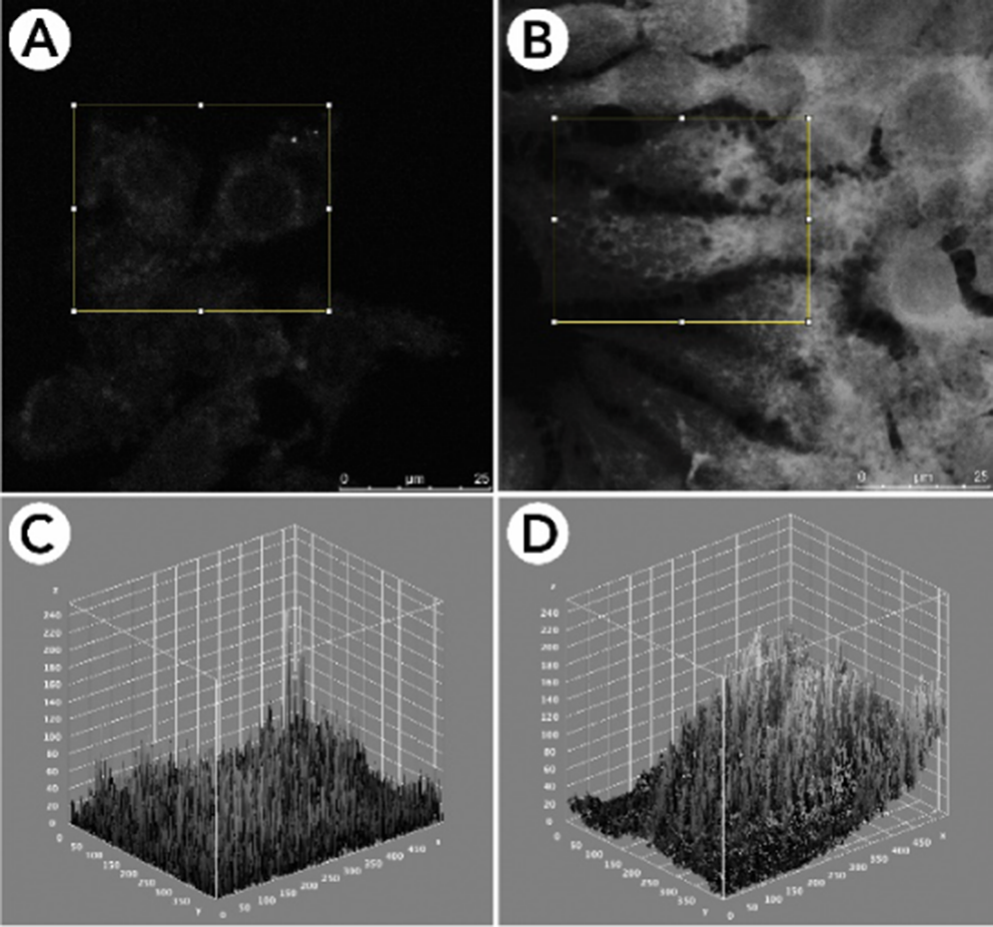
Fluorescence intensity analysis (**1o**) based on pixel values for the green channel in fixed (left) and live (right) cells. Images A and B show the ROI defined in each image (yellow rectangle). Images C and D present the 3D plot of pixel values found within the ROIs.

## Conclusions

This study demonstrated that both solvent and reagent can exhibit noninnocent behavior in the GBB MCR, significantly impacting the reaction outcome. Methanol was shown to act not only as a solvent but also as a cocatalyst, therefore influencing the kinetics and stabilizing key intermediates, as supported by kinetic modeling, MS monitoring, and DFT calculations. The results also revealed the formation of dead-end intermediates and viable alternative pathways involving methanol-assisted proton transfers and imidate intermediates. Additionally, the influence of solvent properties was quantitatively evaluated using KAT parameters, identifying α and π* as critical descriptors for enhancing reaction productivity. These results provide a molecular-level rationale for the superior performance of methanol and highlight the importance of considering specific solvent–solute interactions in the design and optimization of MCRs.

In addition to mechanistic insights, the study also led to the synthesis of fluorescent GBB adducts, two of which were successfully applied as imaging probes in both live and fixed cells, displaying favorable photophysical properties and cellular uptake. These findings reinforce the need to consider both solvent and reagent effects in the rational design of MCRs and expand their potential in synthetic and biological applications. Furthermore, additional investigations are needed regarding the possibility of an S_N_2 pathway, which may lead to a deeper understanding of the solvent’s role in this transformation and provide new perspectives for MCR reactions.

## Method

### General Procedure for the Preparation of Compounds **1a**–**1q**

In a 2.0 mL vial was added 0.5 mL of methanol. Next, 1.0 equiv (0.25 mmol) of aldehyde, 1.0 equiv (0.25 mmol) of cyclic amidine (pyridin-2-amine, pyrimidin-2-amine or benzo­[*d*]­thiazol-2-amine), 1.0 equiv (0.25 mmol) of isocyanide, and 0.1 equiv of PTSA·H_2_O (0.025 mmol) were added. The reaction mixture was kept at room temperature under magnetic stirring for 6 h. The solvent was then removed under reduced pressure and the product purified through column chromatography.

### Additional Experimental and Computational Details

We described all the corresponding procedures in the Supporting Information.

## Supplementary Material





## Data Availability

The data underlying this study are available in the published article and its Supporting Information.

## Notes

Details for all experimental procedures, additional optimization experiments, copies of ^1^H, ^13^C­{^1^H}, and ^19^F NMR and IR spectra for all compounds, and HRMS and UV spectra (PDF)
Computational details, relative and absolute energies, reaction energy profiles, and Cartesian coordinates of all computed structures (PDF)
