# Supplementary material for: Solvents’ and Reagents’ Noninnocent Roles in the Groebke–Blackburn–Bienaymé (GBB) Multicomponent Reaction: Experimental and Computational Evidence
Source: ACS Org Inorg Au. 2025 Jun 12;5(4):288–98. doi: 10.1021/acsorginorgau.5c00049 (PMC12332792; doi:10.1021/acsorginorgau.5c00049)
Supplement: Supplementary file 1 [file gg5c00049_si_001.pdf]

Solvents' and Reagents' Noninnocent Roles in the Groebke–Blackburn–Bienaymé (GBB) Multicomponent Reaction: Experimental and Computational Evidence

Marcelo H. R. Carvalho,[‡] Pedro P. De Castro,[†,*] Pedro Beck,[§] Hélio F. Dos Santos,[‡] Fabricio Machado,[§] José R. Correa,[§] Brenno A. D. Neto,[§,*] and Giovanni W. Amarante[‡,*]

[‡] Chemistry Department, Federal University of Juiz de Fora, Campus Martelos, Juiz de Fora, Minas Gerais, 36036-900, Brazil.

[†] Pharmacy Department, Federal University of Juiz de Fora – Campus Governador Valadares, Governador Valadares, Minas Gerais, 35010-180, Brazil.

[§] Laboratory of Medicinal and Technological Chemistry, University of Brasília, Chemistry Institute (IQ-UnB), Campus Universitário Darcy Ribeiro, Brasília, Distrito Federal, 70910-900, Brazil.

* pedro.possa@ufjf.br; brenno.ipi@gmail.com, giovanni.amarante@ufjf.br

Supporting Information

Table of contents

1. Experimental section........S3

1.1. General remarks........S3

1.2. Additional optimization data........S4

1.3. General procedure for the preparation of compounds 1a-1q........S5

1.4. Characterization data for compounds 1a-1q........S6

1.5. General procedure for the NMR reaction monitoring........S11

1.6. Bioimaging experiments........S12

1.7. Computational methods........S13

2. NMR, IR and HRMS spectra of compounds 1a-1q........S14

3. Mass spectrometry, UV-Vis and fluorescence emission spectra........S42

3.1. UV-Vis and fluorescence emission spectra of compounds 1n-1o........S42

3.2. Additional mass spectrometry spectra........S43

4. General overview of the evaluated mechanisms........S47

5. Energy profile (ΔG) of the evaluated mechanisms........S53

6. Imaginary frequencies for all transition states........S59

7. Electronic energies (E), Enthalpies (H) and Gibbs free energies (G) of all optimized structures........S62

8. Electronic energies (ΔE), Enthalpies (ΔH) and Gibbs free energies (ΔG) variation along all pathways........S71

9. Images of all optimized structures and selected bond lengths........S80

1. Experimental section

1.1. General remarks

All acquired chemicals were used without additional purification. Analytical Thin Layer Chromatography was conducted on TLC plates (silica gel 60 F254) and observed using a UV lamp. Yields are based on compounds that have been chromatographically purified and are spectroscopically pure. ^{1}H NMR was acquired at 400, 500 or 600 MHz; ^{19}F NMR was acquired at 376 MHz; ^{13}C{^{1}H} NMR spectra were recorded at frequencies of 100, 125 or 150 MHz. Chemical shifts for ^{1}H and ^{13}C{^{1}H} NMR are expressed as δ (parts per million) in relation to the solvent signal ($CDCl_3$ at 7.26 ppm (singlet) and 77.160 ppm (triplet), respectively). Chemical shifts are reported employing the following abbreviation standard: s, singlet; d, doublet; dd, doublet of doublets; ddd, doublet of doublet of doublets; t, triplet; td, triplet of doublets; q, quartet; quint, quintet; sext, sextet; br, broad; and m, multiplet. High-resolution mass spectra (HRMS) were recorded on a Triple Quad with a TOF analyzer. Electrospray ionization mass spectrometry ESI-MS(/MS) measurements were performed in the positive ion mode, using a *m/z* 50-1000 range.

1.2. Additional optimization data

Table S1. Additional optimization of the Groebke–Blackburn–Bienaymé reaction

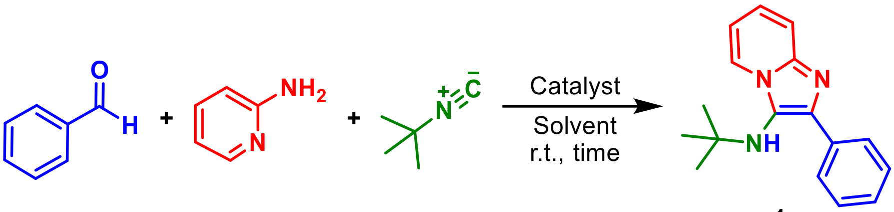

1a

Entry	Time (h)	Catalyst	Solvent	Conversion[a] [%]
**1**	6	Cx_4SO_3H (5 mol %)	MeOH	72
**2**	6	HCl (10 mol %)	MeOH	83
**3**	6	H_2SO_4 (10 mol %)	MeOH	70
**4**	6	HPW (10 mol %)	MeOH	62
**5**	6	MSI_3PW (5 mol %)	MeOH	58
**6**	6	DIPEA (10 mol %)	MeOH	-
**7**	6	Morpholine (10 mol %)	MeOH	-
**8**	6	DABCO (10 mol %)	MeOH	7

[a] Conversion calculated through the ^1H NMR analysis of the crude reaction mixture.

α	β	π*	Solvent
0.98	0.66	0.60	Methanol
0.86	0.75	0.54	Ethanol
0.76	0.84	0.48	Isopropanol
0.84	0.84	0.47	Butanol
0.68	0.93	0.41	t-Butanol
0.77	0.81	0.40	Octanol
0.19	0.40	0.75	MeCN
0.13	0.10	0.82	DCM

KAT values used in this work.

1.3. General procedure for the preparation of compounds 1a-1q

1.3.1. Reactions carried out in 0.25 mmol scale

In a 2.0 mL vial was added 0.5 mL of methanol. Next, 1.0 equivalent (0.25 mmol) of aldehyde, 1.0 equivalent (0.25 mmol) of cyclic amidine (pyridin-2-amine, pyrimidin-2-amine or benzo[*d*]thiazol-2-amine), 1.0 equivalent (0.25 mmol) of isocyanide, and 0.1 equivalent of PTSA·H_2O (0.025 mmol) were added. The reaction mixture was kept at room temperature under magnetic stirring for 6 hours. The solvent was then removed under reduced pressure and the product purified through column chromatography.

1.3.2. Reaction carried out in 2 mmol scale for compound 1a

In a 10.0 mL round bottom flask was added 4 mL of methanol. Next, 1.0 equivalent (2 mmol, 204.1 μL) of benzaldehyde, 1.0 equivalent (2 mmol, 188.2 mg) of 2-aminopyridine, 1.0 equivalent (2 mmol, 226.2 μL) of *tert*-butyl isocyanide, and 0.1 equivalent of PTSA·H_2O (0.2 mmol, 38.1 mg) were added. The reaction mixture was kept at room temperature under magnetic stirring for 6 hours. The solvent was then removed under reduced pressure and the product purified through column chromatography using an isocratic *2:1* v/v solution of hexanes/ethyl acetate as eluent, affording the product as a white solid (419 mg, 79% yield).

1.4. Characterization data for compounds 1a-1q

N-(tert-butyl)-2-phenylimidazo[1,2-a]pyridin-3-amine (**1a**):[1] The compound was purified through column chromatography using an isocratic *2:1* v/v solution of hexanes/ethyl acetate as eluent, affording the product as a white solid (60 mg, 90% - 0.25 mmol scale; 419 mg, 79% - 2 mmol scale). m.p. 158-159 °C. ^{1}H NMR ($CDCl_3$, 500 MHz): δ 8.20 (d, 1H, *J* = 6.8 Hz), 7.89 (d, 2H, *J* = 7.6 Hz), 7.53 (d, 1H, *J* = 9.0 Hz), 7.41 (t, 2H, *J* = 7.6 Hz), 7.29 (t, 1H, *J* = 7.4 Hz), 7.10 (t, 1H, *J* = 7.5 Hz), 6.73 (t, 1H, *J* = 6.8 Hz), 3.22 (br, 1H), 1.01 (s, 9H). ^{13}C{^{1}H} NMR ($CDCl_3$, 125 MHz): δ 141.9, 139.1, 134.8, 130.0, 128.5, 128.3, 127.7, 124.7, 123.7, 117.2, 111.8, 56.6, 30.4. IR (ATR, cm^{-1}):3313; 2965; 1439; 1387; 1360; 1335; 1209; 746; 694.

N-(tert-butyl)-2-(2-chlorophenyl)imidazo[1,2-a]pyridin-3-amine (**1b**):[2] The compound was purified through column chromatography using an isocratic *2:1* v/v solution of hexanes/ethyl acetate as eluent, affording the product as a yellow solid (65 mg, 87%). m.p. 116-118 °C. ^{1}H NMR ($CDCl_3$, 600 MHz): δ 8.33 (d, 1H, *J* = 6.9 Hz), 7.73 (dd, 1H, *J* = 7.6 Hz, *J* = 1.4 Hz), 7.61 (d, 1H, *J* = 9.0 Hz), 7.45 (d, 1H, *J* = 8.0 Hz), 7.37 (t, 1H, *J* = 7.4 Hz), 7.32 (td, 1H, *J* = 7.5 Hz, *J* = 1.4 Hz), 7.20 (t, 1H, *J* = 7.8 Hz), 6.83 (t, 1H, *J* = 6.8 Hz), 3.21 (br, 1H), 0.92 (s, 9H). ^{13}C{^{1}H} NMR ($CDCl_3$, 150 MHz): δ 142.0, 136.9, 134.2, 132.9, 132.5, 129.7, 129.5, 127.3, 125.3, 125.0, 123.8, 117.2, 111.9, 56.0, 30.0. IR (ATR, cm^{-1}): 3234; 2962; 1332; 1224; 747; 736.

N-(tert-butyl)-2-(3-chlorophenyl)imidazo[1,2-a]pyridin-3-amine (**1c**):[3] The compound was purified through column chromatography using an isocratic *2:1* v/v solution of hexanes/ethyl acetate as eluent, affording the product as a yellow solid (66 mg, 87%). m.p. 126-129 °C. ^{1}H NMR ($CDCl_3$, 600 MHz): δ 8.29 (d, 1H, *J* = 5.8 Hz), 8.04 (s, 1H), 7.90 (d, 1H, *J* = 6.4 Hz), 7.67 (d, 1H, *J* = 7.5 Hz), 7.35 (t, 1H, *J* = 6.5 Hz), 7.28 (d, 1H, *J* = 6.7 Hz), 7.24 (t, 1H, *J* = 6.7 Hz), 6.87 (t, 1H, *J* = 6.7 Hz), 3.40 (br, 1H), 1.07 (s, 9H). ^{13}C{^{1}H} NMR ($CDCl_3$, 150 MHz): δ 140.9, 134.4, 129.9, 128.2, 128.1, 126.6, 126.3, 125.2, 124.2, 124.0, 116.4, 56.8, 30.6. IR (ATR, cm^{-1}): 3299; 2962; 1368; 1337; 1186; 754; 733.

N-(tert-butyl)-2-(4-chlorophenyl)imidazo[1,2-a]pyridin-3-amine (**1d**):[3] The compound was purified through column chromatography using an isocratic *2:1* v/v solution of

hexanes/ethyl acetate as eluent, affording the product as a white solid (52 mg, 69%). m.p. 142-144 °C. ^{1}H NMR ($CDCl_3$, 600 MHz): δ 8.28 (d, 1H, *J* = 6.8 Hz), 7.94 (d, 2H, *J* = 7.8 Hz), 7.62 (d, 1H, *J* = 8.9 Hz), 7.35 (d, 2H, *J* = 7.6 Hz), 7.24 (t, 1H, *J* = 7.7 Hz), 6.88 (t, 1H, *J* = 6.8 Hz), 3.48 (br, 1H), 1.04 (s, 9H). $^{13}C\{^{1}H\}$ NMR ($CDCl_3$, 150 MHz): δ 141.7, 137.6, 133.6, 133.0, 129.5, 128.6, 125.2, 123.8, 123.7, 117.0, 112.1, 56.7, 30.5. IR (ATR, cm^{-1}): 3275; 2963; 1361; 1330; 1207; 830; 751; 730.

N-(tert-butyl)-2-(4-fluorophenyl)imidazo[1,2-a]pyridin-3-amine (**1e**):[3] The compound was purified through column chromatography using an isocratic *2:1* v/v solution of hexanes/ethyl acetate as eluent, affording the product as a white solid (57 mg, 81%). m.p. 157-159 °C. ^{1}H NMR ($CDCl_3$, 600 MHz): δ 8.29 (d, 1H, *J* = 6.9 Hz), 7.95 (dd, 2H, *J* = 8.3 Hz, *J* = 5.6 Hz), 7.64 (d, 1H, *J* = 8.9 Hz), 7.23 (t, 1H, *J* = 7.7 Hz), 7.09 (t, 2H, *J* = 8.6 Hz), 6.87 (t, 1H, *J* = 7.5 Hz), 3.41 (br, 1H), 1.03 (s, 9H). $^{13}C\{^{1}H\}$ NMR ($CDCl_3$, 100 MHz): δ 162.3 (d, *J* = 245.0 Hz), 142.2, 138.9, 131.6 (d, *J* = 3.2 Hz), 129.9 (d, *J* = 8.0 Hz), 124.2, 123.5, 123.3, 117.4, 115.3 (d, *J* = 21.3 Hz), 111.5, 56.5, 30.5. ^{19}F NMR ($CDCl_3$, 376 MHz): δ −114.8. IR (ATR, cm^{-1}): 3278; 2966; 1499; 1358; 1327; 1213; 840; 751; 730.

2-(4-bromophenyl)-N-(tert-butyl)imidazo[1,2-a]pyridin-3-amine (**1f**):[3] The compound was purified through column chromatography using an isocratic *2:1* v/v solution of hexanes/ethyl acetate as eluent, affording the product as a white solid (54 mg, 63%). m.p. 142-143 °C. ^{1}H NMR ($CDCl_3$, 600 MHz): δ 8.23 (d, 1H, *J* = 6.8 Hz), 7.87 (d, 2H, *J* = 8.4 Hz), 7.57 (d, 1H, *J* = 8.9 Hz), 7.52 (d, 2H, *J* = 8.4 Hz), 7.19 (t, 1H, *J* = 7.5 Hz), 6.83 (t, 1H, *J* = 6.8 Hz), 3.27 (br, 1H), 1.04 (s, 9H). $^{13}C\{^{1}H\}$ NMR ($CDCl_3$, 150 MHz): δ 141.4, 132.2, 131.5, 129.7, 128.8, 125.4, 123.7, 123.6, 121.8, 116.7, 112.2, 56.6, 30.4. IR (ATR, cm^{-1}): 3282; 2963; 1437; 1360; 1334; 1204; 1008; 833; 751; 730.

N-(tert-butyl)-2-(4-methoxyphenyl)imidazo[1,2-a]pyridin-3-amine (**1g**):[3] The compound was purified through column chromatography using an isocratic *2:1* v/v solution of hexanes/ethyl acetate as eluent, affording the product as a yellow solid (60 mg, 81%). m.p. 130-132 °C. ^{1}H NMR ($CDCl_3$, 600 MHz): δ 8.24 (d, 1H, *J* = 6.8 Hz), 7.86 (d, 2H, *J* = 8.8 Hz), 7.59 (d, 1H, *J* = 8.8 Hz), 7.17-7.14 (m, 1H), 6.95 (d, 2H, *J* = 8.8 Hz), 6.80 (t, 1H, *J* = 8.8 Hz), 3.84 (s, 3H), 3.22 (br, 1H), 1.04 (s, 9H). $^{13}C\{^{1}H\}$ NMR ($CDCl_3$, 150 MHz): δ 159.3, 141.3, 138.3, 129.5, 126.7, 125.0, 123.7, 123.1, 116.7, 113.9, 111.9, 56.5,

55.4, 30.5. IR (ATR, cm^{-1}): 3319; 2963; 1501; 1361; 1296; 1251; 1217; 1029; 830; 751; 730.

N-(tert-butyl)-2-(3-methoxyphenyl)imidazo[1,2-a]pyridin-3-amine (**1h**):[4] The compound was purified through column chromatography using an isocratic *2:1* v/v solution of hexanes/ethyl acetate as eluent, affording the product as a yellow solid (64 mg, 86%). m.p. 109-110 °C. ^{1}H NMR ($CDCl_3$, 600 MHz): δ 8.23 (dd, 1H, J = 6.8 Hz, J = 1.0 Hz), 7.57 (dd, 1H, J = 9.0 Hz, J = 0.8 Hz), 7.51 (t, 1H, J = 2.1 Hz), 7.47 (d, 1H, J = 7.6 Hz), 7.30 (t, 1H, J = 8.0 Hz), 7.14 (ddd, 1H, J = 8.8 Hz, J = 6.6 Hz, J = 1.3 Hz), 6.85 (ddd, 1H, J = 8.2 Hz, J = 2.6 Hz, J = 0.8 Hz), 6.78 (td, 1H, J = 6.8 Hz, J = 1.0 Hz), 3.87 (s, 3H), 1.03 (s, 9H). $^{13}C\{^{1}H\}$ NMR ($CDCl_3$, 150 MHz): δ 159.7, 141.6, 138.6, 135.9, 129.3, 124.9, 123.8, 123.7, 120.7, 117.0, 114.0, 113.3, 111.9, 56.6, 55.5, 30.4. IR (ATR, cm^{-1}): 3277; 2969; 1611; 1581; 1340; 1289; 1204; 1042; 775; 751; 734.

N-(tert-butyl)-2-(4-(trifluoromethyl)phenyl)imidazo[1,2-a]pyridin-3-amine (**1i**): The compound was purified through column chromatography using an isocratic *2:1* v/v solution of hexanes/ethyl acetate as eluent, affording the product as a white solid (52 mg, 62%). m.p. 122-123 °C. ^{1}H NMR ($CDCl_3$, 600 MHz): δ 8.19 (d, 1H, J = 6.9 Hz), 8.11 (d, 2H, J = 8.0 Hz), 7.64 (d, 2H, J = 8.2 Hz), 7.54 (d, 1H, J = 9.0 Hz), 7.17-7.14 (m, 1H), 6.79 (t, 1H, J = 6.7 Hz), 1.04 (s, 9H). $^{13}C\{^{1}H\}$ NMR ($CDCl_3$, 150 MHz): δ 142.2, 138.6, 137.7, 129.3 (q, J = 32.0 Hz), 128.3, 125.3 (q, J = 3.7 Hz), 125.0, 124.4 (q, J = 270.3 Hz), 124.3, 123.6, 117.4, 112.0, 56.7, 30.5. ^{19}F NMR ($CDCl_3$, 376 MHz): δ −62.4. IR (ATR, cm^{-1}): 3274; 2961; 1317; 1162; 1118; 847; 737. HRMS (ESI-TOF): *m/z* $[M + H]^+$ Calcd for $C_{18}H_{19}F_3N_3$ 334.1526; Found 334.1530.

N-(tert-butyl)-2-(furan-2-yl)imidazo[1,2-a]pyridin-3-amine (**1j**):[5] The compound was purified through column chromatography using an isocratic *2:1* v/v solution of hexanes/ethyl acetate as eluent, affording the product as a yellow solid (59 mg, 93%). m.p. 109-110 °C. ^{1}H NMR ($CDCl_3$, 600 MHz): δ 8.32 (d, 1H, J = 6.9 Hz), 7.68 (d, 1H, J = 9.0 Hz), 7.51 (dd, 1H, J = 1.6 Hz, J = 0.7 Hz), 7.27 (t, 1H, J = 8.6 Hz), 7.12 (d, 1H, J = 3.1 Hz), 6.88 (t, 1H, J = 6.8 Hz), 6.54 (dd, 1H, J = 3.3 Hz, J = 1.7 Hz), 1.17 (s, 9H). $^{13}C\{^{1}H\}$ NMR ($CDCl_3$, 150 MHz): δ 148.3, 142.1, 141.2, 128.5, 126.5, 124.2, 124.1, 116.2, 112.7, 112.7, 112.0, 109.3, 56.7, 30.0. IR (ATR, cm^{-1}): 3319; 2966; 1344; 1193; 1002; 751; 720.

N-(tert-butyl)-2-(pyridin-2-yl)imidazo[1,2-a]pyridin-3-amine (**1k**):[6] The compound was purified through column chromatography using an isocratic *2:1* v/v solution of hexanes/ethyl acetate as eluent, affording the product as a beige solid (59 mg, 89%). m.p. 89-91 °C. ^{1}H NMR (CDCl$_3$, 600 MHz): δ 8.58-8.57 (m, 1H), 8.33 (d, 1H, *J* = 6.9 Hz), 8.26 (d, 1H, *J* = 8.0 Hz), 7.79 (td, 1H, *J* = 7.8 Hz, *J* = 1.8 Hz), 7.67 (d, 1H, *J* = 9.1 Hz), 7.23-7.17 (m, 2H), 6.83 (t, 1H, *J* = 6.7 Hz), 1.14 (s, 9H). ^{13}C{^{1}H} NMR (CDCl$_3$, 150 MHz): δ 153.6, 148.5, 141.0, 137.0, 133.1, 129.1, 127.5, 124.5, 122.3, 121.9, 116.9, 112.3, 57.4, 30.1. IR (ATR, cm^{-1}): 3299; 2966; 1587; 1341; 1200; 775; 748; 688.

2-(benzo[d][1,3]dioxol-5-yl)-N-(tert-butyl)imidazo[1,2-a]pyridin-3-amine (**1l**): The compound was purified through column chromatography using an isocratic *2:1* v/v solution of hexanes/ethyl acetate as eluent, affording the product as a white solid (46 mg, 60%). m.p. 132-135 °C. ^{1}H NMR (CDCl$_3$, 600 MHz): δ 8.22 (d, 1H, *J* = 6.8 Hz), 7.57 (d, 1H, *J* = 8.9 Hz), 7.46 (d, 1H, *J* = 1.3 Hz), 7.41 (dd, 1H, *J* = 8.0 Hz, *J* = 1.5 Hz), 7.15 (t, 1H, *J* = 7.8 Hz), 6.86 (d, 1H, *J* = 8.0 Hz), 6.79 (t, 1H, *J* = 6.7 Hz), 3.15 (br, 1H), 1.05 (s, 9H). ^{13}C{^{1}H} NMR (CDCl$_3$, 150 MHz): δ 147.8, 147.3, 141.4, 138.5, 128.5, 124.9, 123.6, 123.2, 122.2, 116.8, 111.9, 108.8, 108.4, 101.2, 56.6, 30.5. IR (ATR, cm^{-1}): 3295; 2969; 2873; 1451; 1361; 1237; 1042; 751; 737. HRMS (ESI-TOF): *m/z* [M + H]$^+$ Calcd for $C_{18}H_{20}N_3O_2$ 310.1550; Found 310.1556.

N-(tert-butyl)-2-butylimidazo[1,2-a]pyridin-3-amine (**1m**): The compound was purified through column chromatography using an isocratic 3*:1* v/v solution of hexanes/ethyl acetate as eluent, affording the product as a yellow solid (53 mg, 87%). m.p. 89-90 °C. ^{1}H NMR (CDCl$_3$, 600 MHz): δ 8.17 (d, 1H, *J* = 5.6 Hz), 7.53 (d, 1H, *J* = 8.9 Hz), 7.12 (t, 1H, *J* = 7.4 Hz), 6.76 (t, 1H, *J* = 6.7 Hz), 2.75-2.72 (m, 3H), 1.79 (quint, 2H, *J* = 7.7 Hz), 1.40 (sext, 2H, *J* = 7.5 Hz), 1.19 (s, 9H), 0.94 (t, 3H, *J* = 7.4 Hz). ^{13}C{^{1}H} NMR (CDCl$_3$, 150 MHz): δ 141.5, 140.5, 124.4, 123.5, 123.4, 116.4, 111.5, 55.7, 31.7, 30.5, 27.3, 23.1, 14.1. IR (ATR, cm^{-1}): 3234; 2963; 1340; 1119; 754; 737. HRMS (ESI-TOF): *m/z* [M + H]$^+$ Calcd for $C_{15}H_{24}N_3$ 246.1965; Found 246.1964.

N-(tert-butyl)-2-(4-methoxyphenyl)imidazo[1,2-a]pyrimidin-3-amine (**1n**):[7] The compound was purified through column chromatography using an isocratic *2:1* v/v solution of hexanes/ethyl acetate as eluent, affording the product as a yellow oil (22 mg,

30%). ^{1}H NMR ($CDCl_3$, 600 MHz): δ 8.22-8-21 (m, 2H), 7.38 (d, 2H, *J* = 8.6 Hz), 7.07 (d, 2H, *J* = 8.7 Hz), 6.68 (dd, 1H, *J* = 6.4 Hz, *J* = 4.6 Hz), 3.87 (s, 3H), 3.79 (br, 1H), 1.48 (s, 9H). ^{13}C{^{1}H} NMR ($CDCl_3$, 150 MHz): δ 159.3, 151.1, 146.2, 144.7, 130.0, 127.0, 120.9, 115.5, 113.7, 107.7, 55.5, 52.5, 30.2. IR (ATR, cm^{-1}): 3407; 2961; 1553; 1515; 1245; 1172; 751.

N-(tert-butyl)-2-phenylimidazo[1,2-a]pyrimidin-3-amine (**1o**):[8] The compound was purified through column chromatography using an isocratic *2:1* v/v solution of hexanes/ethyl acetate as eluent, affording the product as a yellow solid (14 mg, 21%). m.p. 124-126 °C. ^{1}H NMR ($CDCl_3$, 400 MHz): δ 8.34 (dd, 1H, *J* = 6.6 Hz, *J* = 1.9 Hz), 8.24 (d, 1H, *J* = 4.4 Hz, *J* = 1.9 Hz), 7.56-7.46 (m, 4H), 7.39-7.34 (m, 1H), 6.69 (dd, 1H, *J* = 6.6 Hz, *J* = 4.4 Hz), 1.51 (s, 9H). ^{13}C{^{1}H} NMR ($CDCl_3$, 100 MHz): δ 151.4, 145.1, 130.1, 130.0, 129.1, 128.3, 128.0, 127.7, 127.1, 107.8, 52.5, 30.2. IR (ATR, cm^{-1}): 3418; 2959; 2919; 1557; 1437; 1180; 758; 693.

N-(tert-butyl)-2-phenylbenzo[d]imidazo[2,1-b]thiazol-3-amine (**1p**):[9] The compound was purified through column chromatography using an isocratic *9:1* v/v solution of hexanes/ethyl acetate as eluent, affording the product as a yellow solid (25 mg, 31%). m.p. 169-172 °C. ^{1}H NMR ($CDCl_3$, 600 MHz): δ 8.37 (d, 1H, *J* = 8.1 Hz), 7.76 (d, 2H, *J* = 7.3 Hz), 7.65 (d, 1H, *J* = 7.5 Hz), 7.43-7.38 (m, 3H), 7.32 (t, 1H, *J* = 7.7 Hz), 7.28 (t, 1H, *J* = 7.4 Hz), 3.32 (br, 1H), 1.04 (s, 9H). ^{13}C{^{1}H} NMR ($CDCl_3$, 150 MHz): δ 143.9, 140.2, 134.3, 133.7, 130.5, 128.5, 127.9, 127.5, 125.7, 124.7, 124.1, 115.0, 56.8, 30.0. IR (ATR, cm^{-1}): 3319; 2961; 1215; 1027; 832; 753; 735.

N-benzyl-2-phenylimidazo[1,2-a]pyridin-3-amine (**1q**):[10] The compound was purified through column chromatography using an isocratic *2:1* v/v solution of hexanes/ethyl acetate as eluent, affording the product as a yellow solid (55 mg, 73%). m.p. 117-118 °C. ^{1}H NMR ($CDCl_3$, 600 MHz): δ 7.95-7.91 (m, 3H), 7.57 (d, 1H, *J* = 9.0 Hz), 7.35 (t, 2H, *J* = 7.5 Hz), 7.25-7.18 (m, 6H), 7.09 (ddd, 1H, *J* = 10.1 Hz, *J* = 6.7 Hz, *J* = 1.1 Hz), 6.70 (td, 1H, *J* = 6.8 Hz, *J* = 0.9 Hz), 4.11 (d, 1H, *J* = 6.1 Hz), 3.85 (br, 1H). ^{13}C{^{1}H} NMR ($CDCl_3$, 150 MHz): δ 138.8, 128.9, 128.8, 128.7, 128.4, 128.3, 128.2, 128.0, 127.7, 127.1, 125.8, 122.8, 116.4, 112.7, 52.2. IR (NaCl, cm^{-1}): 3255; 3055; 3017; 1440; 1190; 733; 693.

1.5. General procedure for the NMR reaction monitoring

In a NMR tube was added 0.5 mL of $CDCl_3$, followed by the addition of methanol (none, 0.25 mmol, 1.25 mmol, 2.50 mmol, 3.75 mmol or 5.00 mol, corresponding to 0, 1, 5, 10, 15 or 20 equivalent, respectively). Next, 1.0 equivalent (0.25 mmol) of benzaldehyde, 1.0 equivalent (0.25 mmol) of 2-aminopyridine, and 1.0 equivalent (0.25 mmol) of *tert*-butyl isocyanide were added. The reaction mixture was kept at room temperature and was periodically monitored through the acquisition of ^{1}H NMR spectra.

1.6. Bioimaging experiments

1.6.1. Compounds Solutions

The compounds 1n and 1o were diluted to 50 mM in Dulbecco's Modified Eagle Medium (DMEM) with 0.1% DMSO, supplemented with 10% fetal calf serum.

1.6.2. Cell Lineage Maintenance

MCF-7 cells (human mammary adenocarcinoma cells) were used. The cell samples were maintained according to ATCC (American Type Culture Collection) recommendations at 37 °C in a 5% CO_2 atmosphere.

1.6.3. Evaluation Assays

The cells were seeded on 13 mm round glass coverslips at the bottom of a 24-well plate, allowed to adhere overnight, and washed three times with serum-free medium to remove non-adherent cells. After reaching 75% confluence, the samples were washed three times in Phosphate Buffered Saline (PBS) 1X (pH 7.4) at 37 °C. The samples were then divided into two groups (live and fixed cells), and each group was incubated for 30 minutes with the compounds as follows. The live cells were first washed three times in PBS 1X (pH 7.4) at 37°C. Half of the sample was incubated with compound 1n, and the other half was incubated with compound 1o for 30 minutes at 37 °C. The samples were then washed three times in PBS 1X at 37 °C and fixed in 3.7% formaldehyde at room temperature for 30 minutes. The fixed group was washed three times in PBS 1X at 37 °C, fixed for 30 minutes in 3.7% formaldehyde, and then incubated with compounds 1n and 1o for 30 minutes, as previously described for the live cell group. All samples were washed again three times in PBS 1X (pH 7.4) at room temperature, and the coverslips were mounted on glass slides using ProLong Gold Antifade (Invitrogen, OR, USA) according to the manufacturer's recommendations. The negative control consisted of cell samples maintained only in cell medium supplemented with 10% fetal calf serum and 0.1% DMSO, the diluent solution used. The samples were analyzed using a TCS-SP5 Leica Confocal microscope. All assays were performed in triplicate, with three repetitions conducted for each experimental condition.

1.7. Computational methods

All calculations were carried out using the Gaussian 09 package (revision D.01).[11] The density functional theory (DFT) using the hybrid M06-2X functional was used in all calculations. The optimization of all molecular complexes (MCs) and transition states (TSs) was performed at the gas phase using the 6-31++G(d,p) basis set (grid=ultrafine). The choice for this theory level was based on previous studies.[12,13] The TSs were optimized using the Berny algorithm, presented a single imaginary frequency and were fully characterized through the analysis of the intrinsic reaction coordinate (IRC). The vibrational analysis of each structure was carried out to determine the thermal corrections to enthalpy and Gibbs free energy at a temperature of 298.15 K (25 °C) and a pressure of 1 atm, aiming to precisely reproduce the experimental conditions.

Next, single-point calculations were carried out using the geometries optimized at the gas phase, using the M06-2X functional, the 6-31++G(d,p) basis set, and the solvation model based on density (SMD) for methanol, dichloromethane or toluene. The Gibbs free energy of each structure in solution ($\boldsymbol{G^{\circ}_{sol}}$) was then calculated through equation (1), in which the terms are respectively the electronic energy, the solvation Gibbs free energy and the thermal correction to enthalpy and entropy.

$$\boldsymbol{G^{\circ}_{sol} = E_{gas} + G_{sol} + G^{\circ}_{T}} \qquad (1)$$

For transition states in which methanol also act as a reagent, a correction of -1.90 kcal mol^{-1} was included to consider the concentration of these solvents in the activation barriers. This correction was determined according to equation (2).

$$\boldsymbol{\Delta G^{\circ}_{sol} = \Delta G^{\circ}_{sol} - nRTln[solvent]} \qquad (2)$$

In which *n* is the number of solvent molecules involved.

2. NMR, IR and HRMS spectra of compounds 1a-1q

Figure S1. ^{1}H NMR of compound **1a** (500 MHz, $CDCl_3$).

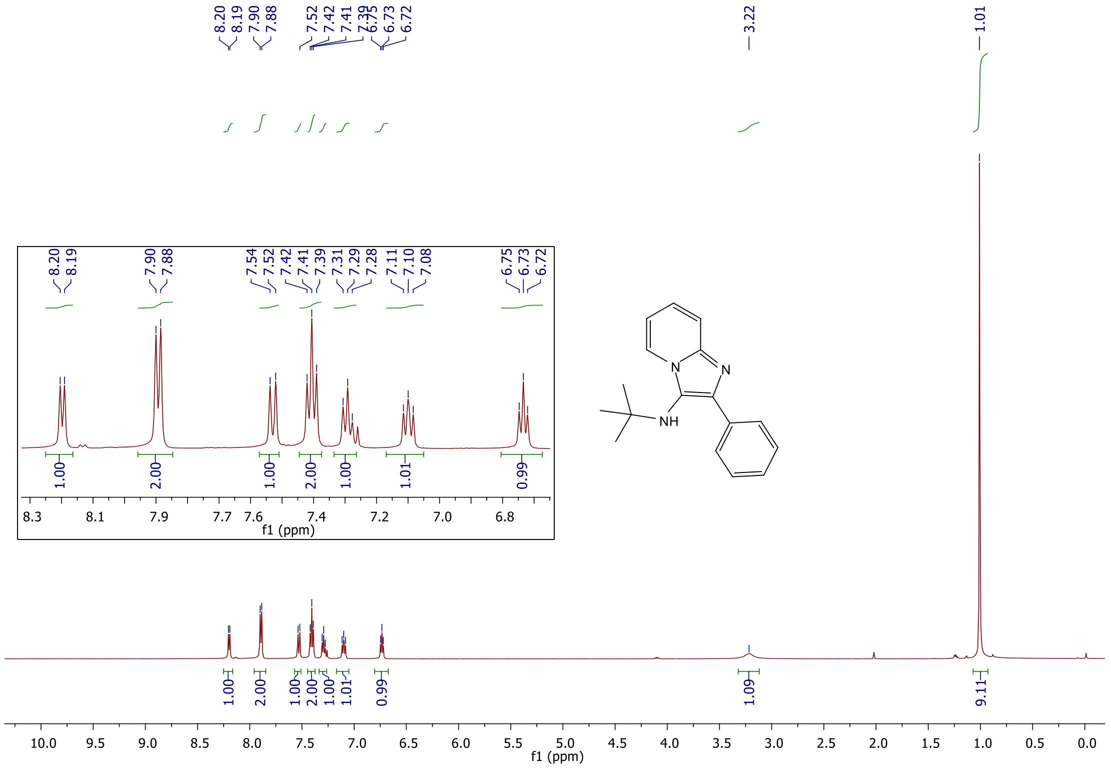

Figure S2. ^{13}C{^{1}H} NMR of compound **1a** (125 MHz, $CDCl_3$).

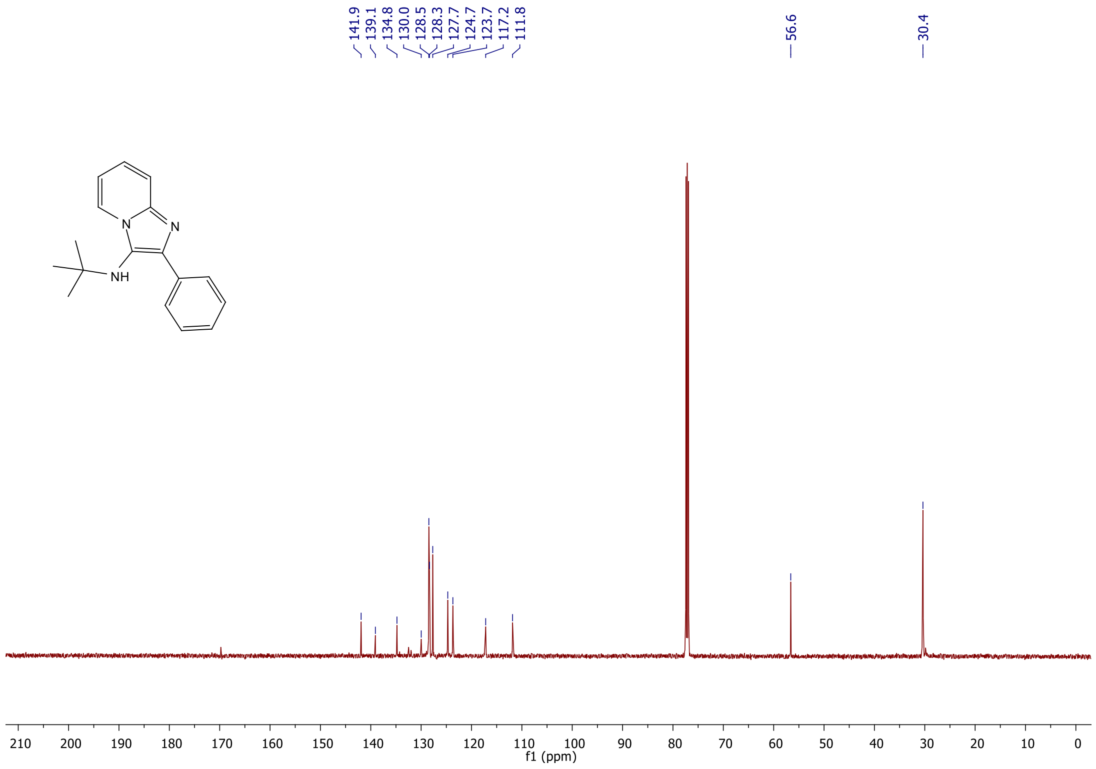

Figure S3. IR (ATR) of compound **1a**.

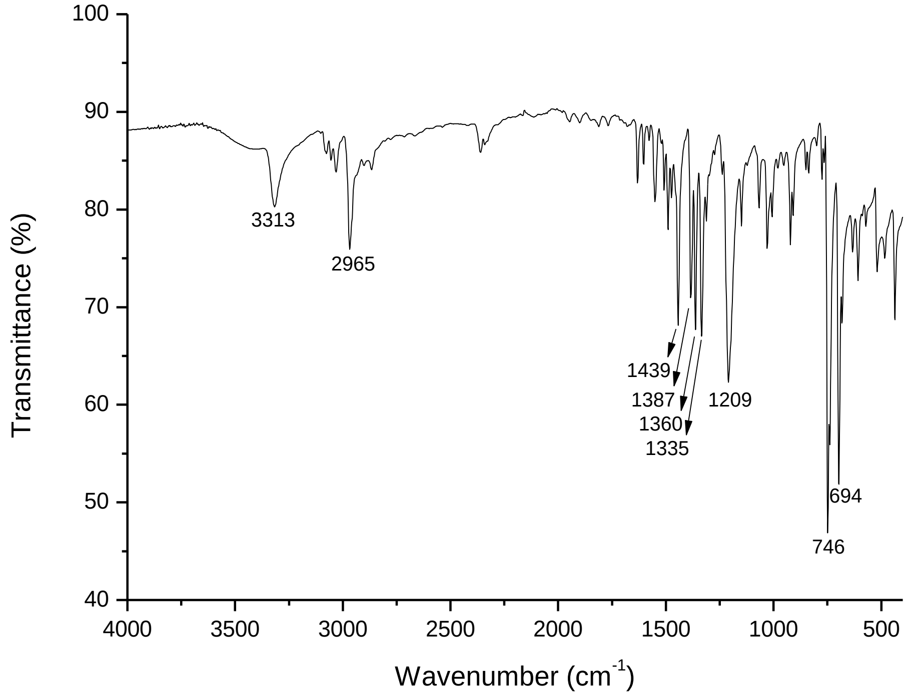

Figure S4. ^{1}H NMR of compound **1b** (600 MHz, $CDCl_3$).

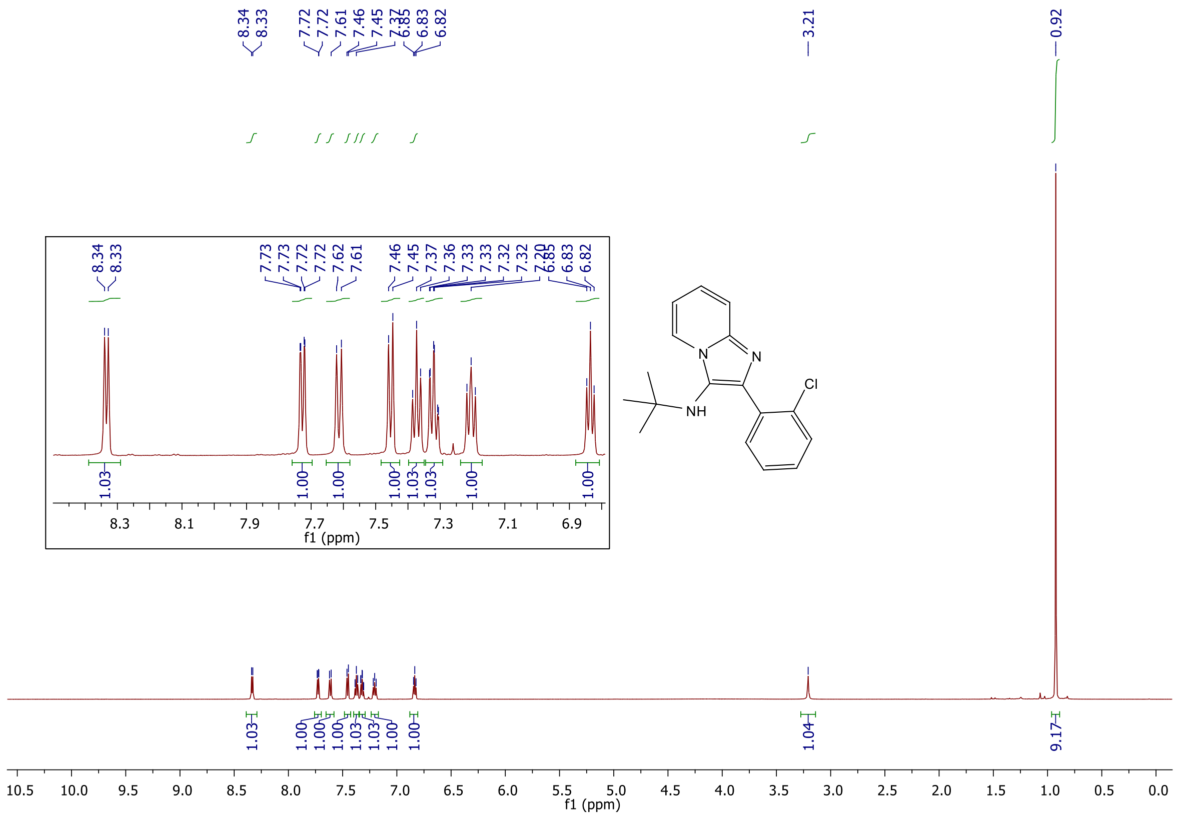

Figure S5. $^{13}C\{^1H\}$ NMR of compound **1b** (150 MHz, $CDCl_3$).

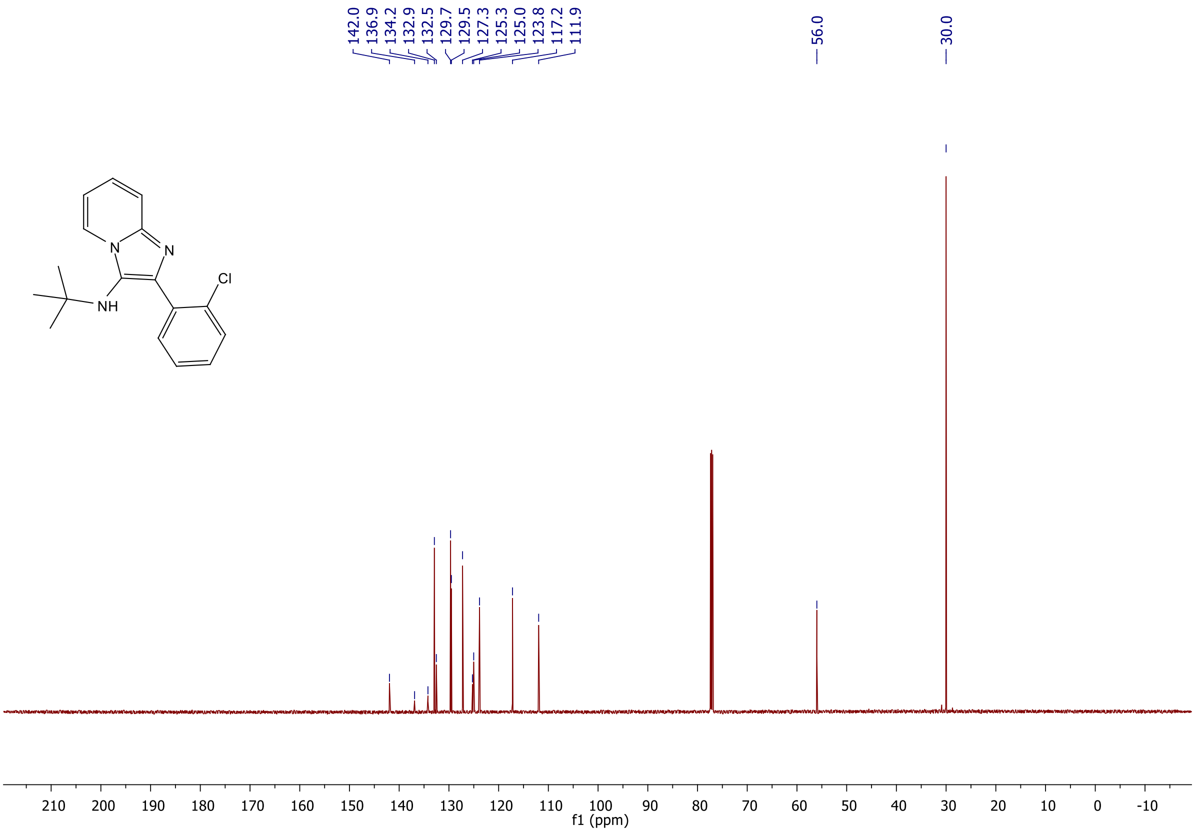

Figure S6. IR (ATR) of compound **1b.**

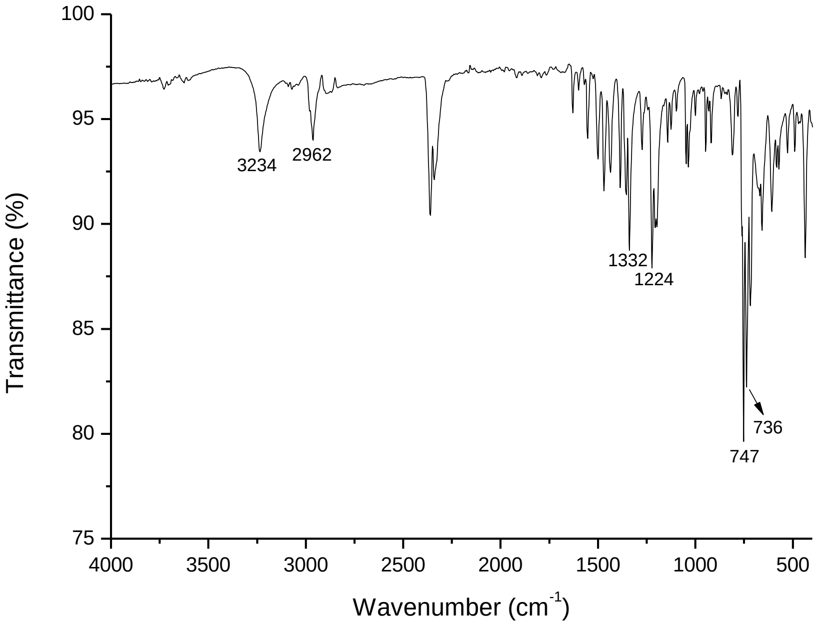

Figure S7. ^{1}H NMR of compound **1c** (600 MHz, $CDCl_3$).

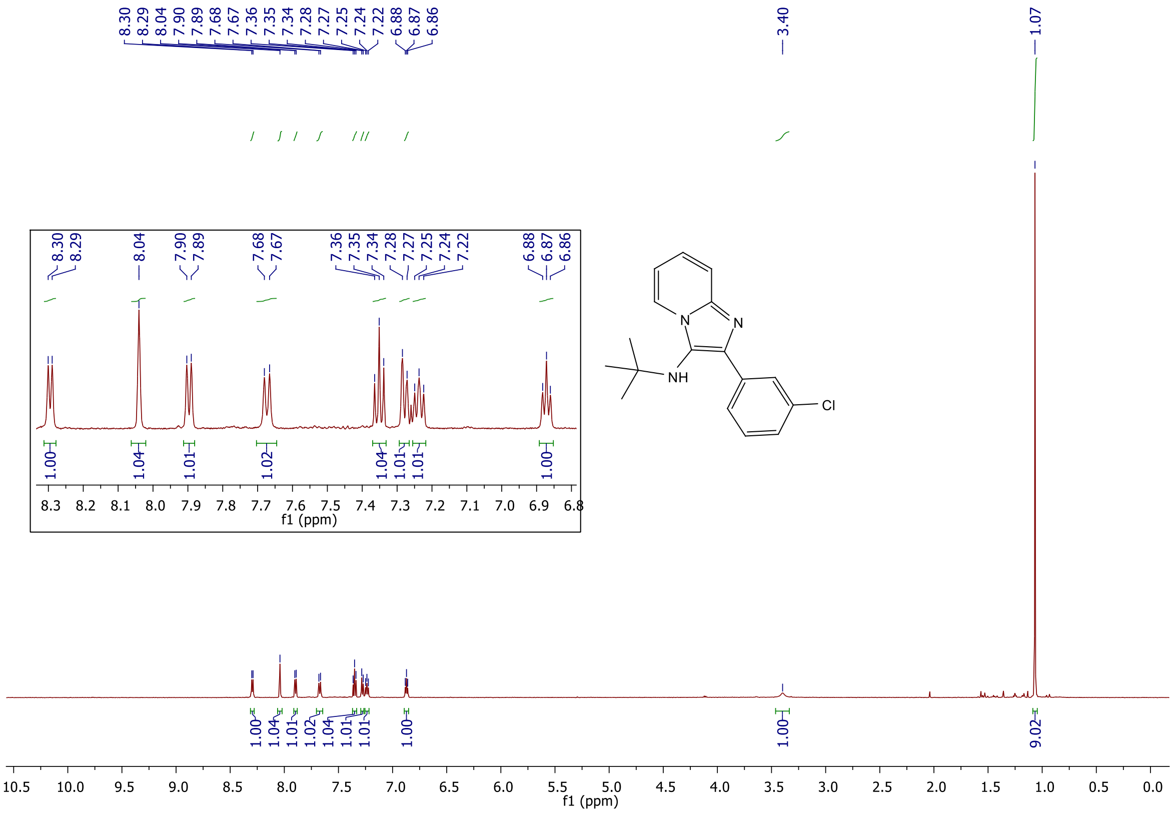

Figure S8. $^{13}C\{^{1}H\}$ NMR of compound **1c** (150 MHz, $CDCl_3$).

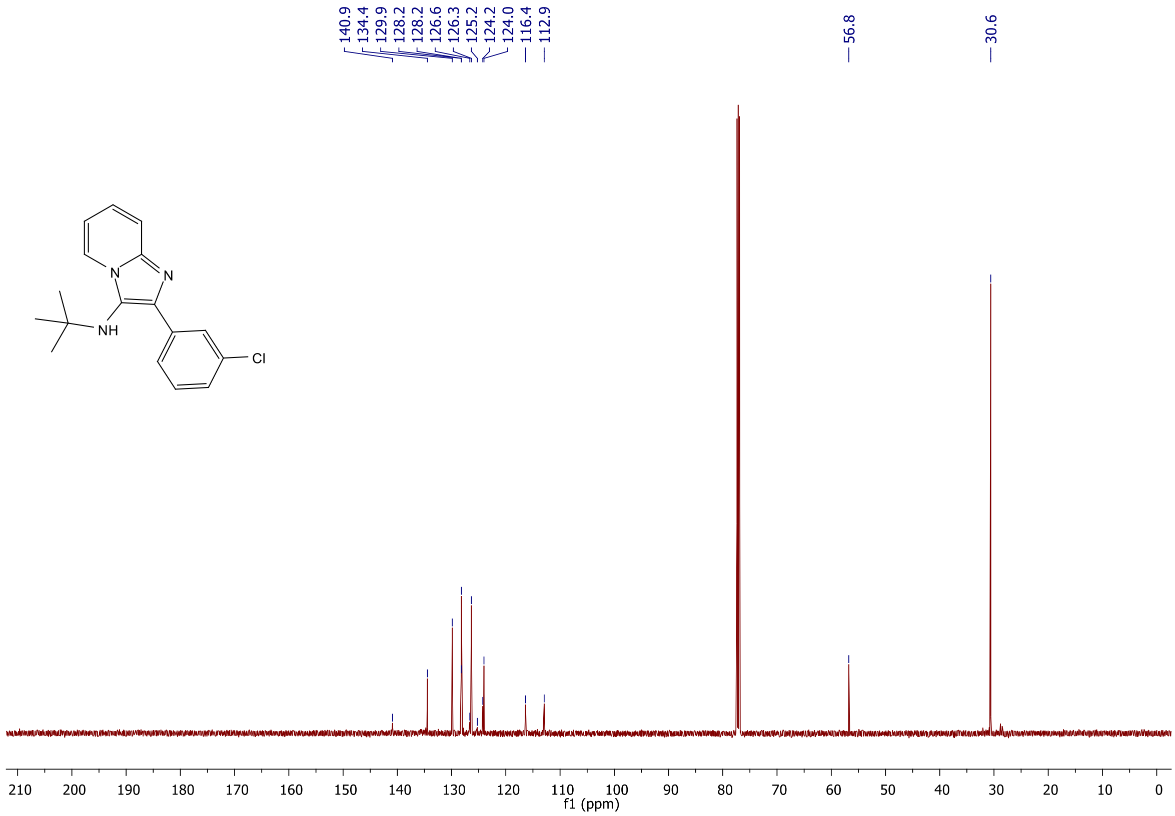

Figure S9. IR (ATR) of compound **1c**.

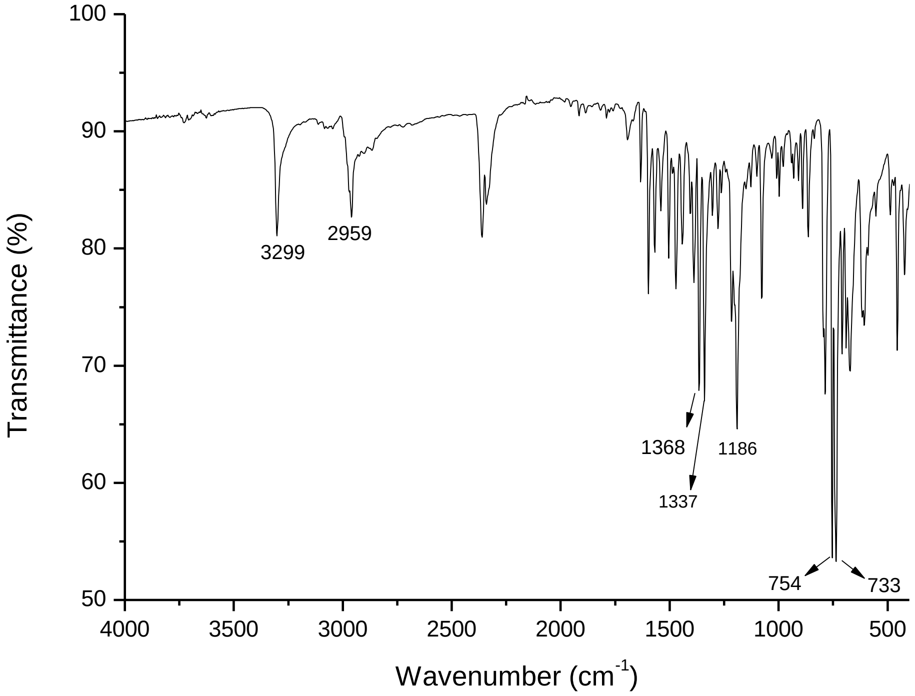

Figure S10. ^{1}H NMR of compound **1d** (600 MHz, $CDCl_3$).

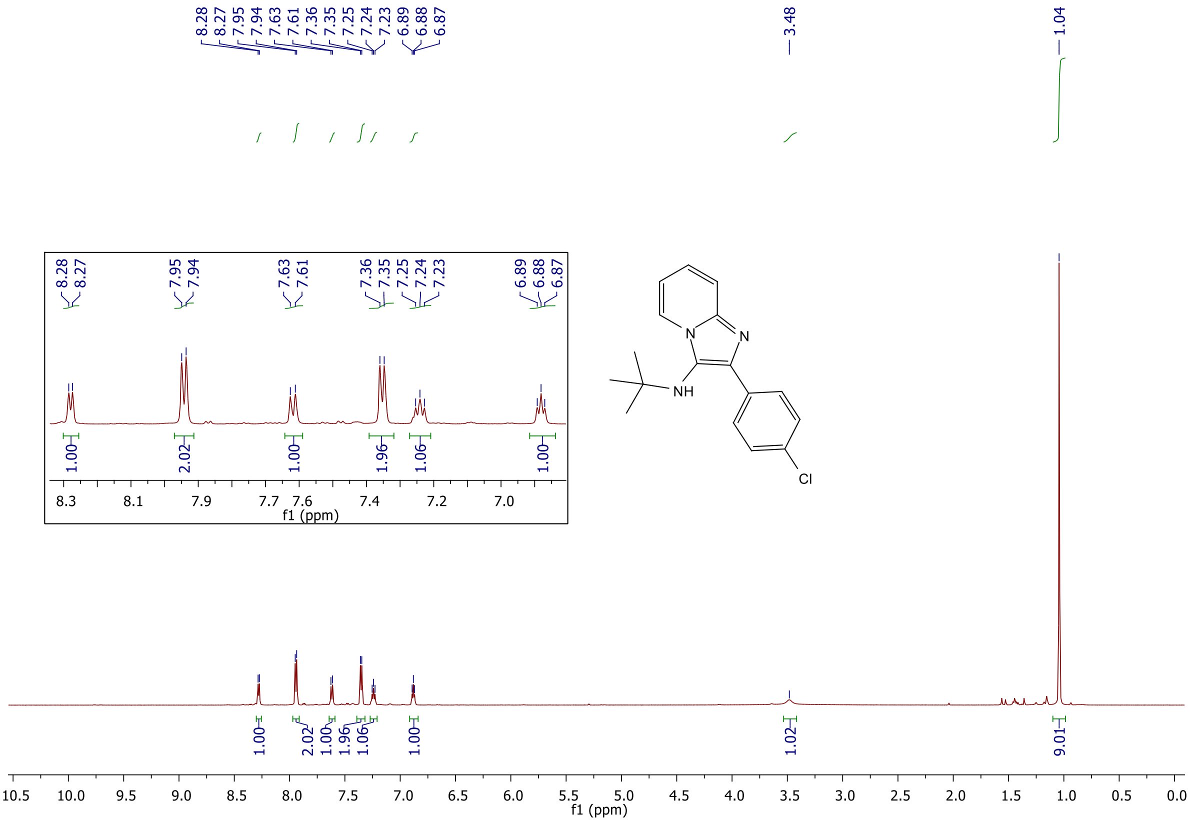

Figure S11. $^{13}C\{^{1}H\}$ NMR of compound **1d** (150 MHz, $CDCl_3$).

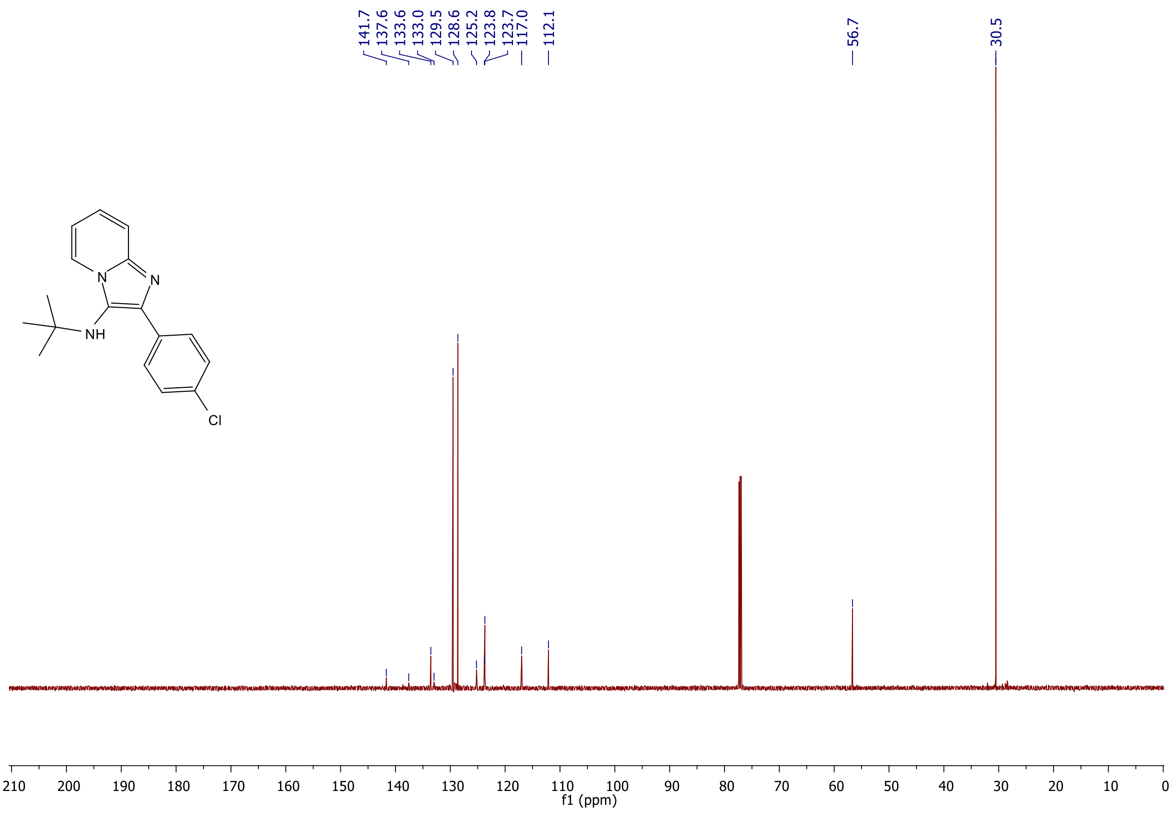

Figure S12. IR (ATR) of compound **1d**.

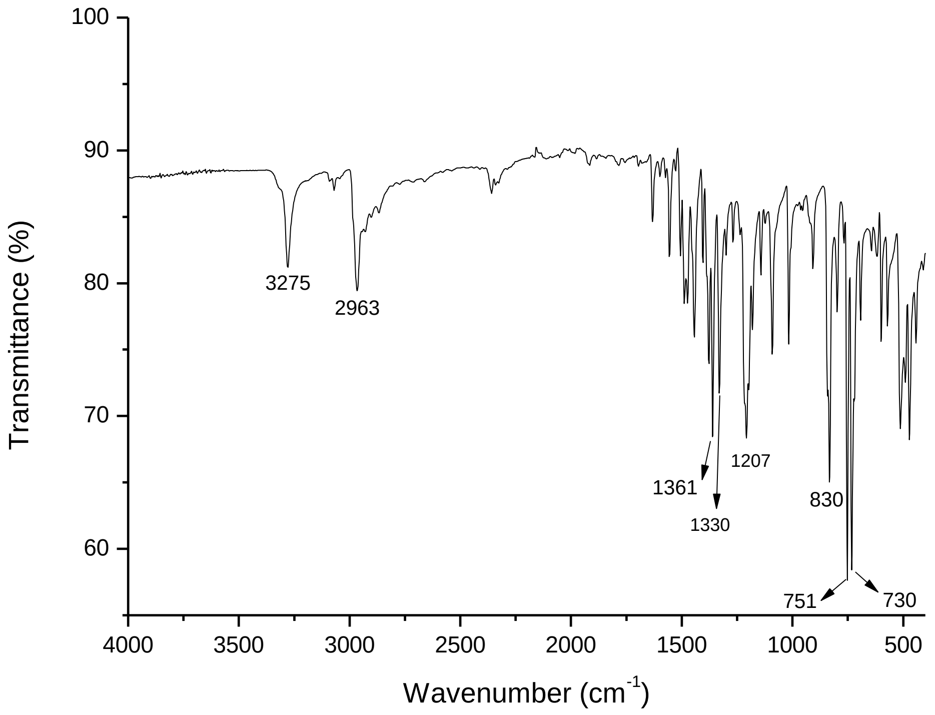

Figure S13. ^{1}H NMR of compound **1e** (600 MHz, $CDCl_3$).

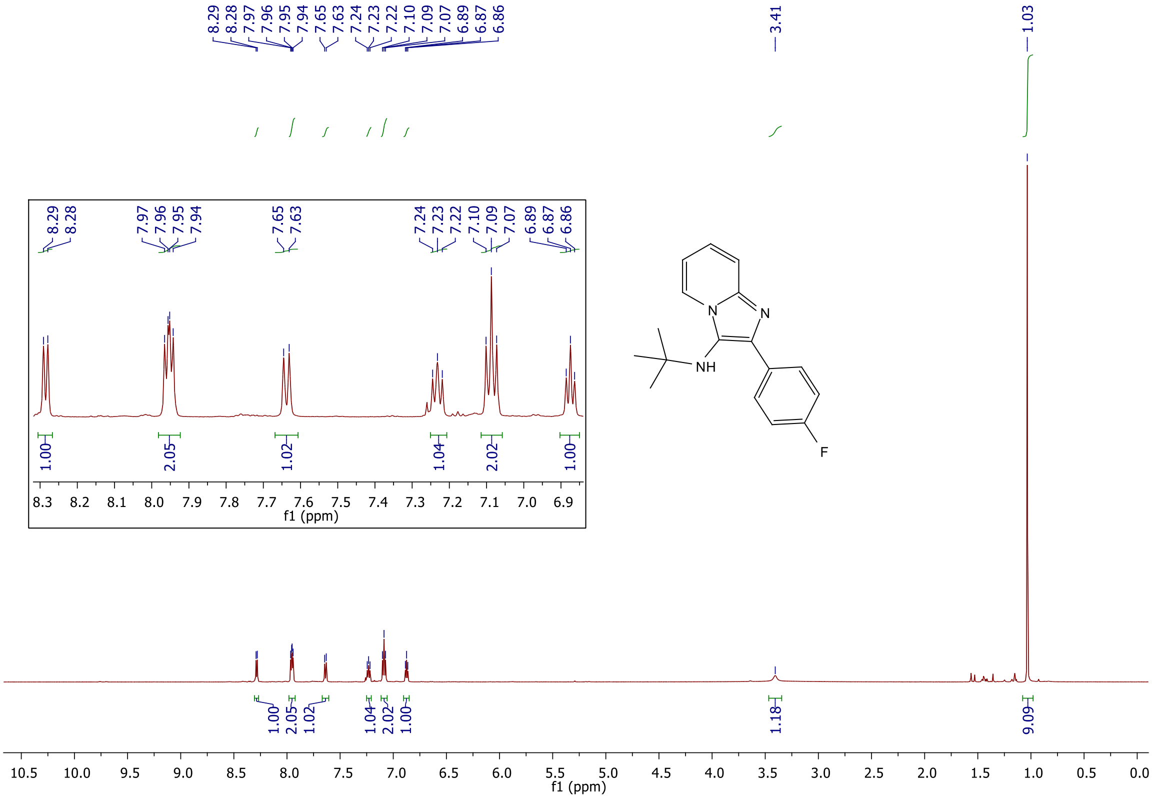

Figure S14. $^{13}C\{^{1}H\}$ NMR of compound **1e** (100 MHz, $CDCl_3$).

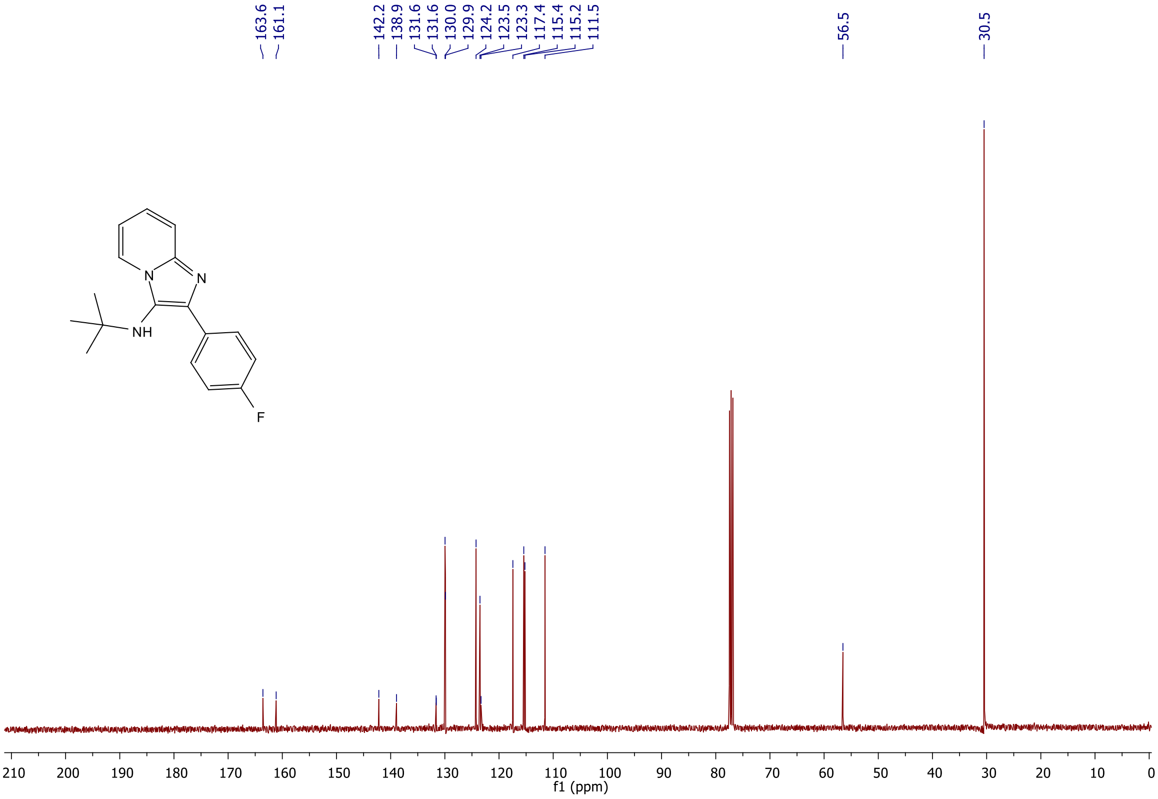

Figure S15. ^{19}F NMR of compound **1e** (376 MHz, $CDCl_3$).

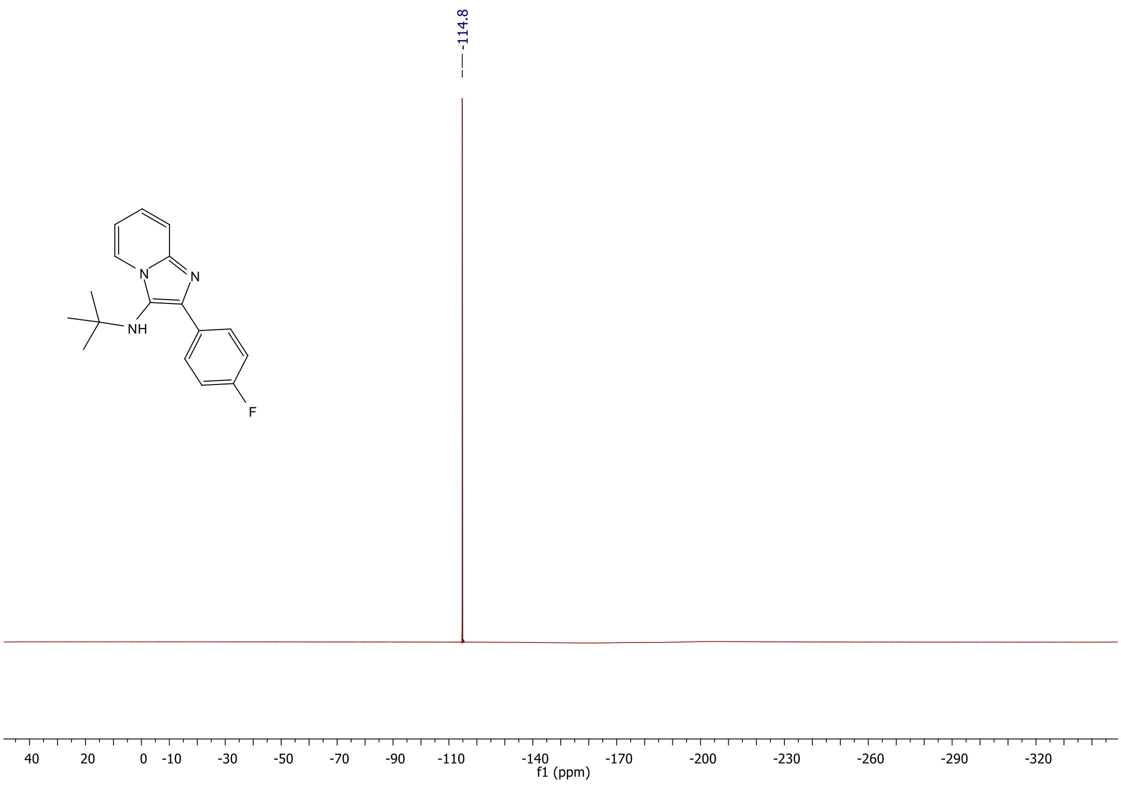

Figure S16. IR (ATR) of compound **1e**.

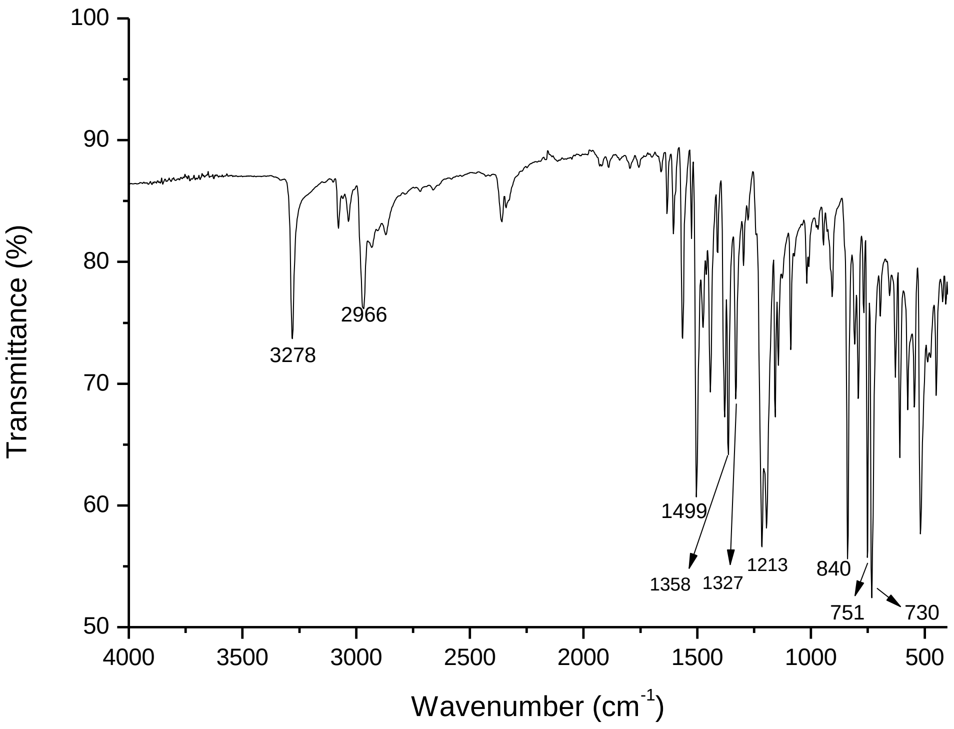

Figure S17. ^{1}H NMR of compound **1f** (600 MHz, $CDCl_3$).

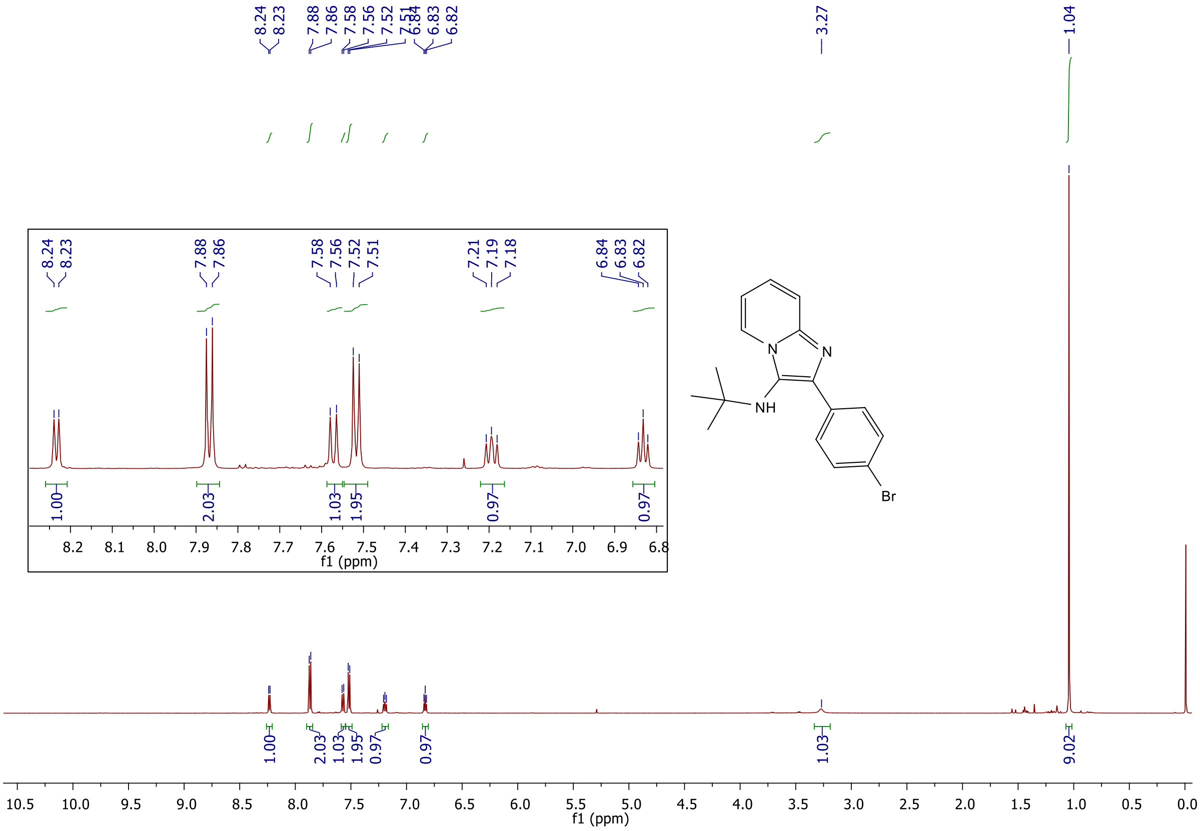

Figure S18. $^{13}C\{^{1}H\}$ NMR of compound **1f** (150 MHz, $CDCl_3$).

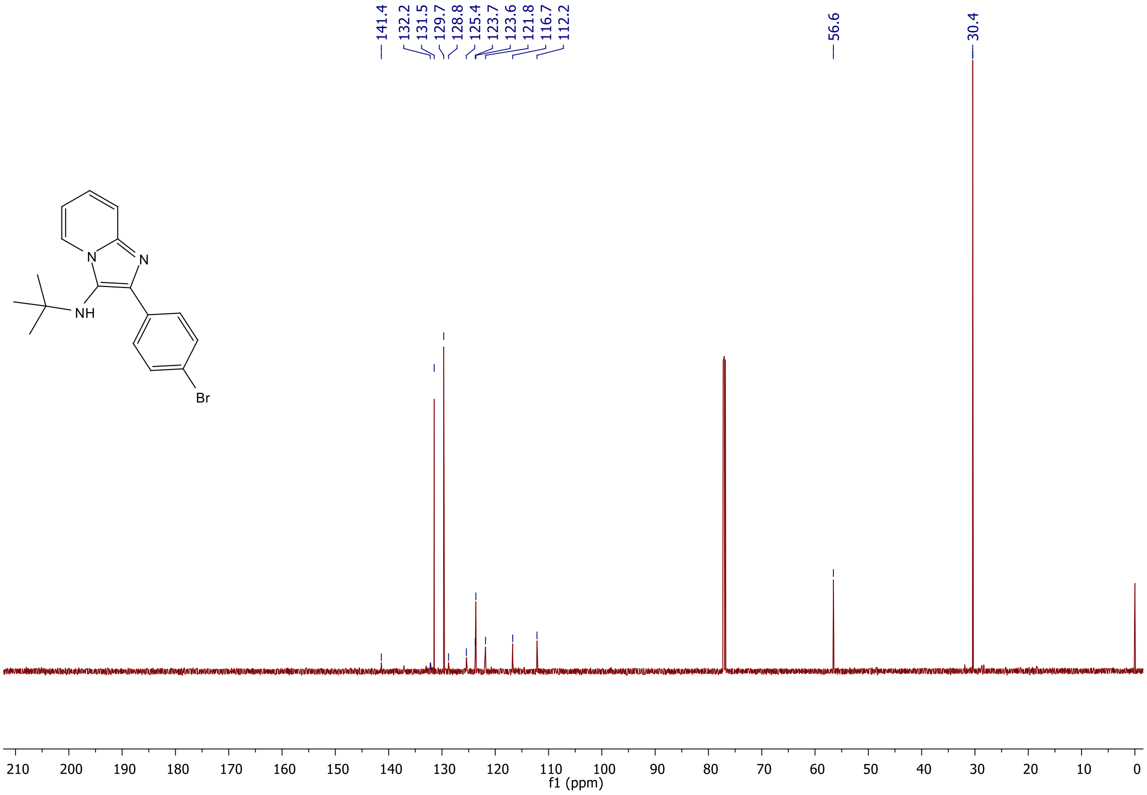

Figure S19. IR (ATR) of compound **1f.**

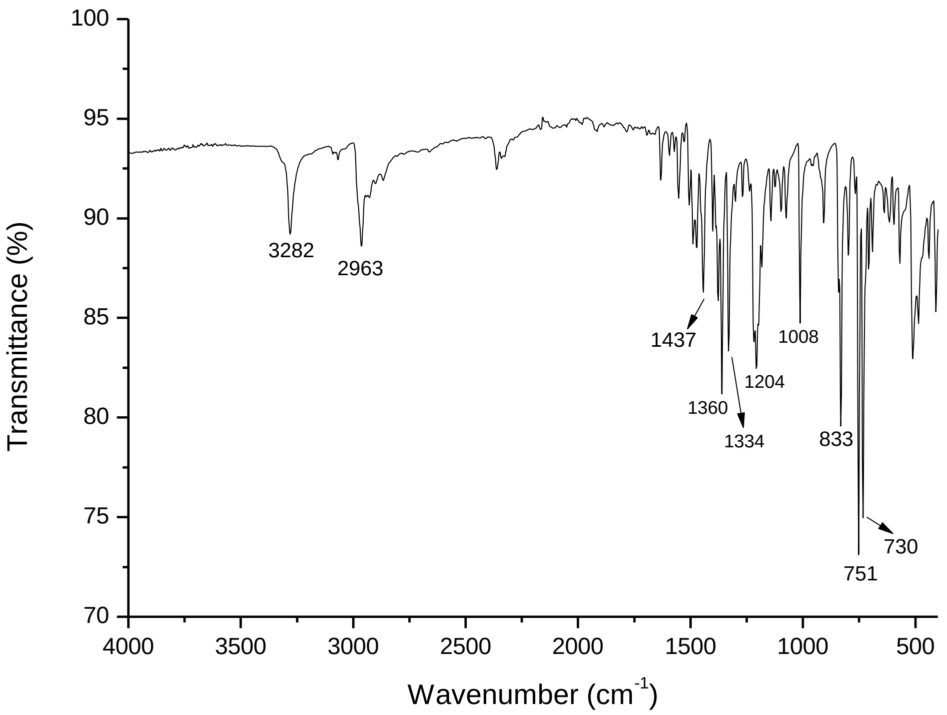

Figure S20. ^{1}H NMR of compound **1g** (600 MHz, $CDCl_3$).

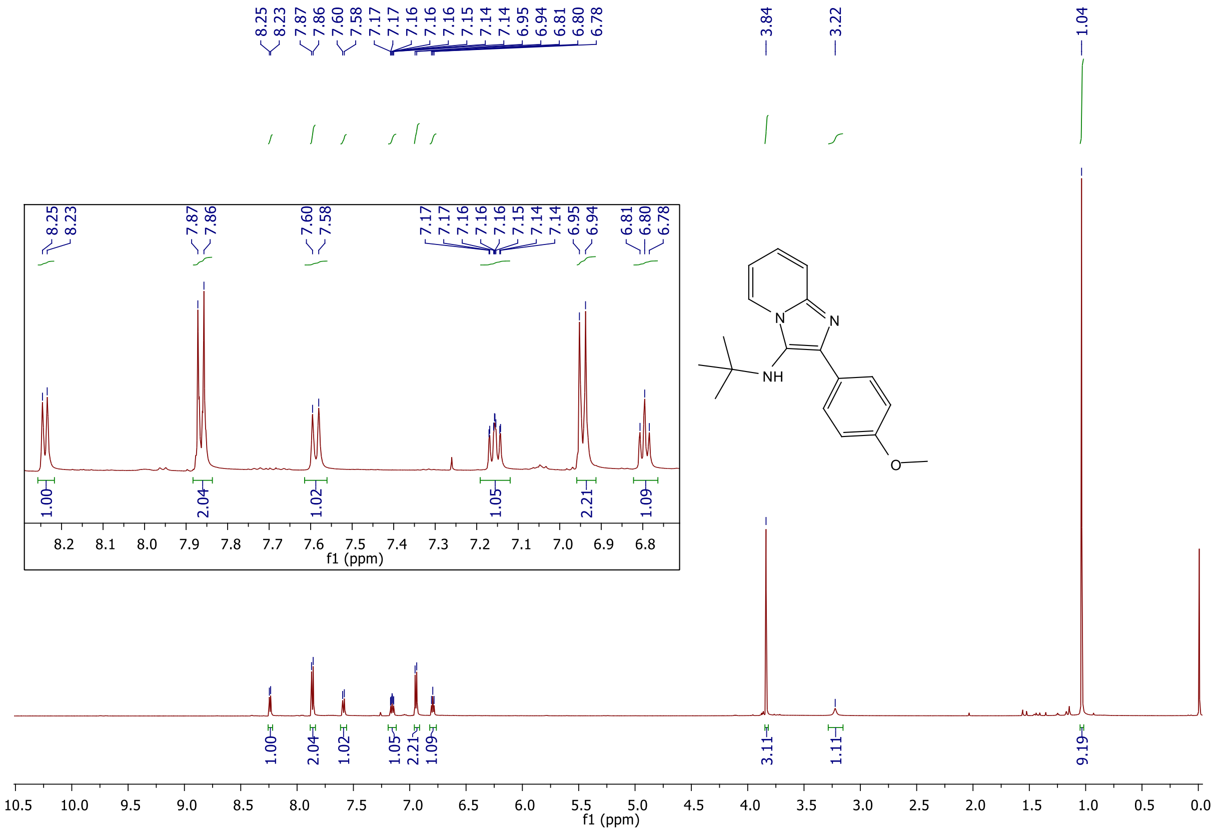

Figure S21. $^{13}C\{^1H\}$ NMR of compound **1g** (150 MHz, $CDCl_3$).

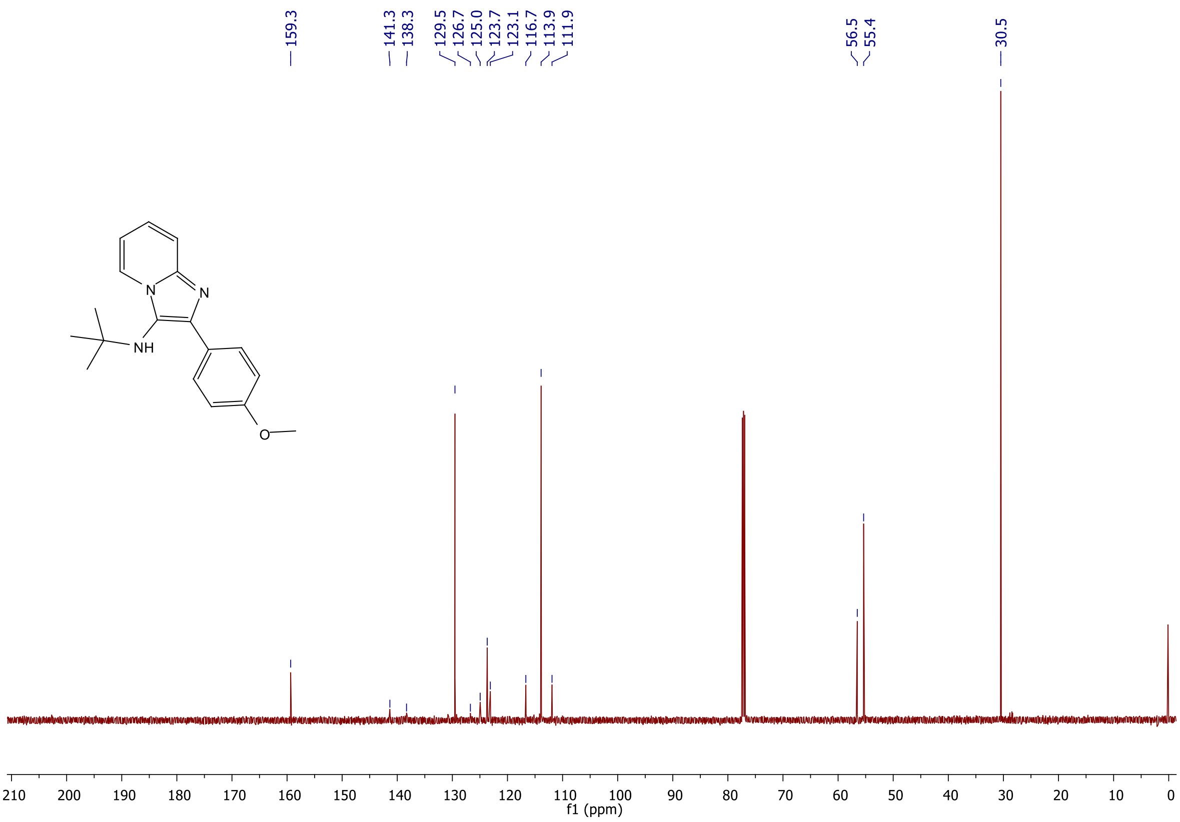

Figure S22. IR (ATR) of compound **1g.**

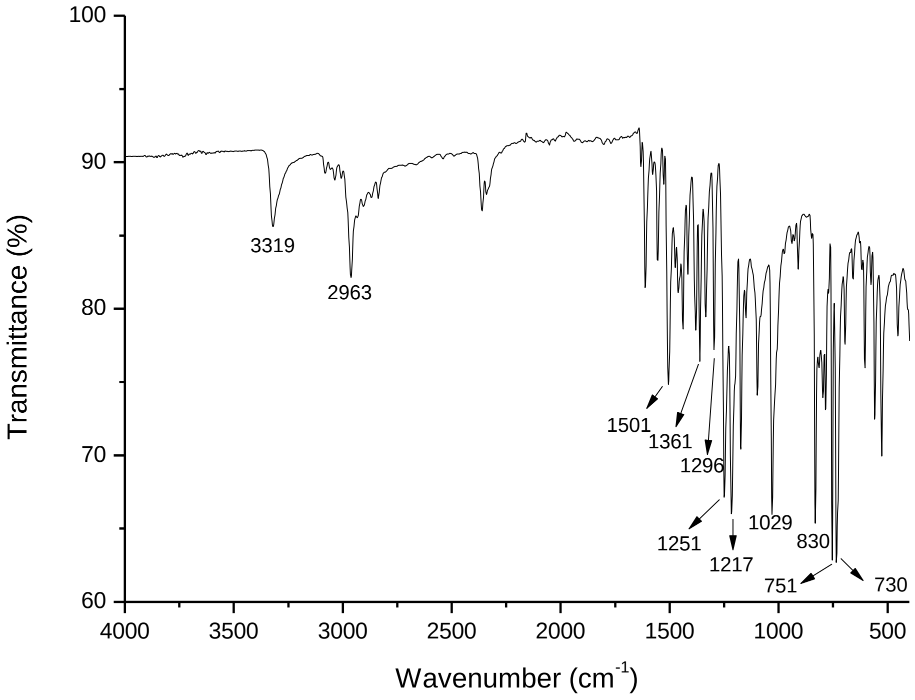

Figure S23. ^{1}H NMR of compound **1h** (600 MHz, $CDCl_3$).

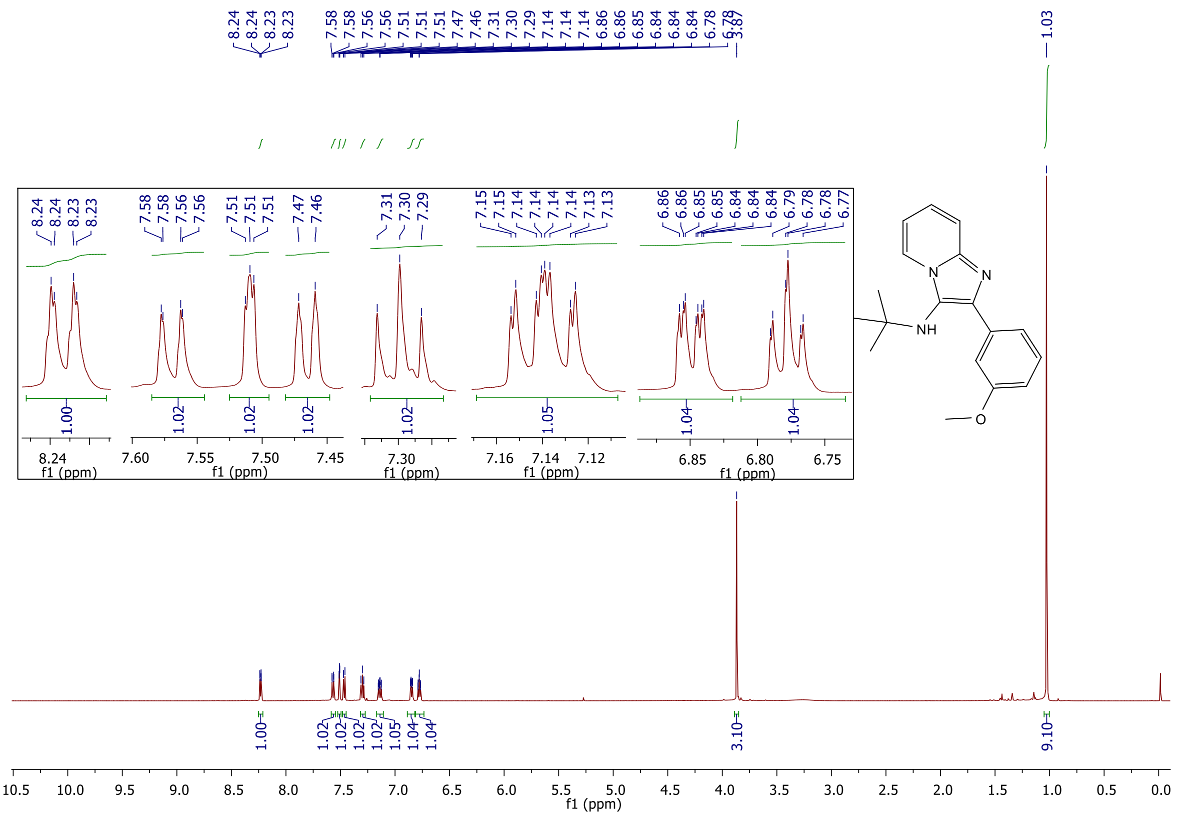

Figure S24. $^{13}C\{^{1}H\}$ NMR of compound **1h** (150 MHz, $CDCl_3$).

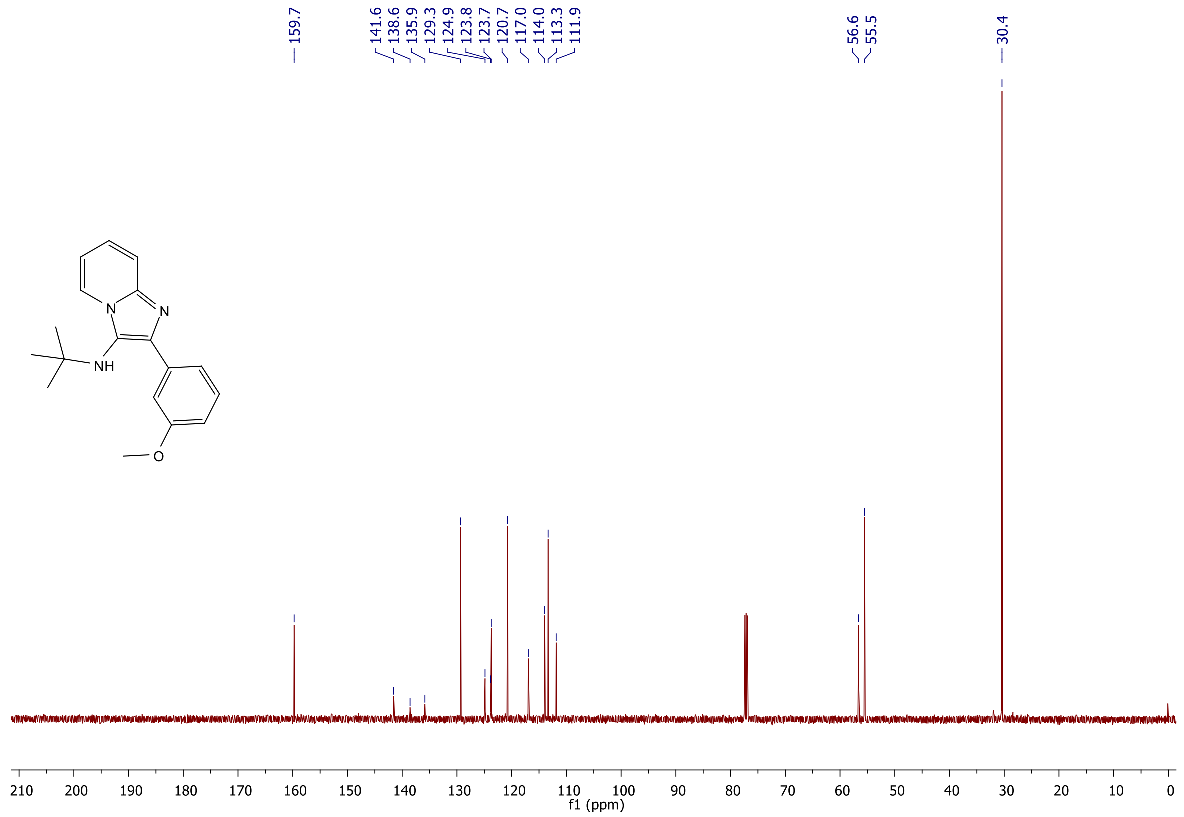

Figure S25. IR (ATR) of compound **1h.**

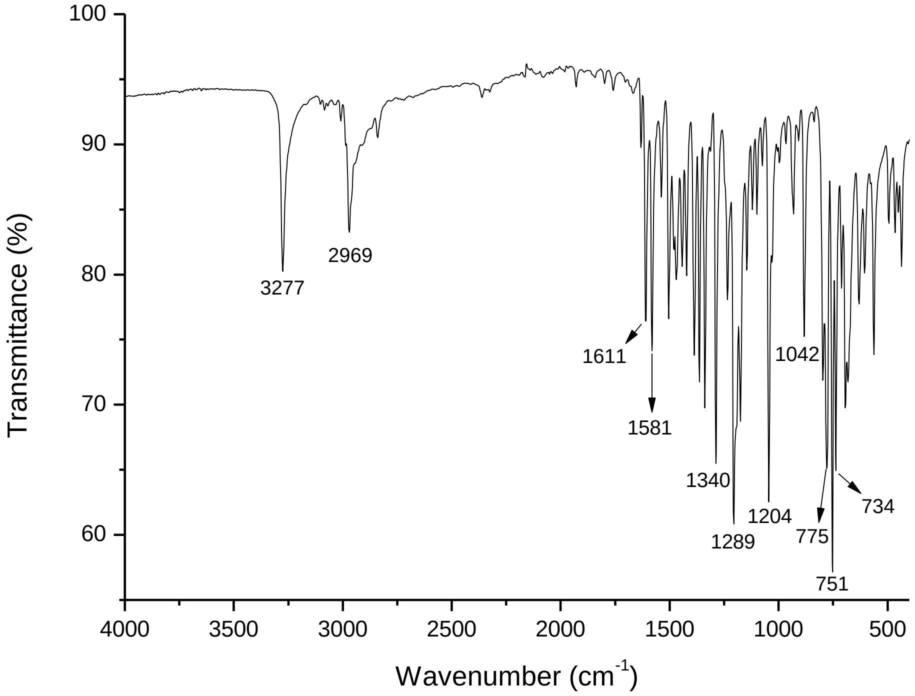

Figure S26. ^{1}H NMR of compound **1i** (600 MHz, $CDCl_3$).

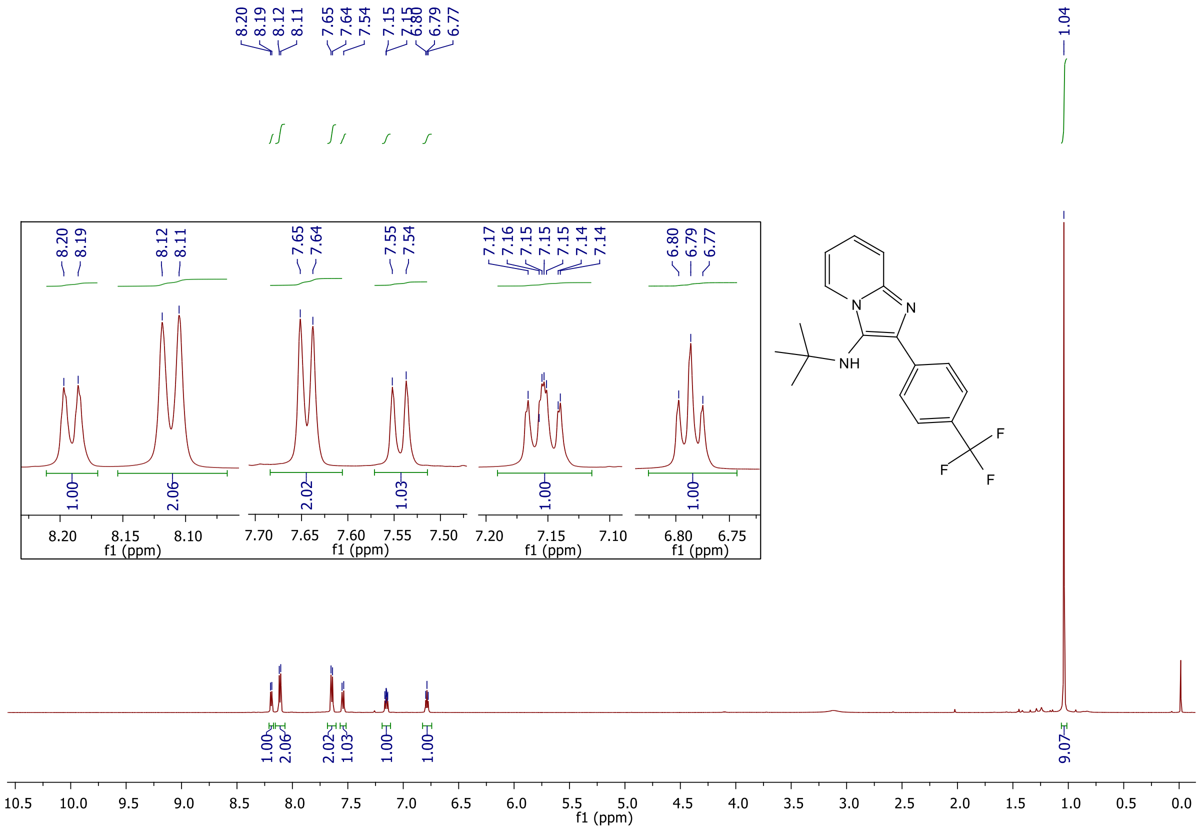

Figure S27. $^{13}C\{^{1}H\}$ NMR of compound **1i** (150 MHz, $CDCl_3$).

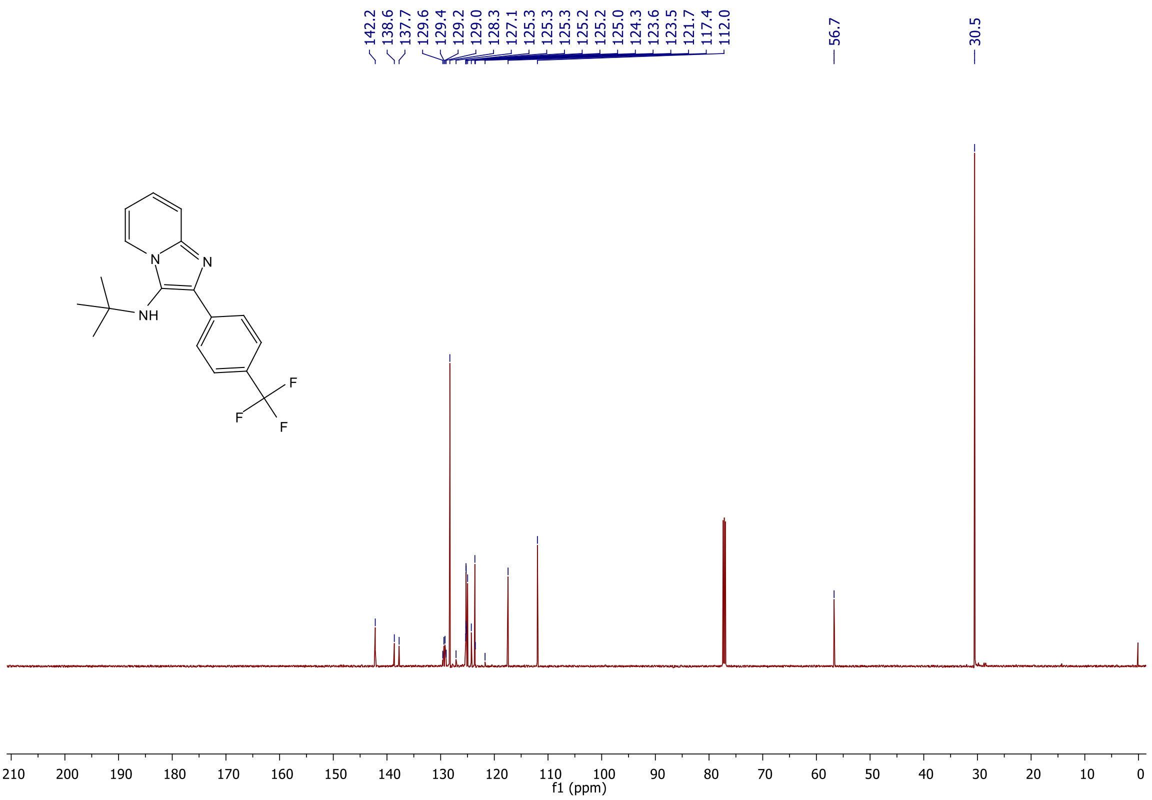

Figure S28. $^{19}F\{^{1}H\}$ NMR of compound **1i** (376 MHz, $CDCl_3$).

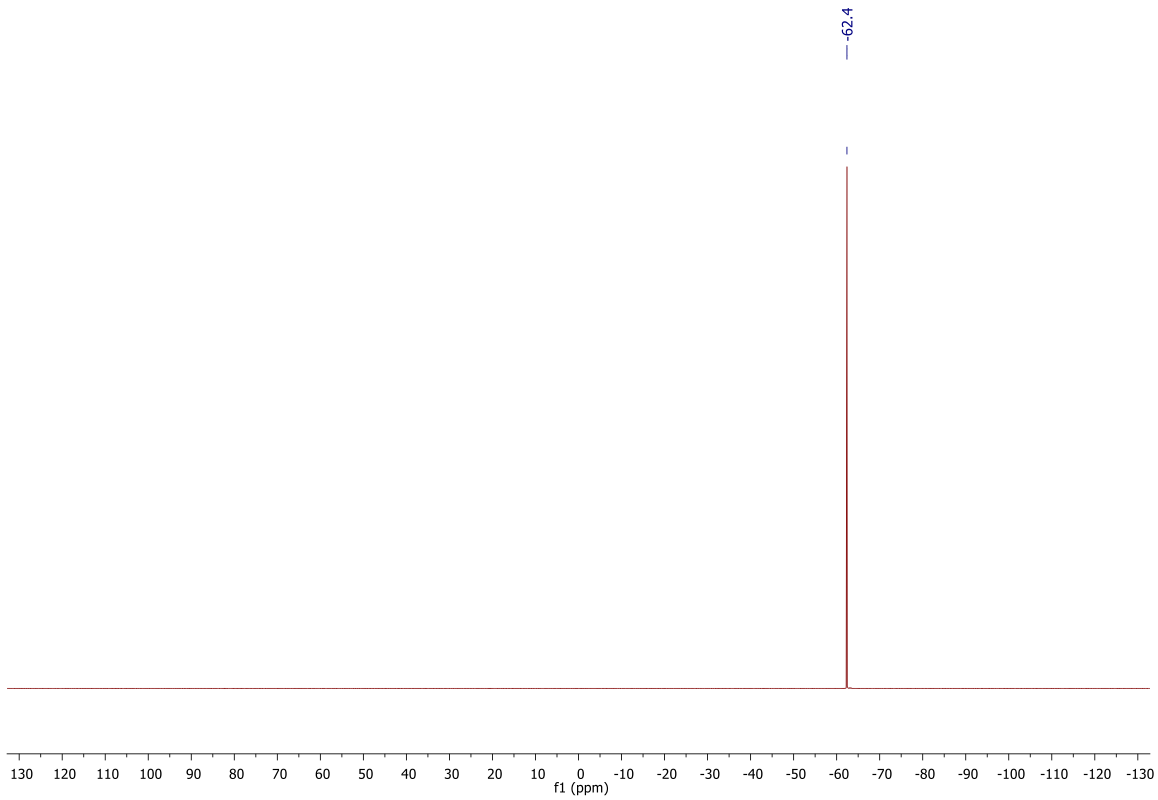

Figure S29. IR (ATR) of compound **1i.**

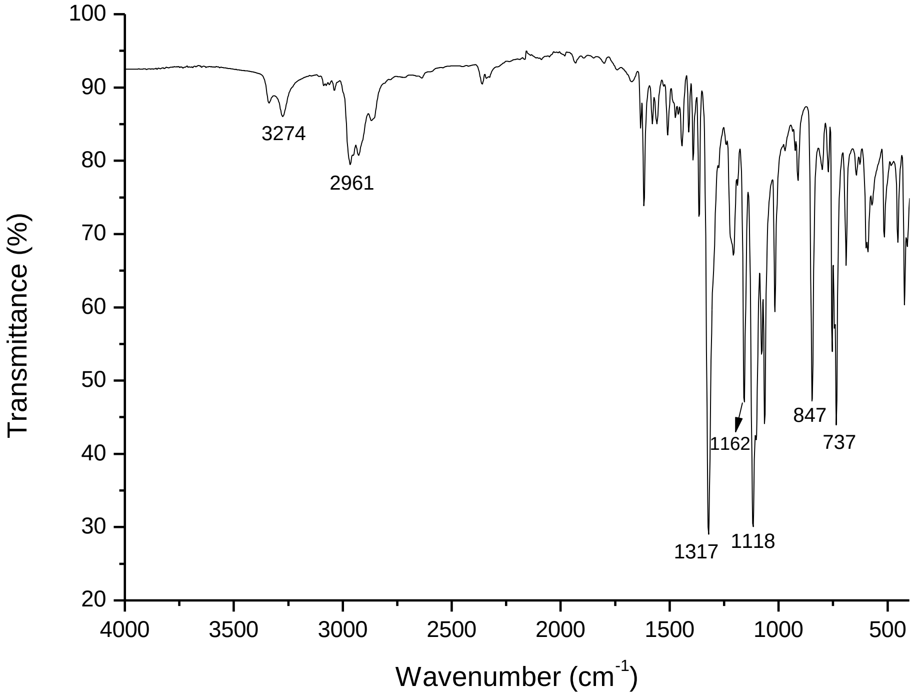

Figure S30. HRMS (ESI-TOF) $[M + H]^+$ of compound **1i**.

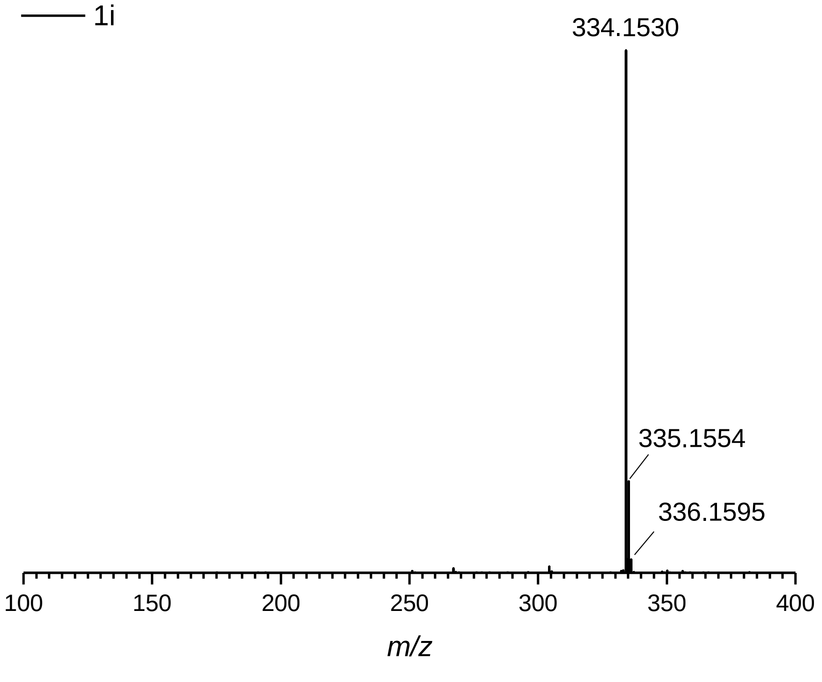

Figure S31. ^{1}H NMR of compound **1j** (600 MHz, $CDCl_3$).

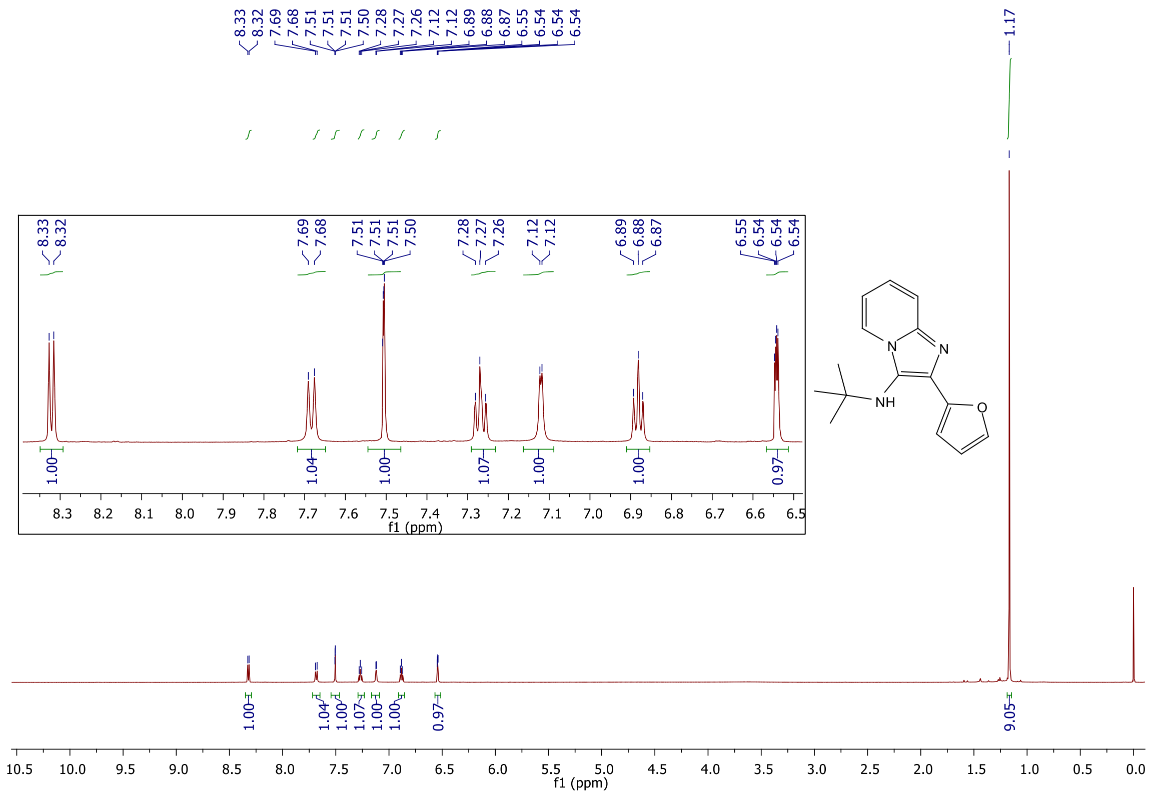

Figure S32. $^{13}C\{^{1}H\}$ NMR of compound **1j** (150 MHz, $CDCl_3$).

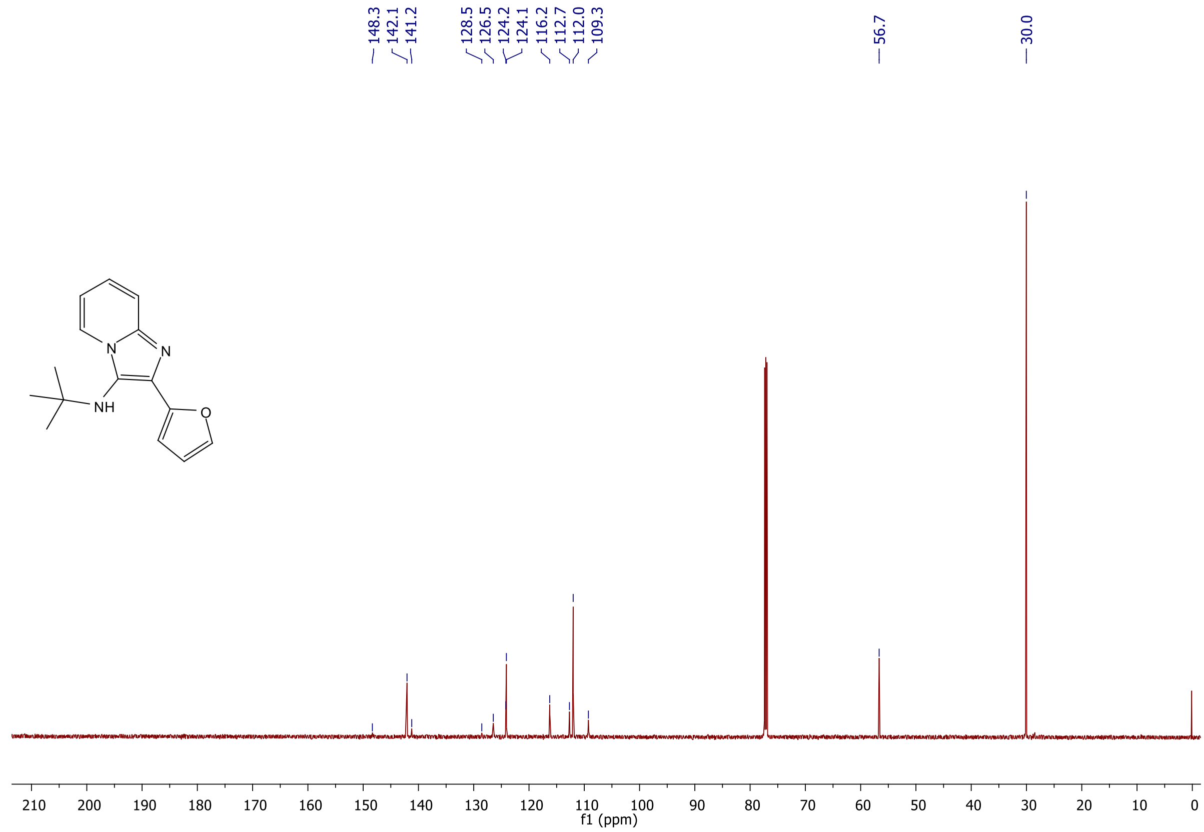

Figure S33. IR (ATR) of compound **1j**.

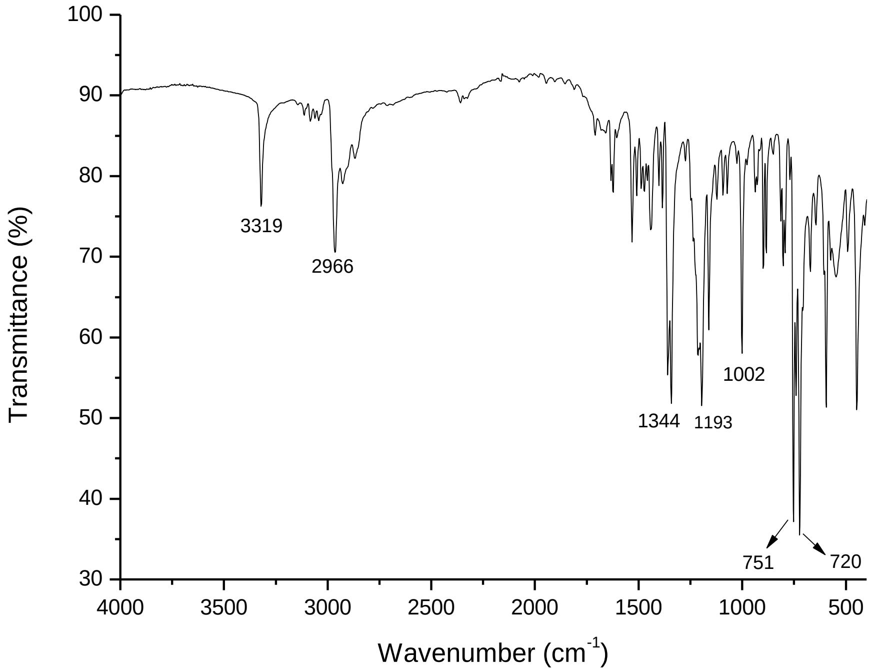

Figure S34. ^{1}H NMR of compound **1k** (600 MHz, $CDCl_3$).

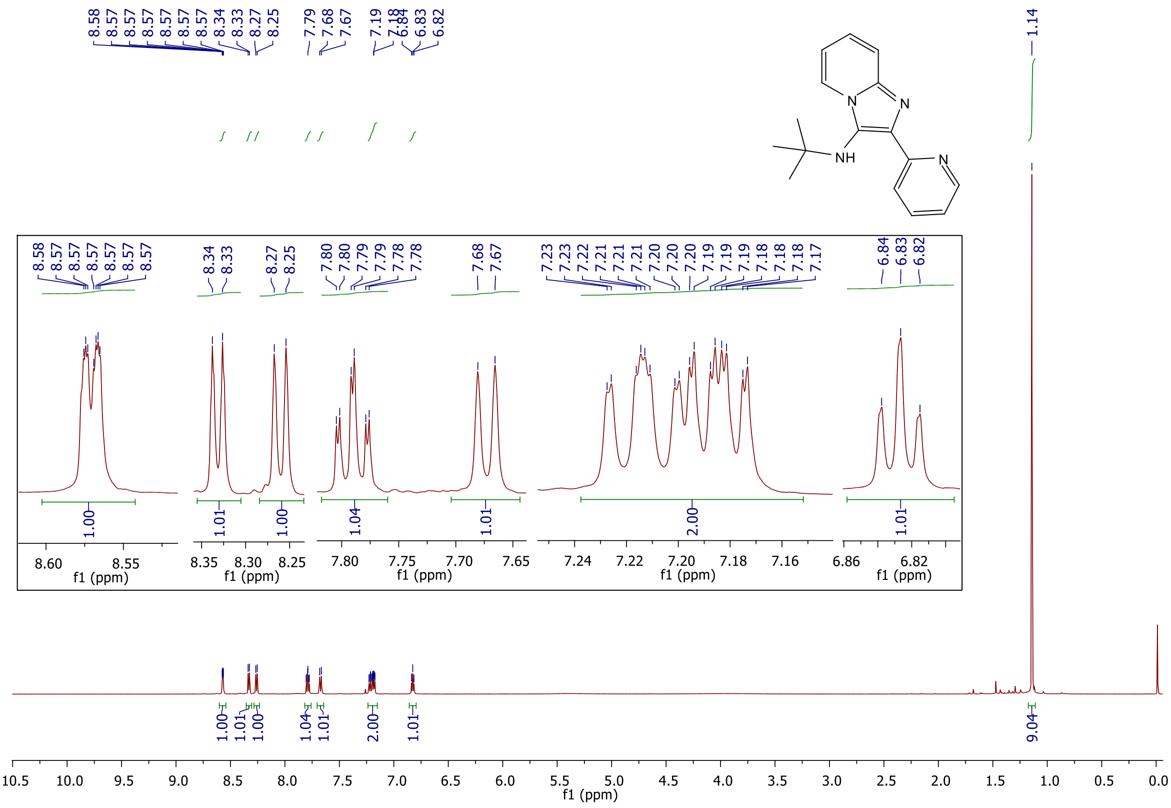

Figure S35. $^{13}C\{^{1}H\}$ NMR of compound **1k** (150 MHz, $CDCl_3$).

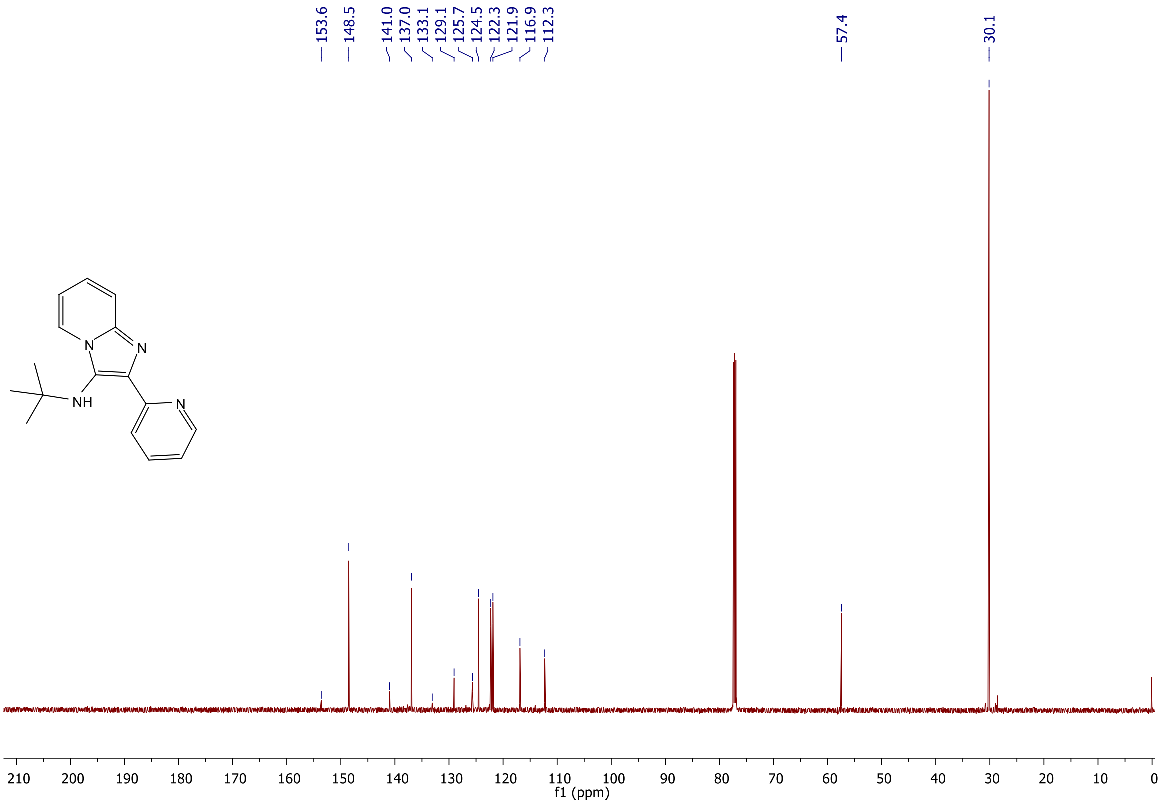

Figure S36. IR (ATR) of compound **1k**.

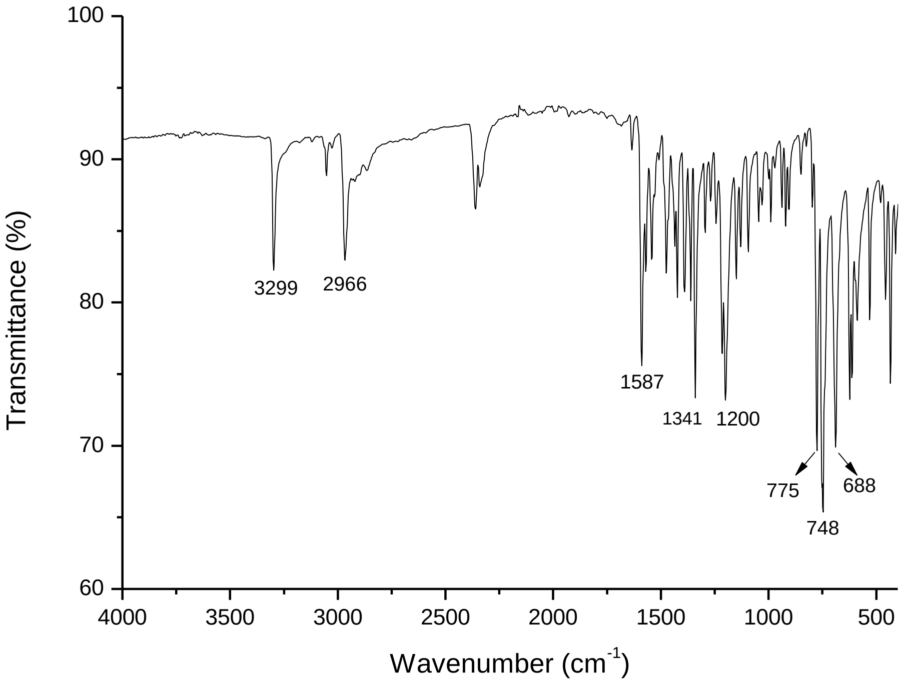

Figure S37. ^{1}H NMR of compound **1l** (600 MHz, $CDCl_3$).

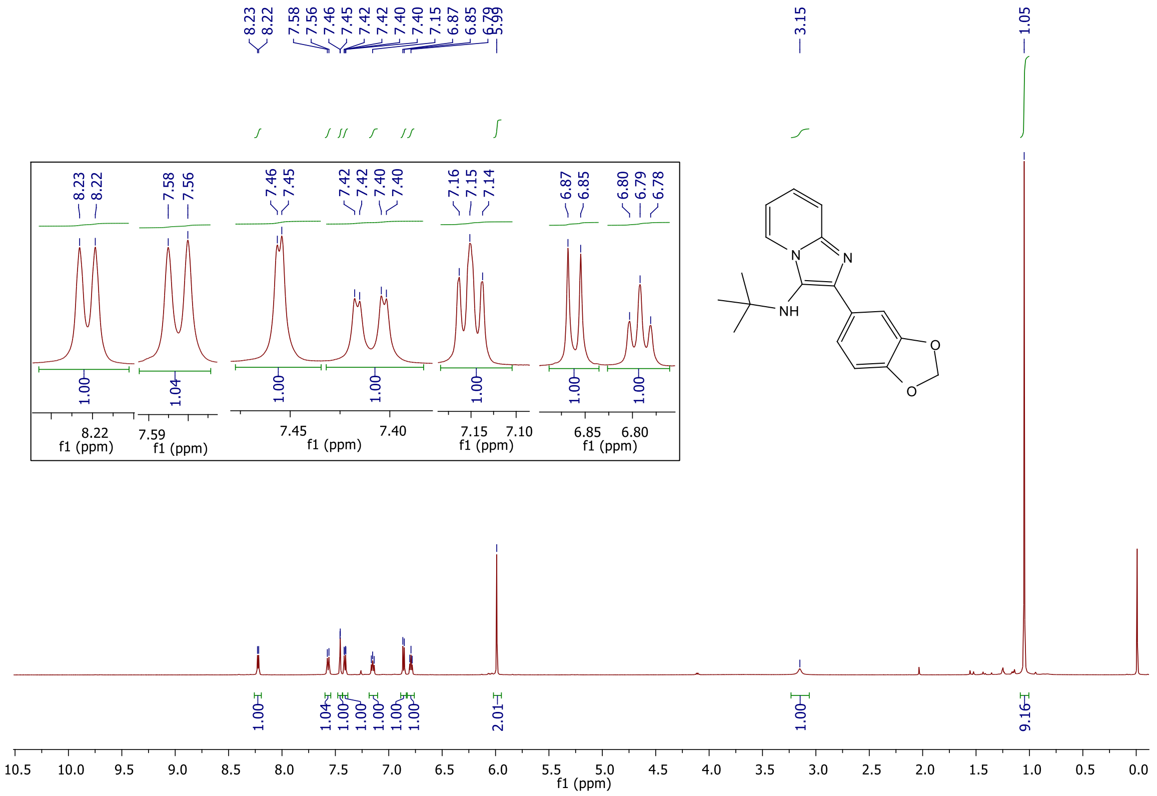

Figure S38. $^{13}C\{^{1}H\}$ NMR of compound **1l** (150 MHz, $CDCl_3$).

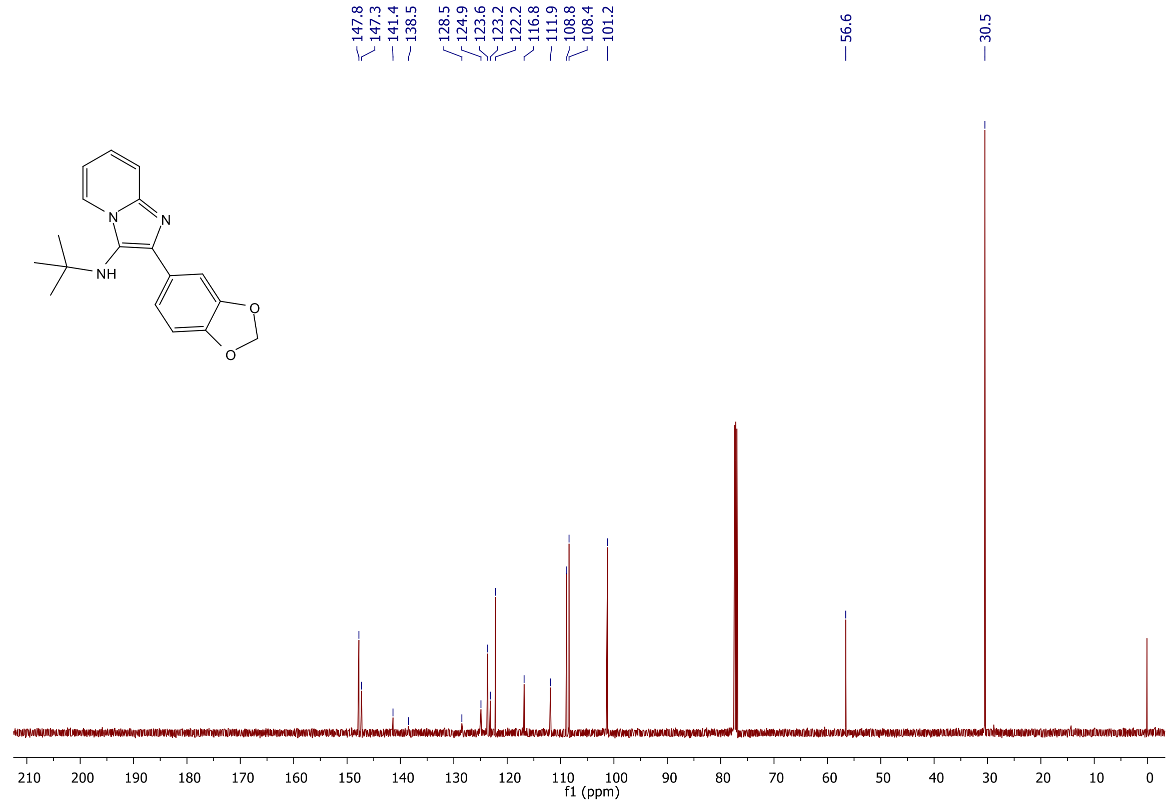

Figure S39. IR (ATR) of compound **1l**.

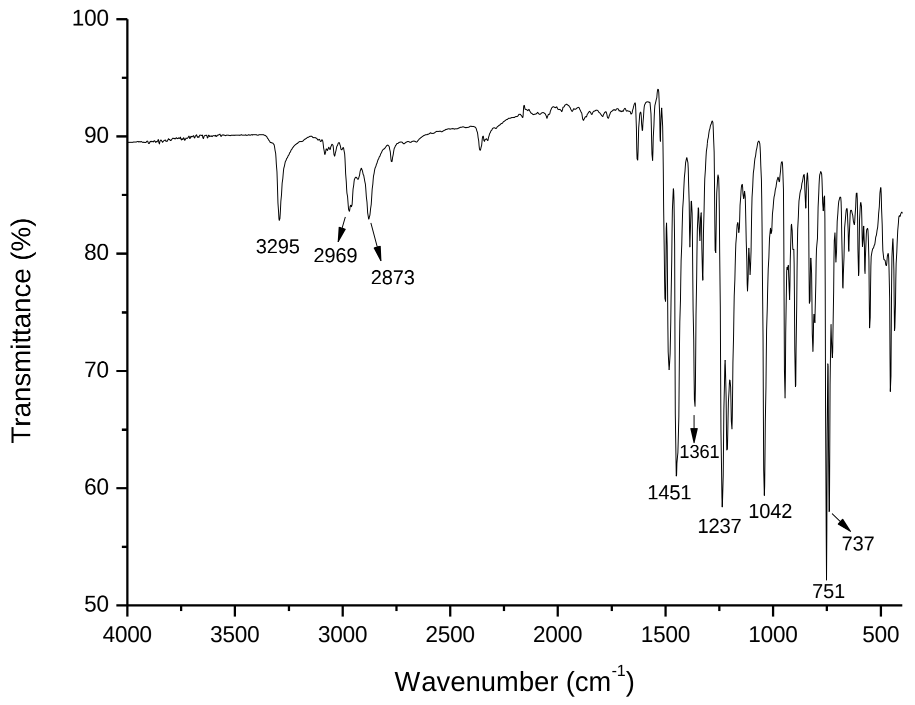

Figure S40. HRMS (ESI-TOF) $[M + H]^+$ of compound **1l**.

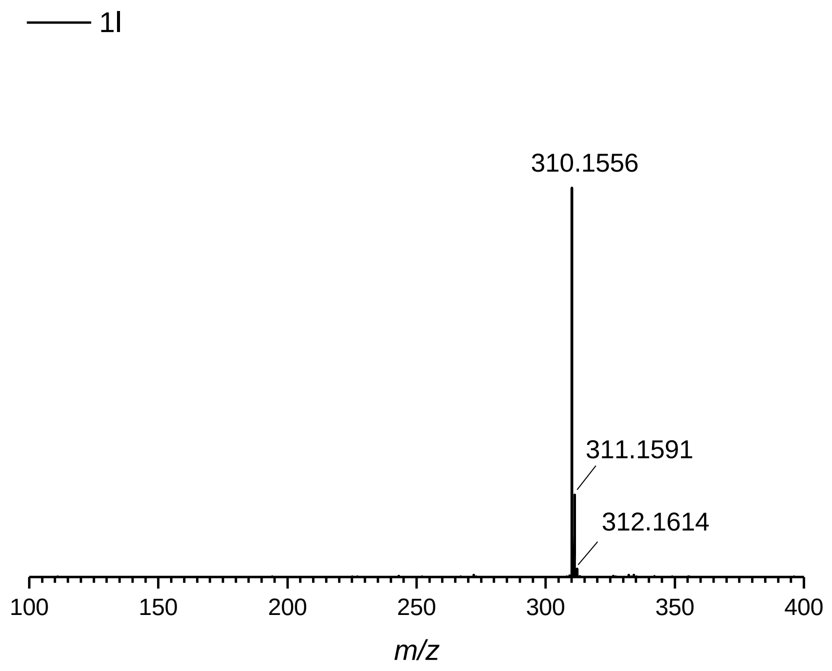

Figure S41. ^{1}H NMR of compound **1m** (600 MHz, $CDCl_3$).

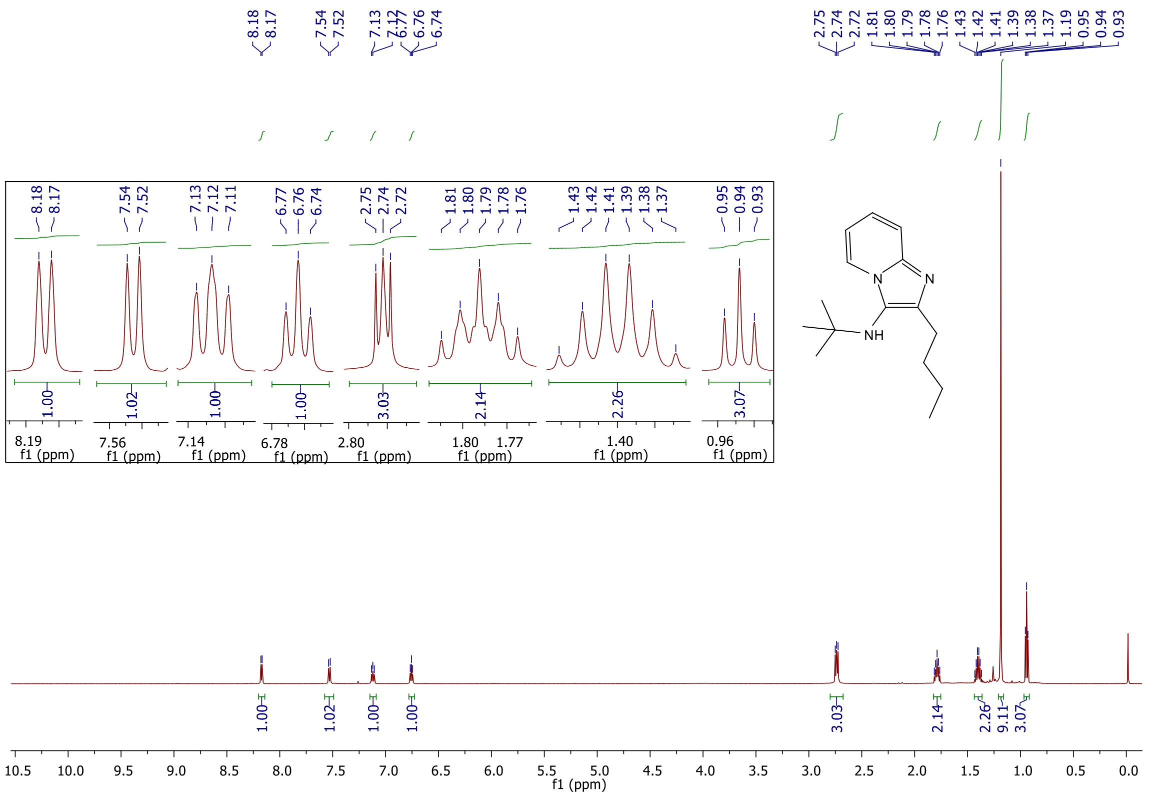

Figure S42. $^{13}C\{^{1}H\}$ NMR of compound **1m** (150 MHz, $CDCl_3$).

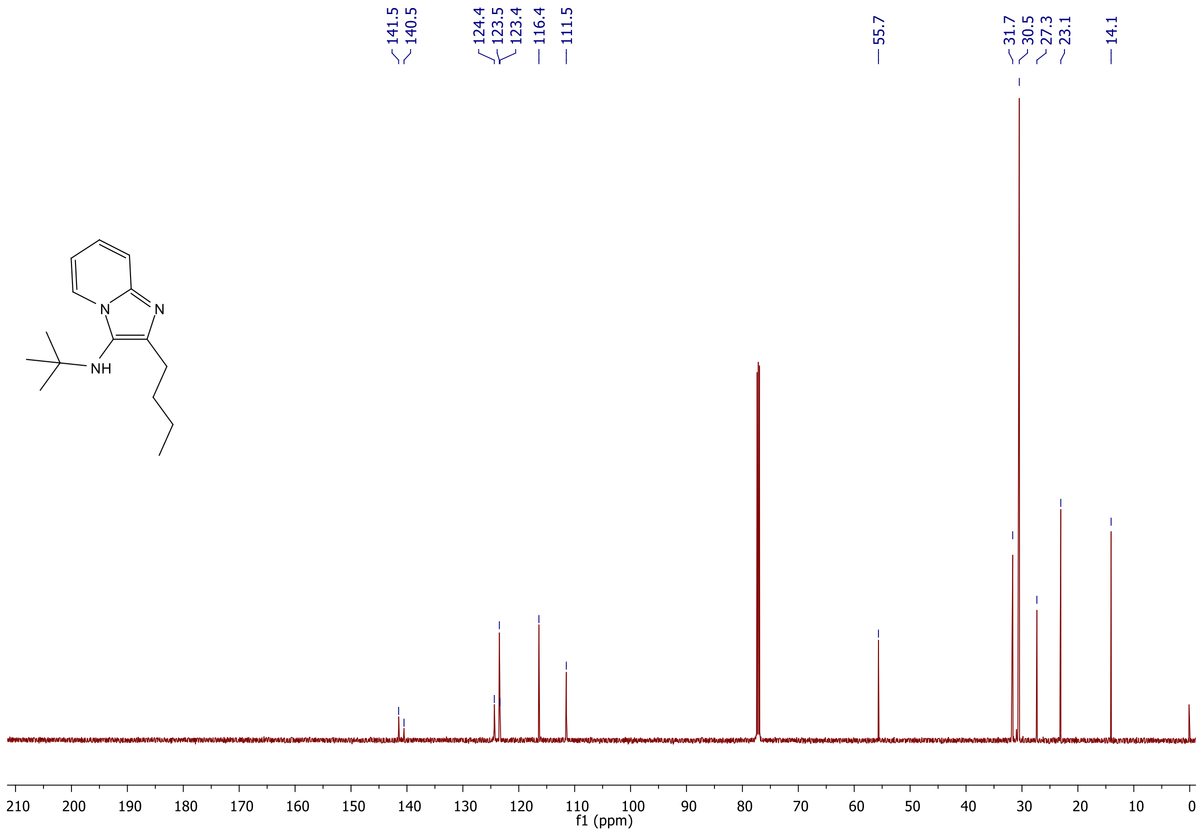

Figure S43. IR (ATR) of compound **1m**.

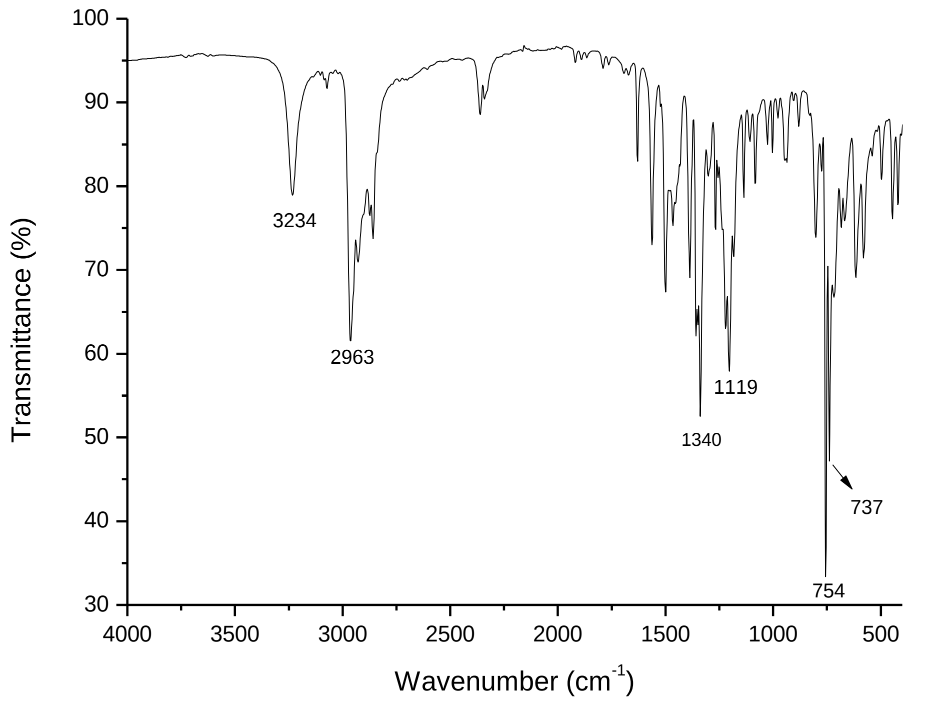

Figure S44. HRMS (ESI-TOF) $[M + H]^+$ of compound **1m**.

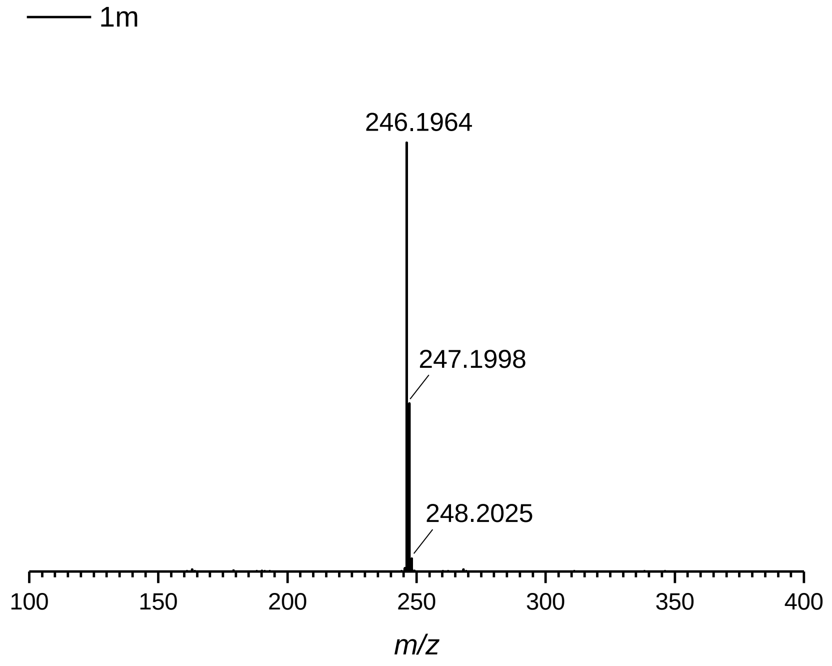

Figure S45. ^{1}H NMR of compound **1n** (600 MHz, $CDCl_3$).

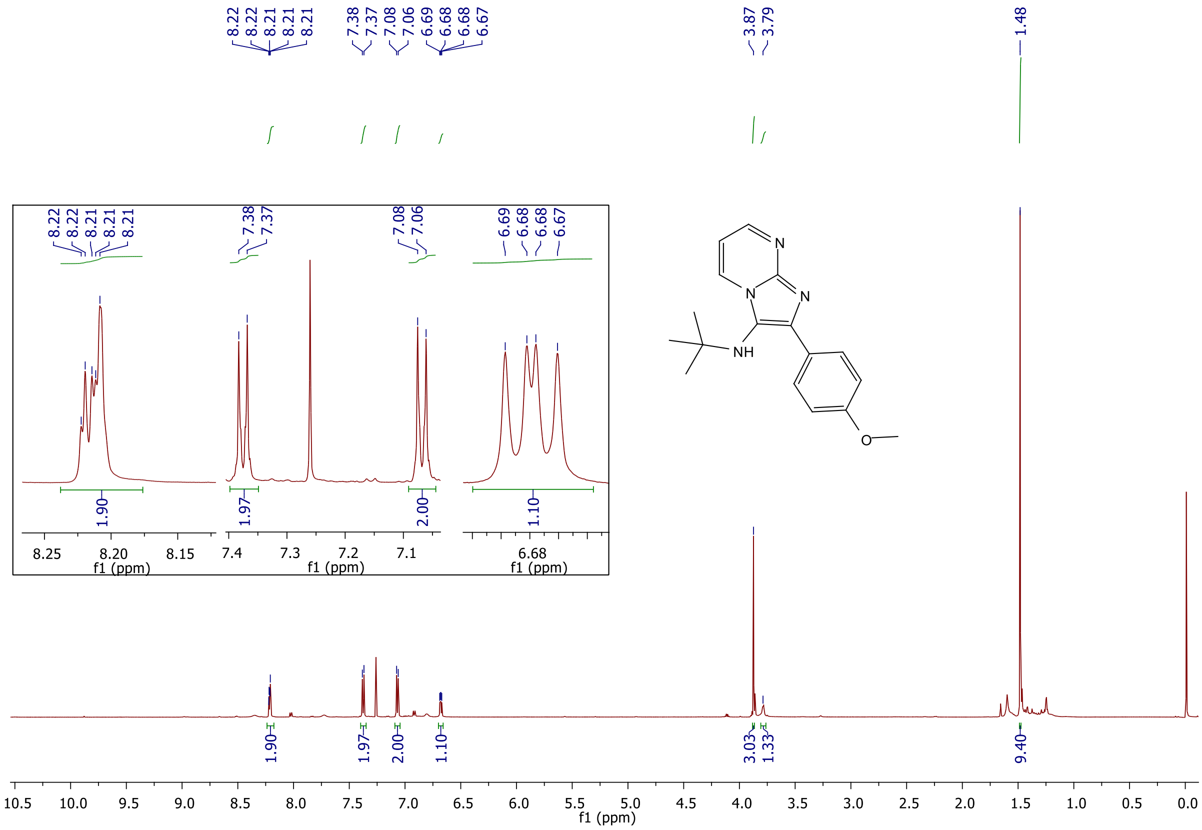

Figure S46. $^{13}C\{^{1}H\}$ NMR of compound **1n** (150 MHz, $CDCl_3$).

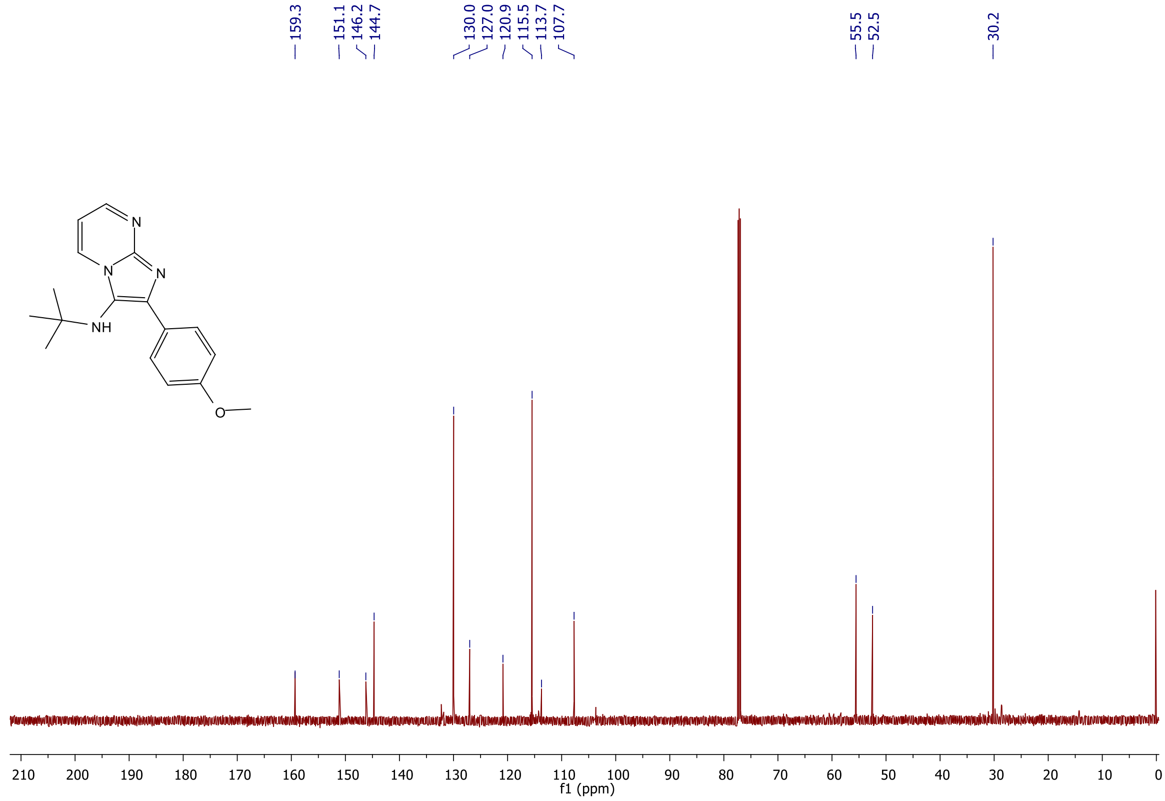

Figure S47. IR (ATR) of compound **1n**.

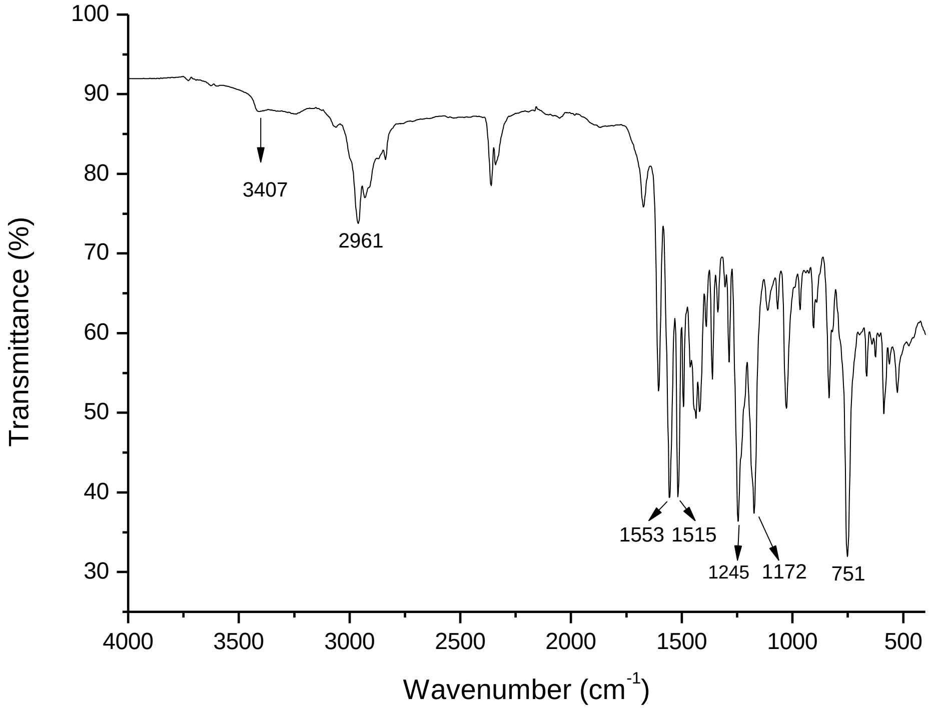

Figure S48. ^{1}H NMR of compound **1o** (400 MHz, $CDCl_3$).

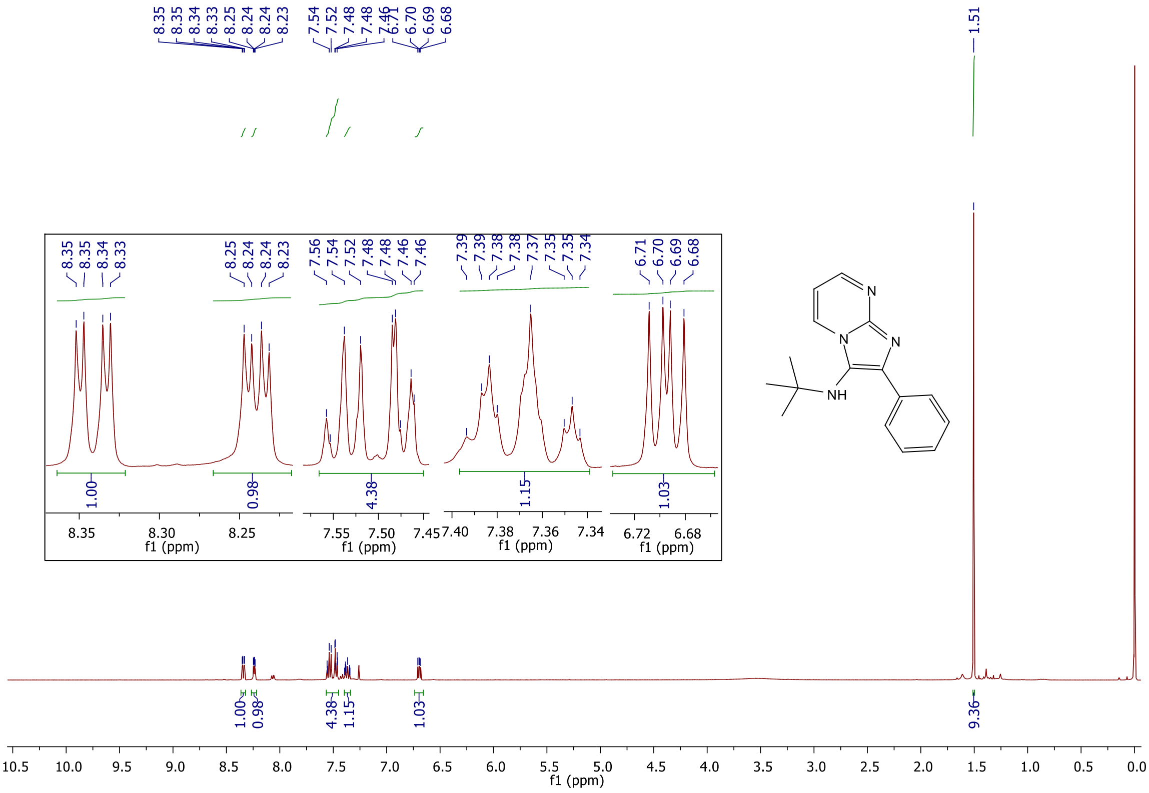

Figure S49. $^{13}C\{^{1}H\}$ NMR of compound **1o** (100 MHz, $CDCl_3$).

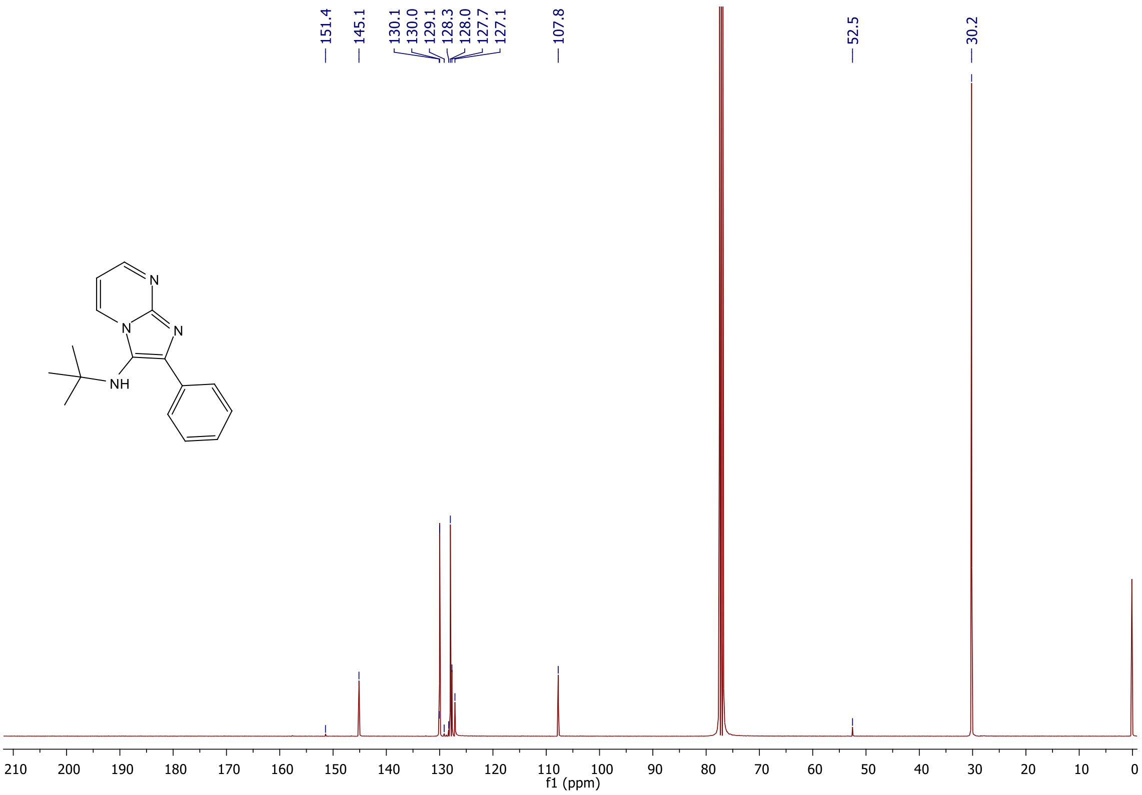

Figure S50. IR (ATR) of compound **1o**.

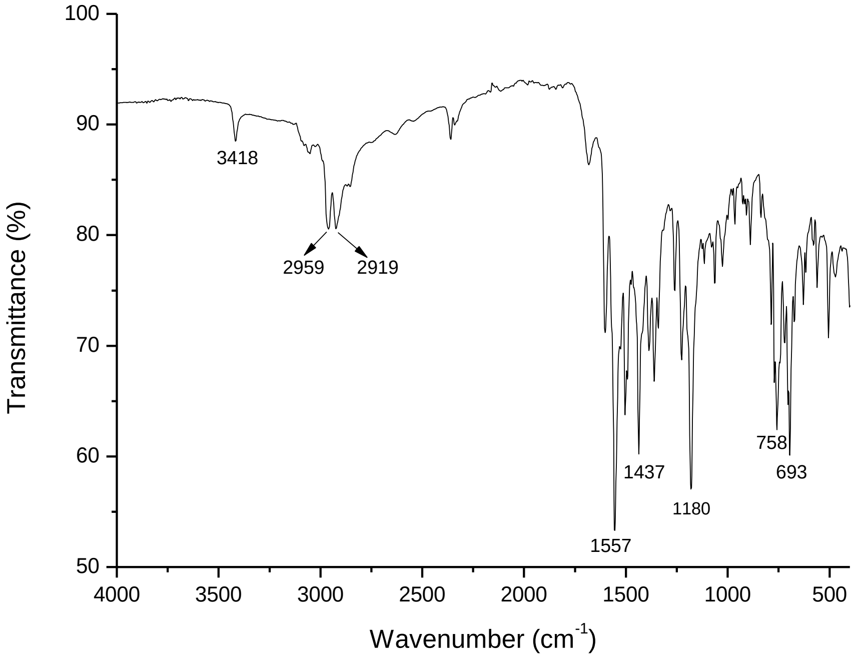

Figure S51. ^{1}H NMR of compound **1p** (600 MHz, $CDCl_3$).

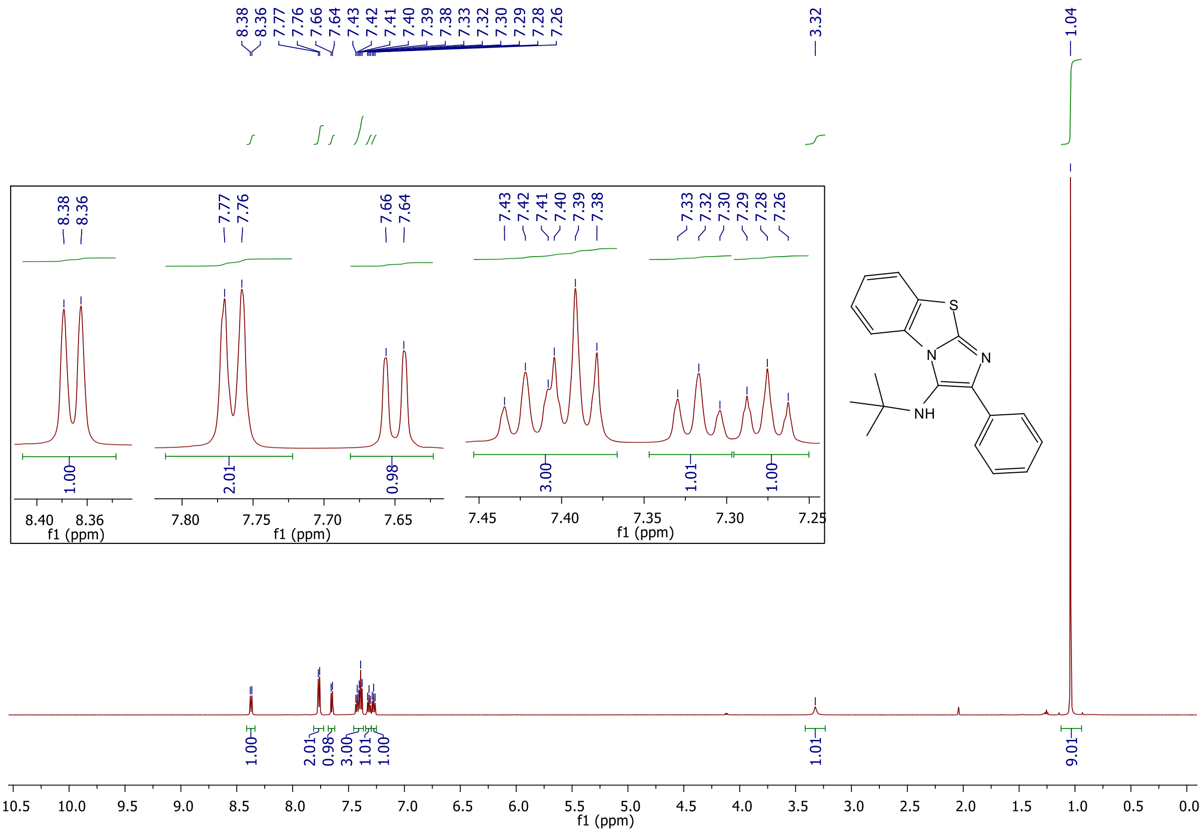

Figure S52. ^{13}C{^{1}H} NMR of compound **1p** (150 MHz, $CDCl_3$).

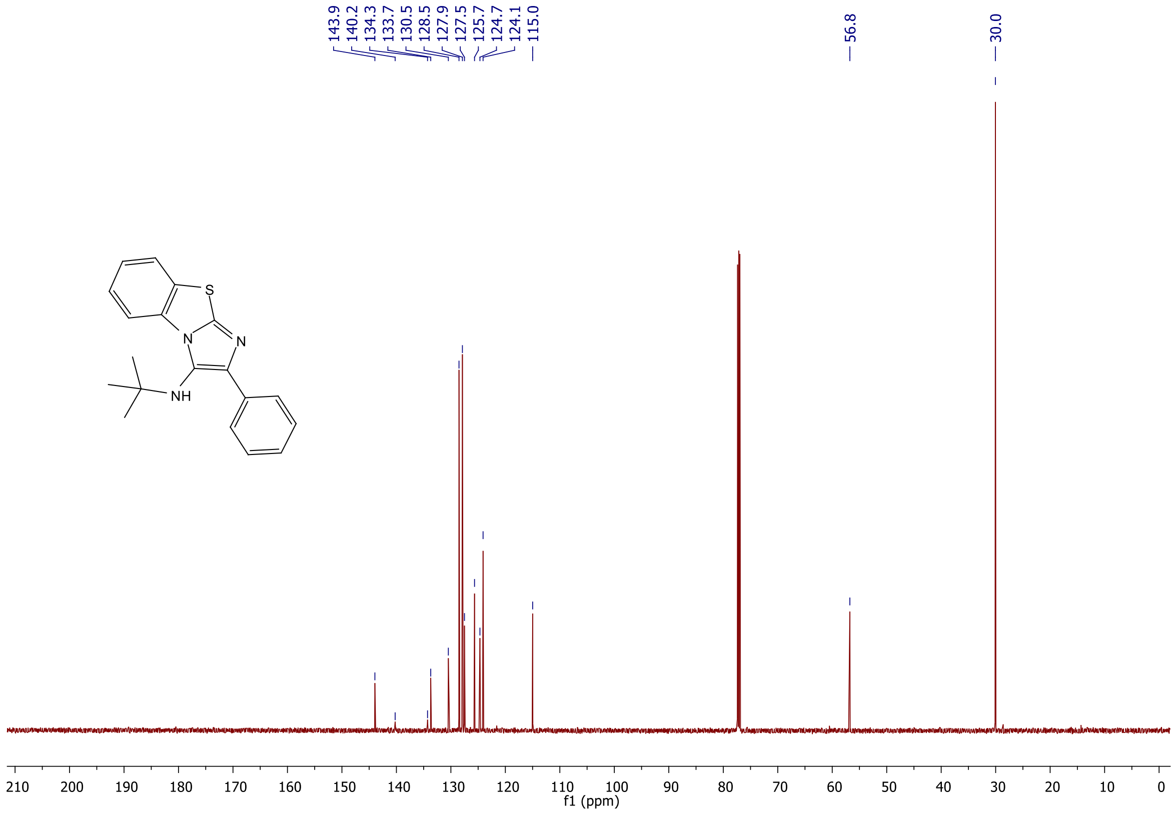

Figure S53. IR (ATR) of compound **1p**.

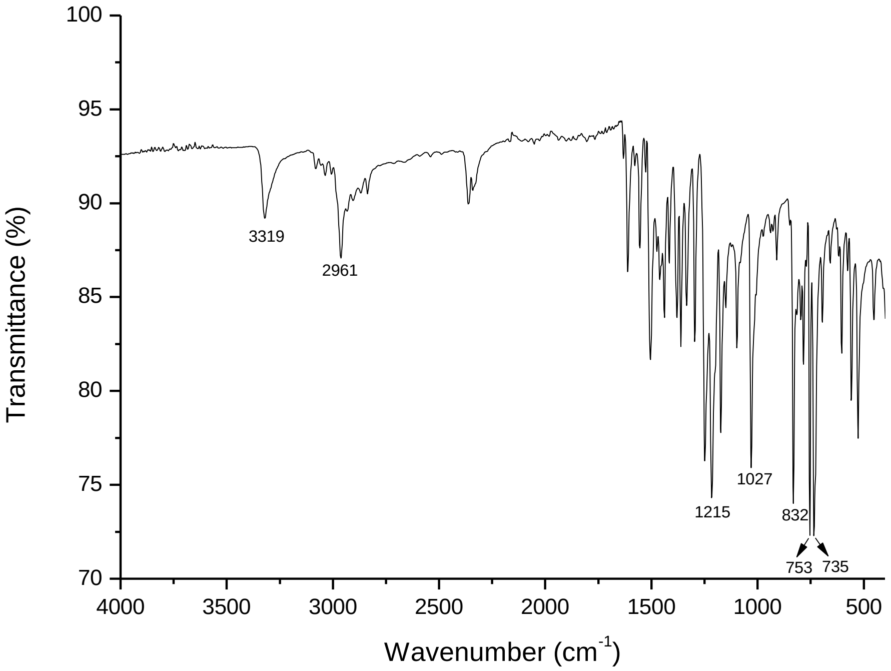

Figure S54. ^{1}H NMR of compound **1q** (600 MHz, $CDCl_3$).

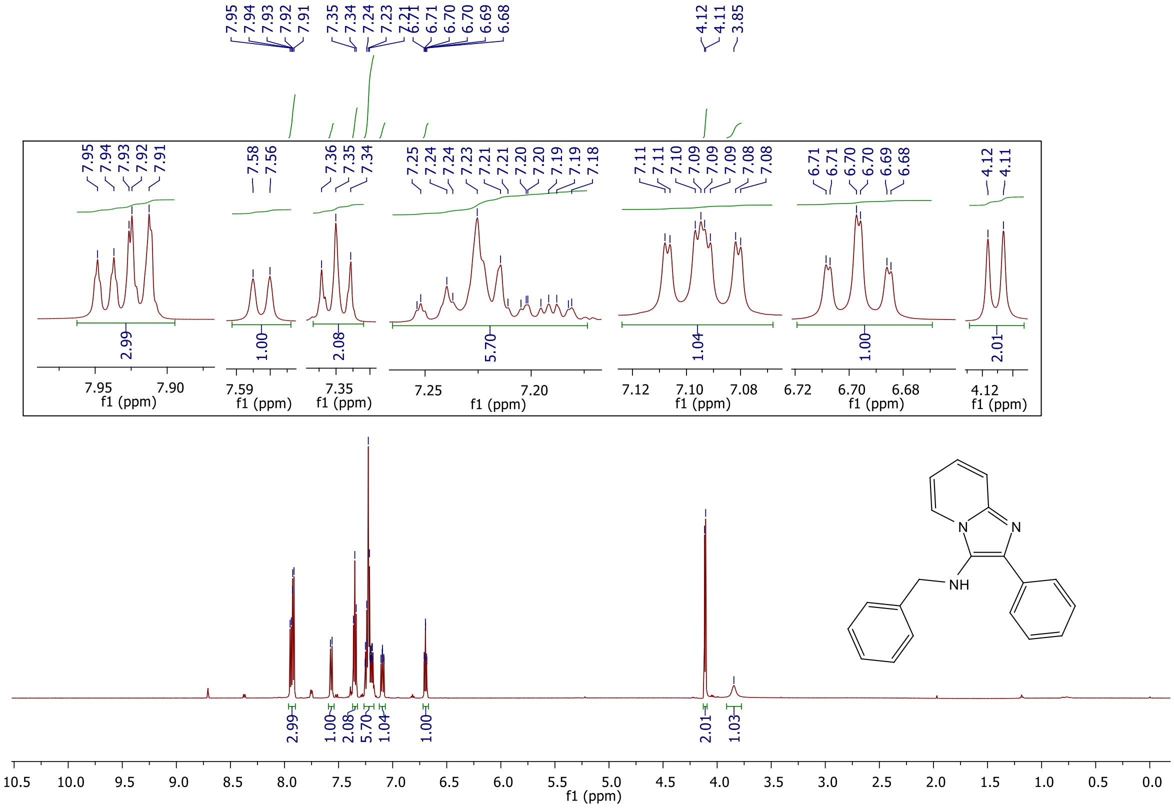

Figure S55. $^{13}C\{^{1}H\}$ NMR of compound **1q** (150 MHz, $CDCl_3$).

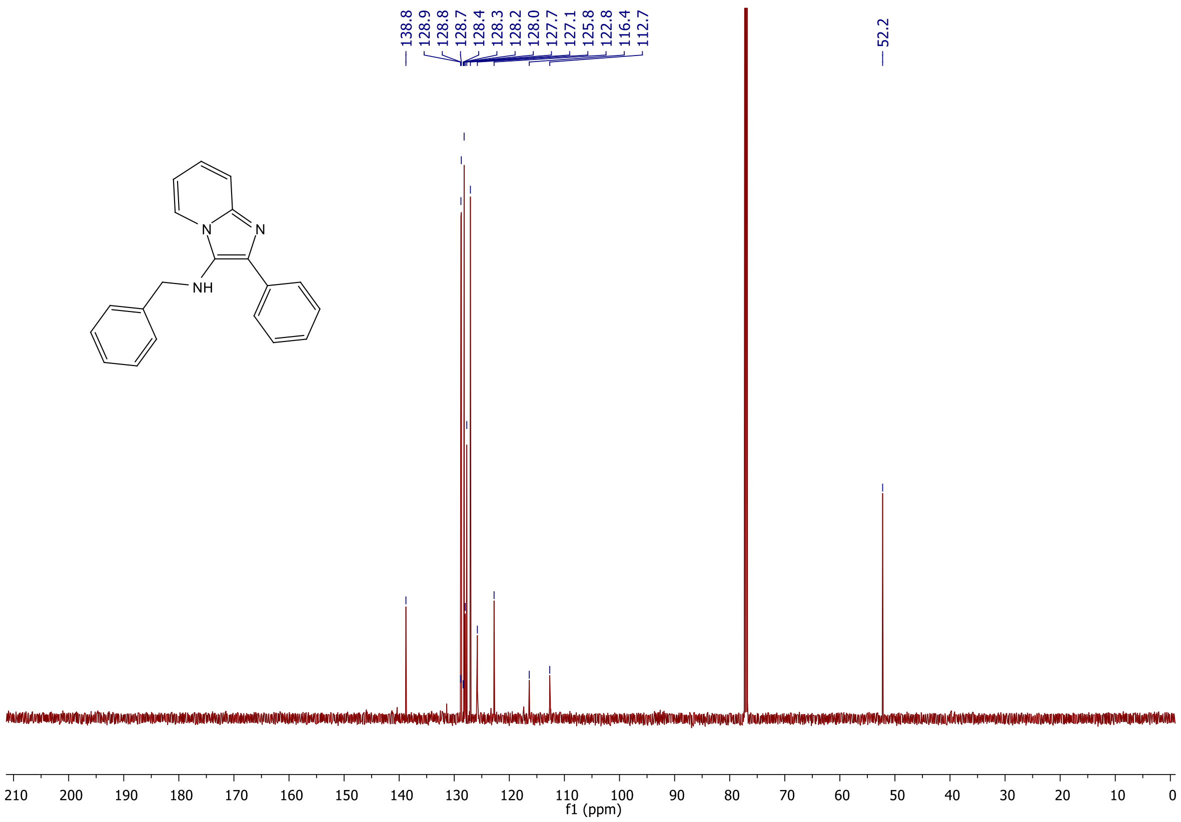

Figure S56. IR (ATR) of compound **1q**.

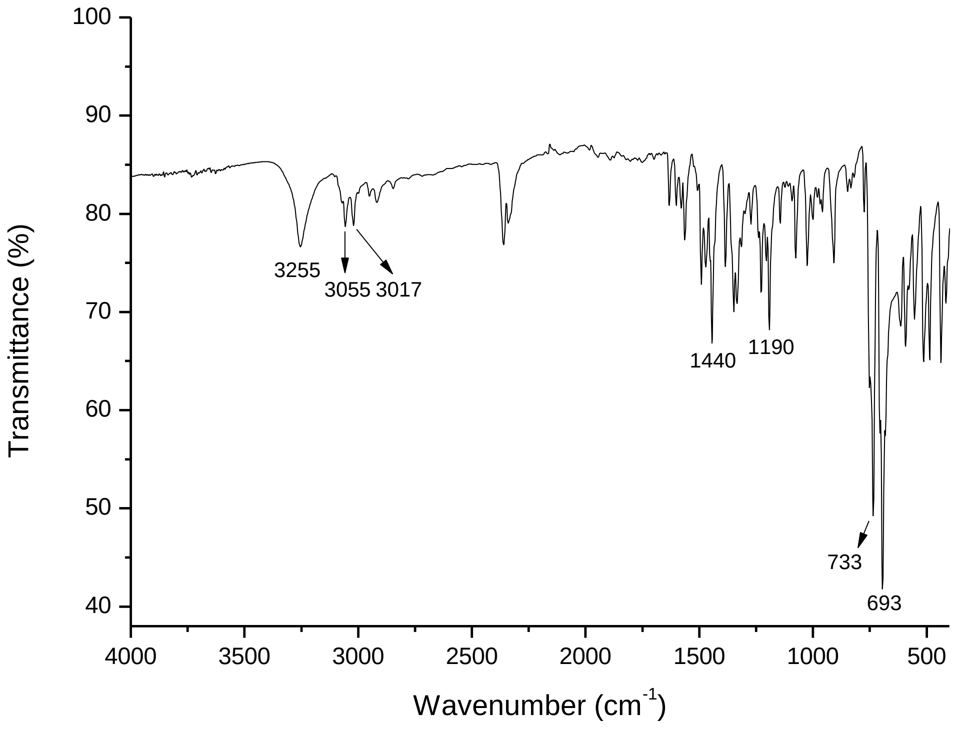

3. Mass spectrometry, UV-Vis and fluorescence emission spectra

3.1. UV-Vis and fluorescence emission spectra of compounds 1n-1o

Figure S57. UV-Vis and fluorescence emission of **1n** (70 mm).

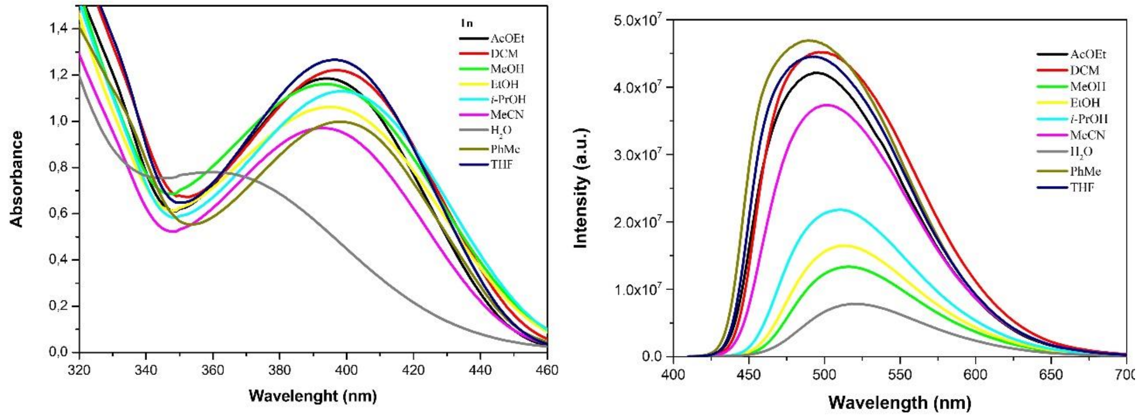

Figure S58. UV-Vis and fluorescence emission of **1o** (70 mm).

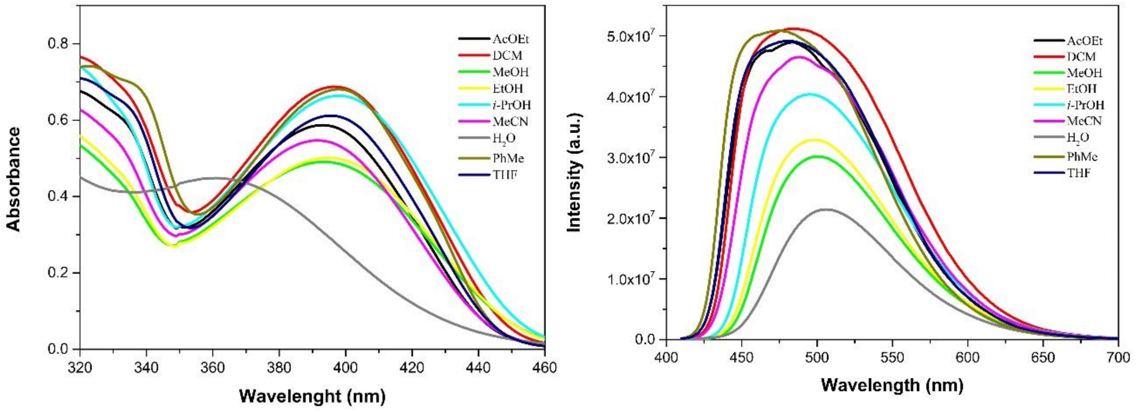

Figure S59. Photostability of **1n** and **1o** (respectively) acquired in aqueous media at room temperature upon excitation at 256 nm while monitoring the emission at their emission maxima. Time-dependence of photoluminescence intensity was carried out with a Xe lamp (400 W).

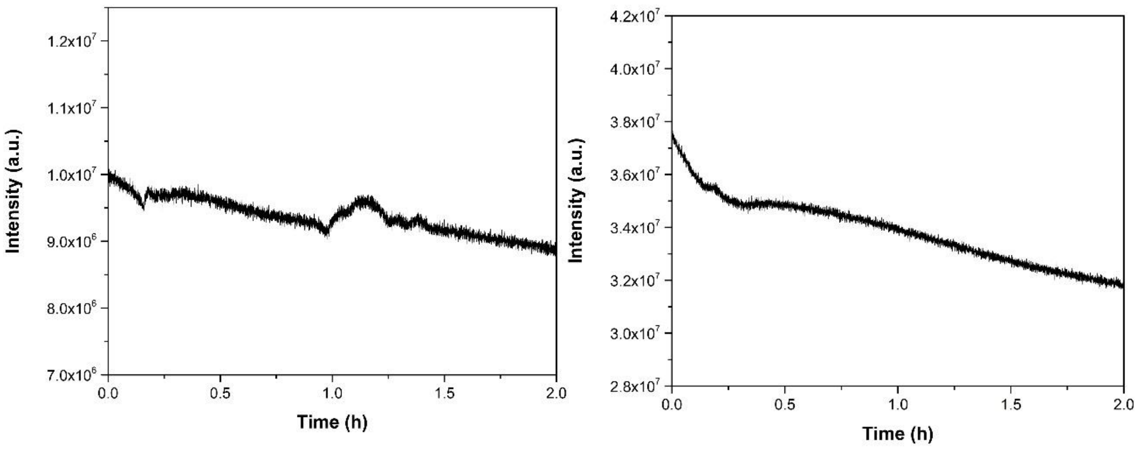

3.2. Additional mass spectrometry spectra

Figure S60. Time ESI(+)-MS monitoring of the catalyzed (PTSA) GBB reaction using a charge-tagged aldehyde derivative.

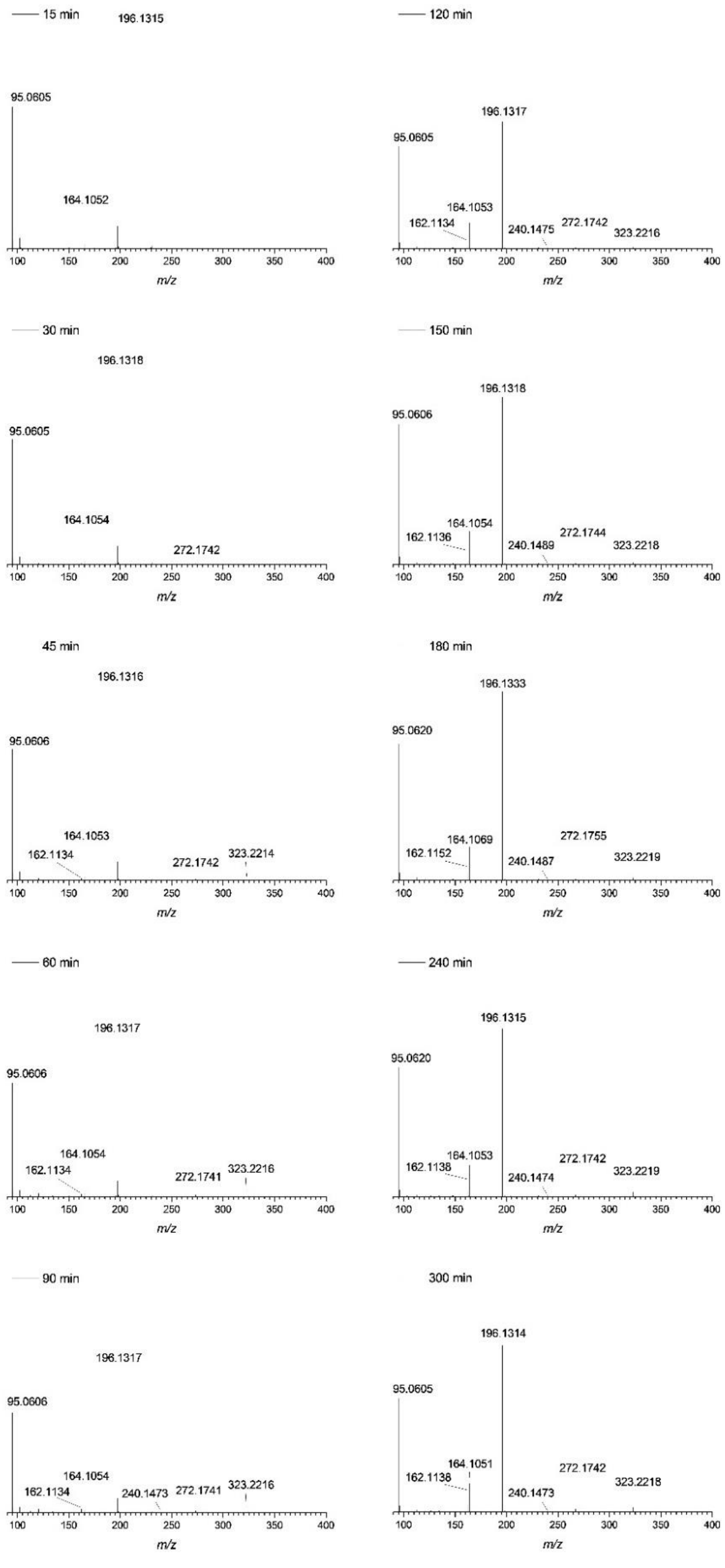

Figure S61. ESI(+)-MS/MS of the doubly charged signal of *m/z* 162.

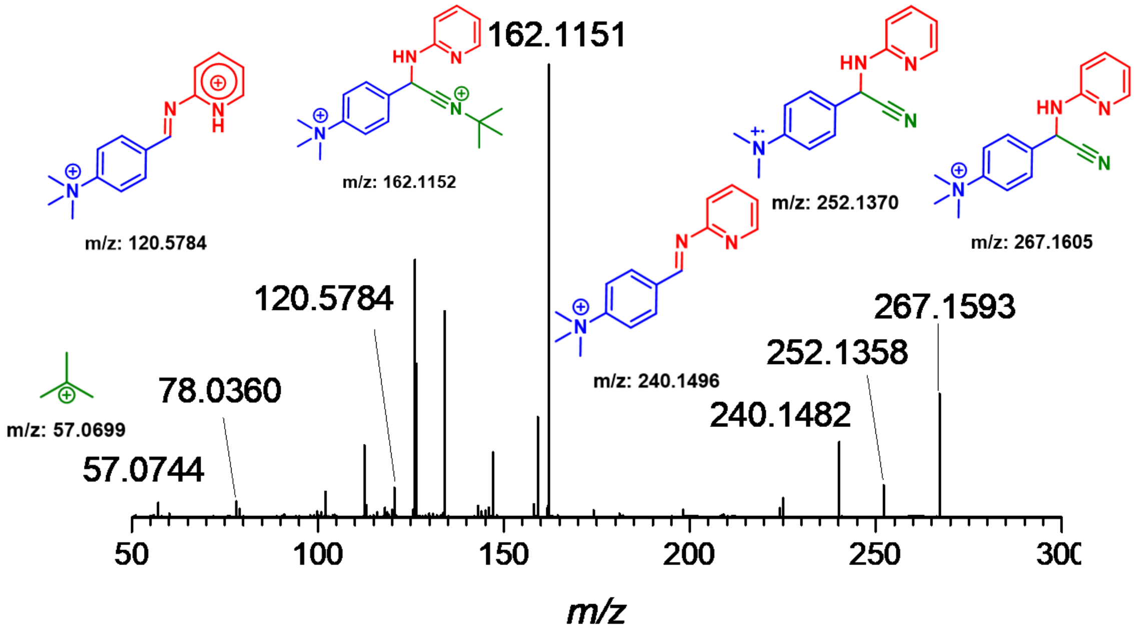

Figure S62. ESI(+)-MS/MS of the signal of *m/z* 164.

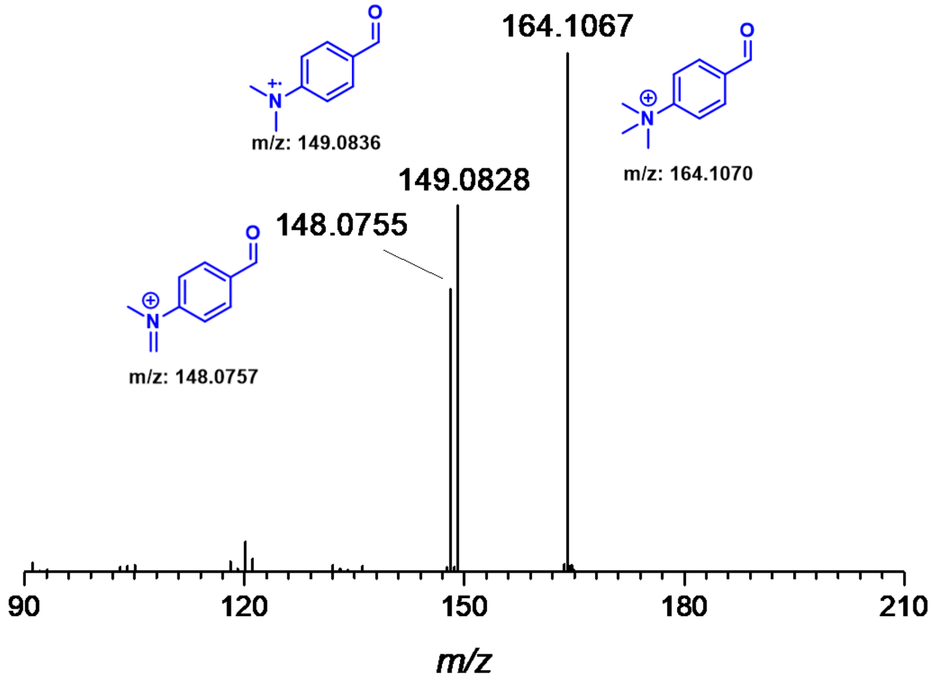

Figure S63. ESI(+)-MS/MS of the signal of *m/z* 196.

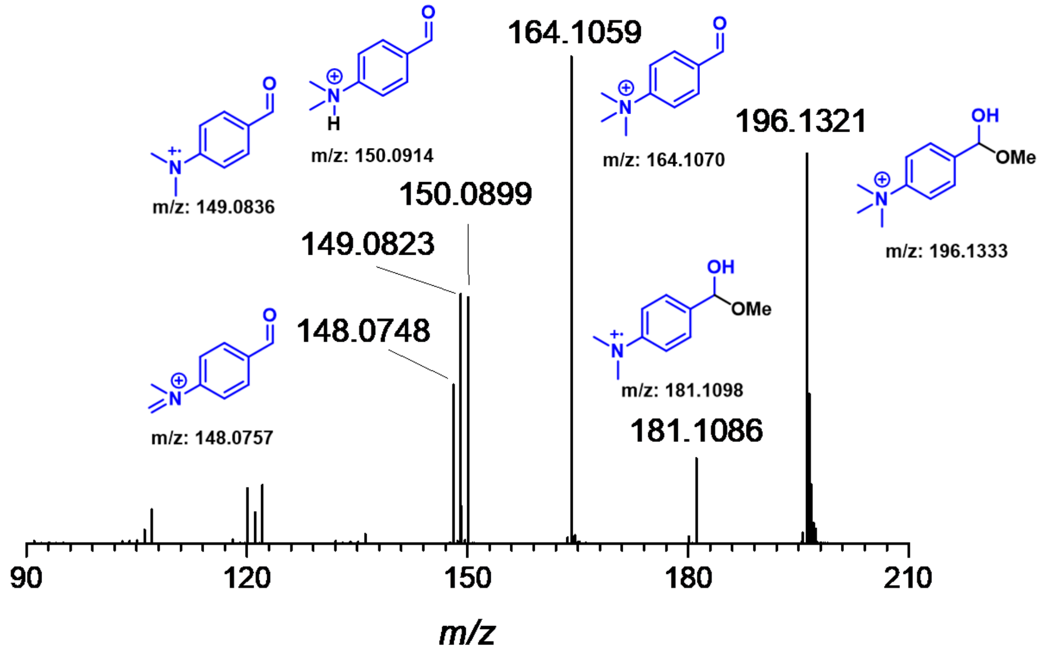

Figure S64. ESI(+)-MS/MS of the signal of *m/z* 240.

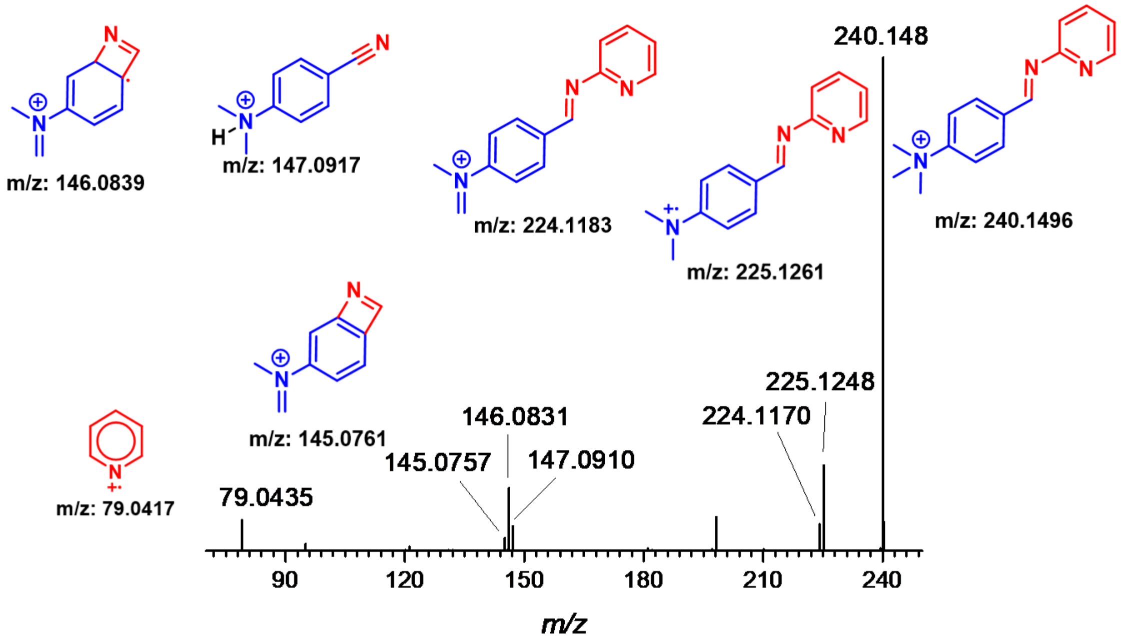

Figure S65. ESI(+)-MS/MS of the signal of *m/z* 272.

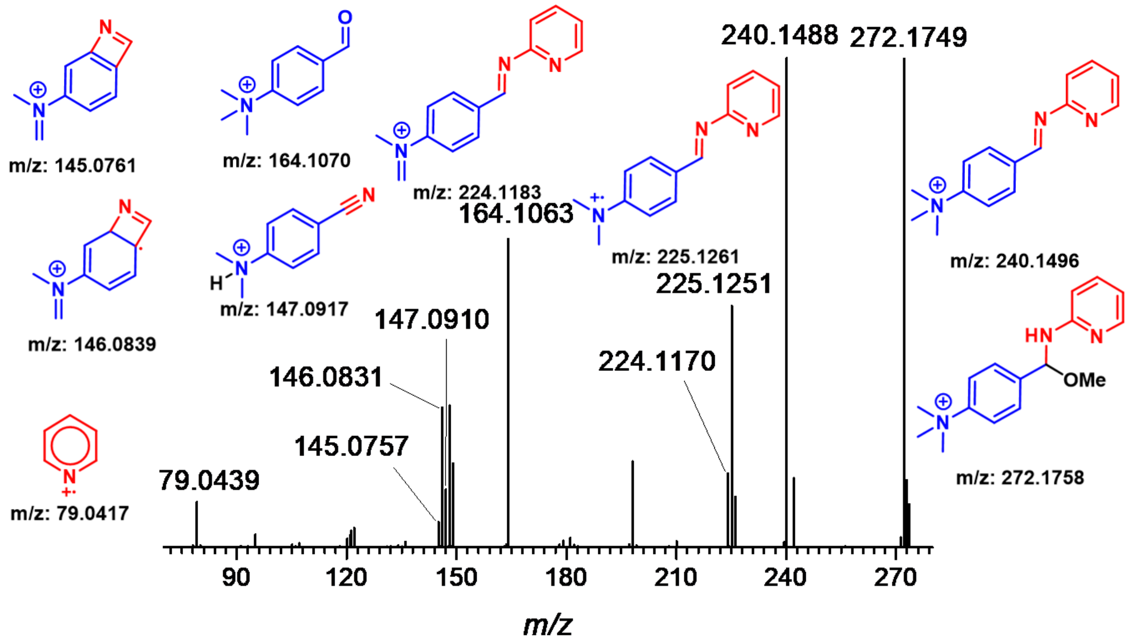

Figure S66. ESI(+)-MS/MS of the signal of *m/z* 323.

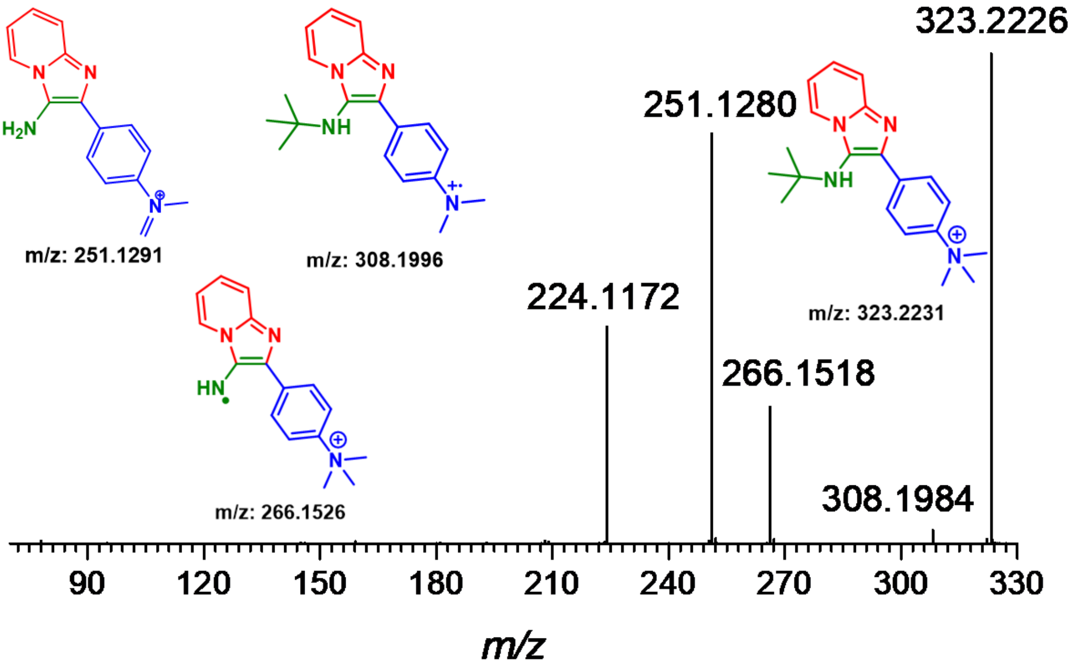

4. General overview of the evaluated mechanisms

4.1. Proposal A: Classic GBB reaction

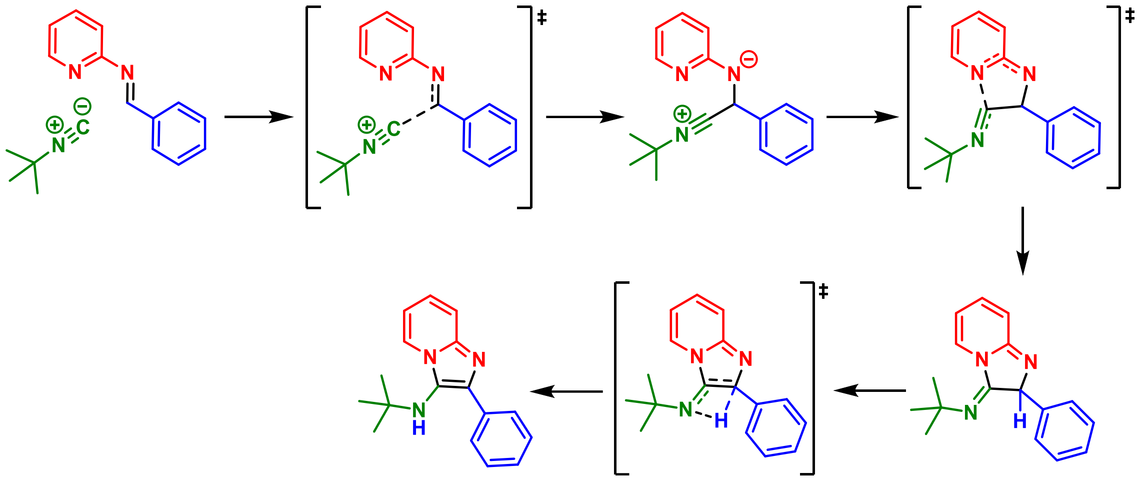

4.2. Proposal B: GBB reaction, 2-aminopyridine acts as a proton shuttle in the third step

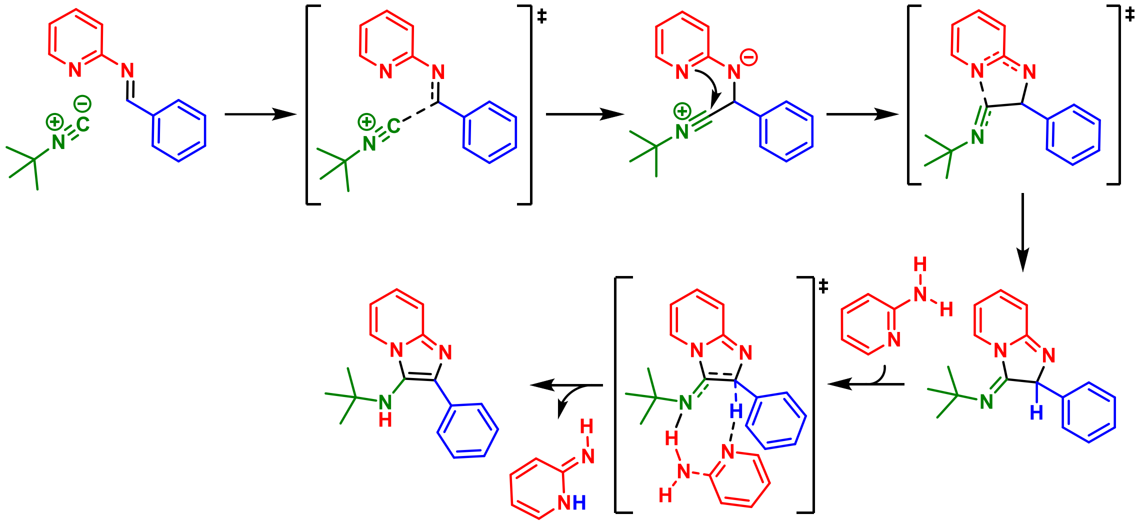

4.3. Proposal C: GBB reaction, methanol addition to nitrilium in the second step, protonating the amine

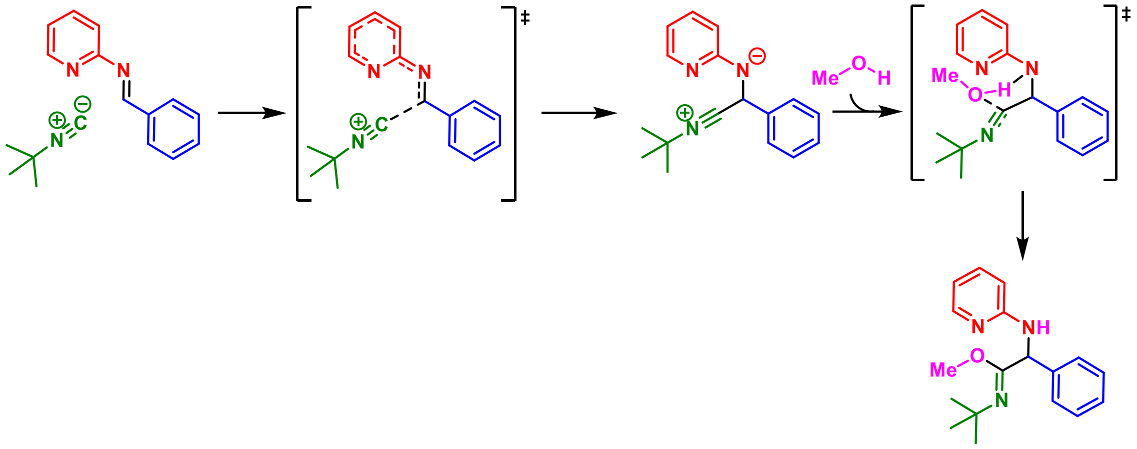

4.4. Proposal D: GBB reaction, methanol addition to nitrilium in the second step, protonating the pyridine

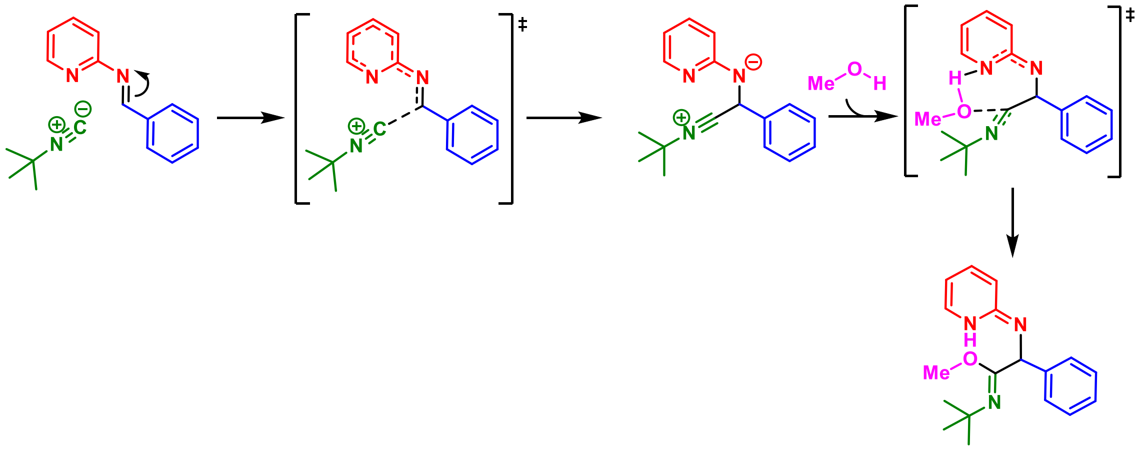

4.5. Proposal E: GBB reaction, methanol acts as a hydrogen bond donor in the first and second steps, and as a proton shuttle in the third step

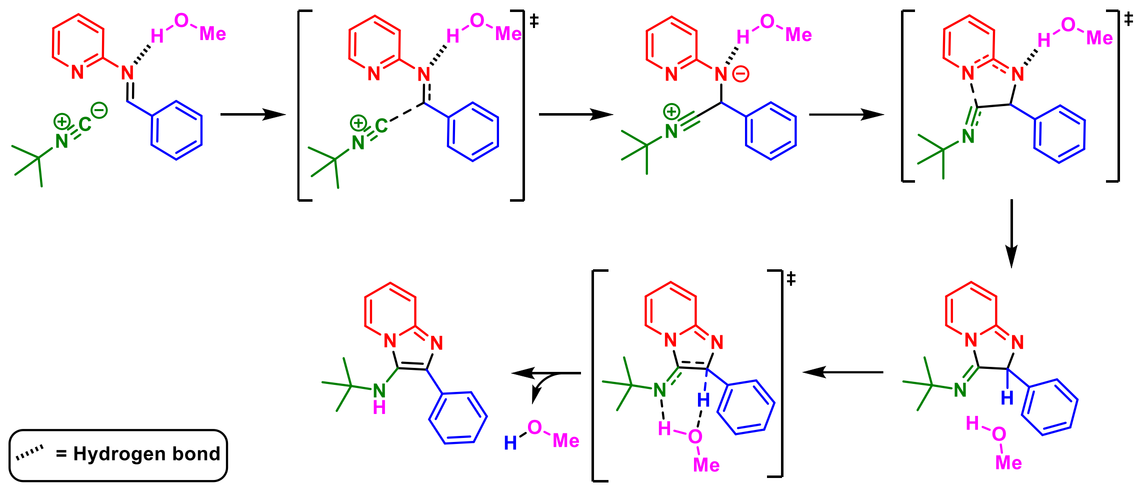

4.6. Proposal F: GBB reaction, methanol acts as a proton shuttle in the third step

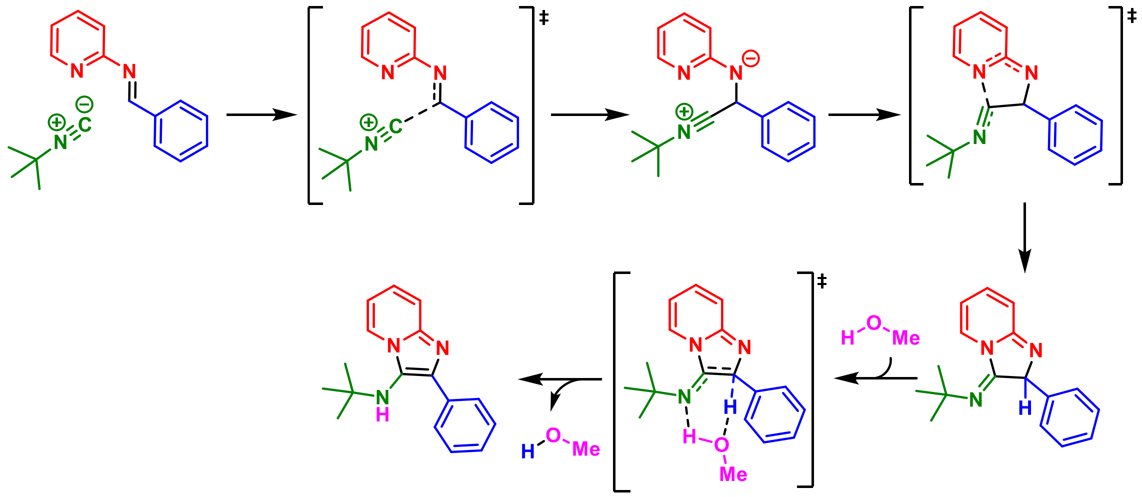

4.7. Proposal G: GBB reaction, methanol acts as a hydrogen bond donor in the first and second steps, and 2-aminopyridine acts a proton shuttle in the third step

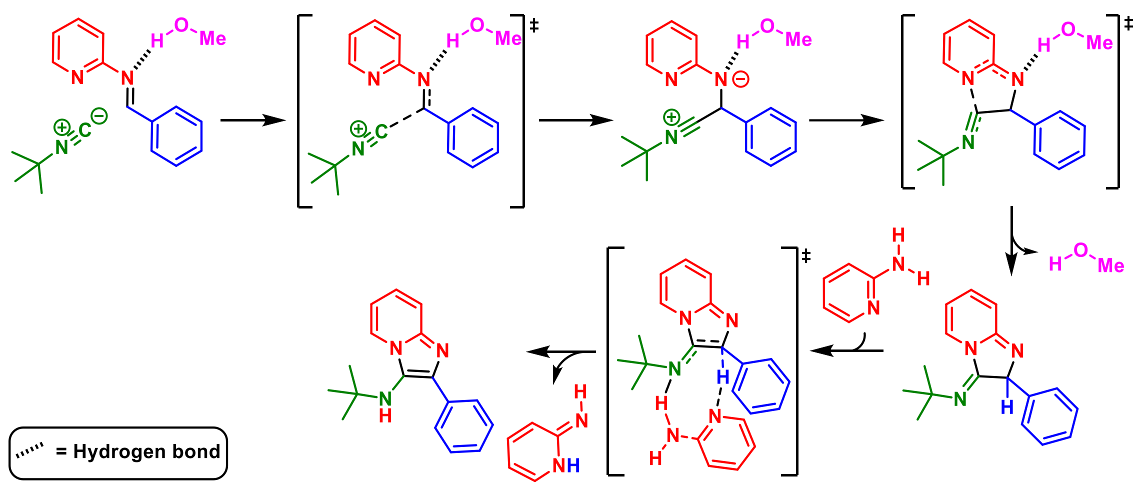

4.8. Proposal H: GBB reaction catalyzed by *p*-toluenesulfonic acid

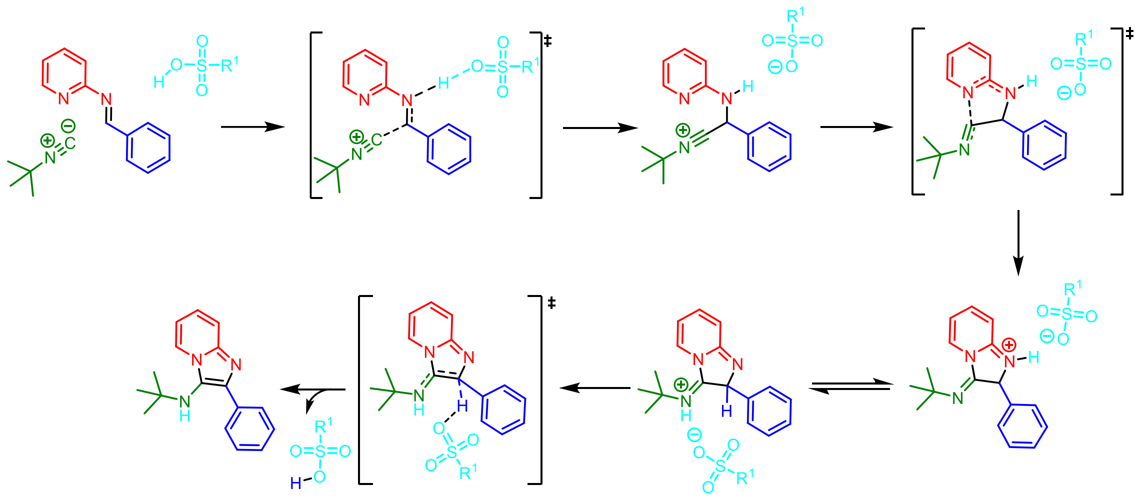

4.9. Proposal I: GBB reaction with a participation of methanol in mechanism, forming a tetrahedral intermediate, catalyzed by *p*-toluenesulfonic acid

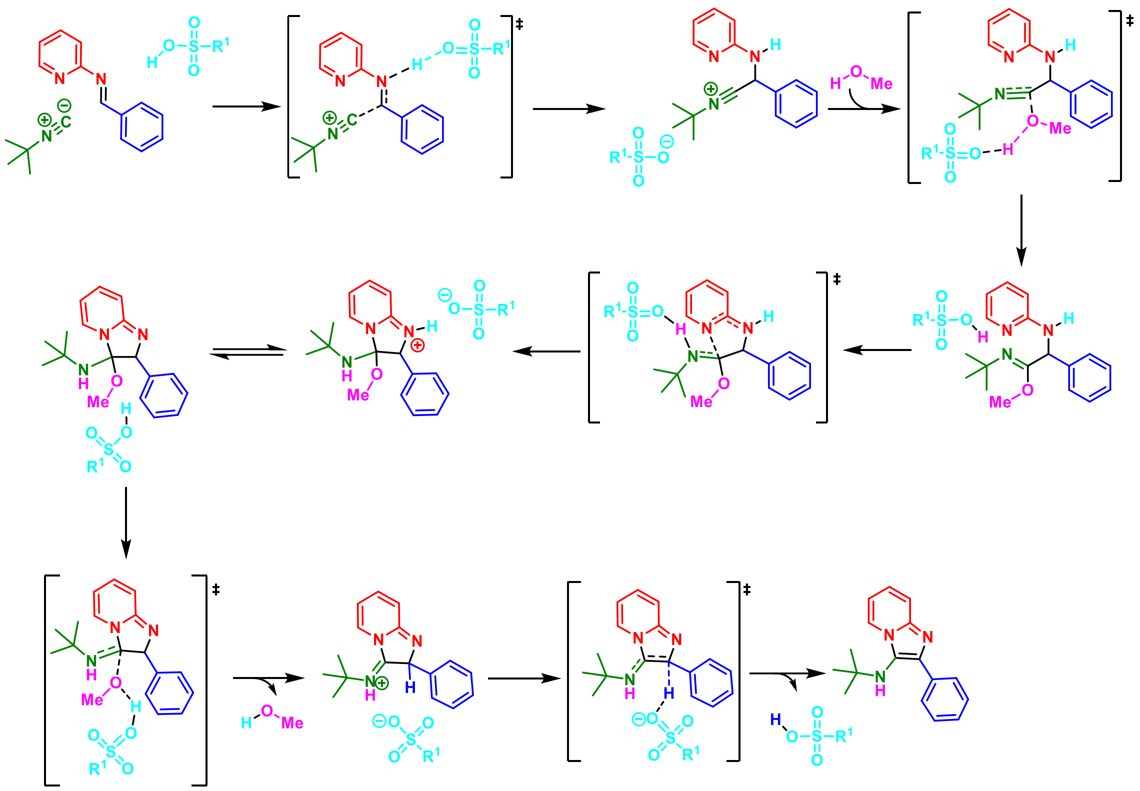

4.10. Proposal J: GBB reaction, methanol acts as a proton shuttle in the third step, catalyzed by *p*-toluenesulfonic acid

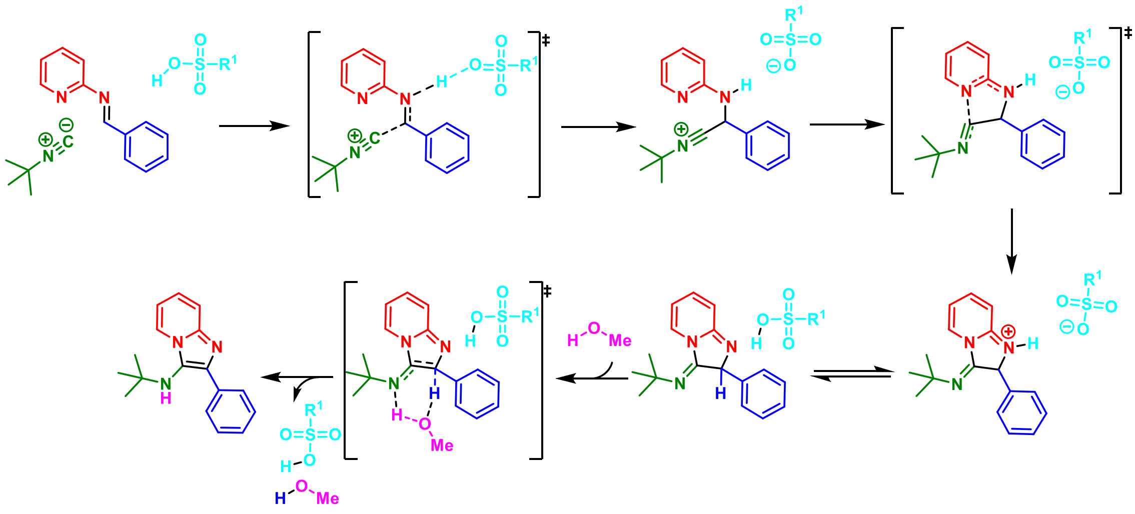

4.11. Proposal K: Methanol addition to imine, catalyzed by *p*-toluenesulfonic acid

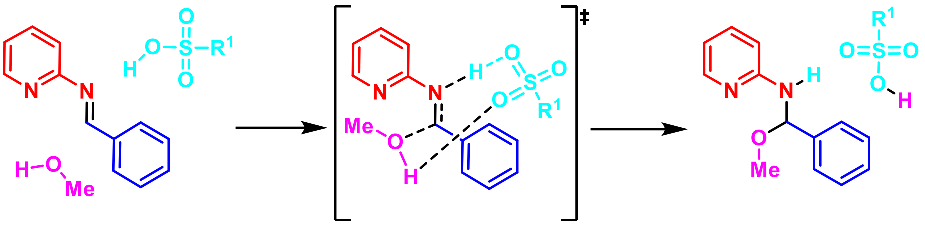

5. Energy profile (ΔG) of the evaluated mechanisms

Figure S67. Proposal A: Classic GBB reaction.

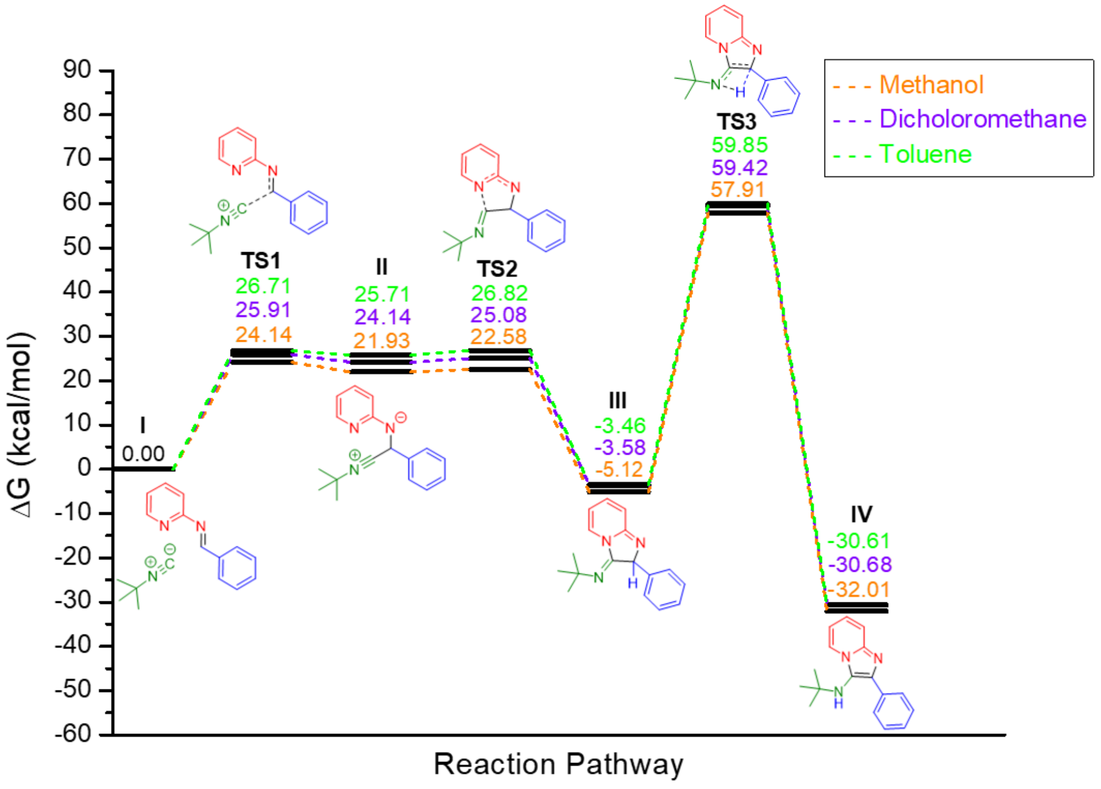

Figure S68. Proposal B: GBB reaction, the 2-aminopyridine acts as a proton shuttle in the third step.

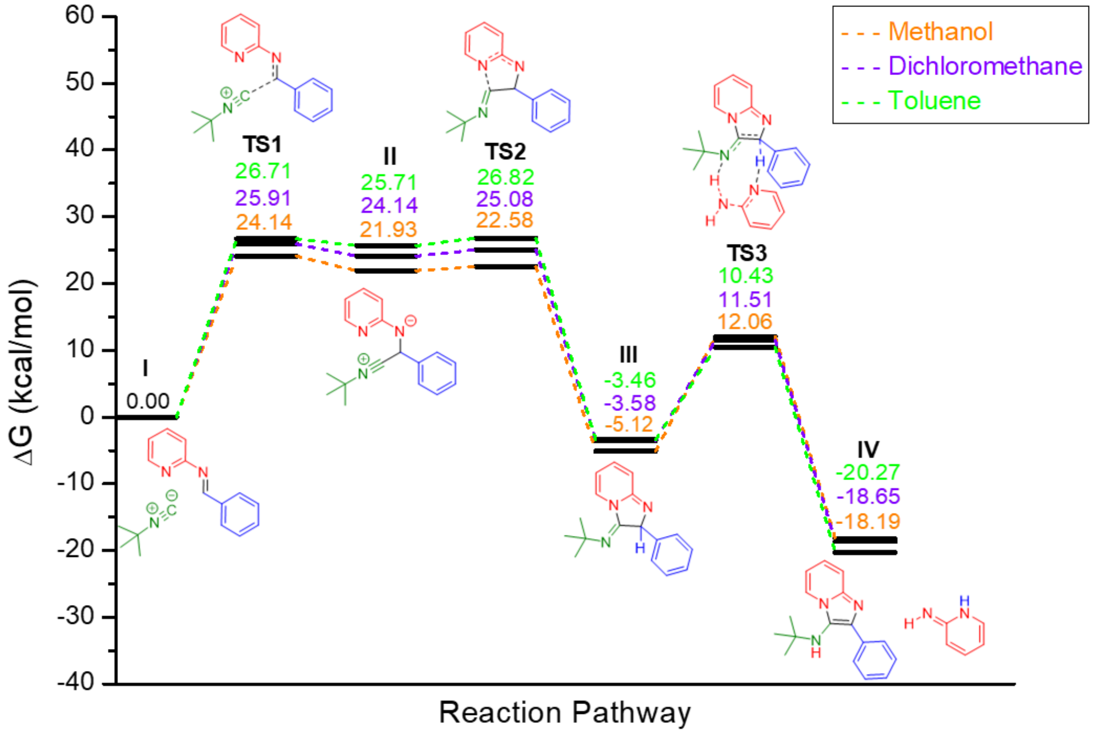

Figure S69. Proposal C: GBB reaction, methanol addition to nitrilium in the second step, protonating the amine.

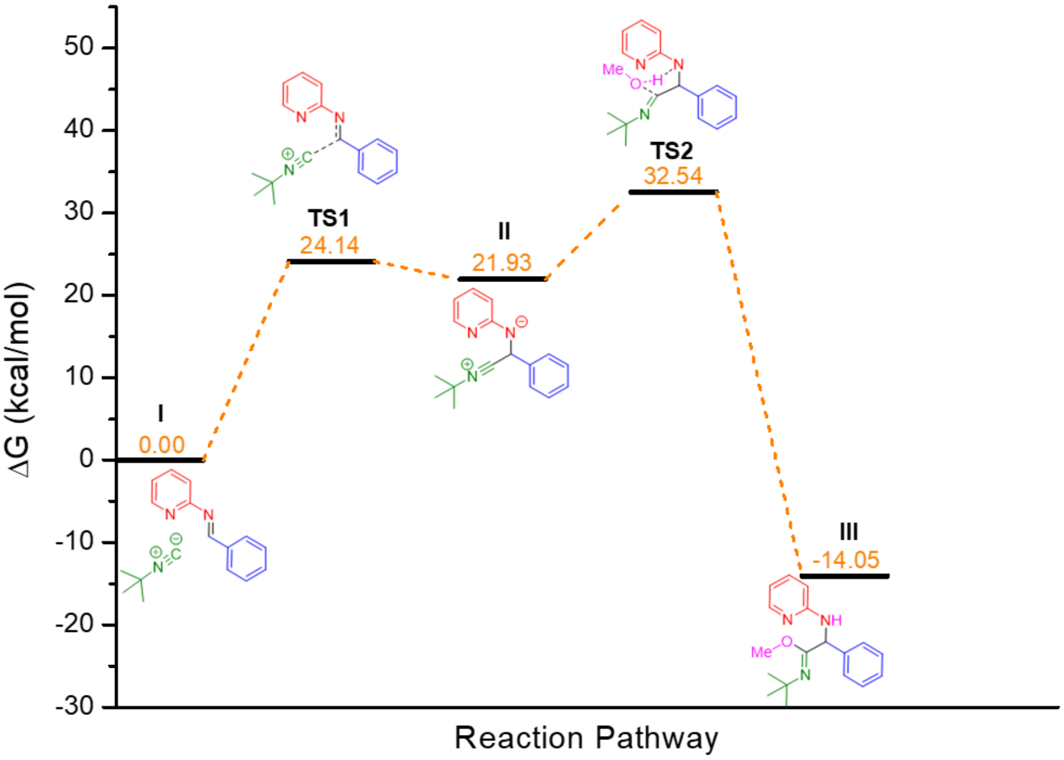

Figure S70. Proposal D: GBB reaction, methanol addition to nitrilium in the second step, protonating the pyridine.

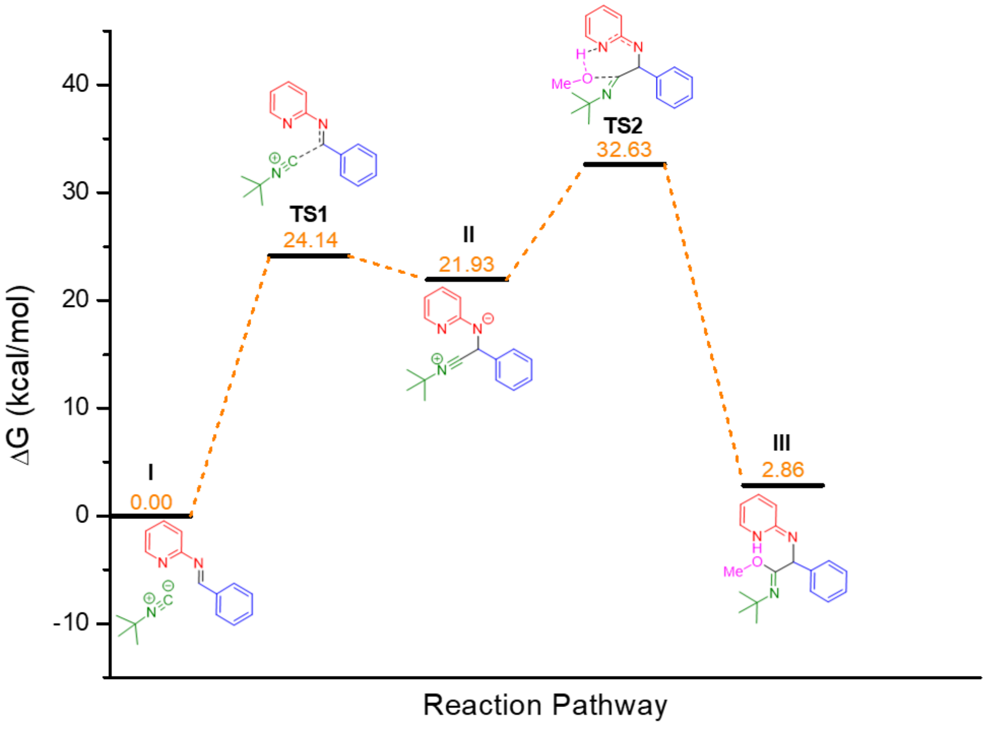

Figure S71. Proposal E: GBB reaction, methanol acts as a hydrogen bond donor in the first and second steps, and as a proton shuttle in the third step.

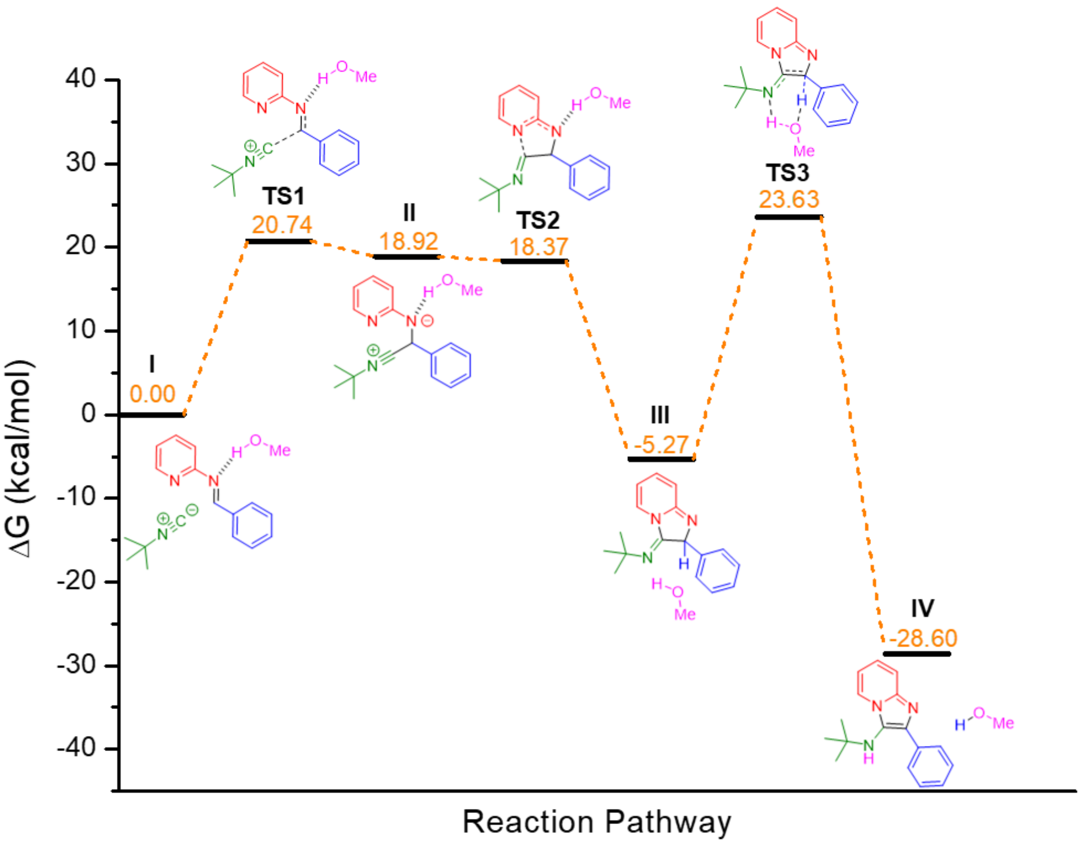

Figure S72. Proposal F: GBB reaction, methanol acts as a proton shuttle in the third step.

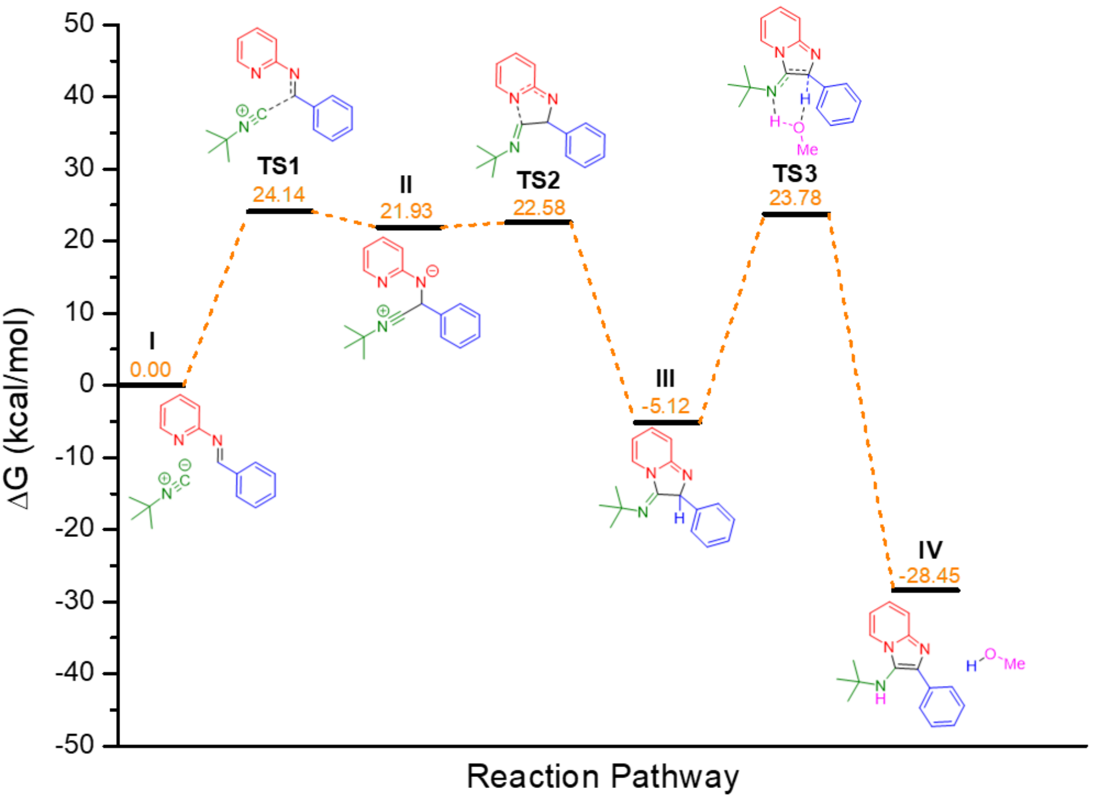

Figure S73. Proposal G: GBB reaction, methanol acts as a hydrogen bond donor in the first and second steps, and 2-aminopyridine acts a proton shuttle in the third step.

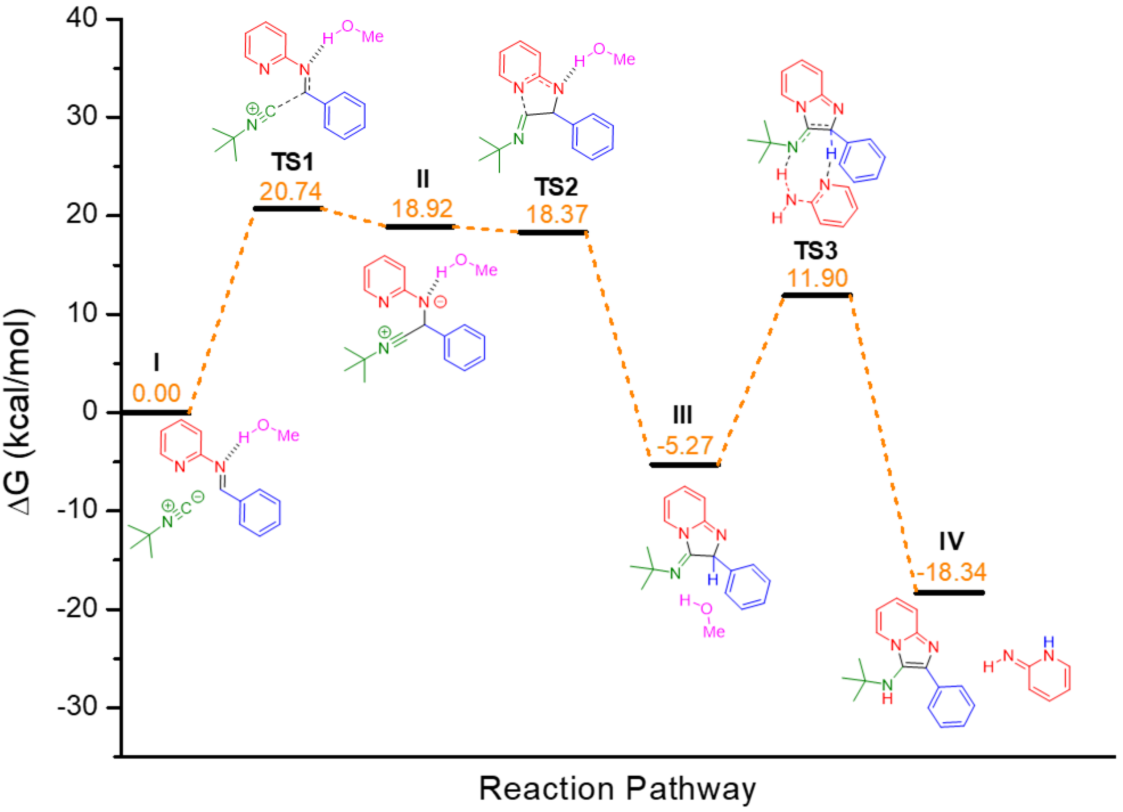

Figure S74. Proposal H: GBB reaction catalyzed by *p*-toluenesulfonic acid.

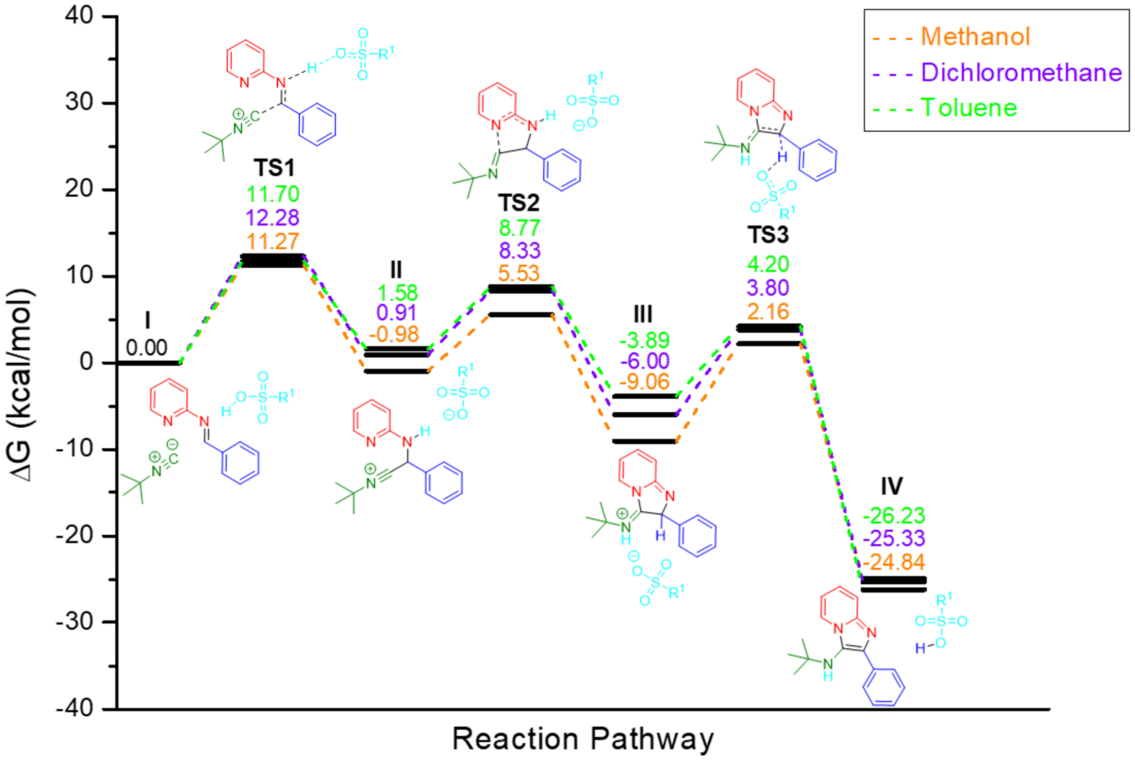

Figure S75. Proposal I: GBB reaction with a participation of methanol in mechanism, forming a tetrahedral intermediate, catalyzed by *p*-toluenesulfonic acid.

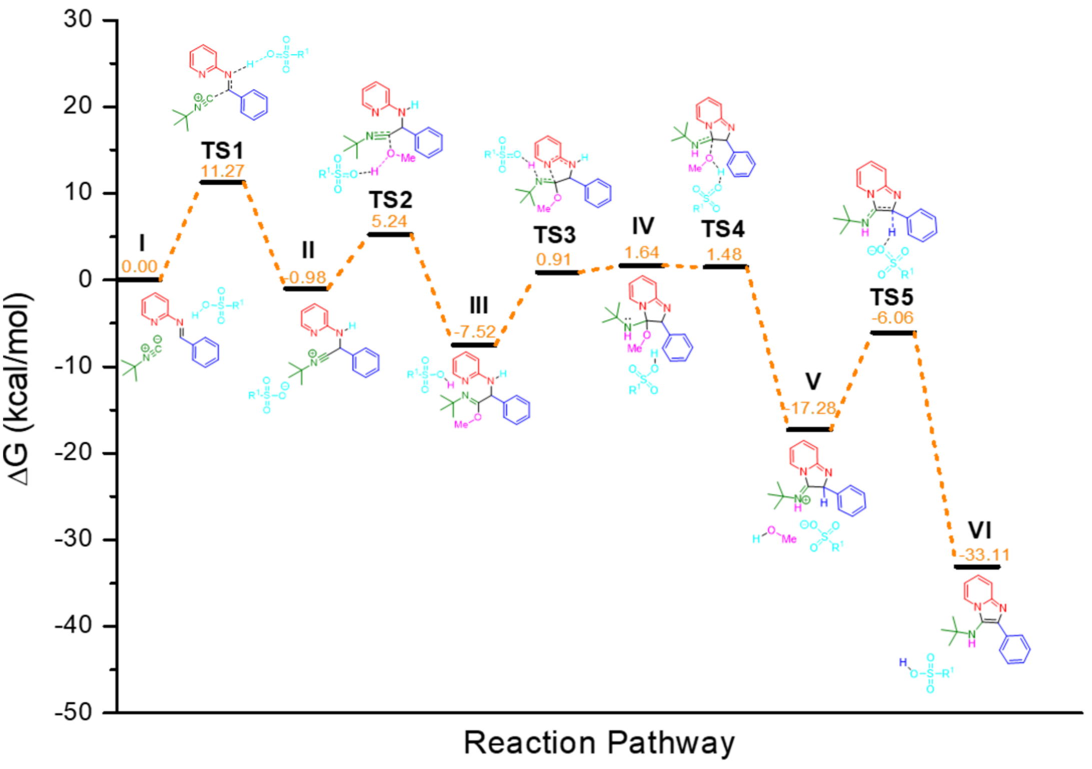

Figure S76. Proposal J: GBB reaction, methanol acts as a proton shuttle in the third step, catalyzed by *p*-toluenesulfonic acid.

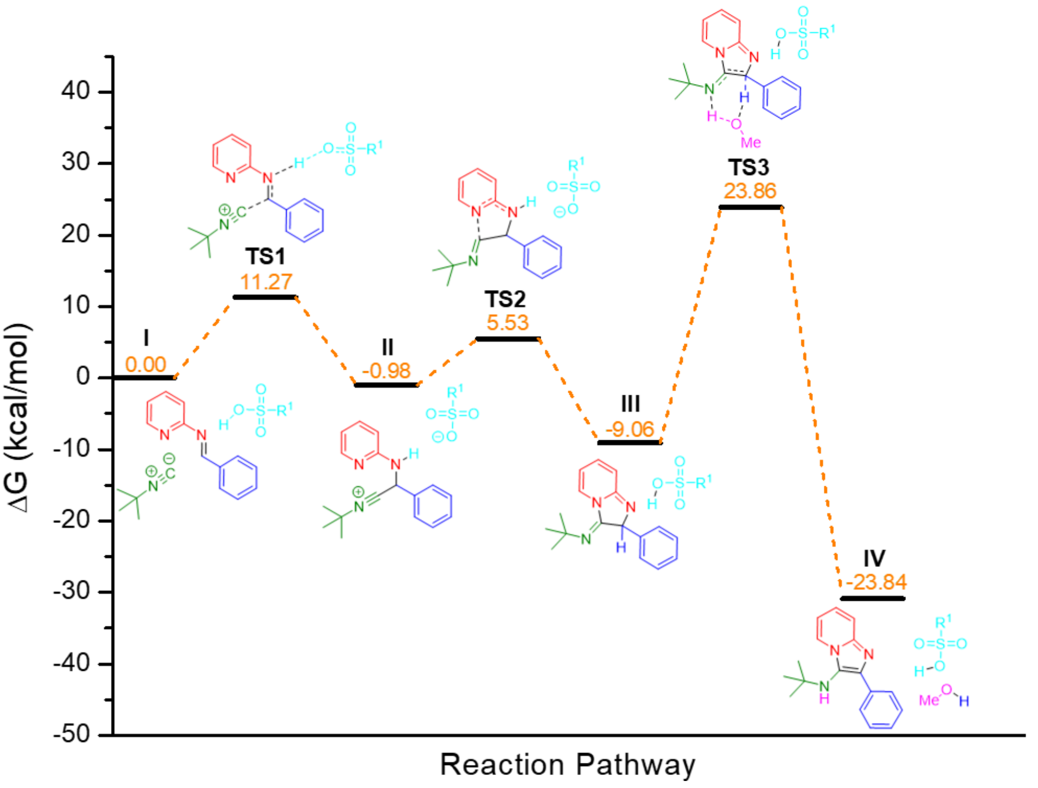

Figure S77. Proposal K: Methanol addition to imine, catalyzed by *p*-toluenesulfonic acid

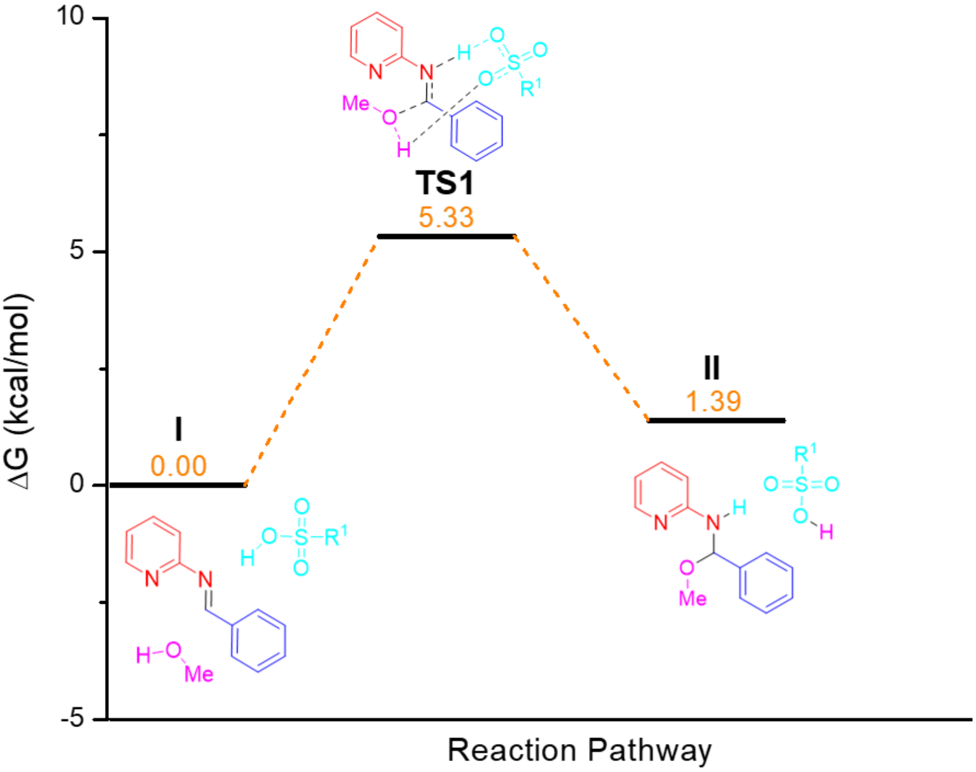
10
5
0
-5
ΔG (kcal/mol)
Reaction Pathway
TS1
5.33
I
0.00
II
1.39
N
H
O
S
O
R1
Me
Me

6. Imaginary frequencies for all transition states

6.1. Proposal A: Classic GBB reaction

Table S2. Imaginary frequencies of TS structures.

Transition State	Frequency (cm^{-1})
**Transition state 1**	-311.32
**Transition state 2**	-160.94
**Transition state 3**	-2074.28

6.2. Proposal B: GBB reaction, 2-aminopyridine acts as a proton shuttle in the third step

Table S3. Imaginary frequencies of TS structures.

Transition State	Frequency (cm^{-1})
**Transition state 1**	-311.32
**Transition state 2**	-160.94
**Transition state 3**	-1093.60

6.3. Proposal C: GBB reaction, methanol addition to nitrilium in the second step, protonating the amine

Table S4. Imaginary frequencies of TS structures.

Transition State	Frequency (cm^{-1})
**Transition state 1**	-311.32
**Transition state 2**	-895.64

6.4. Proposal D: GBB reaction, methanol addition to nitrilium in the second step, protonating the pyridine

Table S5. Imaginary frequencies of TS structures.

Transition State	Frequency (cm^{-1})
**Transition state 1**	-311.32
**Transition state 2**	-369.44

6.5. Proposal E: GBB reaction, methanol acts as a hydrogen bond donor in the first and second steps, and as a proton shuttle in the third step

Table S6. Imaginary frequencies of TS structures.

Transition State	Frequency (cm^{-1})
**Transition state 1**	-330.31
**Transition state 2**	-168.44
**Transition state 3**	-1716.68

6.6. Proposal F: GBB reaction, methanol acts as a proton shuttle in the third step

Table S7. Imaginary frequencies of TS structures.

Transition State	Frequency (cm^{-1})
**Transition state 1**	-311.32
**Transition state 2**	-160.94
**Transition state 3**	-1716.68

6.7. Proposal G: GBB reaction, methanol acts as a hydrogen bond donor in the first and second steps, and 2-aminopyridine acts a proton shuttle in the third step

Table S8. Imaginary frequencies of TS structures.

Transition State	Frequency (cm^{-1})
**Transition state 1**	-330.31
**Transition state 2**	-168.44
**Transition state 3**	-1093.60

6.8. Proposal H: GBB reaction catalyzed by *p*-toluenesulfonic acid

Table S9. Imaginary frequencies of TS structures.

Transition State	Frequency (cm^{-1})
**Transition state 1**	-284.39
**Transition state 2**	-134.15
**Transition state 3**	-1048.21

6.9. Proposal I: GBB reaction with a participation of methanol in mechanism, forming a tetrahedral intermediate, catalyzed by *p*-toluenesulfonic acid

Table S10. Imaginary frequencies of TS structures.

Transition State	Frequency (cm^{-1})
**Transition state 1**	-284.39
**Transition state 2**	-160.03
**Transition state 3**	-150.78
**Transition state 4**	-224.29
**Transition state 5**	-1048.21

6.10. Proposal J: GBB reaction, methanol acts as a proton shuttle in the third step, catalyzed by *p*-toluenesulfonic acid

Table S11. Imaginary frequencies of TS structures.

Transition State	Frequency (cm^{-1})
**Transition state 1**	-284.39
**Transition state 2**	-134.15
**Transition state 3**	-1588.46

6.11. Proposal K: Methanol addition to imine, catalyzed by p-toluenesulfonic acid

Table S12. Imaginary frequencies of TS structures.

Transition State	Frequency (cm^{-1})
**Transition state 1**	-220.88

7. Electronic energies (E), Enthalpies (H) and Gibbs free energies (G) of all optimized structures

Note: All energies values presented below are expressed in Hartree. The energies were calculated using M06-2X/6-31++G(d,p)/SMD=methanol (dichloromethane and toluene were used in some cases) //M06-2X/6-31++G(d,p) level of theory.

7.1. Proposal A: Classic GBB reaction

Table S13. Electronic energies (ΔE), Enthalpies (ΔH) and Gibbs free energies (ΔG) obtained for the solvent methanol.

		E	H	G
	**Molecular complex 1**	-823.1730524	-822.8270294	-822.8984974
**Step 1**	**Transition state 1**	-823.1370267	-822.7922367	-822.8600267
	**Molecular complex 2**	-823.141283	-822.795328	-822.863551
	**Molecular complex 3**	-823.1412759	-822.7953209	-822.8635369
**Step 2**	**Transition state 2**	-823.1408684	-822.7958044	-822.8625074
	**Molecular complex 4**	-823.189624	-822.841384	-822.906646
	**Molecular complex 5**	-823.1896285	-822.8413885	-822.9066565
**Step 3**	**Transition state 3**	-823.0891787	-822.7476297	-822.8062067
	**Molecular complex 6**	-823.2352606	-822.8861356	-822.9495076

Table S14. Electronic energies (ΔE), Enthalpies (ΔH) and Gibbs free energies (ΔG) obtained for the solvent dichloromethane.

		E	H	G
	**Molecular complex 1**	-823.1803009	-822.8342779	-822.9057459
**Step 1**	**Transition state 1**	-823.1414636	-822.7966736	-822.8644636
	**Molecular complex 2**	-823.1450034	-822.7990484	-822.8672714
	**Molecular complex 3**	-823.1449972	-822.7990422	-822.8672582
**Step 2**	**Transition state 2**	-823.1441224	-822.7990584	-822.8657614
	**Molecular complex 4**	-823.1944093	-822.8461693	-822.9114313
	**Molecular complex 5**	-823.1944094	-822.8461694	-822.9114374
**Step 3**	**Transition state 3**	-823.0940223	-822.7524733	-822.8110503
	**Molecular complex 6**	-823.2403771	-822.8912521	-822.9546241

Table S15. Electronic energies (ΔE), Enthalpies (ΔH) and Gibbs free energies (ΔG) obtained for the solvent toluene.

		E	H	G
**Step 1**	**Molecular complex 1**	-823.173261	-822.827238	-822.898706
	**Transition state 1**	-823.133133	-822.788343	-822.856133
	**Molecular complex 2**	-823.1354716	-822.7895166	-822.8577396
**Step 2**	**Molecular complex 3**	-823.1354678	-822.7895128	-822.8577288
	**Transition state 2**	-823.1343231	-822.7892591	-822.8559621
	**Molecular complex 4**	-823.1871851	-822.8389451	-822.9042071
**Step 3**	**Molecular complex 5**	-823.1871822	-822.8389422	-822.9042102
	**Transition state 3**	-823.0862902	-822.7447412	-822.8033182
	**Molecular complex 6**	-823.2332284	-822.8841034	-822.9474754

7.2. Proposal B: GBB reaction, 2-aminopyridine acts as a proton shuttle in the third step

Table S16. Electronic energies (ΔE), Enthalpies (ΔH) and Gibbs free energies (ΔG) obtained for the solvent methanol.

		E	H	G
**Step 1**	**Molecular complex 1**	-823.1730524	-822.8270294	-822.8984974
	**Transition state 1**	-823.1370267	-822.7922367	-822.8600267
	**Molecular complex 2**	-823.141283	-822.795328	-822.863551
**Step 2**	**Molecular complex 3**	-823.1412759	-822.7953209	-822.8635369
	**Transition state 2**	-823.1408684	-822.7958044	-822.8625074
	**Molecular complex 4**	-823.189624	-822.841384	-822.906646
**Step 3**	**Molecular complex 5**	-1126.75268	-1126.289229	-1126.372762
	**Transition state 3**	-1126.721436	-1126.26426	-1126.345385
	**Molecular complex 6**	-1126.775578	-1126.310997	-1126.393582

Table S17. Electronic energies (ΔE), Enthalpies (ΔH) and Gibbs free energies (ΔG) obtained for the solvent dichloromethane.

		E	H	G
	**Molecular complex 1**	-823.1803009	-822.8342779	-822.9057459
**Step 1**	**Transition state 1**	-823.1414636	-822.7966736	-822.8644636
	**Molecular complex 2**	-823.1450034	-822.7990484	-822.8672714
	**Molecular complex 3**	-823.1449972	-822.7990422	-822.8672582
**Step 2**	**Transition state 2**	-823.1441224	-822.7990584	-822.8657614
	**Molecular complex 4**	-823.1944093	-822.8461693	-822.9114313
	**Molecular complex 5**	-1126.757275	-1126.293824	-1126.377357
**Step 3**	**Transition state 3**	-1126.72937	-1126.272194	-1126.353319
	**Molecular complex 6**	-1126.783375	-1126.318794	-1126.401379

Table S18. Electronic energies (ΔE), Enthalpies (ΔH) and Gibbs free energies (ΔG) obtained for the solvent toluene.

		E	H	G
	**Molecular complex 1**	-823.173261	-822.827238	-822.898706
**Step 1**	**Transition state 1**	-823.133133	-822.788343	-822.856133
	**Molecular complex 2**	-823.1354716	-822.7895166	-822.8577396
	**Molecular complex 3**	-823.1354678	-822.7895128	-822.8577288
**Step 2**	**Transition state 2**	-823.1343231	-822.7892591	-822.8559621
	**Molecular complex 4**	-823.1871851	-822.8389451	-822.9042071
	**Molecular complex 5**	-1126.745954	-1126.282503	-1126.366036
**Step 3**	**Transition state 3**	-1126.719956	-1126.26278	-1126.343905
	**Molecular complex 6**	-1126.774815	-1126.310234	-1126.392819

7.3. Proposal C: GBB reaction, methanol addition to nitrilium in the second step, protonating the amine

Table S19. Electronic energies (ΔE), Enthalpies (ΔH) and Gibbs free energies (ΔG) obtained for the solvent methanol.

		E	H	G
	**Molecular complex 1**	-823.1730524	-822.8270294	-822.8984974
**Step 1**	**Transition state 1**	-823.1370267	-822.7922367	-822.8600267
	**Molecular complex 2**	-823.141283	-822.795328	-822.863551
	**Molecular complex 3**	-938.833538	-938.429231	-938.508634
**Step 2**	**Transition state 2**	-938.8133072	-938.4132942	-938.4887042
	**Molecular complex 4**	-938.8994494	-938.4923954	-938.5659724

7.4. Proposal D: GBB reaction, methanol addition to nitrilium in the second step, protonating the pyridine

Table S120. Electronic energies (ΔE), Enthalpies (ΔH) and Gibbs free energies (ΔG) obtained for the solvent methanol.

		E	H	G
	**Molecular complex 1**	-823.1730524	-822.8270294	-822.8984974
**Step 1**	**Transition state 1**	-823.1370267	-822.7922367	-822.8600267
	**Molecular complex 2**	-823.141283	-822.795328	-822.863551
	**Molecular complex 3**	-938.8395389	-938.4348989	-938.5109509
**Step 2**	**Transition state 2**	-938.8190418	-938.4179818	-938.4908768
	**Molecular complex 4**	-938.8735948	-938.4665428	-938.5413458

7.5. Proposal E: GBB reaction, methanol acts as a hydrogen bond donor in the first and second steps, and as a proton shuttle in the third step

Table S21. Electronic energies (ΔE), Enthalpies (ΔH) and Gibbs free energies (ΔG) obtained for the solvent methanol.

		E	H	G
**Step 1**	**Molecular complex 1**	-938.863881	-938.4593936	-938.5408986
	**Transition state 1**	-938.832095	-938.4287998	-938.5048118
	**Molecular complex 2**	-938.836476	-938.4321203	-938.5107533
**Step 2**	**Molecular complex 3**	-938.836340	-938.4318944	-938.5101244
	**Transition state 2**	-938.831782	-938.4282056	-938.5079646
	**Molecular complex 4**	-938.880693	-938.4740915	-938.5486745
**Step 3**	**Molecular complex 5**	-938.883148	-938.4763174	-938.5514964
	**Transition state 3**	-938.829462	-938.4304404	-938.5024134
	**Molecular complex 6**	-938.922510	-938.5148825	-938.5886685

7.6. Proposal F: GBB reaction, methanol acts as a proton shuttle in the third step

Table S22. Electronic energies (ΔE), Enthalpies (ΔH) and Gibbs free energies (ΔG) obtained for the solvent methanol.

		E	H	G
**Step 1**	**Molecular complex 1**	-823.1730524	-822.8270294	-822.8984974
	**Transition state 1**	-823.1370267	-822.7922367	-822.8600267
	**Molecular complex 2**	-823.141283	-822.795328	-822.863551
**Step 2**	**Molecular complex 3**	-823.1412759	-822.7953209	-822.8635369
	**Transition state 2**	-823.1408684	-822.7958044	-822.8625074
	**Molecular complex 4**	-823.189624	-822.841384	-822.906646
**Step 3**	**Molecular complex 5**	-938.883148	-938.4763174	-938.5514964
	**Transition state 3**	-938.829462	-938.4304404	-938.5024134
	**Molecular complex 6**	-938.922510	-938.5148825	-938.5886685

7.7. Proposal G: GBB reaction, methanol acts as a hydrogen bond donor in the first and second steps, and 2-aminopyridine acts a proton shuttle in the third step

Table S23. Electronic energies (ΔE), Enthalpies (ΔH) and Gibbs free energies (ΔG) obtained for the solvent methanol.

		E	H	G
	**Molecular complex 1**	-938.863881	-938.4593936	-938.5408986
**Step 1**	**Transition state 1**	-938.832095	-938.4287998	-938.5048118
	**Molecular complex 2**	-938.836476	-938.4321203	-938.5107533
	**Molecular complex 3**	-938.836340	-938.4318944	-938.5101244
**Step 2**	**Transition state 2**	-938.831782	-938.4282056	-938.5079646
	**Molecular complex 4**	-938.880693	-938.4740915	-938.5486745
	**Molecular complex 5**	-1126.75268	-1126.289229	-1126.372762
**Step 3**	**Transition state 3**	-1126.721436	-1126.26426	-1126.345385
	**Molecular complex 6**	-1126.775578	-1126.310997	-1126.393582

7.8. Proposal H: GBB reaction catalyzed by *p*-toluenesulfonic acid

Table S24. Electronic energies (ΔE), Enthalpies (ΔH) and Gibbs free energies (ΔG) obtained for the solvent methanol.

		E	H	G
	**Molecular complex 1**	-1718.369882	-1717.866464	-1717.968857
**Step 1**	**Transition state 1**	-1718.358756	-1717.854841	-1717.9509
	**Molecular complex 2**	-1718.3798	-1717.873757	-1717.970412
	**Molecular complex 3**	-1718.381279	-1717.875231	-1717.973305
**Step 2**	**Transition state 2**	-1718.373223	-1717.868378	-1717.962944
	**Molecular complex 4**	-1718.399228	-1717.892725	-1717.986191
	**Molecular complex 5**	-1718.396987	-1717.890105	-1717.985176
**Step 3**	**Transition state 3**	-1718.376576	-1717.874994	-1717.967289
	**Molecular complex 6**	-1718.421597	-1717.915635	-1718.010404

Table S25. Electronic energies (ΔE), Enthalpies (ΔH) and Gibbs free energies (ΔG) obtained for the solvent dichloromethane.

		E	H	G
**Step 1**	**Molecular complex 1**	-1718.372457	-1717.869039	-1717.971432
	**Transition state 1**	-1718.359727	-1717.855812	-1717.951871
	**Molecular complex 2**	-1718.379377	-1717.873334	-1717.969989
**Step 2**	**Molecular complex 3**	-1718.379288	-1717.87324	-1717.971314
	**Transition state 2**	-1718.369762	-1717.864917	-1717.959483
	**Molecular complex 4**	-1718.395361	-1717.888858	-1717.982324
**Step 3**	**Molecular complex 5**	-1718.395145	-1717.888263	-1717.983334
	**Transition state 3**	-1718.377006	-1717.875424	-1717.967719
	**Molecular complex 6**	-1718.425322	-1717.91936	-1718.014129

Table S26. Electronic energies (ΔE), Enthalpies (ΔH) and Gibbs free energies (ΔG) obtained for the solvent toluene.

		E	H	G
**Step 1**	**Molecular complex 1**	-1718.360972	-1717.857554	-1717.959947
	**Transition state 1**	-1718.349153	-1717.845238	-1717.941297
	**Molecular complex 2**	-1718.366823	-1717.86078	-1717.957435
**Step 2**	**Molecular complex 3**	-1718.36654	-1717.860492	-1717.958566
	**Transition state 2**	-1718.357387	-1717.852542	-1717.947108
	**Molecular complex 4**	-1718.380322	-1717.873819	-1717.967285
**Step 3**	**Molecular complex 5**	-1718.380907	-1717.874025	-1717.969096
	**Transition state 3**	-1718.365477	-1717.863895	-1717.95619
	**Molecular complex 6**	-1718.41588	-1717.909918	-1718.004687

7.9. Proposal I: GBB reaction with a participation of methanol in mechanism, forming a tetrahedral intermediate, catalyzed by *p*-toluenesulfonic acid

Table S27. Electronic energies (ΔE), Enthalpies (ΔH) and Gibbs free energies (ΔG) obtained for the solvent methanol.

		E	H	G
	**Molecular complex 1**	-1718.369882	-1717.866464	-1717.968857
**Step 1**	**Transition state 1**	-1718.358756	-1717.854841	-1717.9509
	**Molecular complex 2**	-1718.3798	-1717.873757	-1717.970412
	**Molecular complex 3**	-1834.069105	-1833.504747	-1833.612496
**Step 2**	**Transition state 2**	-1834.059226	-1833.49672	-1833.599565
	**Molecular complex 4**	-1834.084776	-1833.519643	-1833.622927
	**Molecular complex 5**	-1834.124508	-1833.557853	-1833.6594
**Step 3**	**Transition state 3**	-1834.109792	-1833.544922	-1833.642929
	**Molecular complex 6**	-1834.115131	-1833.548846	-1833.644793
	**Molecular complex 7**	-1834.07242	-1833.506849	-1833.606687
**Step 4**	**Transition state 4**	-1834.068749	-1833.506438	-1833.603916
	**Molecular complex 8**	-1834.101992	-1833.535771	-1833.63685
	**Molecular complex 9**	-1718.396987	-1717.890105	-1717.985176
**Step 5**	**Transition state 5**	-1718.376576	-1717.874994	-1717.967289
	**Molecular complex 10**	-1718.421597	-1717.915635	-1718.010404

7.10. Proposal J: GBB reaction, methanol acts as a proton shuttle in the third step, catalyzed by *p*-toluenesulfonic acid

Table S28. Electronic energies (ΔE), Enthalpies (ΔH) and Gibbs free energies (ΔG) obtained for the solvent methanol.

		E	H	G
**Step 1**	**Molecular complex 1**	-1718.369882	-1717.866464	-1717.968857
	**Transition state 1**	-1718.358756	-1717.854841	-1717.9509
	**Molecular complex 2**	-1718.3798	-1717.873757	-1717.970412
**Step 2**	**Molecular complex 3**	-1718.381279	-1717.875231	-1717.973305
	**Transition state 2**	-1718.373223	-1717.868378	-1717.962944
	**Molecular complex 4**	-1718.399228	-1717.892725	-1717.986191
**Step 3**	**Molecular complex 5**	-1834.0788	-1833.571918	-1833.666989
	**Transition state 3**	-1834.020787	-1833.519205	-1833.6115
	**Molecular complex 6**	-1834.113004	-1833.607042	-1833.701811

7.11. Proposal K: Methanol addition to imine, catalyzed by *p*-toluenesulfonic acid

Table S29. Electronic energies (ΔE), Enthalpies (ΔH) and Gibbs free energies (ΔG) obtained for the solvent methanol.

		E	H	G
**Step 1**	**Molecular complex 1**	-1583.483316	-1583.062283	-1583.152323
	**Transition state 1**	-1583.474567	-1583.054232	-1583.140802
	**Molecular complex 2**	-1583.485303	-1583.063066	-1583.150106

8. Electronic energies (ΔE), Enthalpies (ΔH) and Gibbs free energies (ΔG) variation along all pathways

Note: All energies values presented below are expressed in kcal·mol^{-1}. The energies were calculated using M06-2X/6-31++G(d,p)/SMD=methanol (dichloromethane and toluene were used in some cases) //M06-2X/6-31G++(d,p) level of theory.

8.1. Proposal A: Classic GBB reaction

Table S30. Electronic energies (ΔE), Enthalpies (ΔH) and Gibbs free energies (ΔG) obtained for the solvent methanol.

		ΔE	ΔH	ΔG
	**Molecular complex 1**	0.00	0.00	0.00
**Step 1**	**Transition state 1**	22.61	21.83	24.14
	**Molecular complex 2**	19.94	19.89	21.93
	**Molecular complex 3**	0.00	0.00	0.00
**Step 2**	**Transition state 2**	0.26	-0.30	0.65
	**Molecular complex 4**	-30.34	-28.91	-27.05
	**Molecular complex 5**	0.00	0.00	0.00
**Step 3**	**Transition state 3**	63.03	58.83	63.03
	**Molecular complex 6**	-28.63	-28.08	-26.89

Table S31. Electronic energies (ΔE), Enthalpies (ΔH) and Gibbs free energies (ΔG) obtained for the solvent dichloromethane.

		ΔE	ΔH	ΔG
	**Molecular complex 1**	0.00	0.00	0.00
**Step 1**	**Transition state 1**	24.37	23.60	25.91
	**Molecular complex 2**	22.15	22.11	24.14
	**Molecular complex 3**	0.00	0.00	0.00
**Step 2**	**Transition state 2**	0.55	-0.01	0.94
	**Molecular complex 4**	-31.01	-29.57	-27.72
	**Molecular complex 5**	0.00	0.00	0.00
**Step 3**	**Transition state 3**	62.99	58.80	62.99
	**Molecular complex 6**	-28.85	-28.29	-27.10

Table S32. Electronic energies (ΔE), Enthalpies (ΔH) and Gibbs free energies (ΔG) obtained for the solvent toluene.

		ΔE	ΔH	ΔG
**Step 1**	**Molecular complex 1**	0.00	0.00	0.00
	**Transition state 1**	25.18	24.41	26.71
	**Molecular complex 2**	23.71	23.67	25.71
**Step 2**	**Molecular complex 3**	0.00	0.00	0.00
	**Transition state 2**	0.72	0.16	1.11
	**Molecular complex 4**	-32.45	-31.02	-29.17
**Step 3**	**Molecular complex 5**	0.00	0.00	0.00
	**Transition state 3**	63.31	59.11	63.31
	**Molecular complex 6**	-28.89	-28.34	-27.15

8.2. Proposal B: GBB reaction, 2-aminopyridine acts as a proton shuttle in the third step

Table S33. Electronic energies (ΔE), Enthalpies (ΔH) and Gibbs free energies (ΔG) obtained for the solvent methanol.

		ΔE	ΔH	ΔG
**Step 1**	**Molecular complex 1**	0.00	0.00	0.00
	**Transition state 1**	22.61	21.83	24.14
	**Molecular complex 2**	19.94	19.89	21.93
**Step 2**	**Molecular complex 3**	0.00	0.00	0.00
	**Transition state 2**	0.26	-0.30	0.65
	**Molecular complex 4**	-30.34	-28.91	-27.05
**Step 3**	**Molecular complex 5**	0.00	0.00	0.00
	**Transition state 3**	19.61	15.67	17.18
	**Molecular complex 6**	-14.37	-13.66	-13.06

Table S34. Electronic energies (ΔE), Enthalpies (ΔH) and Gibbs free energies (ΔG) obtained for the solvent dichloromethane.

		**ΔE**	**ΔH**	**ΔG**
	**Molecular complex 1**	0.00	0.00	0.00
**Step 1**	**Transition state 1**	24.37	23.60	25.91
	**Molecular complex 2**	22.15	22.11	24.14
	**Molecular complex 3**	0.00	0.00	0.00
**Step 2**	**Transition state 2**	0.55	-0.01	0.94
	**Molecular complex 4**	-31.01	-29.57	-27.72
	**Molecular complex 5**	0.00	0.00	0.00
**Step 3**	**Transition state 3**	17.51	13.57	15.08
	**Molecular complex 6**	-16.38	-15.67	-15.07

Table S35. Electronic energies (ΔE), Enthalpies (ΔH) and Gibbs free energies (ΔG) obtained for the solvent toluene.

		**ΔE**	**ΔH**	**ΔG**
	**Molecular complex 1**	0.00	0.00	0.00
**Step 1**	**Transition state 1**	25.18	24.41	26.71
	**Molecular complex 2**	23.71	23.67	25.71
	**Molecular complex 3**	0.00	0.00	0.00
**Step 2**	**Transition state 2**	0.72	0.16	1.11
	**Molecular complex 4**	-32.45	-31.02	-29.17
	**Molecular complex 5**	0.00	0.00	0.00
**Step 3**	**Transition state 3**	16.31	12.38	13.89
	**Molecular complex 6**	-18.11	-17.40	-16.81

8.3. Proposal C: GBB reaction, methanol addition to nitrilium in the second step, protonating the amine

Table S36. Electronic energies (ΔE), Enthalpies (ΔH) and Gibbs free energies (ΔG) obtained for the solvent methanol.

		**ΔE**	**ΔH**	**ΔG**
**Step 1**	**Molecular complex 1**	0.00	0.00	0.00
	**Transition state 1**	22.61	21.83	24.14
	**Molecular complex 2**	19.94	19.89	21.93
**Step 2***	**Molecular complex 3**	0.00	0.00	0.00
	**Transition state 2**	12.70	10.00	10.61
	**Molecular complex 4**	-41.36	-39.64	-35.98

*For the transition state of this step, the solvent methanol participated in the mechanism, and a correction of 1.90 $kcal \cdot mol^{-1}$ was applied for ΔG.

7.4. Proposal D: GBB reaction, methanol addition to nitrilium in the second step, protonating the pyridine

Table S37. Electronic energies (ΔE), Enthalpies (ΔH) and Gibbs free energies (ΔG) obtained for the solvent methanol.

		**ΔE**	**ΔH**	**ΔG**
**Step 1**	**Molecular complex 1**	0.00	0.00	0.00
	**Transition state 1**	22.61	21.83	24.14
	**Molecular complex 2**	19.94	19.89	21.93
**Step 2***	**Molecular complex 3**	0.00	0.00	0.00
	**Transition state 2**	12.86	10.62	10.70
	**Molecular complex 4**	-21.37	-19.86	-19.07

*For the transition state of this step, the solvent methanol participated in the mechanism, and a correction of 1.90 $kcal \cdot mol^{-1}$ was applied for ΔG.

8.5. Proposal E: GBB reaction, methanol acts as a hydrogen bond donor in the first and second steps, and as a proton shuttle in the third step

Table S38. Electronic energies (ΔE), Enthalpies (ΔH) and Gibbs free energies (ΔG) obtained for the solvent methanol.

		**ΔE**	**ΔH**	**ΔG**
**Step 1***	**Molecular complex 1**	0.00	0.00	0.00
	**Transition state 1**	19.95	19.20	20.74
	**Molecular complex 2**	17.20	17.11	18.92
**Step 2***	**Molecular complex 3**	0.00	0.00	0.00
	**Transition state 2**	2.86	2.31	-0.54
	**Molecular complex 4**	-27.83	-26.48	-24.19
**Step 3***	**Molecular complex 5**	0.00	0.00	0.00
	**Transition state 3**	33.69	28.79	28.90
	**Molecular complex 6**	-24.70	-24.20	-23.33

*For the transition state of this step, the solvent methanol participated in the mechanism, and a correction of 1.90 kcal·mol^{-1} was applied for ΔG.

8.6. Proposal F: GBB reaction, methanol acts as a proton shuttle in the third step

Table S39. Electronic energies (ΔE), Enthalpies (ΔH) and Gibbs free energies (ΔG) obtained for the solvent methanol.

		**ΔE**	**ΔH**	**ΔG**
**Step 1**	**Molecular complex 1**	0.00	0.00	0.00
	**Transition state 1**	22.61	21.83	24.14
	**Molecular complex 2**	19.94	19.89	21.93
**Step 2**	**Molecular complex 3**	0.00	0.00	0.00
	**Transition state 2**	0.26	-0.30	0.65
	**Molecular complex 4**	-30.34	-28.91	-27.05
**Step 3***	**Molecular complex 5**	0.00	0.00	0.00
	**Transition state 3**	33.69	28.79	28.90
	**Molecular complex 6**	-24.70	-24.20	-23.33

*For the transition state of this step, the solvent methanol participated in the mechanism, and a correction of 1.90 kcal·mol^{-1} was applied for ΔG.

8.7. Proposal G: GBB reaction, methanol acts as a hydrogen bond donor in the first and second steps, and 2-aminopyridine acts a proton shuttle in the third step

Table S40. Electronic energies (ΔE), Enthalpies (ΔH) and Gibbs free energies (ΔG) obtained for the solvent methanol.

		ΔE	ΔH	ΔG
**Step 1***	**Molecular complex 1**	0.00	0.00	0.00
	**Transition state 1**	19.95	19.20	20.74
	**Molecular complex 2**	17.20	17.11	18.92
**Step 2***	**Molecular complex 3**	0.00	0.00	0.00
	**Transition state 2**	2.86	2.31	-0.54
	**Molecular complex 4**	-27.83	-26.48	-24.19
**Step 3**	**Molecular complex 5**	0,00	0,00	0,00
	**Transition state 3**	19,61	15,67	17,18
	**Molecular complex 6**	-14,37	-13,66	-13,06

*For the transition state of this step, the solvent methanol participated in the mechanism, and a correction of 1.90 kcal·mol^{-1} was applied for ΔG.

8.8. Proposal H: GBB reaction catalyzed by *p*-toluenesulfonic acid

Table S41. Electronic energies (ΔE), Enthalpies (ΔH) and Gibbs free energies (ΔG) obtained for the solvent methanol.

		ΔE	ΔH	ΔG
**Step 1**	**Molecular complex 1**	0.00	0.00	0.00
	**Transition state 1**	6.98	7.29	11.27
	**Molecular complex 2**	-6.22	-4.58	-0.98
**Step 2**	**Molecular complex 3**	0.00	0.00	0.00
	**Transition state 2**	5.06	4.30	6.50
	**Molecular complex 4**	-11.26	-10.98	-8.09
**Step 3**	**Molecular complex 5**	0.00	0.00	0.00
	**Transition state 3**	12.81	9.48	11.22
	**Molecular complex 6**	-15.44	-16.02	-15.83

Table S42. Electronic energies (ΔE), Enthalpies (ΔH) and Gibbs free energies (ΔG) obtained for the solvent dichloromethane.

		**ΔE**	**ΔH**	**ΔG**
	**Molecular complex 1**	0.00	0.00	0.00
**Step 1**	**Transition state 1**	7.99	8.30	12.28
	**Molecular complex 2**	-4.34	-2.69	0.91
	**Molecular complex 3**	0.00	0.00	0.00
**Step 2**	**Transition state 2**	5.98	5.22	7.42
	**Molecular complex 4**	-10.09	-9.80	-6.91
	**Molecular complex 5**	0.00	0.00	0.00
**Step 3**	**Transition state 3**	11.38	8.06	9.80
	**Molecular complex 6**	-18.94	-19.51	-19.32

Table S43. Electronic energies (ΔE), Enthalpies (ΔH) and Gibbs free energies (ΔG) obtained for the solvent toluene.

		**ΔE**	**ΔH**	**ΔG**
	**Molecular complex 1**	0.00	0.00	0.00
**Step 1**	**Transition state 1**	7.42	7.73	11.70
	**Molecular complex 2**	-3.67	-2.02	1.58
	**Molecular complex 3**	0.00	0.00	0.00
**Step 2**	**Transition state 2**	5.74	4.99	7.19
	**Molecular complex 4**	-8.65	-8.36	-5.47
	**Molecular complex 5**	0.00	0.00	0.00
**Step 3**	**Transition state 3**	9.68	6.36	8.10
	**Molecular complex 6**	-21.95	-22.52	-22.33

8.9. Proposal I: GBB reaction with a participation of methanol in mechanism, forming a tetrahedral intermediate, catalyzed by *p*-toluenesulfonic acid

Table S44. Electronic energies (ΔE), Enthalpies (ΔH) and Gibbs free energies (ΔG) obtained for the solvent methanol.

		**ΔE**	**ΔH**	**ΔG**
**Step 1**	**Molecular complex 1**	0.00	0.00	0.00
	**Transition state 1**	6.98	7.29	11.27
	**Molecular complex 2**	-6.22	-4.58	-0.98
**Step 2***	**Molecular complex 3**	0.00	0.00	0.00
	**Transition state 2**	6.20	5.04	6.21
	**Molecular complex 4**	-9.83	-9.35	-6.55
**Step 3**	**Molecular complex 5**	0.00	0.00	0.00
	**Transition state 3**	9.23	8.11	8.44
	**Molecular complex 6**	5.88	5.65	9.17
**Step 4***	**Molecular complex 7**	0.00	0.00	0.00
	**Transition state 4**	2.30	0.26	-0.16
	**Molecular complex 8**	-18.56	-18.15	-18.93
**Step 5**	**Molecular complex 9**	0.00	0.00	0.00
	**Transition state 5**	12.81	9.48	11.22
	**Molecular complex 10**	-15.44	-16.02	-15.83

*For the transition state of this step, the solvent methanol participated in the mechanism, and a correction of 1.90 kcal·mol^{-1} was applied for ΔG.

8.10. Proposal J: GBB reaction, methanol acts as a proton shuttle in the third step, catalyzed by *p*-toluenesulfonic acid

Table S45. Electronic energies (ΔE), Enthalpies (ΔH) and Gibbs free energies (ΔG) obtained for the solvent methanol.

		ΔE	ΔH	ΔG
**Step 1**	**Molecular complex 1**	0.00	0.00	0.00
	**Transition state 1**	6.98	7.29	11.27
	**Molecular complex 2**	-6.22	-4.58	-0.98
**Step 2**	**Molecular complex 3**	0.00	0.00	0.00
	**Transition state 2**	5.06	4.30	6.50
	**Molecular complex 4**	-11.26	-10.98	-8.09
**Step 3***	**Molecular complex 5**	0.00	0.00	0.00
	**Transition state 3**	36.40	33.08	32.92
	**Molecular complex 6**	-21.46	-22.04	-21.85

*For the transition state of this step, the solvent methanol participated in the mechanism, and a correction of 1.90 kcal·mol^{-1} was applied for ΔG.

8.11. Proposal I: Methanol addition to imine, catalyzed by *p*-toluenesulfonic acid

Table S46. Electronic energies (ΔE), Enthalpies (ΔH) and Gibbs free energies (ΔG) obtained for the solvent methanol.

		ΔE	ΔH	ΔG
**Step 1***	**Molecular complex 1**	0.00	0.00	0.00
	**Transition state 1**	5.49	5.05	5.33
	**Molecular complex 2**	-1.25	-0.49	1.39

*For the transition state of this step, the solvent methanol participated in the mechanism, and a correction of 1.90 kcal·mol^{-1} was applied for ΔG.

9. Images of all optimized structures and selected bond lengths

9.1. Proposal A: Classic GBB reaction

9.1.1. Step 1 - Isocyanide nucleophilic attack, forming nitrilium

Figure S78. Molecular complex 1.

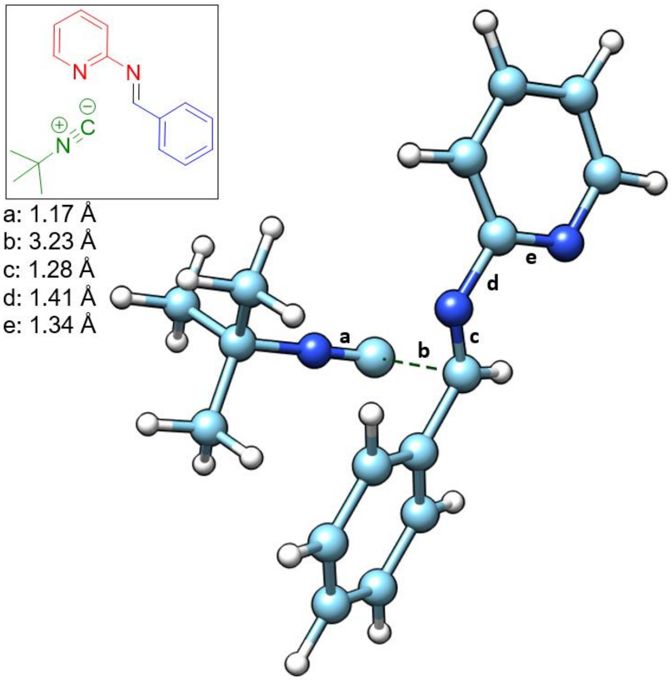

Figure S79. Transition State 1.

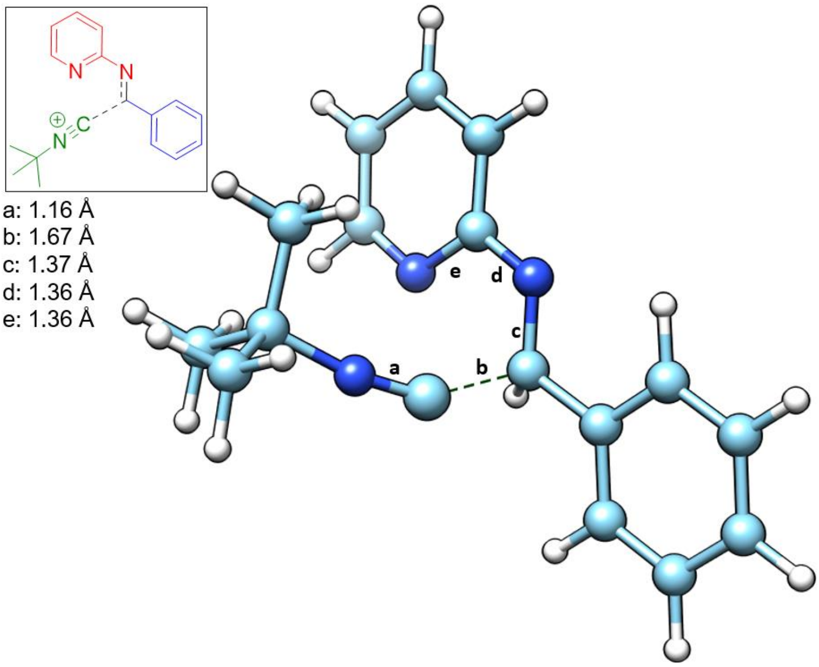

Figure S80. Molecular complex 2.

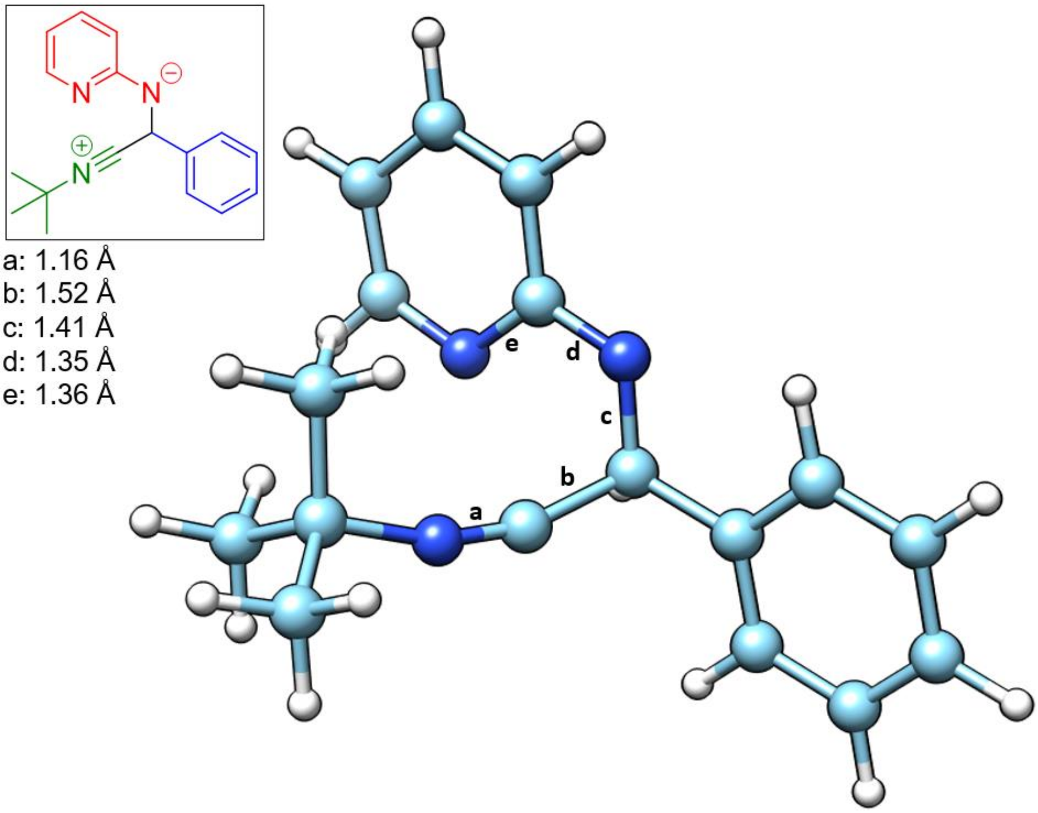

9.1.2. Step 2 – Ring closure

Figure S81. Molecular complex 3.

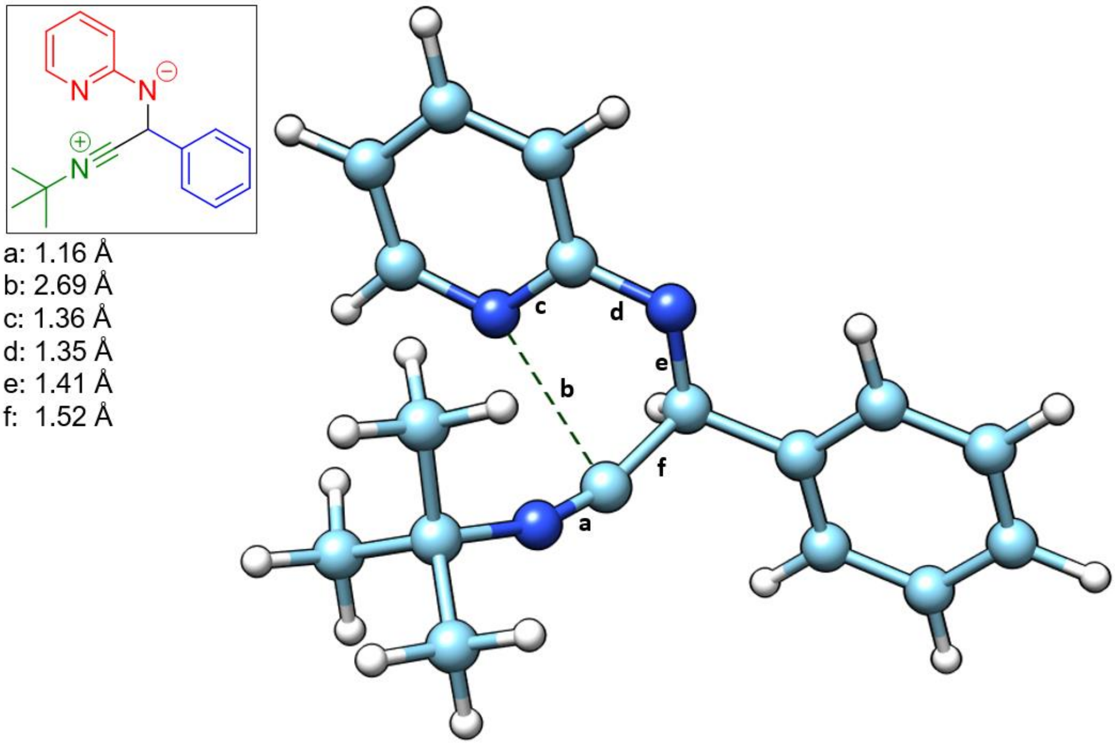

Figure S82. Transition State 2.

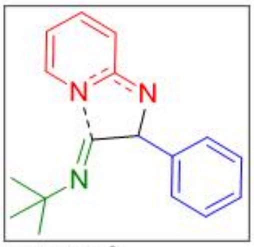

a: 1.16 Å
b: 2.34 Å
c: 1.37 Å
d: 1.34 Å
e: 1.43 Å
f: 1.51 Å

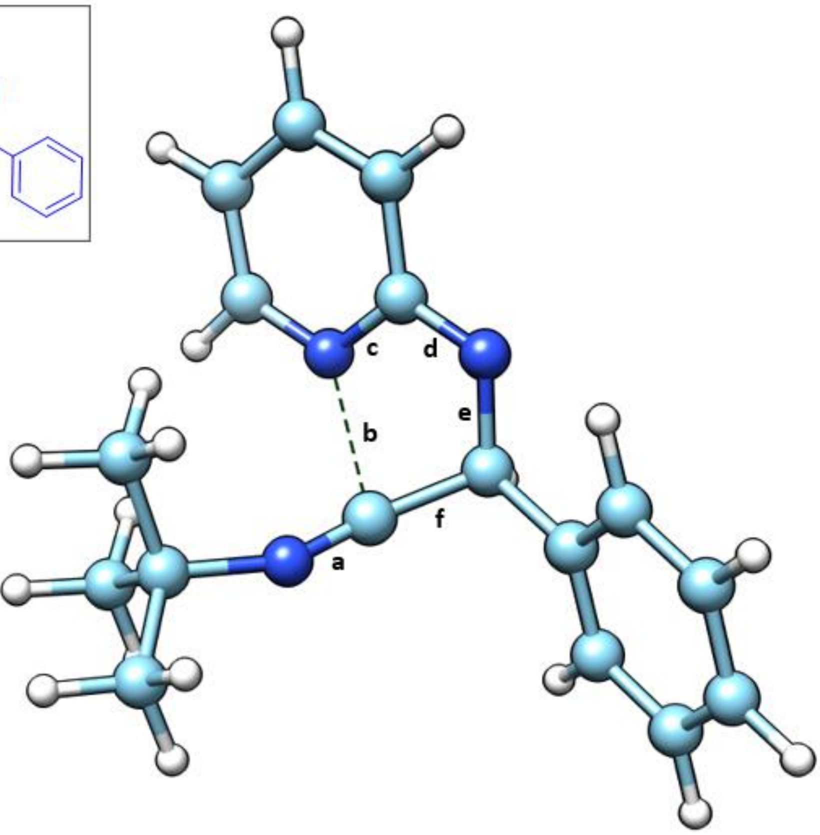

Figure S83. Molecular complex 4.

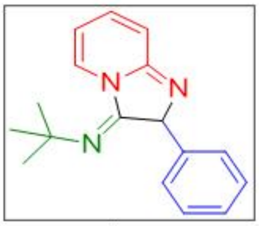

a: 1.26 Å
b: 1.43 Å
c: 1.42 Å
d: 1.28 Å
e: 1.45 Å
f: 1.54 Å

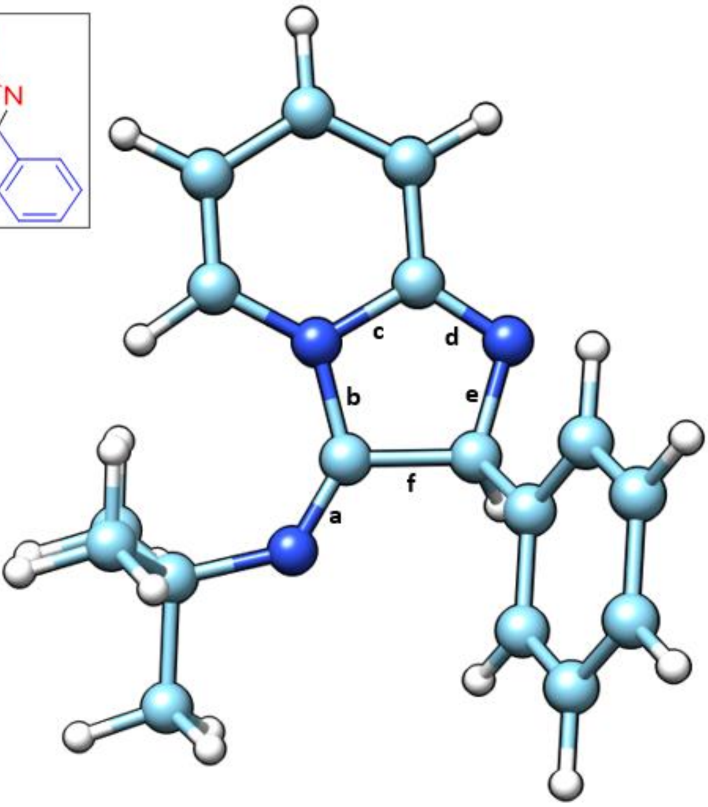

9.1.3. Step 3 – 1,3-Intramolecular hydrogen shift

Figure S84. Molecular complex 5.

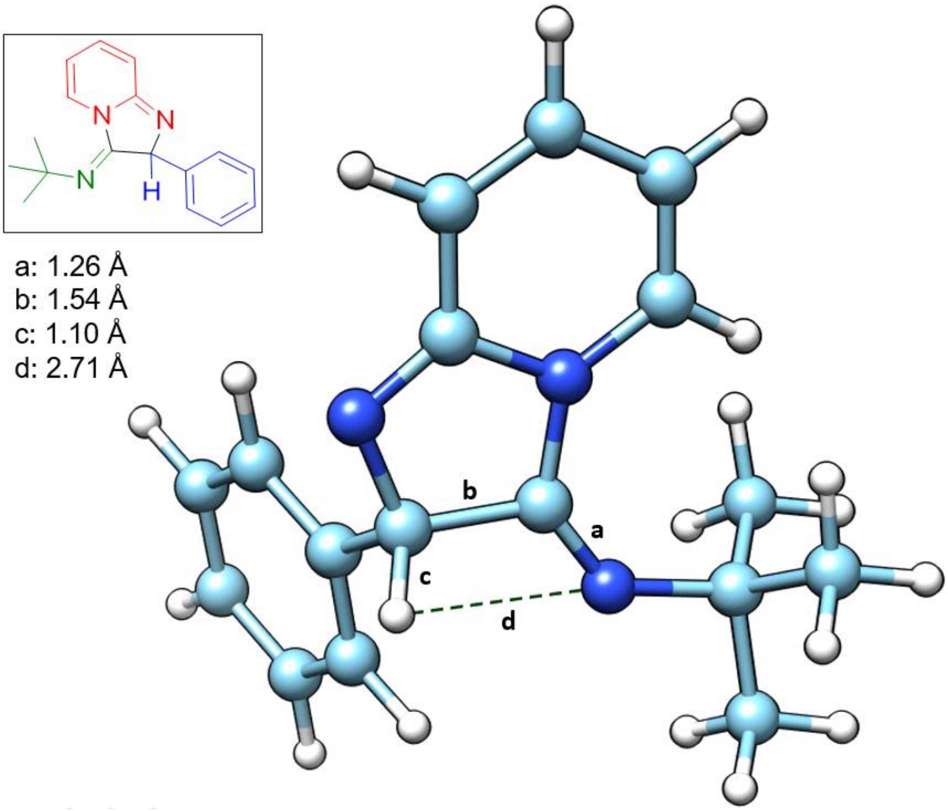

Figure S85. Transition State 3.

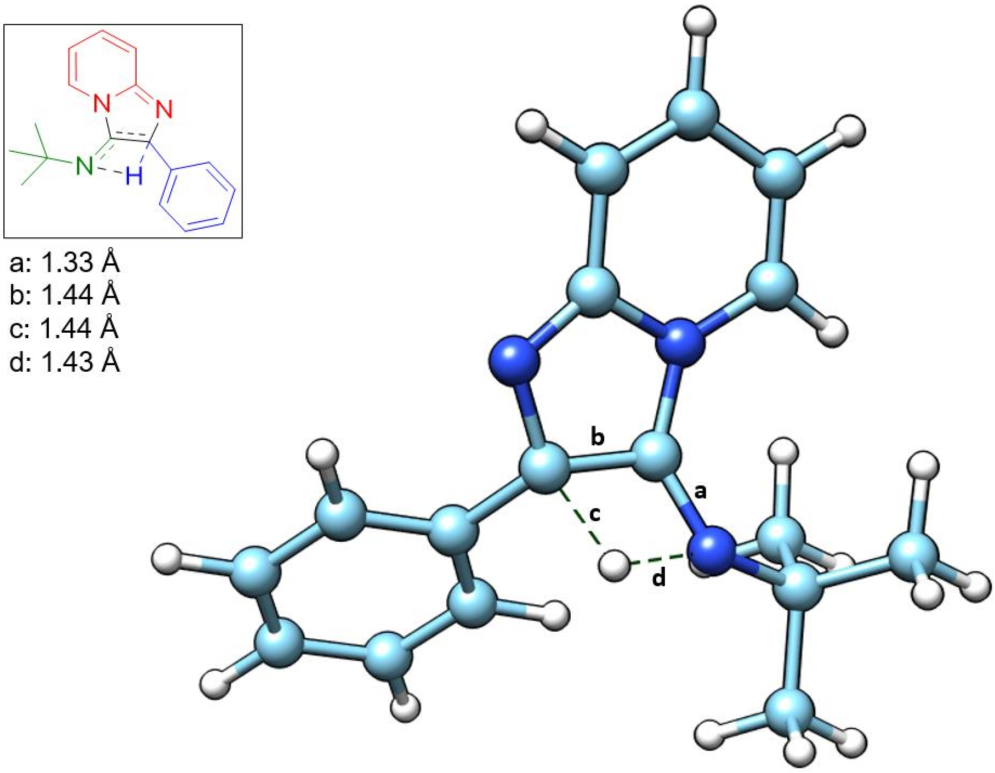

Figure S86. Molecular complex 6.

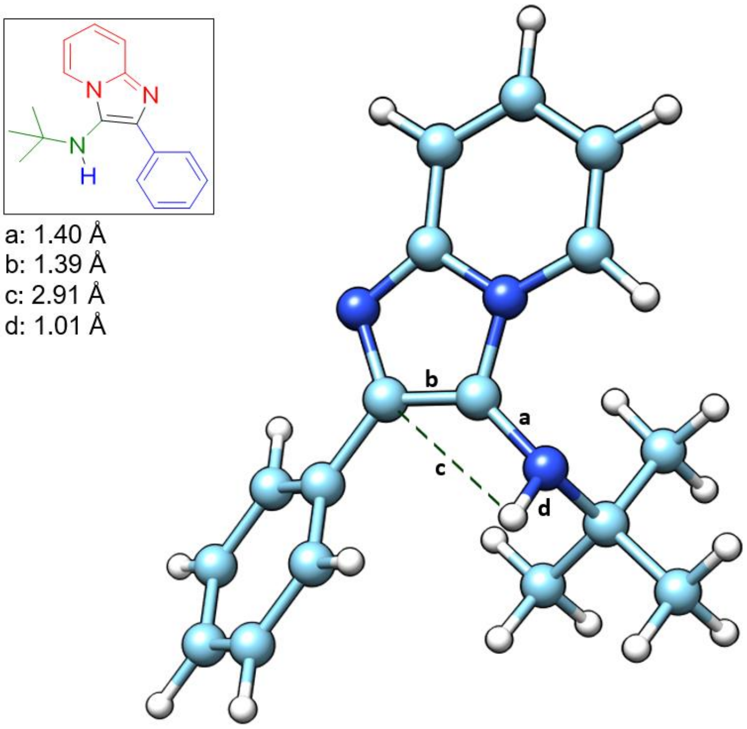

9.2. Proposal B: GBB reaction, 2-aminopyridine acts as a proton shuttle in the third step

9.2.1. Step 1 - Isocyanide nucleophilic attack, forming nitrilium

Figure S87. Molecular complex 1.

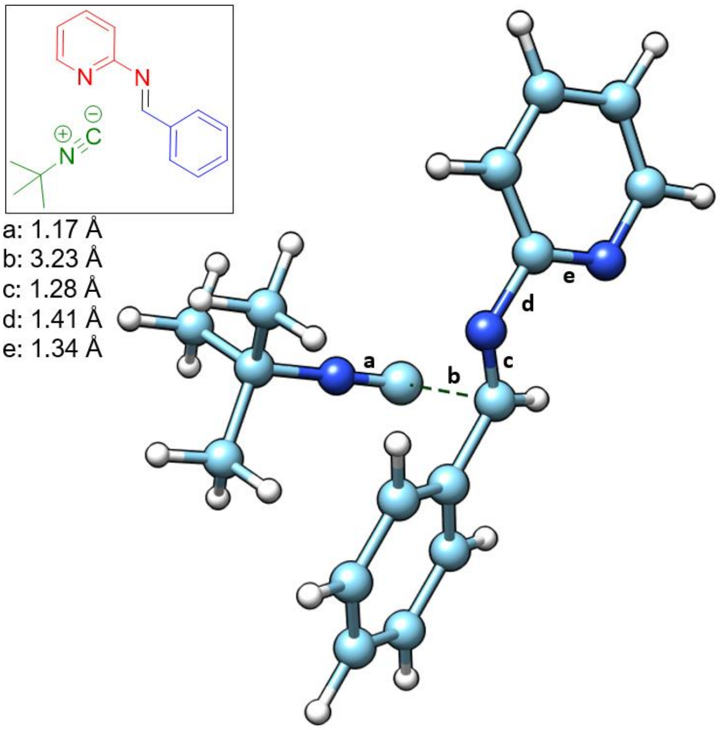

Figure S88. Transition State 1.

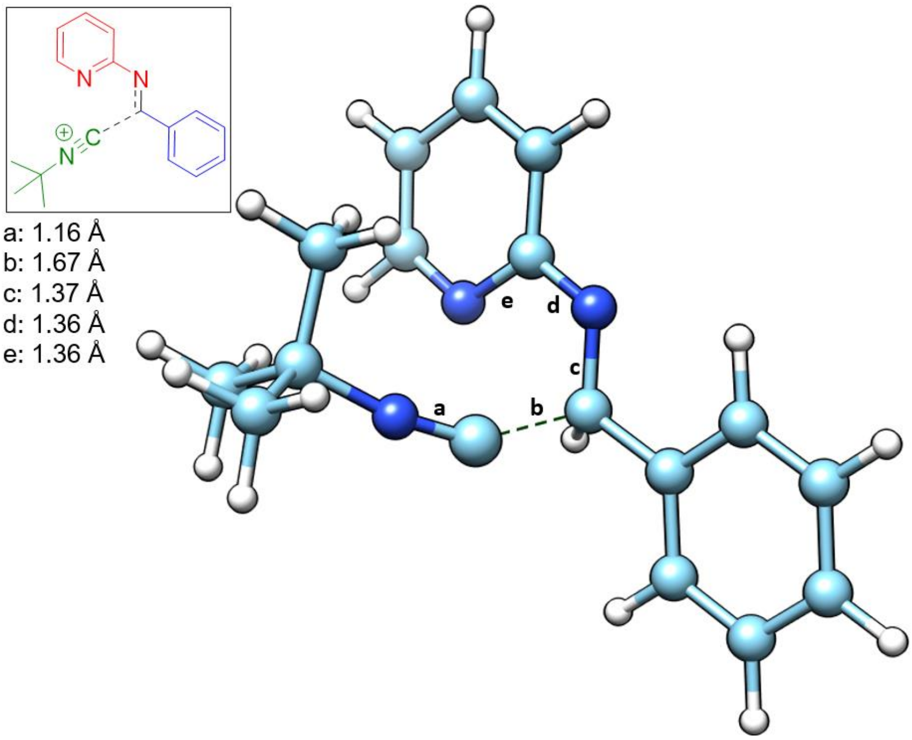

Figure S89. Molecular complex 2.

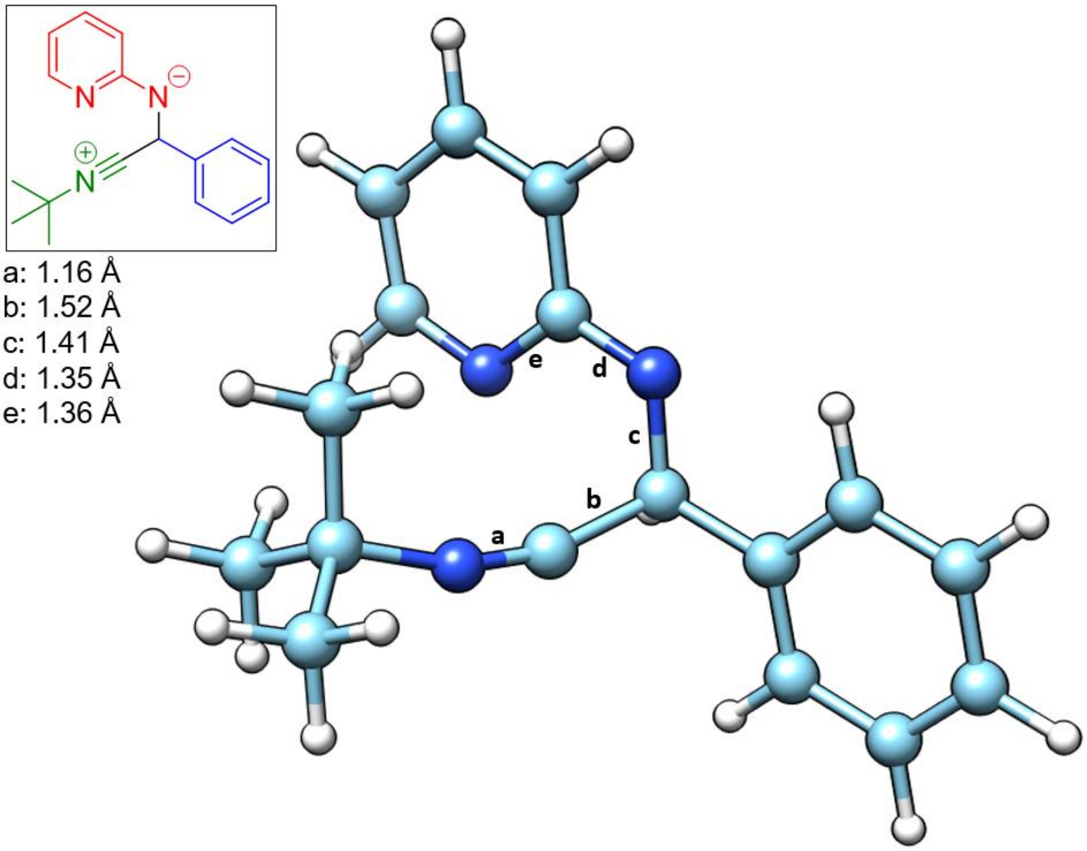

9.2.2. Step 2 – Ring closure

Figure S90. Molecular complex 3.

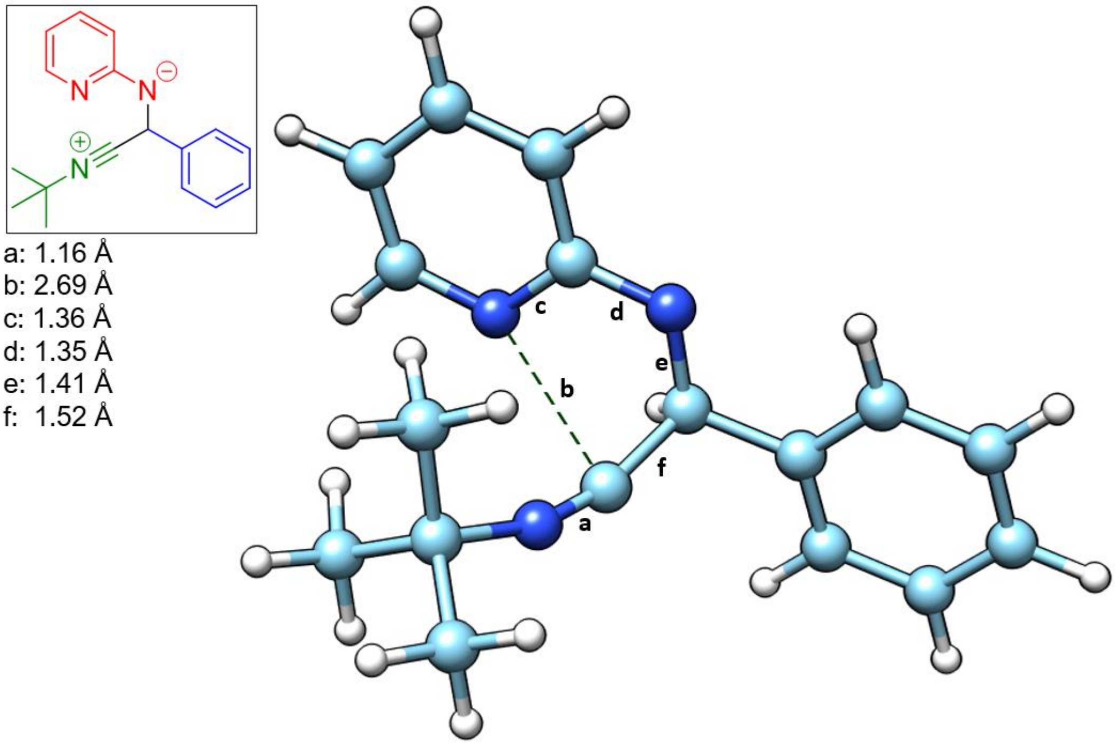

Figure S91. Transition State 2.

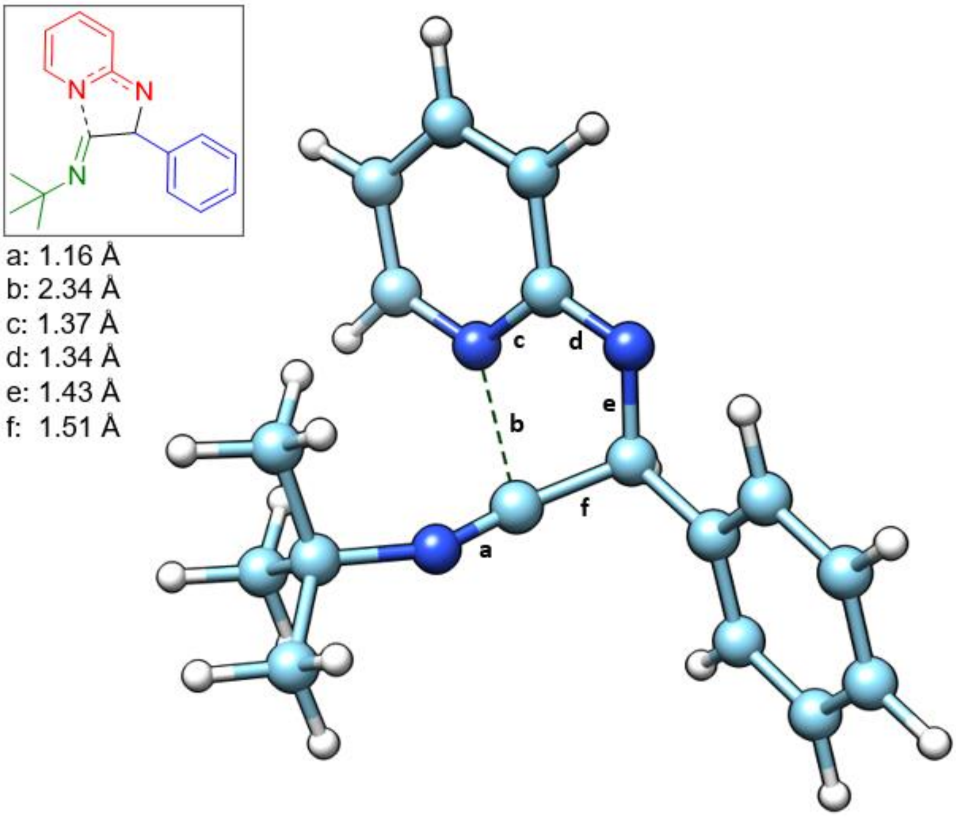

Figure S92. Molecular complex 4.

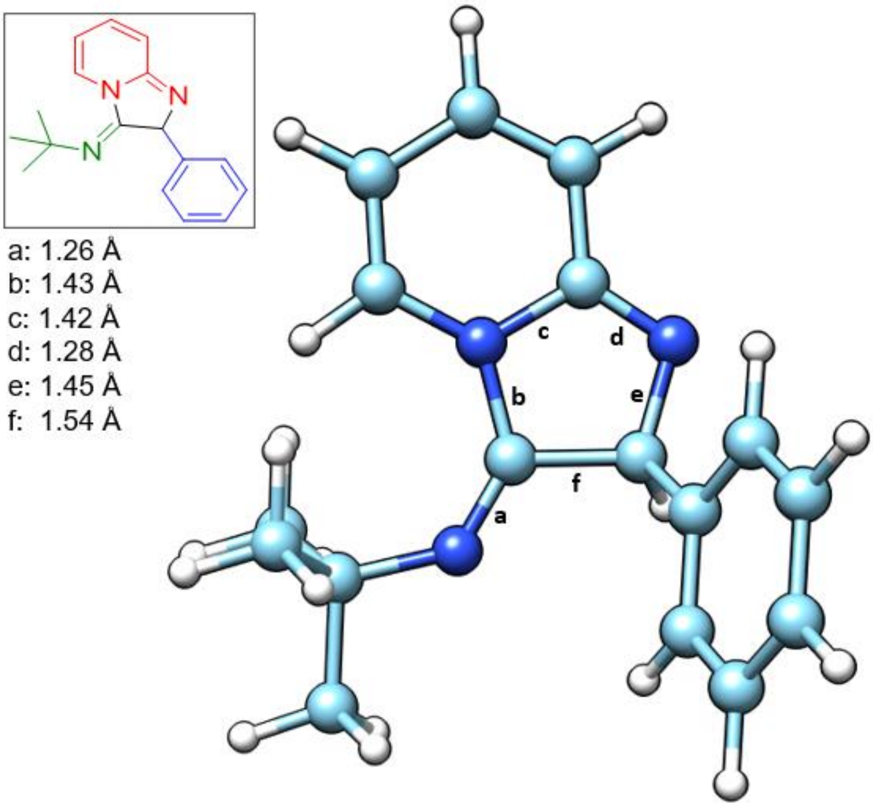

9.2.3. Step 3 – 2-aminopyridine acting as a proton shuttle

Figure S93. Molecular complex 5.

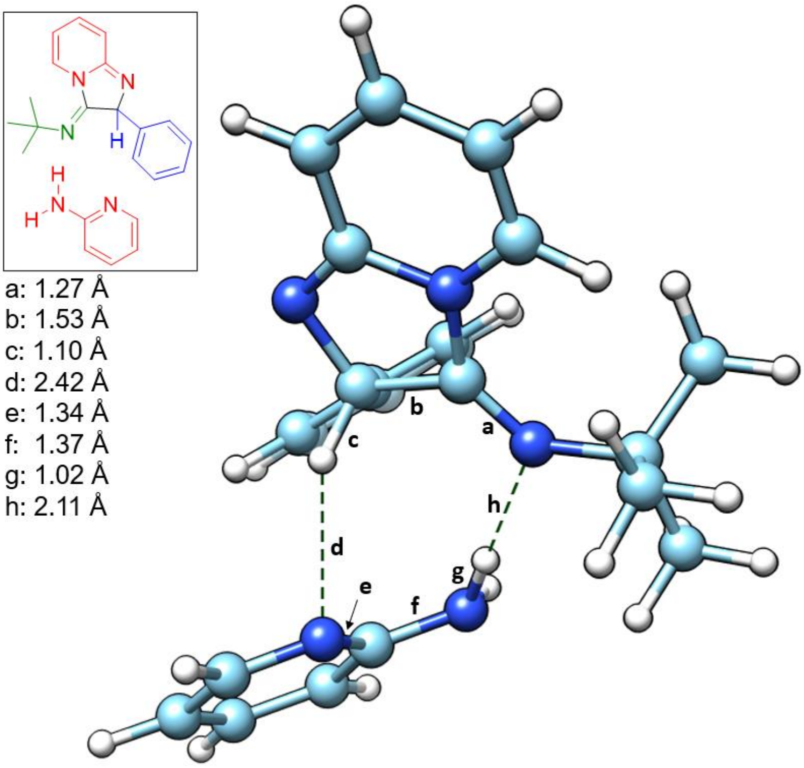

Figure S94. Transition State 3.

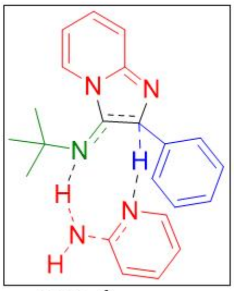

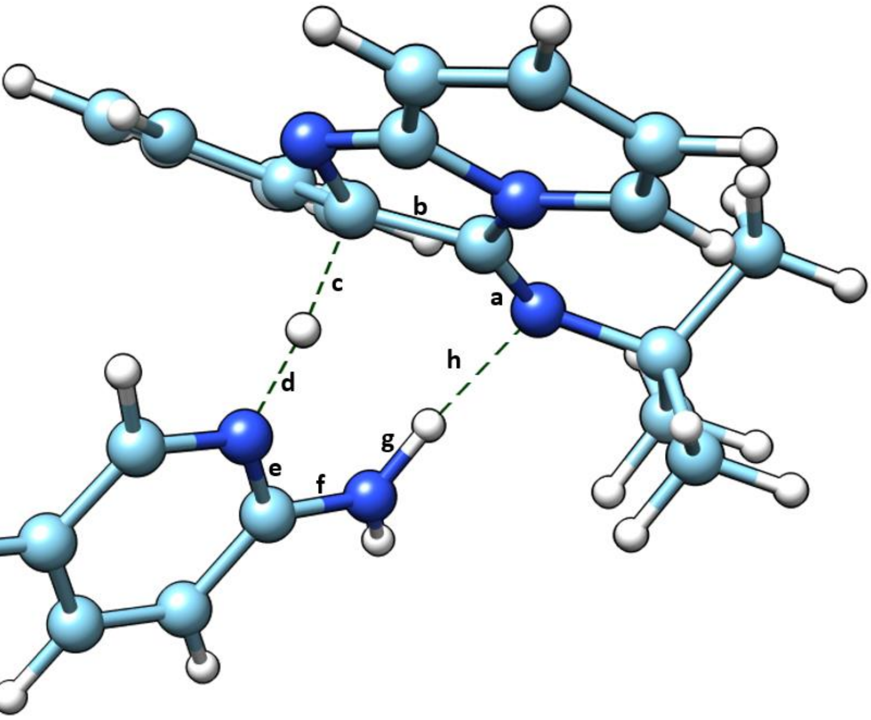

a: 1.30 Å
b: 1.46 Å
c: 1.40 Å
d: 1.29 Å
e: 1.35 Å
f: 1.35 Å
g: 1.04 Å
h: 1.84 Å

Figure S95. Molecular complex 6.

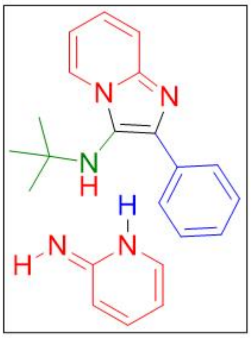

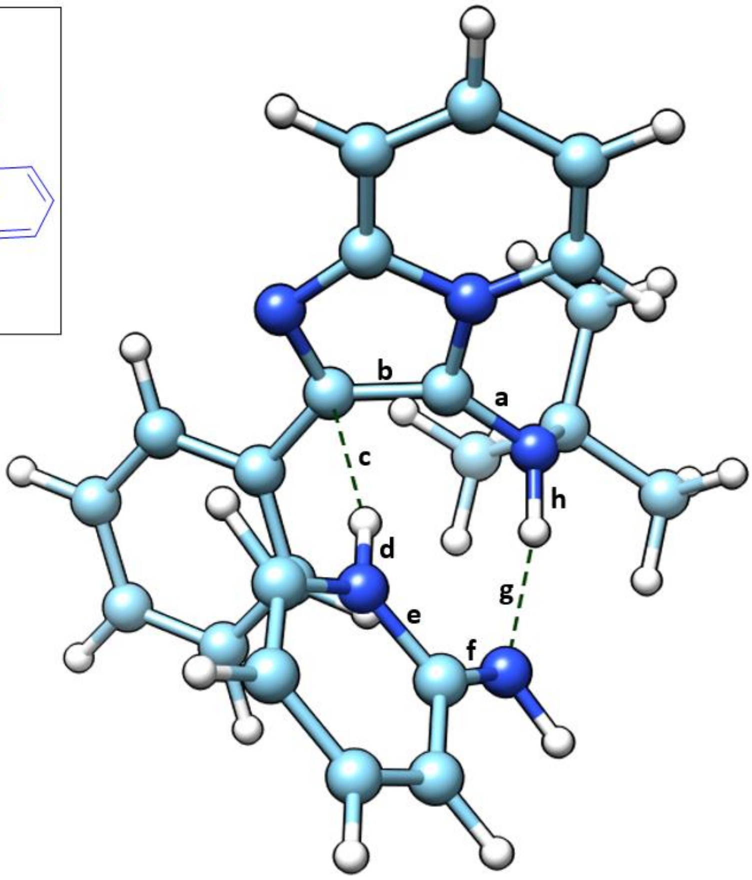

a: 1.39 Å
b: 1.40 Å
c: 2.27 Å
d: 1.02 Å
e: 1.39 Å
f: 1.30 Å
g: 2.13 Å
h: 1.02 Å

9.3. Proposal C: GBB reaction, methanol addition to nitrilium in the second step, protonating the amine

9.3.1. Step 1 - Isocyanide nucleophilic attack, forming nitrilium

Figure S96. Molecular complex 1.

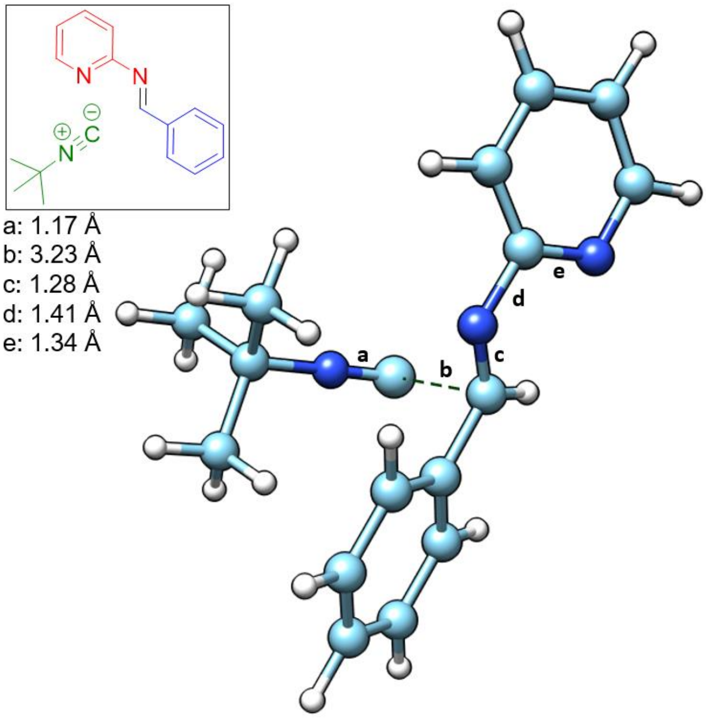

Figure S97. Transition State 1.

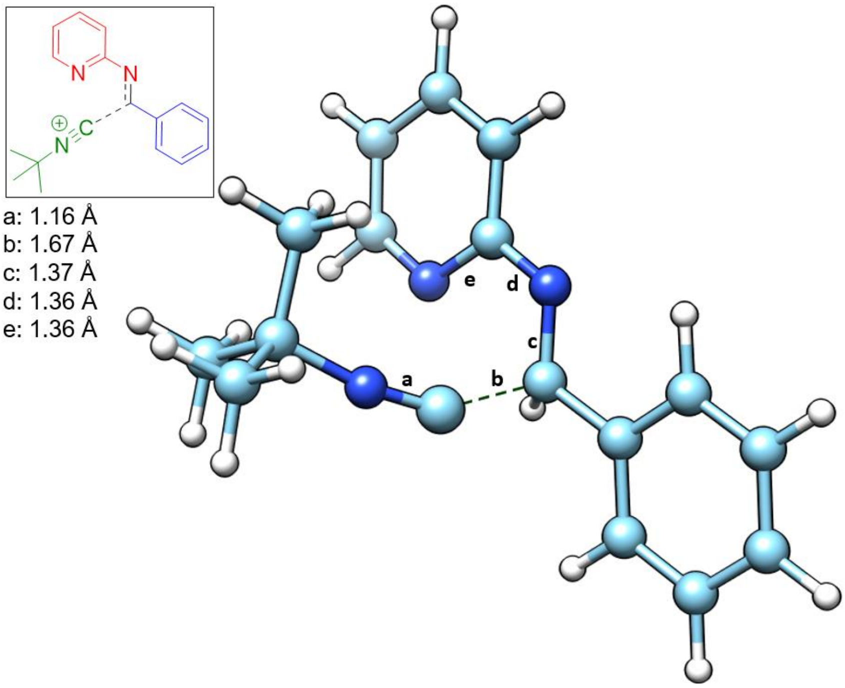

Figure S98. Molecular complex 2.

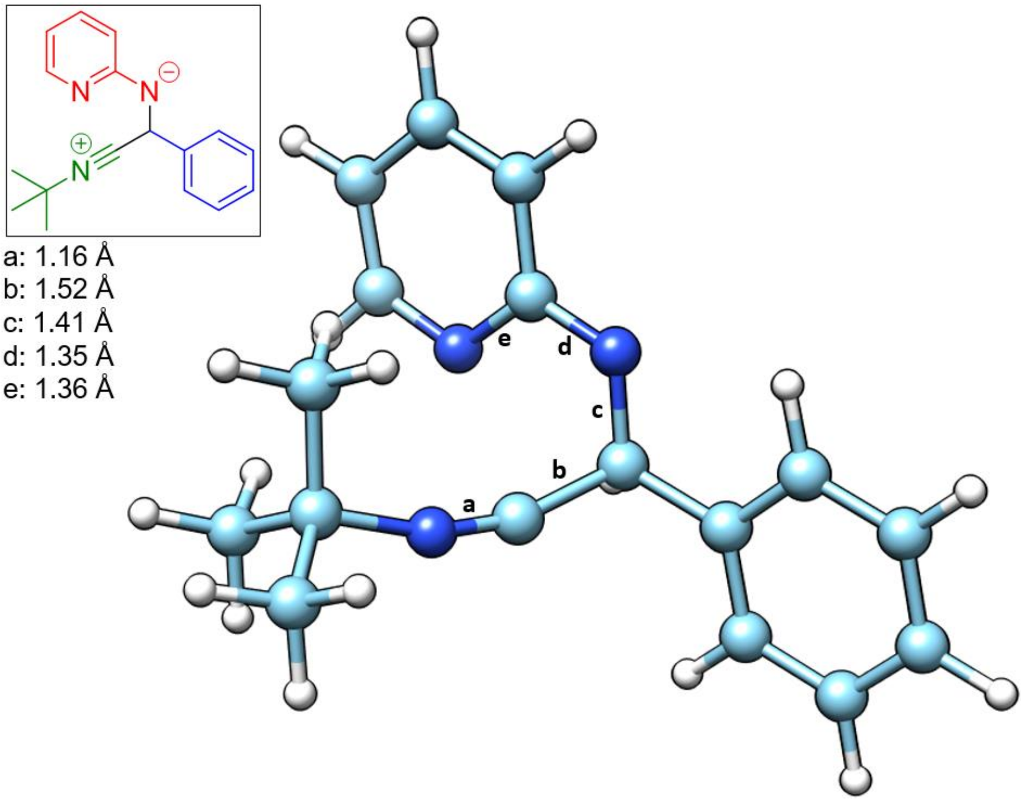

9.3.2. Step 2 – Methanol addition to nitrilium, protonating the amine fragment

Figure S99. Molecular complex 3.

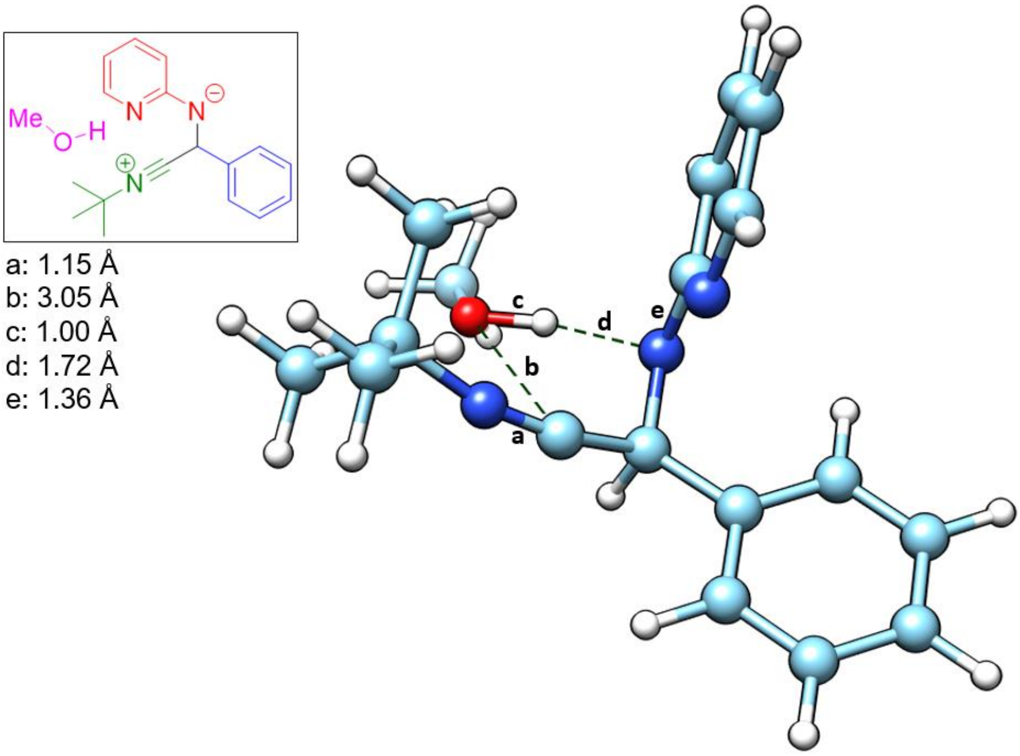

Figure S100. Transition State 2.

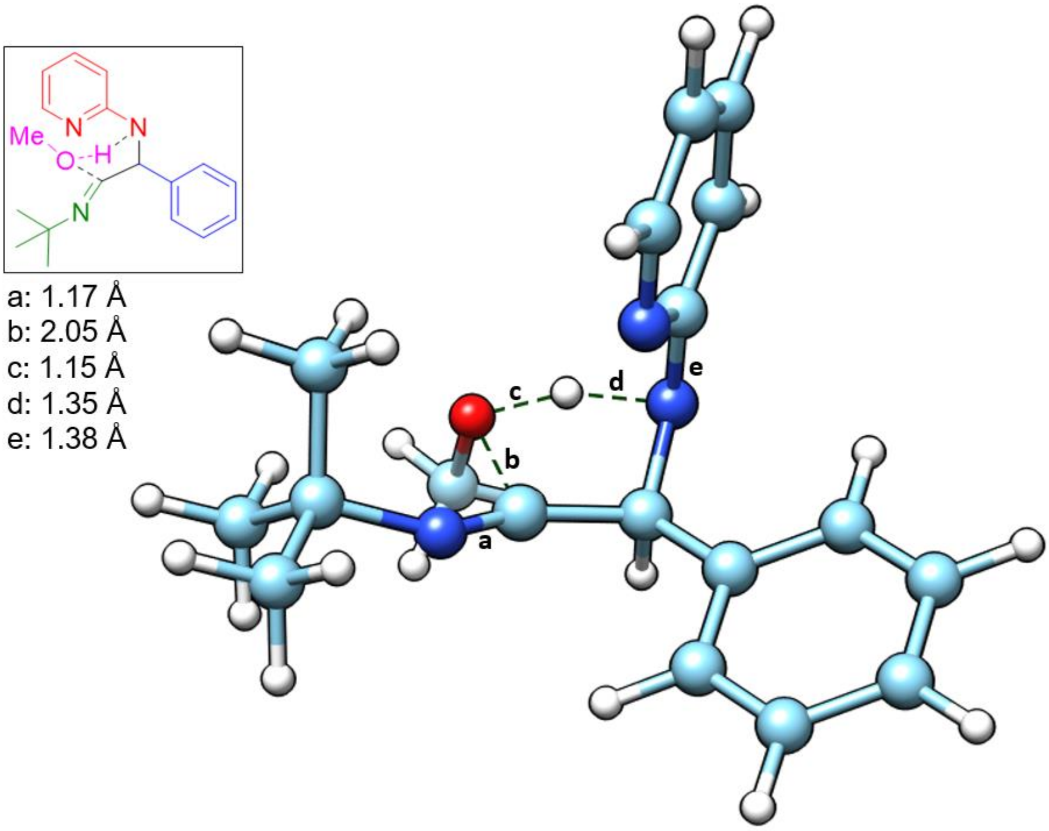

Figure S101. Molecular complex 4.

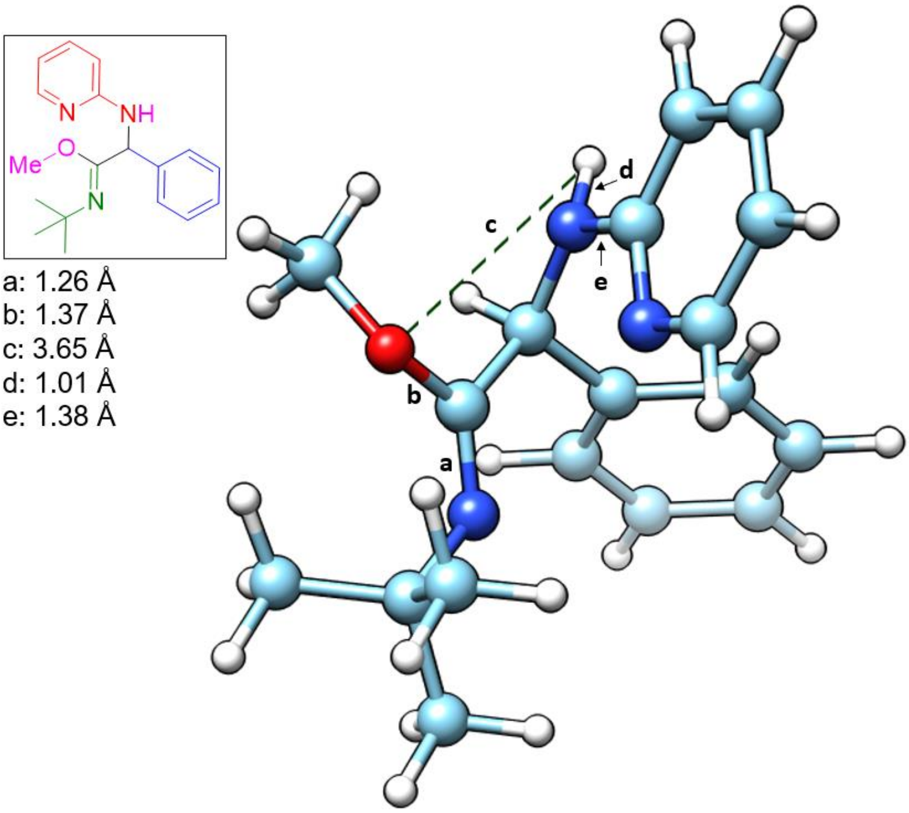

9.4. Proposal D: GBB reaction, methanol addition to nitrilium in the second step, protonating the pyridine

9.4.1. Step 1 - Isocyanide nucleophilic attack, forming nitrilium

Figure S102. Molecular complex 1.

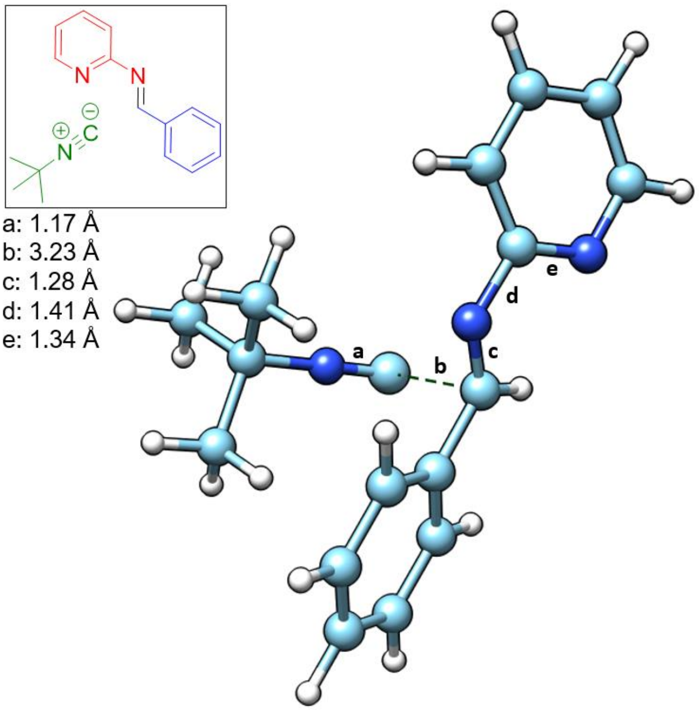

Figure S103. Transition State 1.

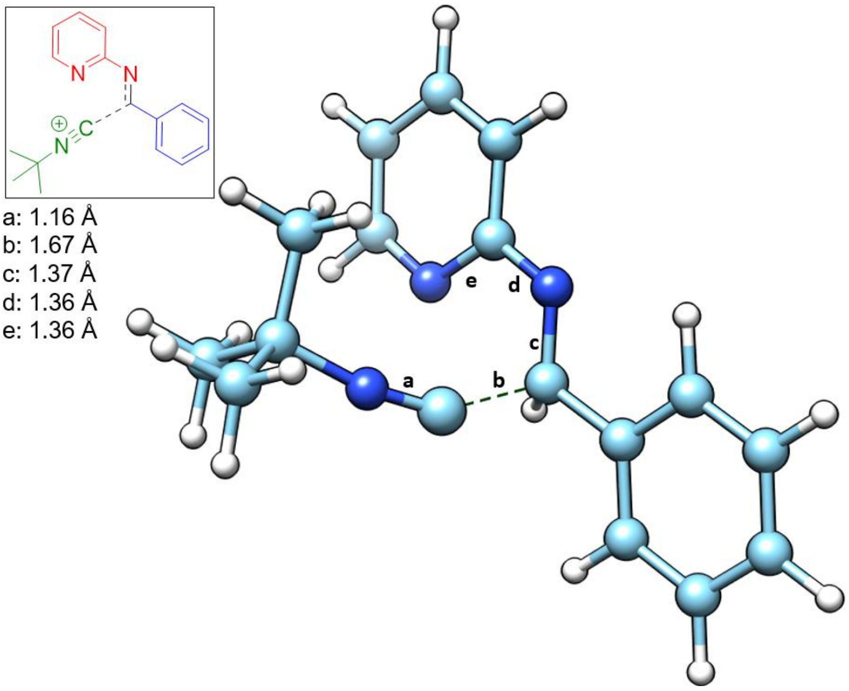

Figure S104. Molecular complex 2.

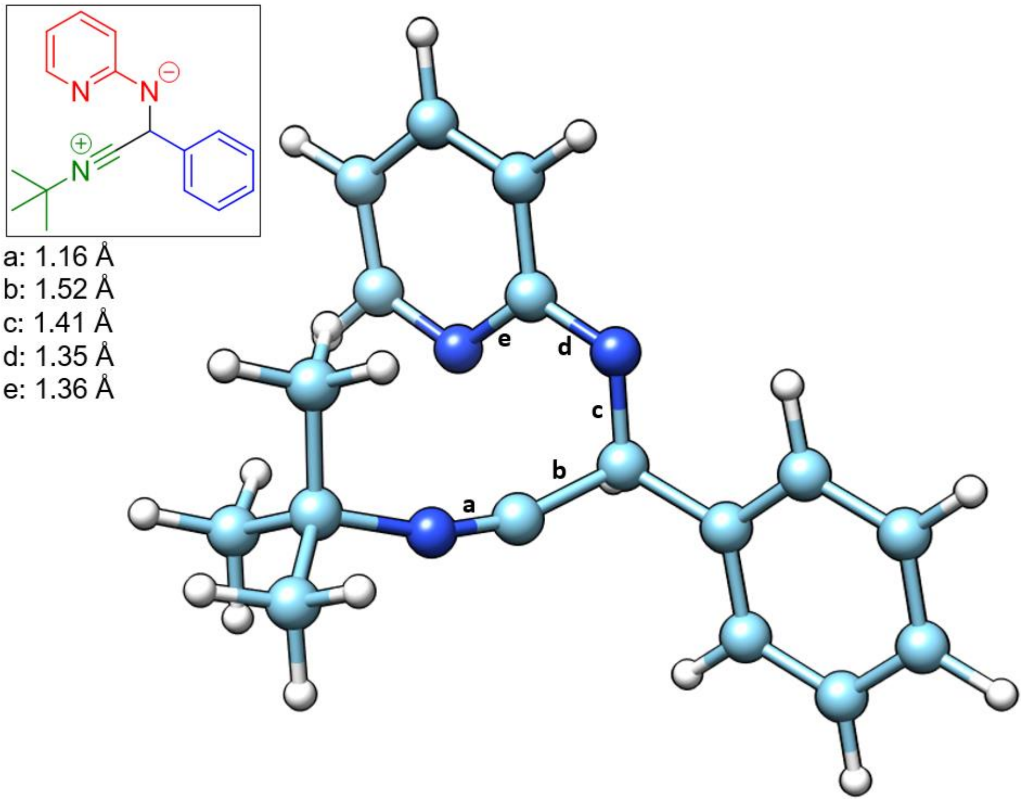

9.4.2. Step 2 – Methanol addition to nitrilium, protonating the pyridine fragment

Figure S105. Molecular complex 3.

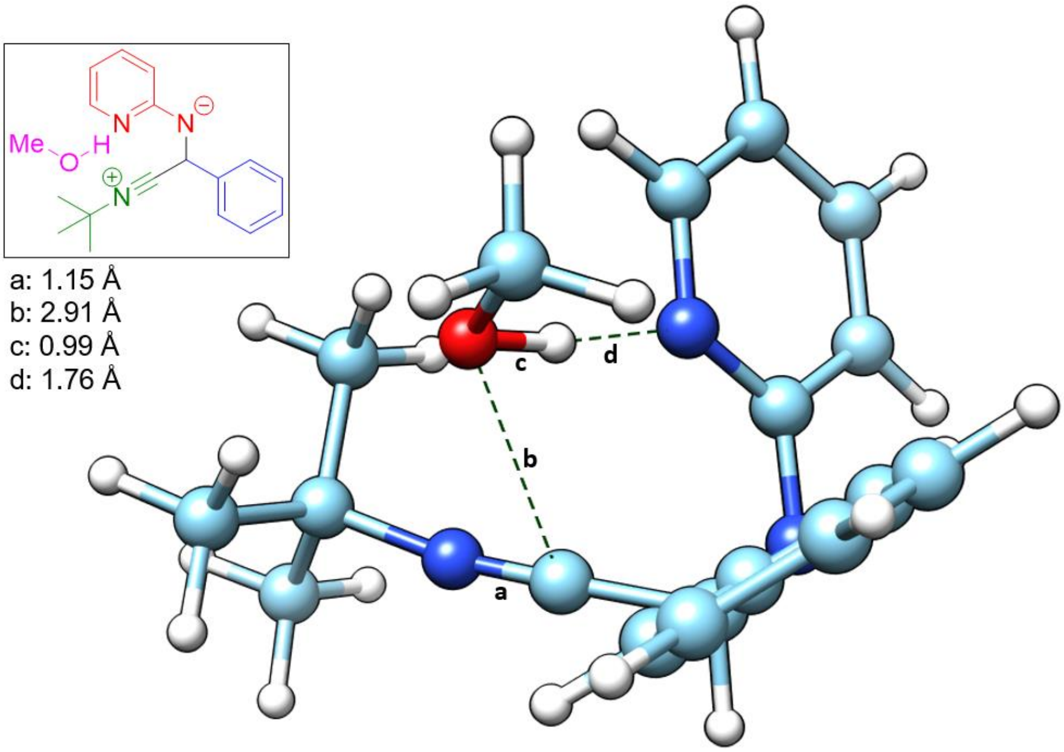

Figure S106. Transition State 2.

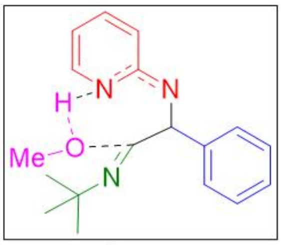

a: 1.18 Å
b: 2.04 Å
c: 1.10 Å
d: 1.40 Å

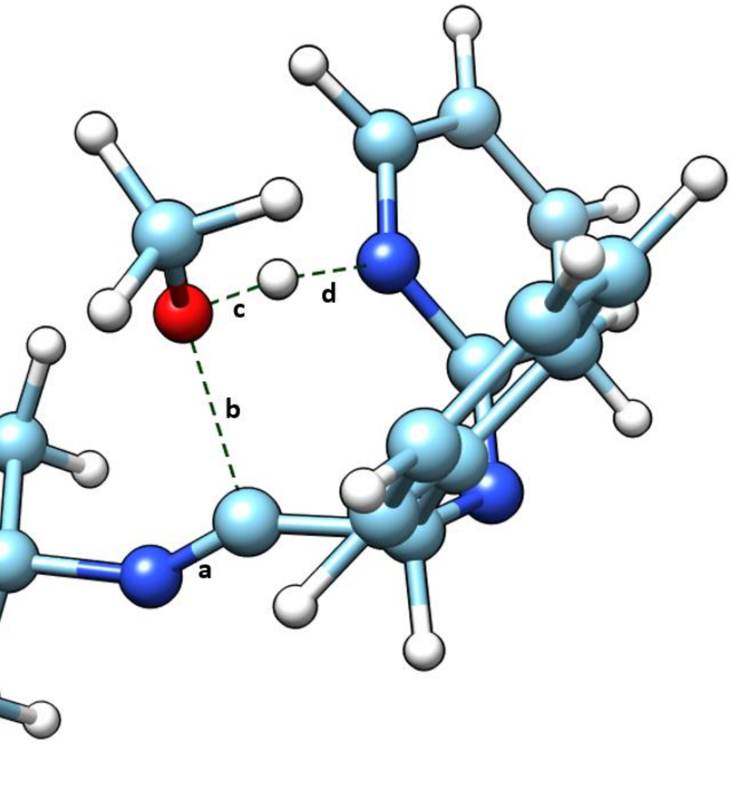

Figure S107. Molecular complex 4.

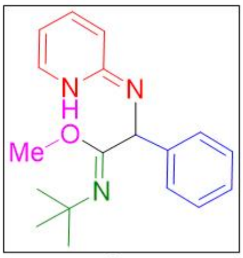

a: 1.26 Å
b: 1.40 Å
c: 1.86 Å
d: 1.02 Å

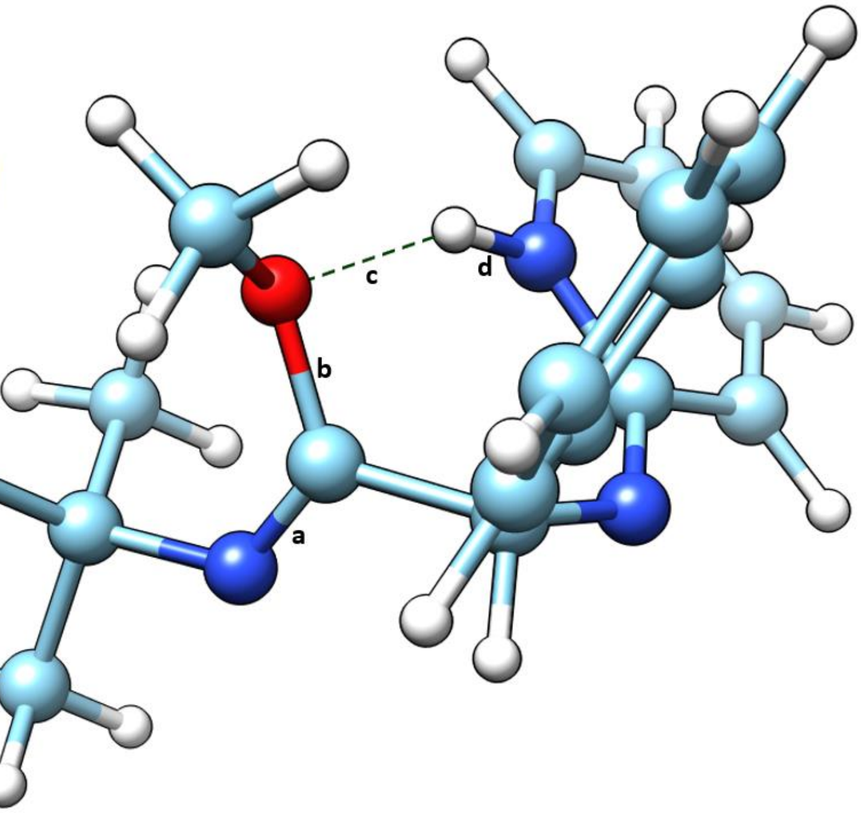

9.5. Proposal E: GBB reaction, methanol acts as a hydrogen bond donor in the first and second steps, and as a proton shuttle in the third step

9.5.1. Step 1 - Isocyanide nucleophilic attack, forming nitrilium (methanol as a hydrogen bond donor)

Figure S108. Molecular complex 1.

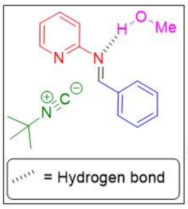

a: 1.17 Å
b: 3.03 Å
c: 1.28 Å
d: 1.41 Å
e: 1.33 Å
f: 2.00 Å

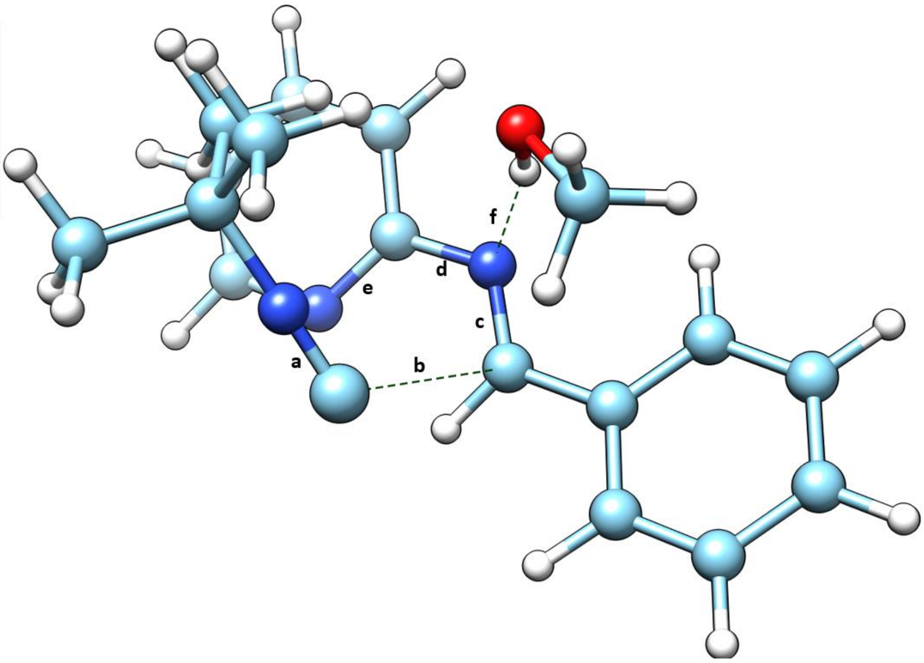

Figure S109. Transition State 1.

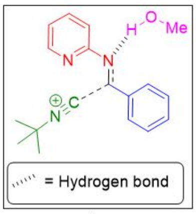

a: 1.16 Å
b: 1.73 Å
c: 1.36 Å
d: 1.37 Å
e: 1.35 Å
f: 1.83 Å

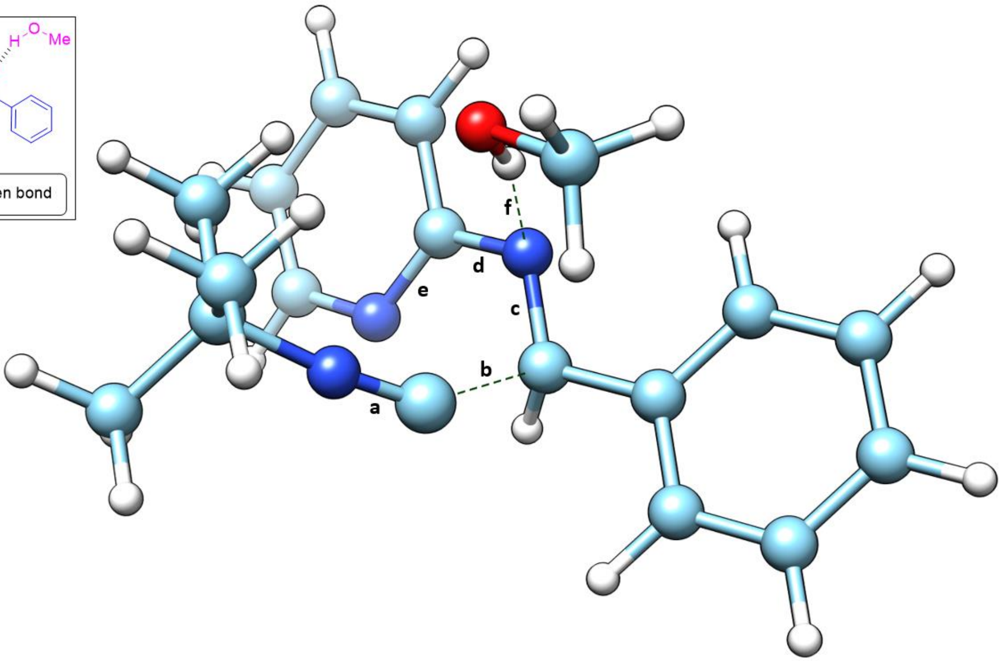

Figure S110. Molecular complex 2.

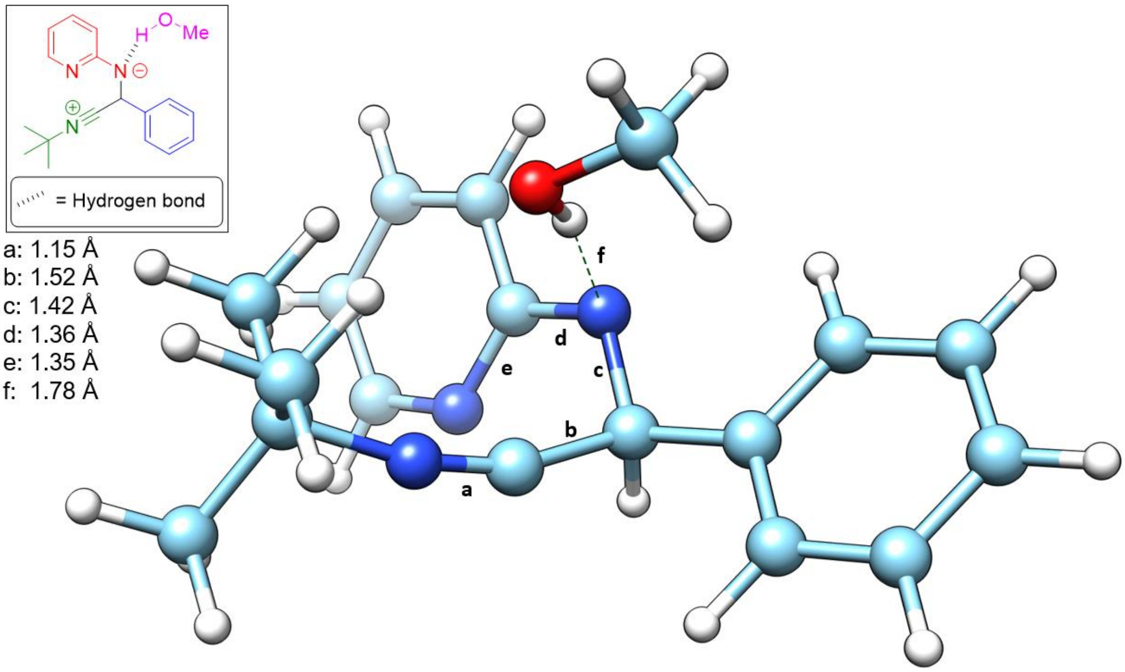

9.5.2. Step 2 – Ring Closure (methanol as a hydrogen bond donor)

Figure S111. Molecular complex 3.

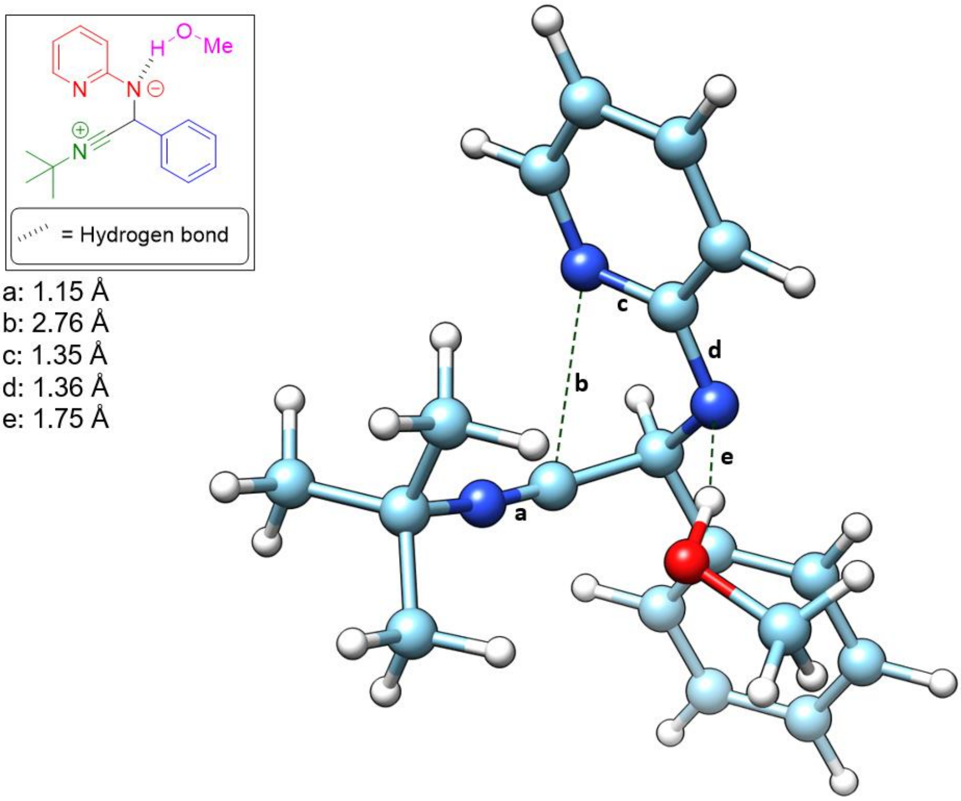

Figure S112. Transition State 2.

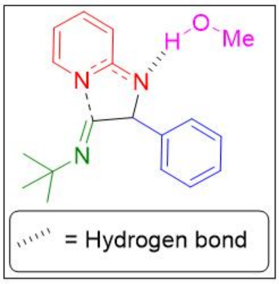

a: 1.16 Å
b: 2.20 Å
c: 1.36 Å
d: 1.34 Å
e: 1.90 Å

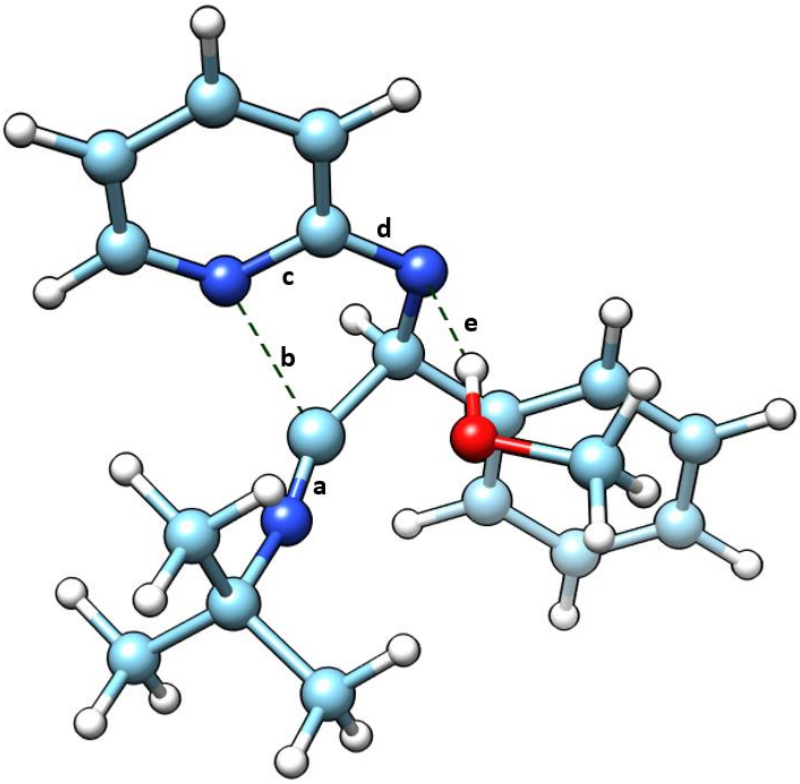

Figure S113. Molecular complex 4.

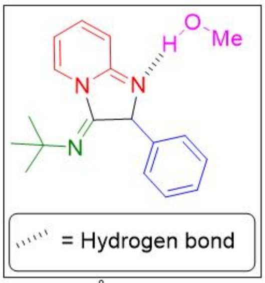

a: 1.26 Å
b: 1.43 Å
c: 1.42 Å
d: 1.29 Å
e: 3.52 Å

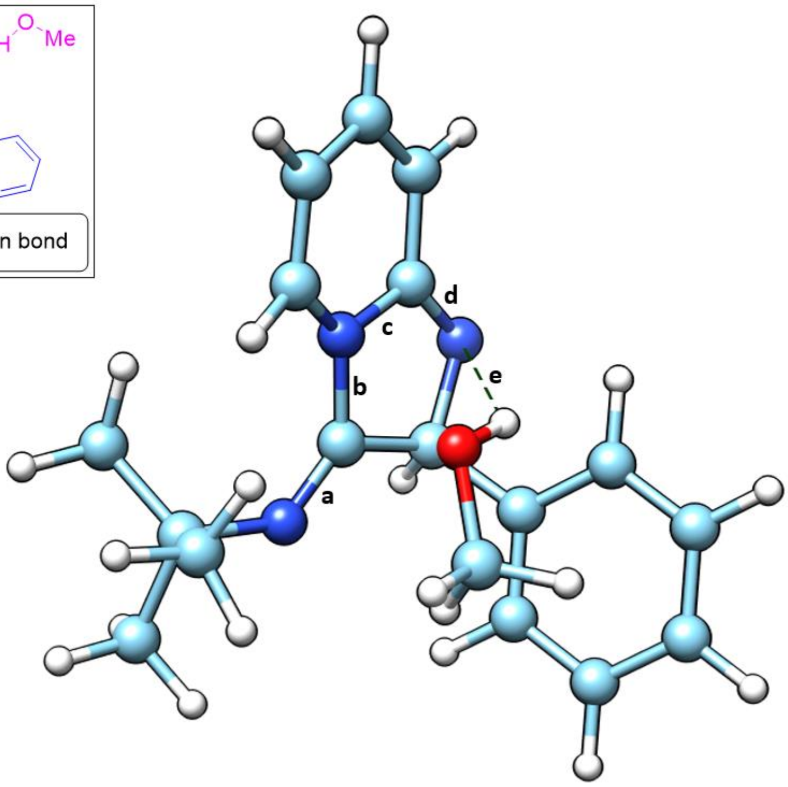

9.5.3. Step 3 – Methanol acting as a proton shuttle

Figure S114. Molecular complex 5.

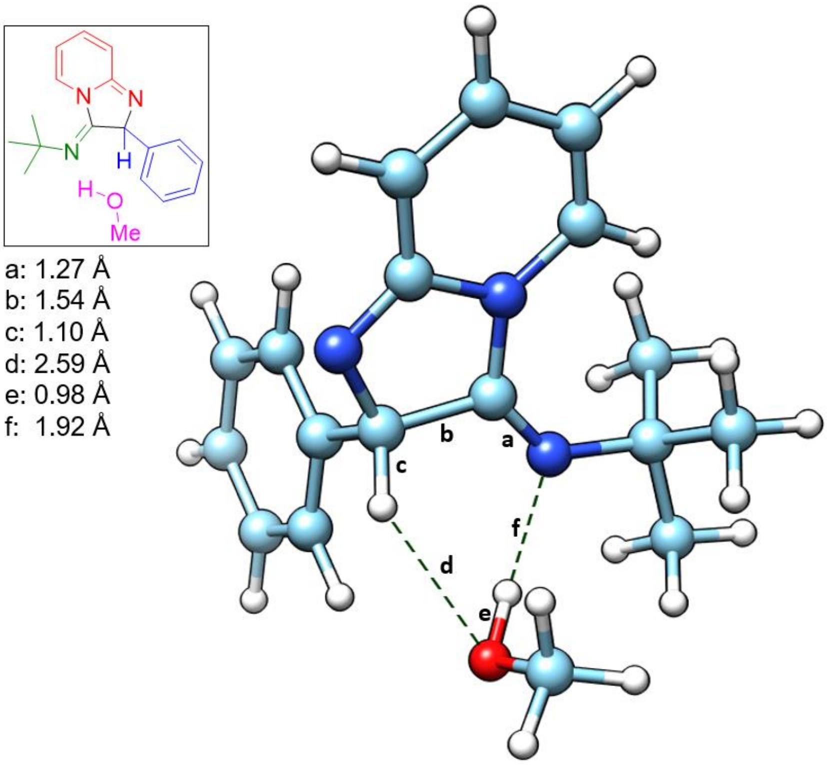

Figure S115. Transition State 3.

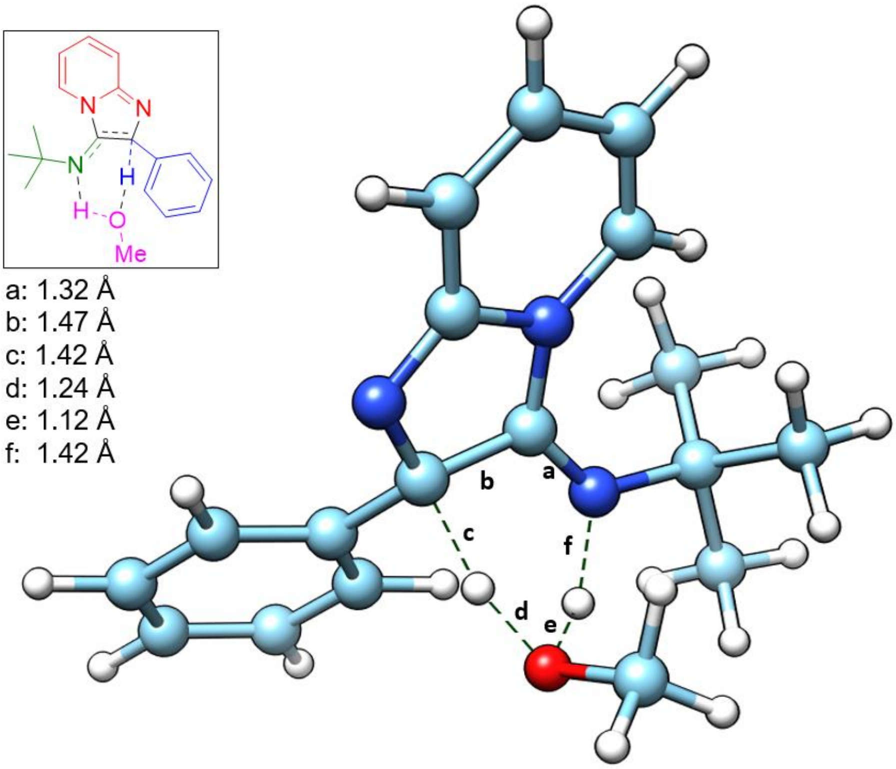

Figure S116. Molecular complex 6.

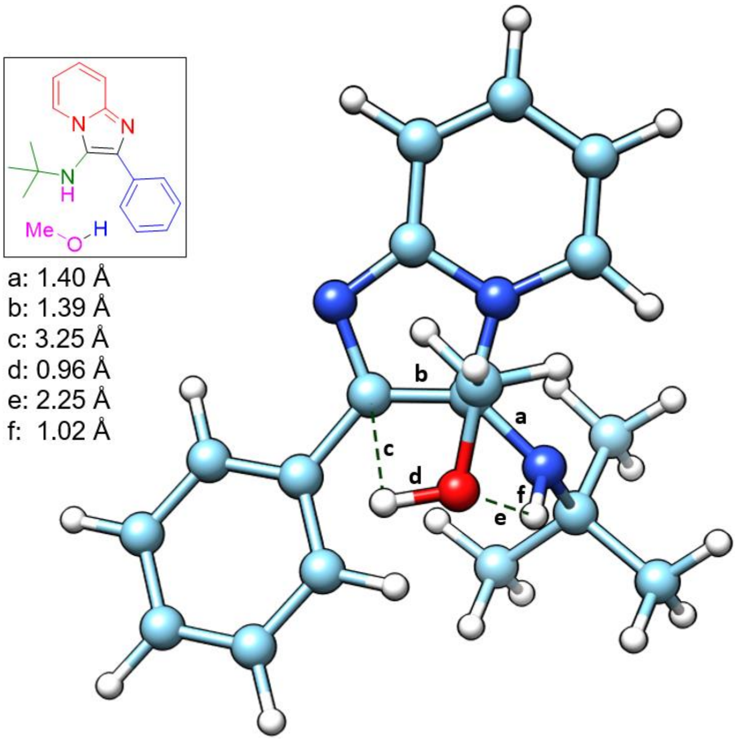

9.6. Proposal F: GBB reaction, methanol acts as a proton shuttle in the third step

9.6.1. Step 1 - Isocyanide nucleophilic attack, forming nitrilium

Figure S117. Molecular complex 1.

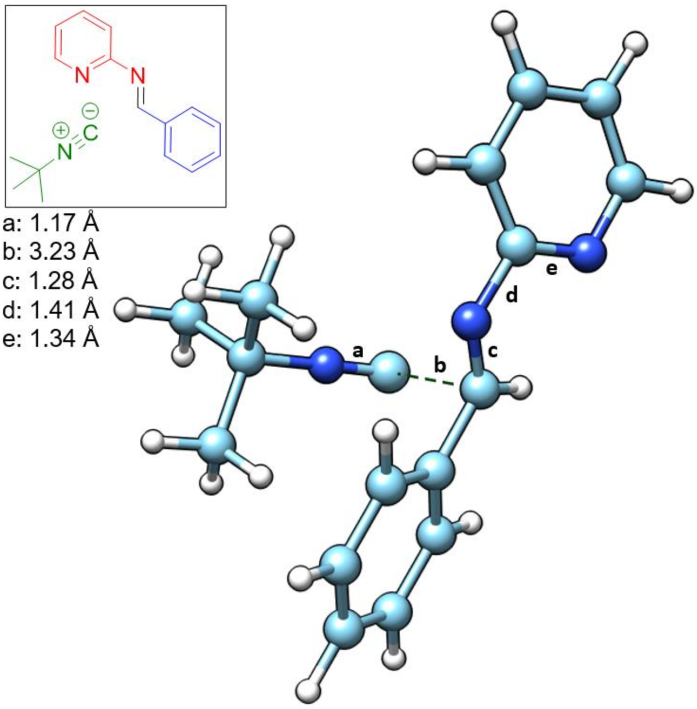

Figure S118. Transition State 1.

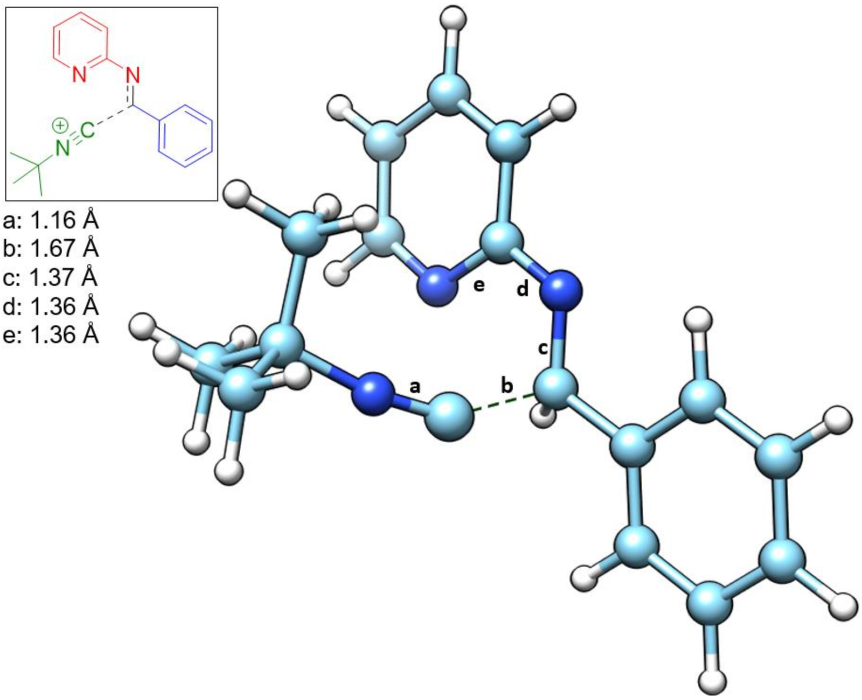

a: 1.16 Å
b: 1.67 Å
c: 1.37 Å
d: 1.36 Å
e: 1.36 Å

Figure S119. Molecular complex 2.

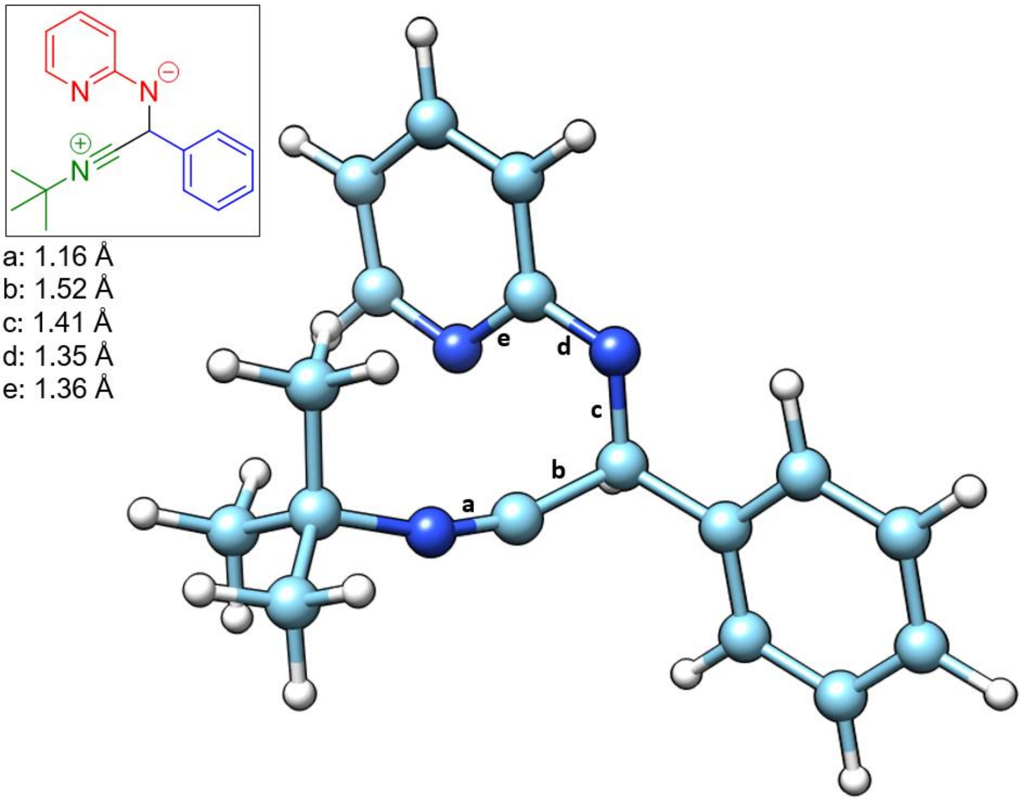

a: 1.16 Å
b: 1.52 Å
c: 1.41 Å
d: 1.35 Å
e: 1.36 Å

9.6.2. Step 2 – Ring closure

Figure S120. Molecular complex 3.

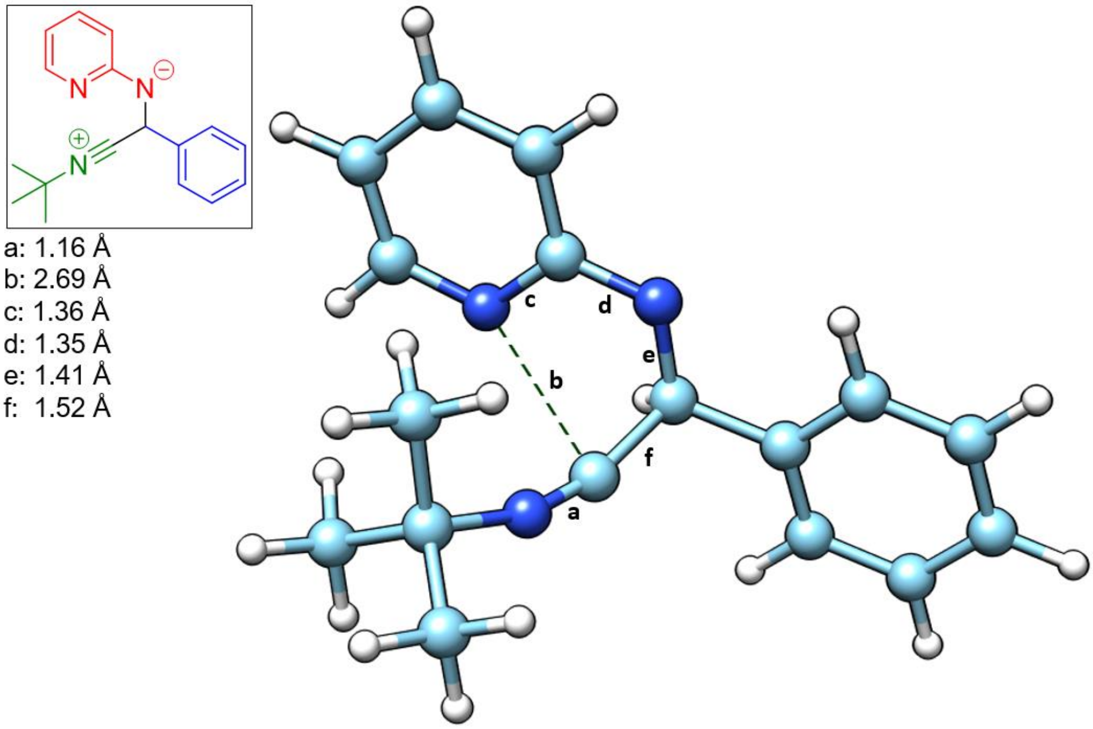

Figure S121. Transition State 2.

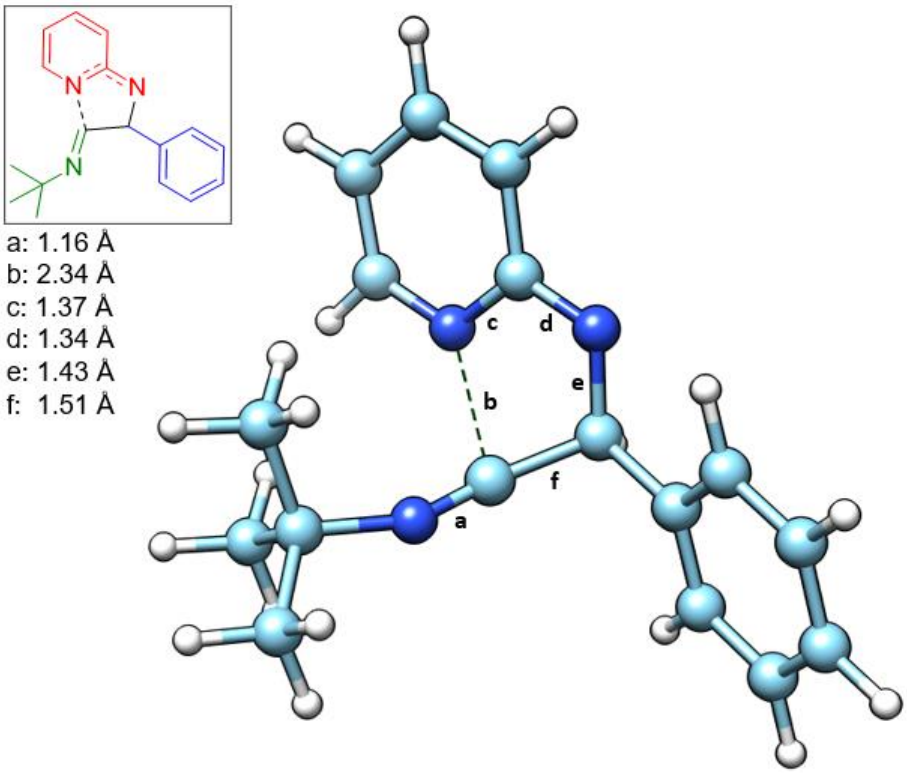

Figure S122. Molecular complex 4.

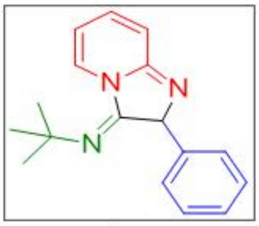

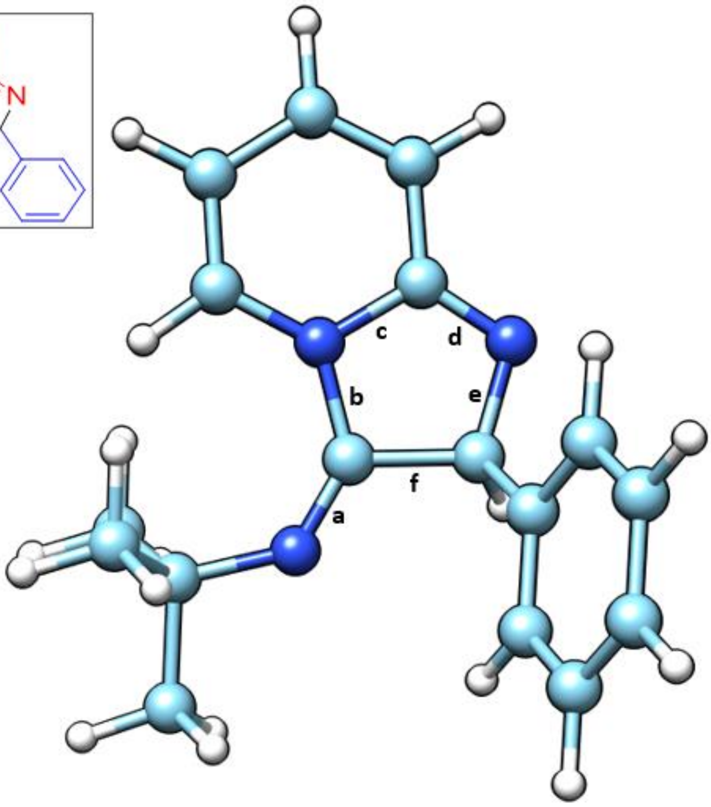

a: 1.26 Å
b: 1.43 Å
c: 1.42 Å
d: 1.28 Å
e: 1.45 Å
f: 1.54 Å

9.6.3. Step 3 – Methanol acting as a proton shuttle

Figure S123. Molecular complex 5.

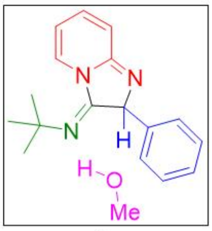

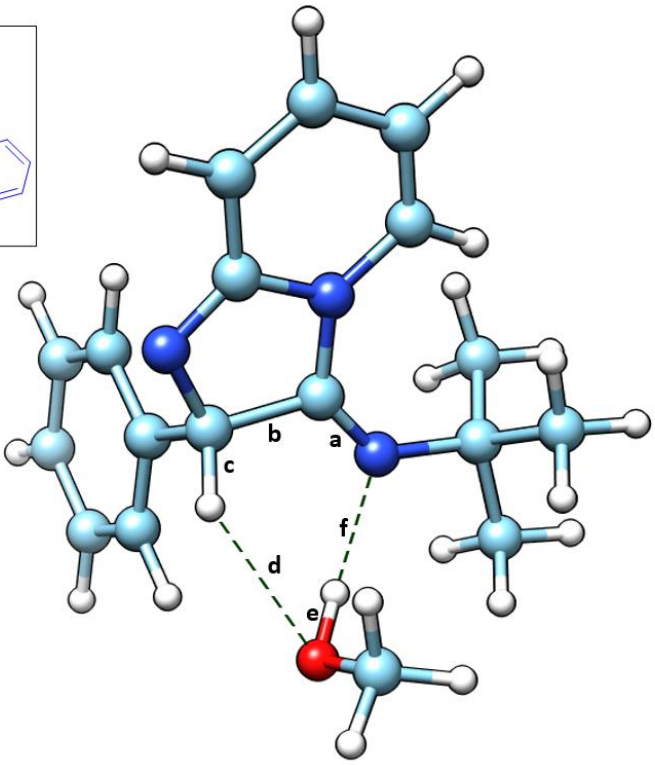

a: 1.27 Å
b: 1.54 Å
c: 1.10 Å
d: 2.59 Å
e: 0.98 Å
f: 1.92 Å

Figure S124. Transition State 3.

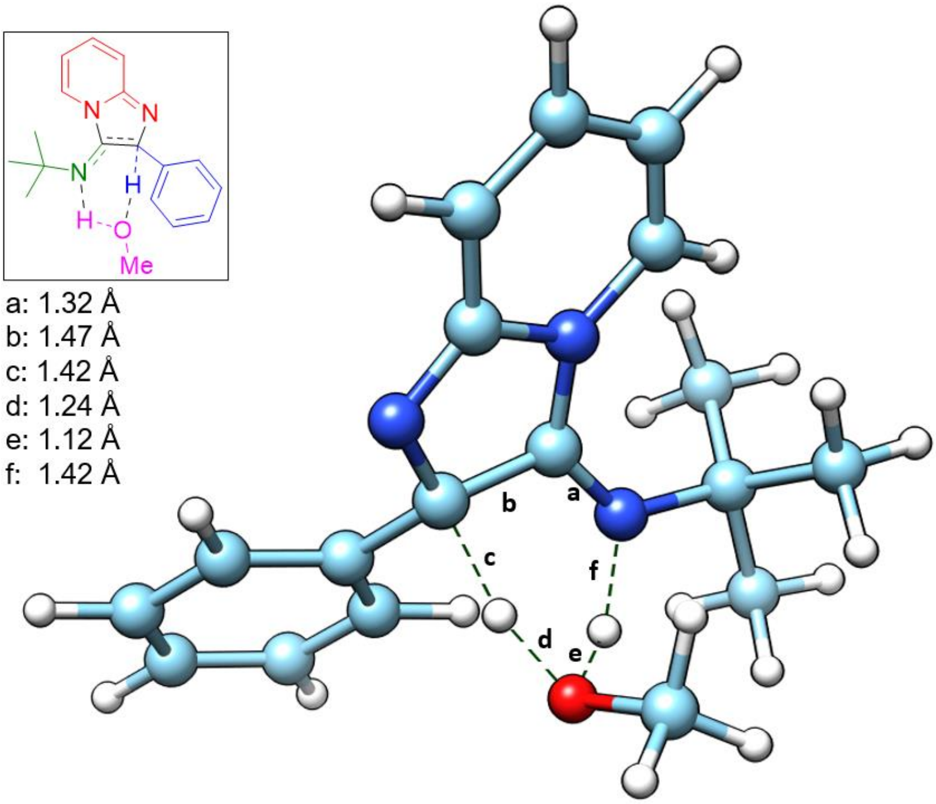

Figure S125. Molecular complex 6.

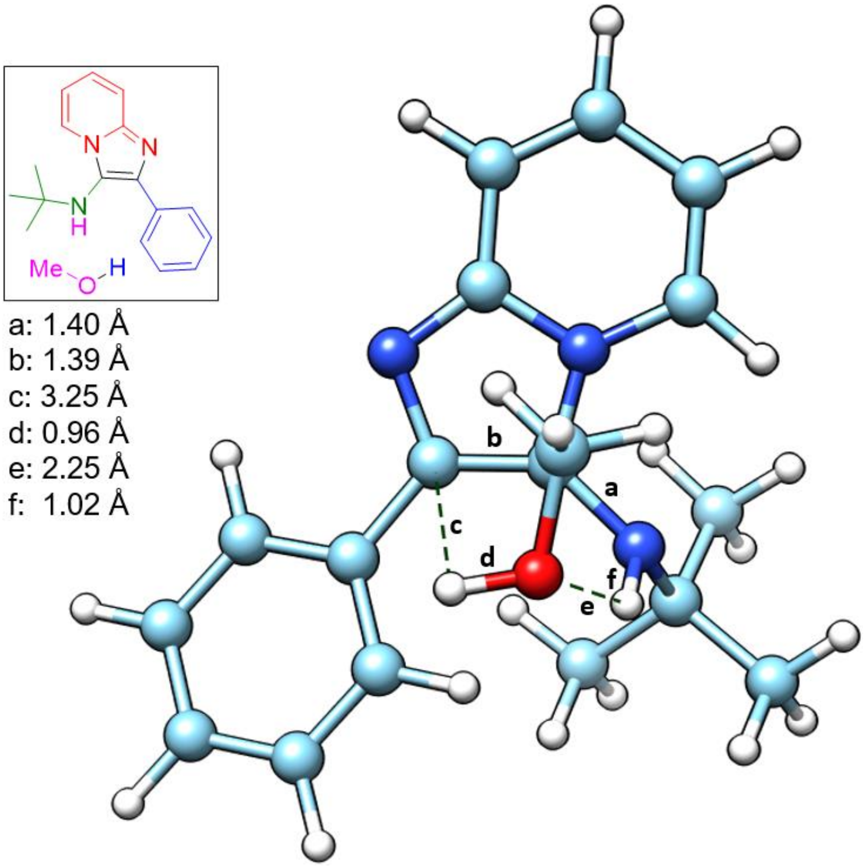

9.7. Proposal G: GBB reaction, methanol acts as a hydrogen bond donor in the first and second steps, and 2-aminopyridine acts a proton shuttle in the third step

9.7.1. Step 1 - Isocyanide nucleophilic attack, forming nitrilium (methanol as a hydrogen bond donor)

Figure S126. Molecular complex 1.

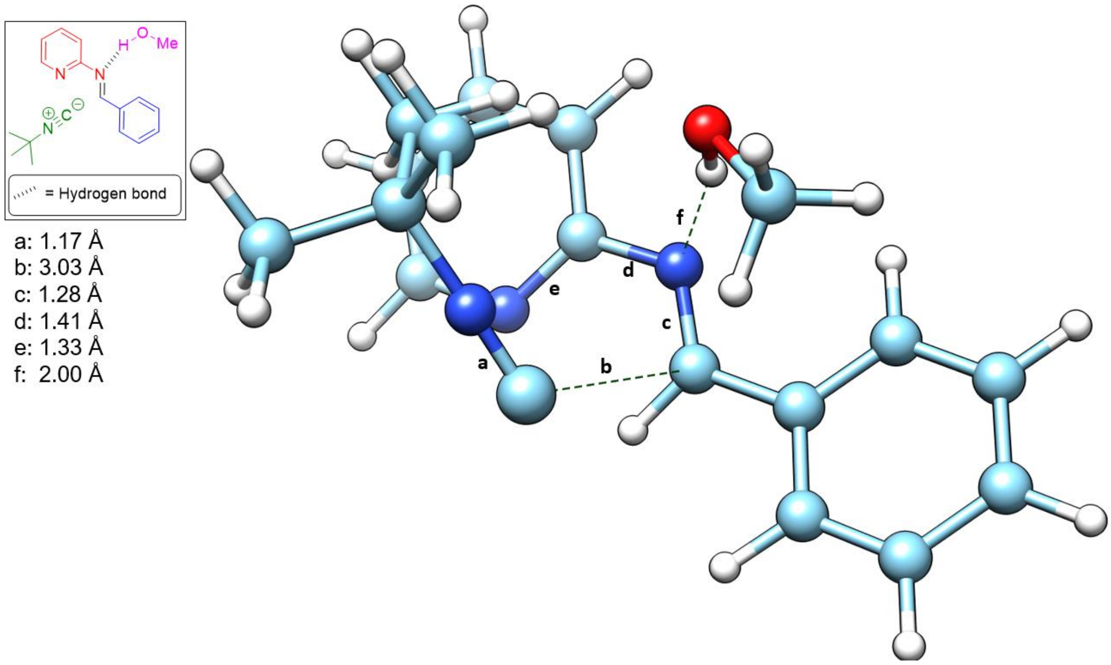

Figure S127. Transition State 1.

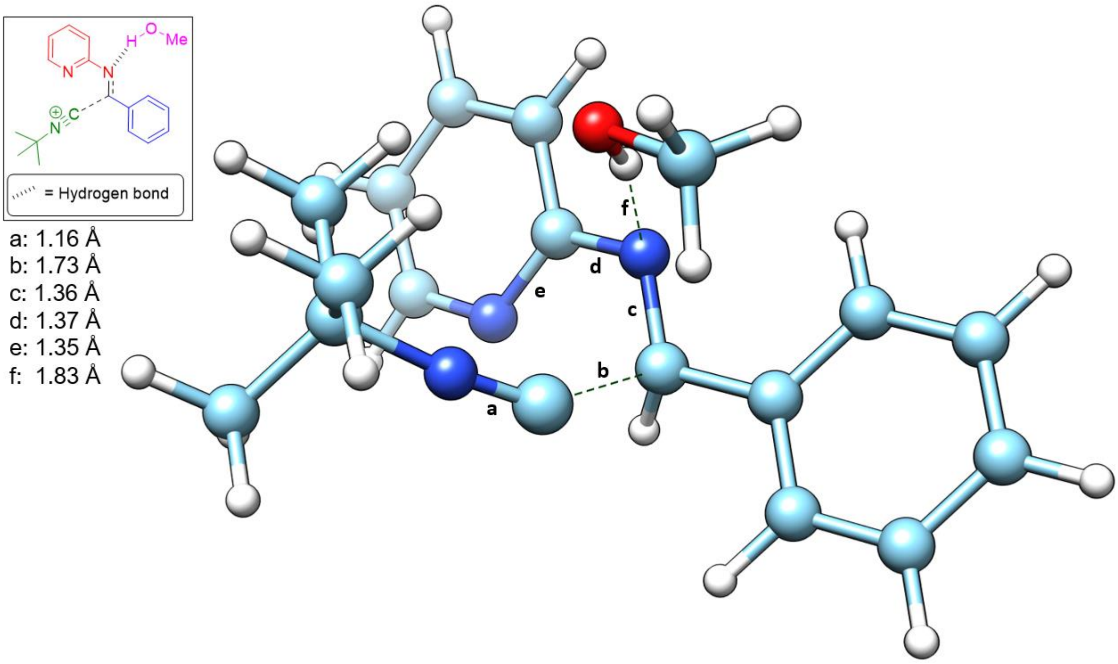

Figure S128. Molecular complex 2.

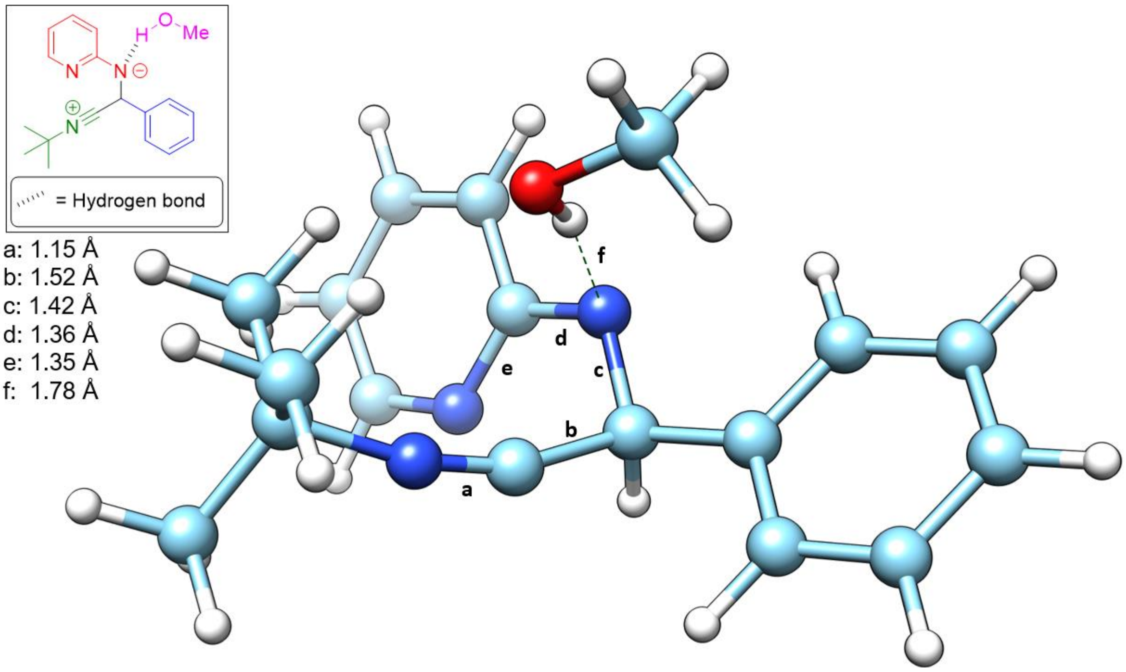

9.7.2. Step 2 – Ring Closure (methanol as a hydrogen bond donor)

Figure S129. Molecular complex 3.

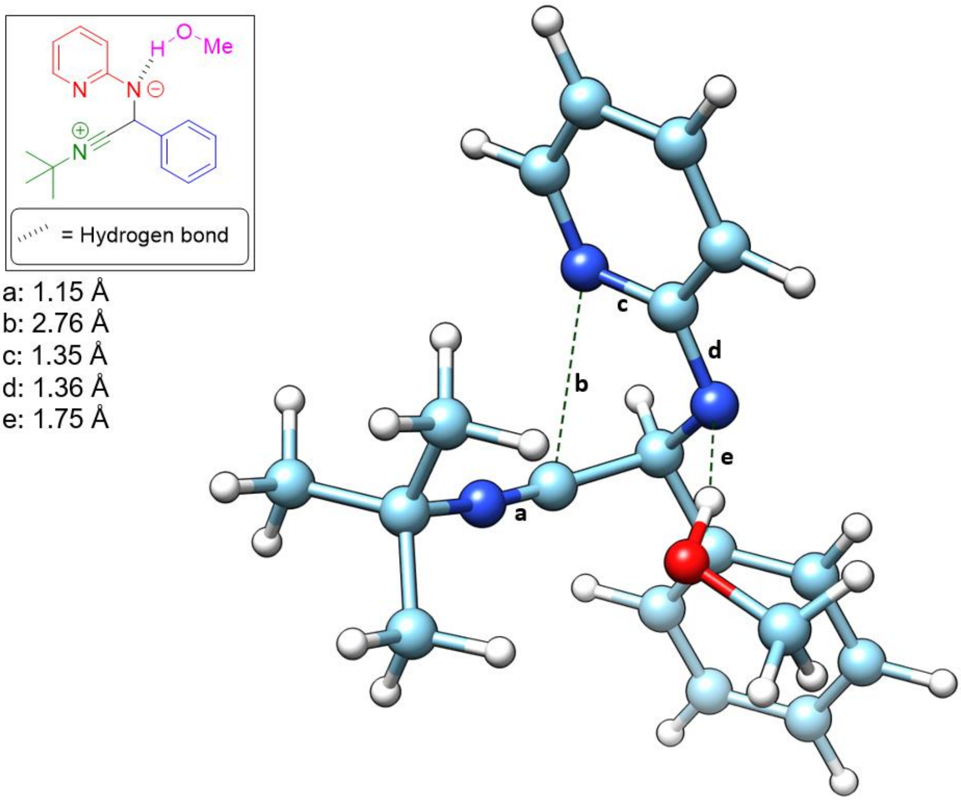

Figure S130. Transition State 2.

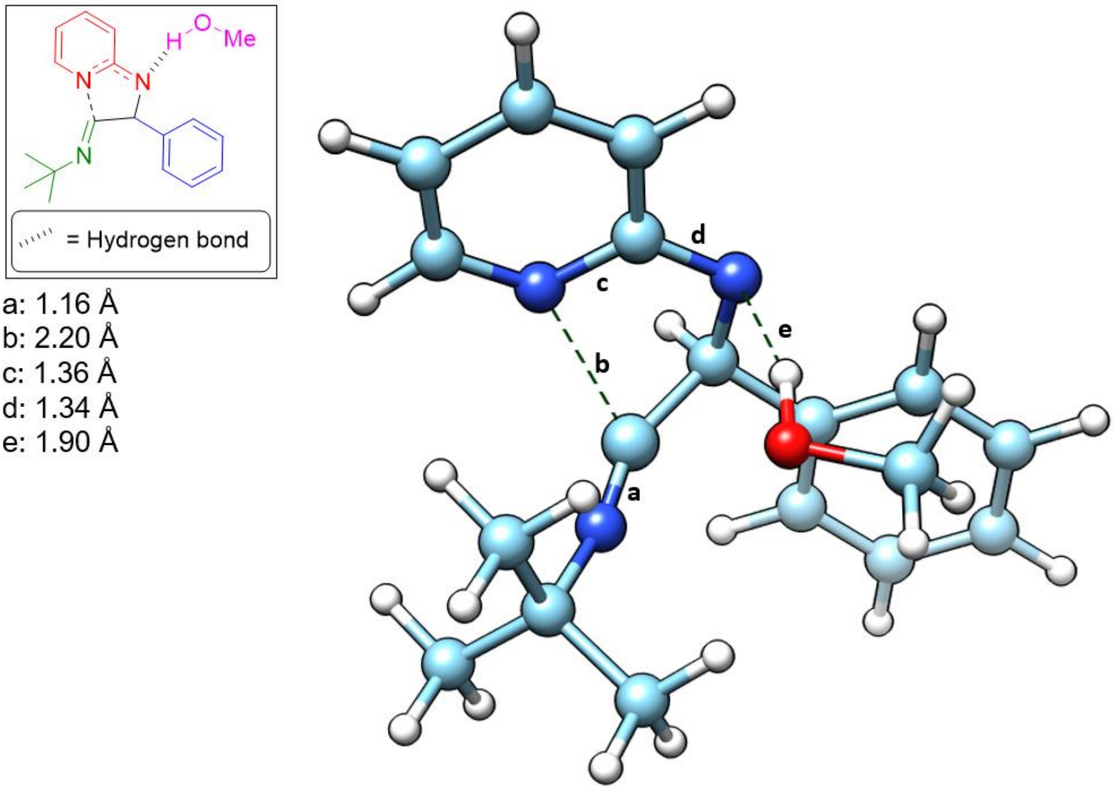

Figure S131. Molecular complex 4.

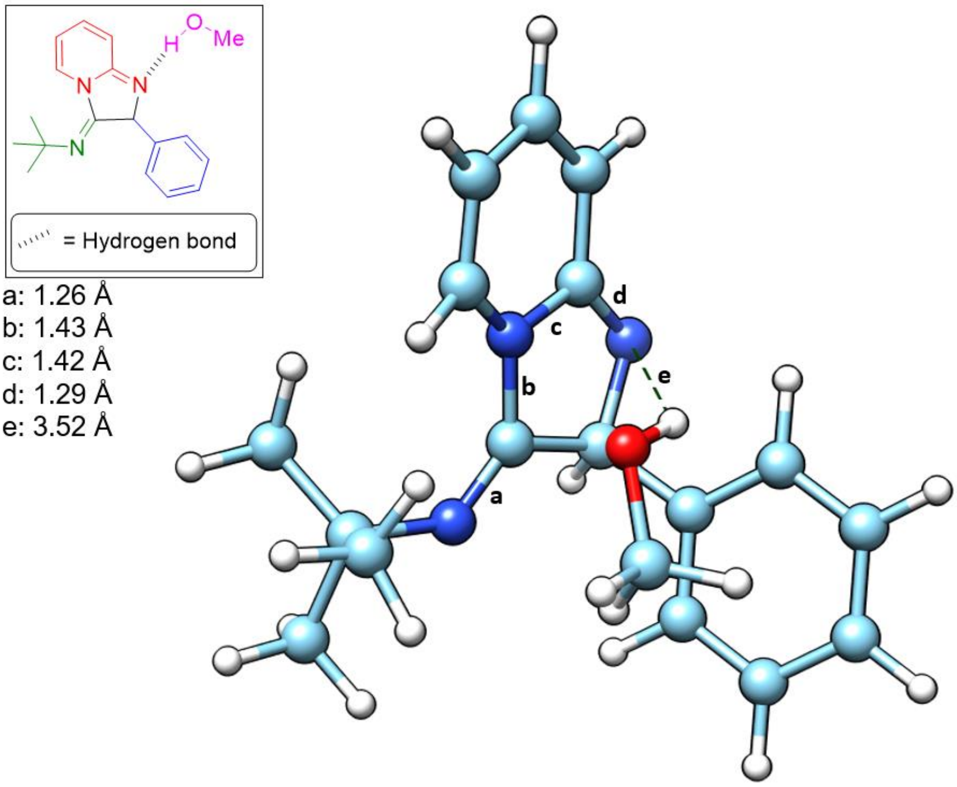

9.7.3. Step 3 – 2-aminopyridine acting as a proton shuttle

Figure S132. Molecular complex 5.

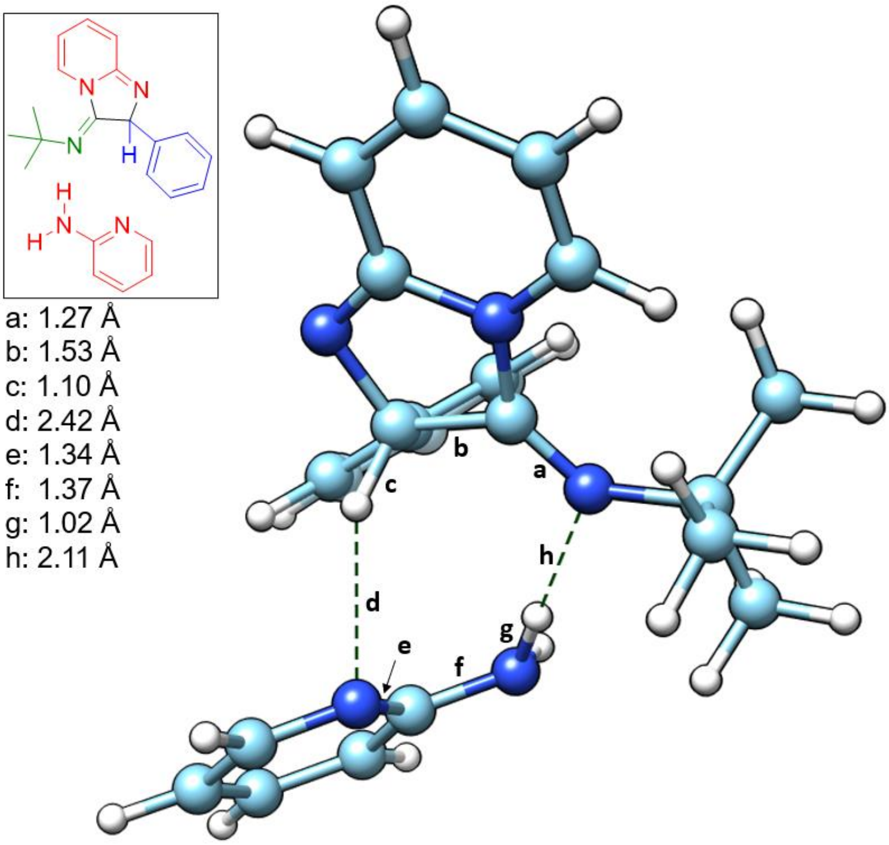

a: 1.27 Å
b: 1.53 Å
c: 1.10 Å
d: 2.42 Å
e: 1.34 Å
f: 1.37 Å
g: 1.02 Å
h: 2.11 Å

Figure S133. Transition State 3.

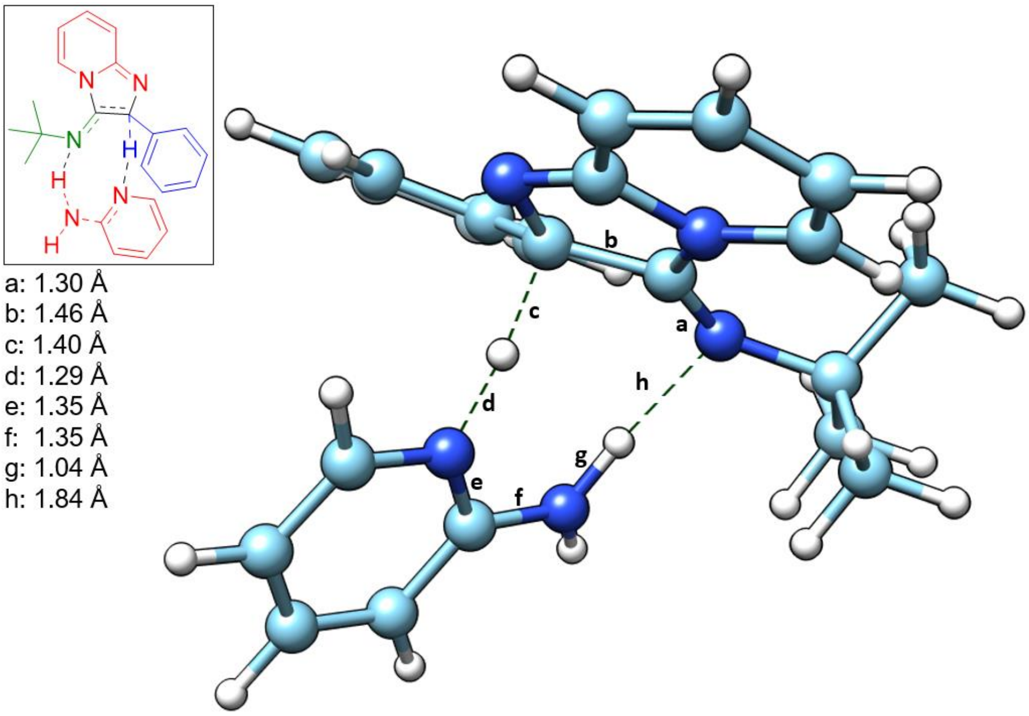

a: 1.30 Å
b: 1.46 Å
c: 1.40 Å
d: 1.29 Å
e: 1.35 Å
f: 1.35 Å
g: 1.04 Å
h: 1.84 Å

Figure S134. Molecular complex 6.

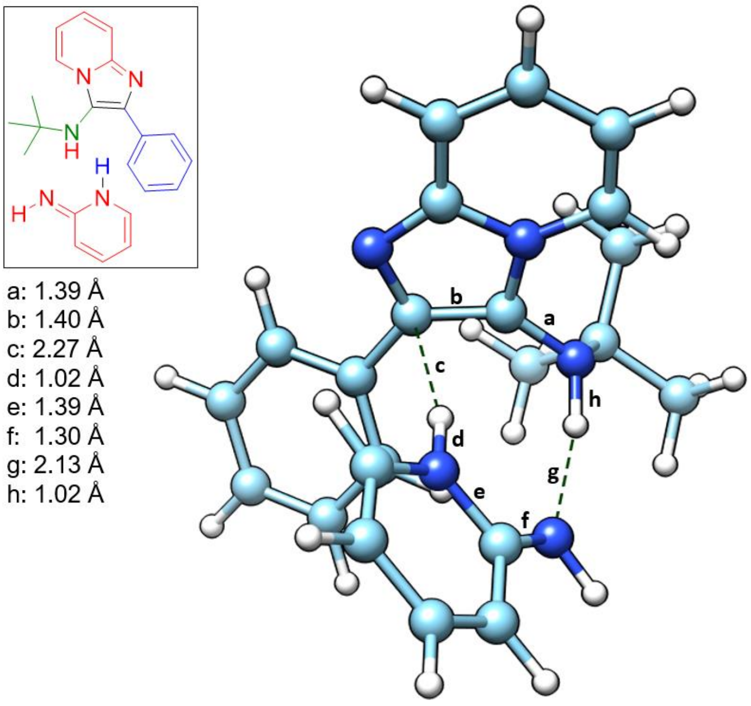

9.8. Proposal H: GBB reaction catalyzed by *p*-toluenesulfonic acid

9.8.1. Step 1 - Isocyanide nucleophilic attack, forming nitrilium

Figure S135. Molecular complex 1.

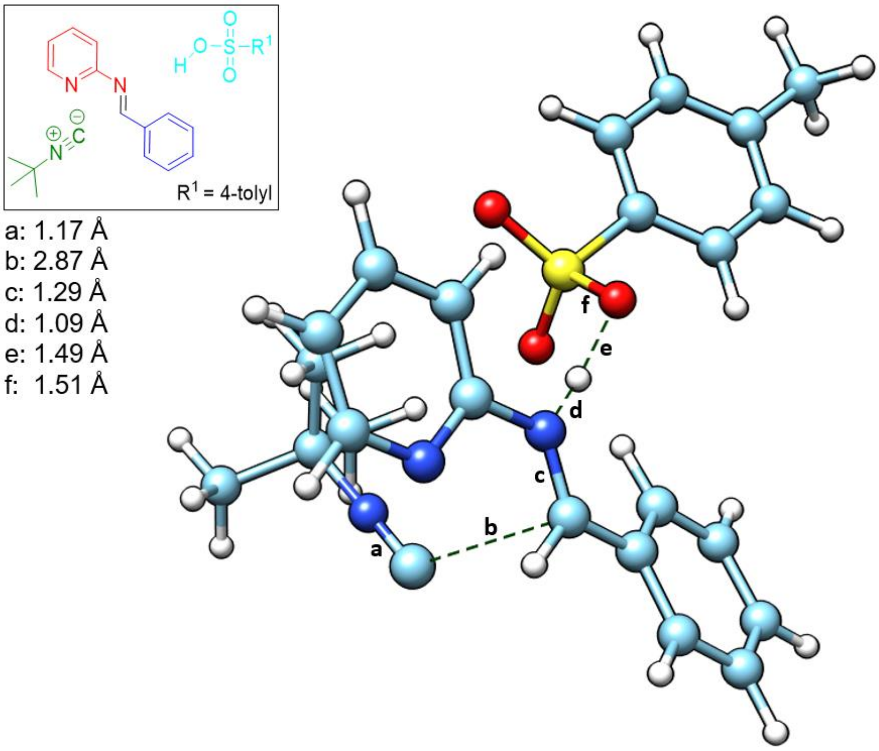

Figure S136. Transition State 1.

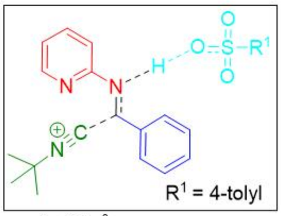

a: 1.16 Å
b: 2.00 Å
c: 1.33 Å
d: 1.05 Å
e: 1.70 Å
f: 1.50 Å

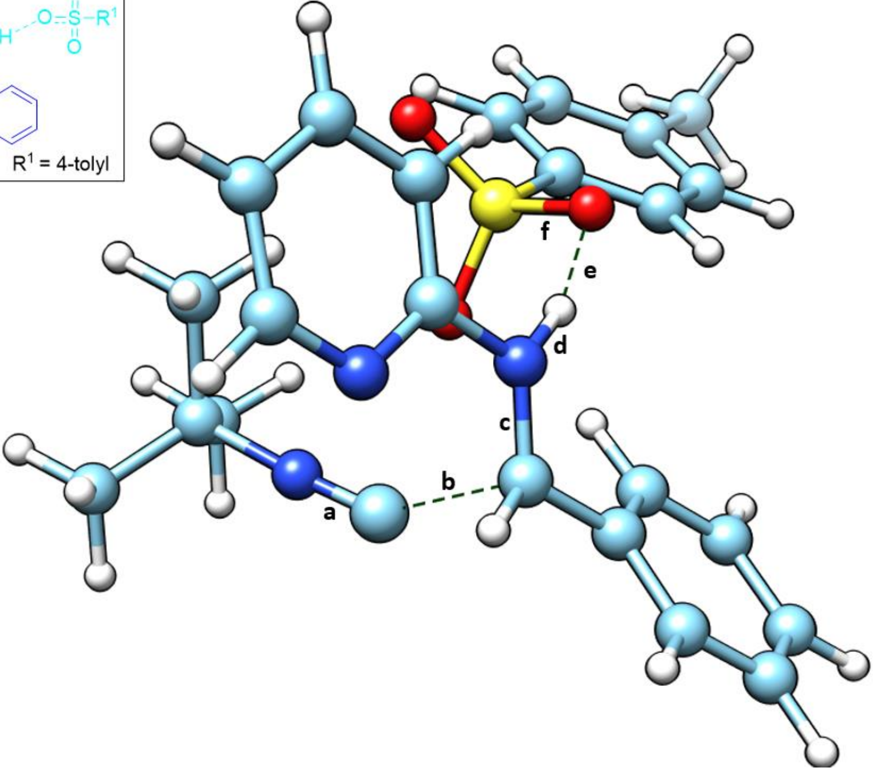

Figure S137. Molecular complex 2.

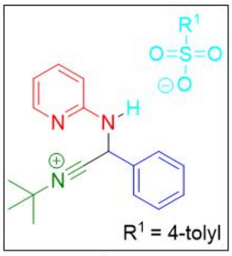

a: 1.15 Å
b: 1.51 Å
c: 1.44 Å
d: 1.03 Å
e: 1.84 Å
f: 1.49 Å

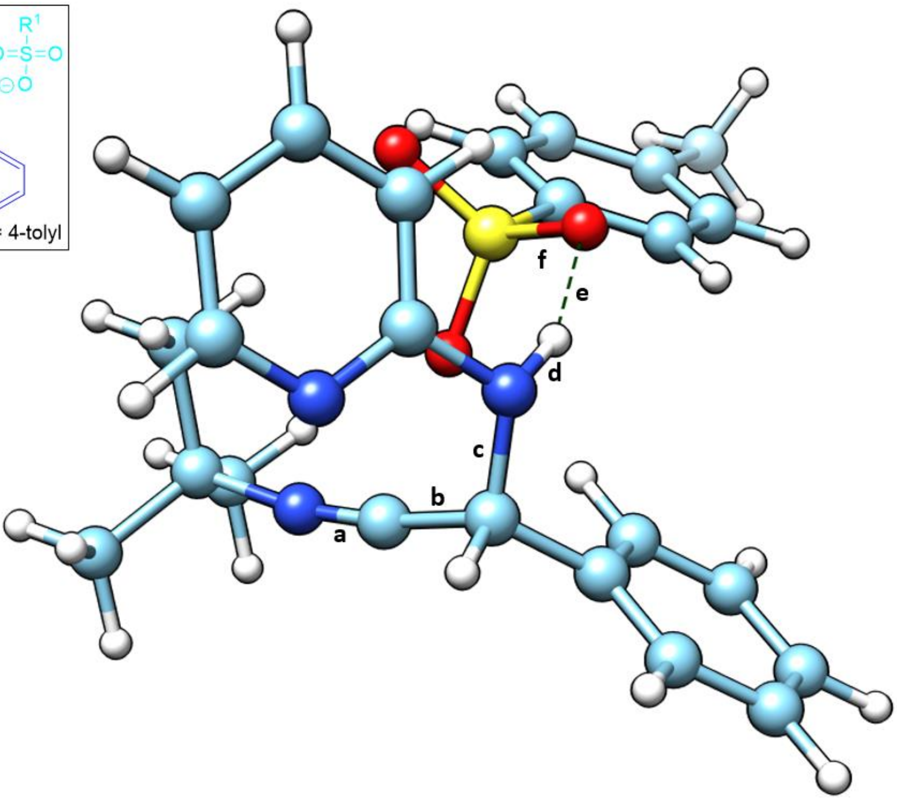

9.8.2. Step 2 – Ring Closure

Figure S138. Molecular complex 3.

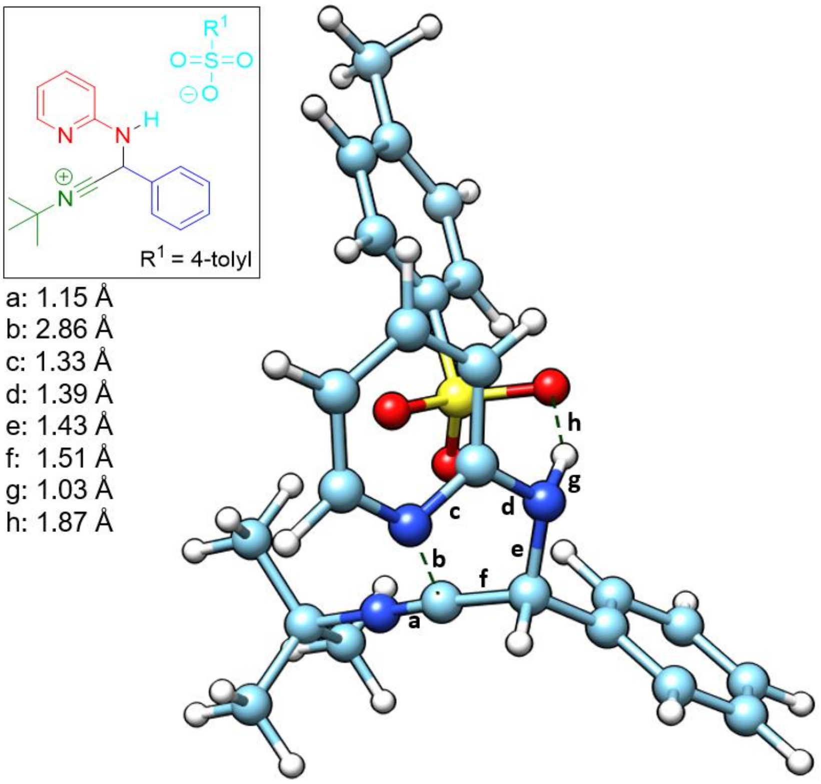

Figure S139. Transition State 2.

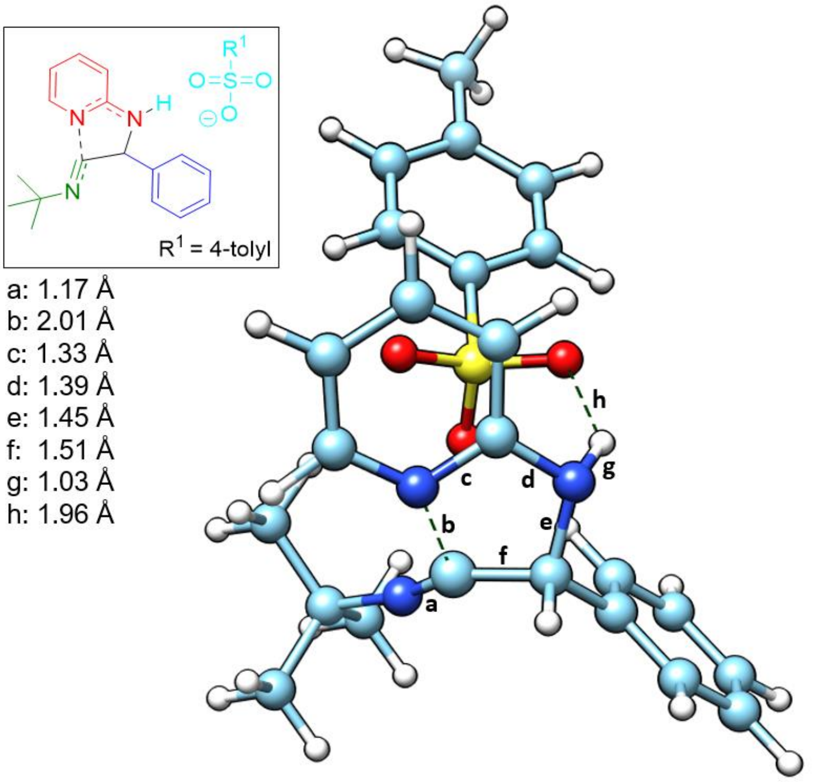

Figure S140. Molecular complex 4.

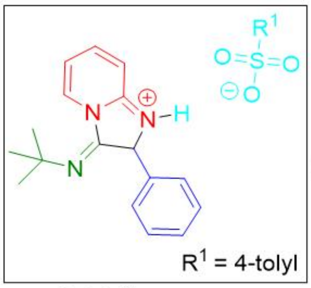

a: 1.24 Å
b: 1.47 Å
c: 1.36 Å
d: 1.34 Å
e: 1.47 Å
f: 1.53 Å
g: 1.03 Å
h: 1.91 Å

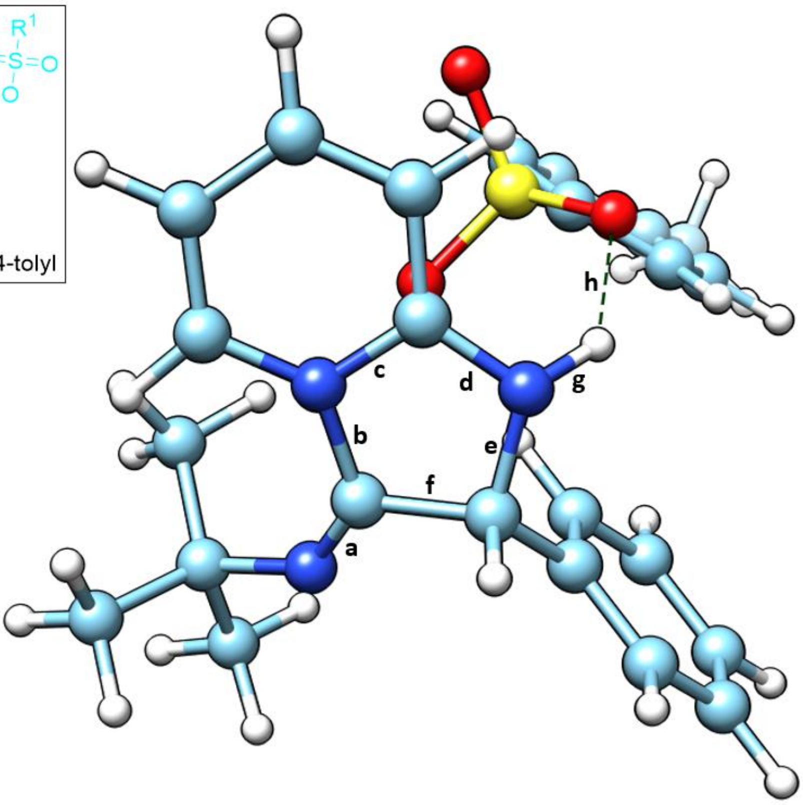

9.8.3. Step 3 – Proton abstraction by tosylate

Figure S141. Molecular complex 5.

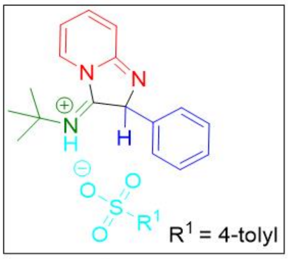

a: 1.30 Å
b: 1.53 Å
c: 1.10 Å
d: 2.39 Å
e: 1.48 Å

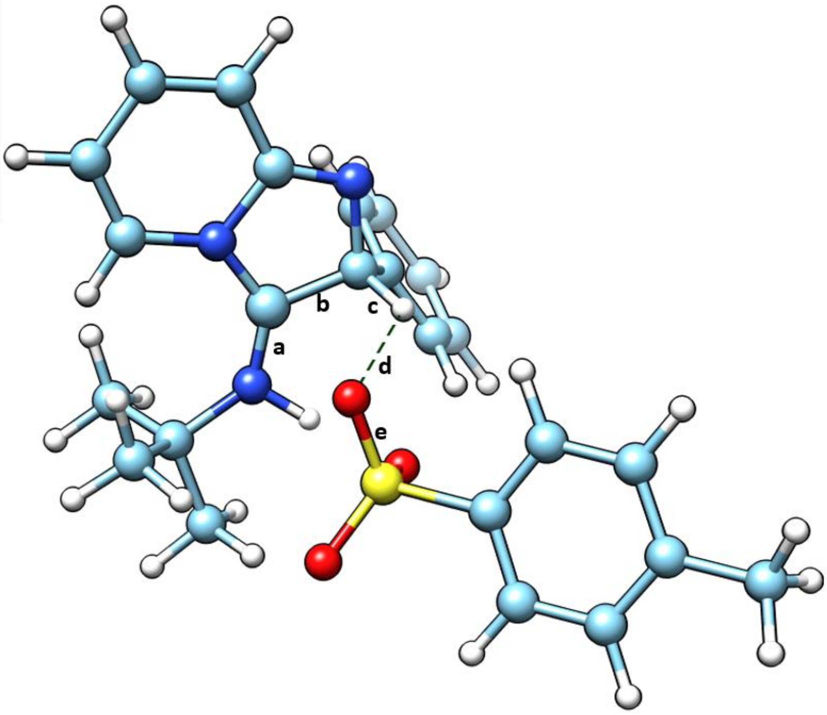

Figure S142. Transition State 3.

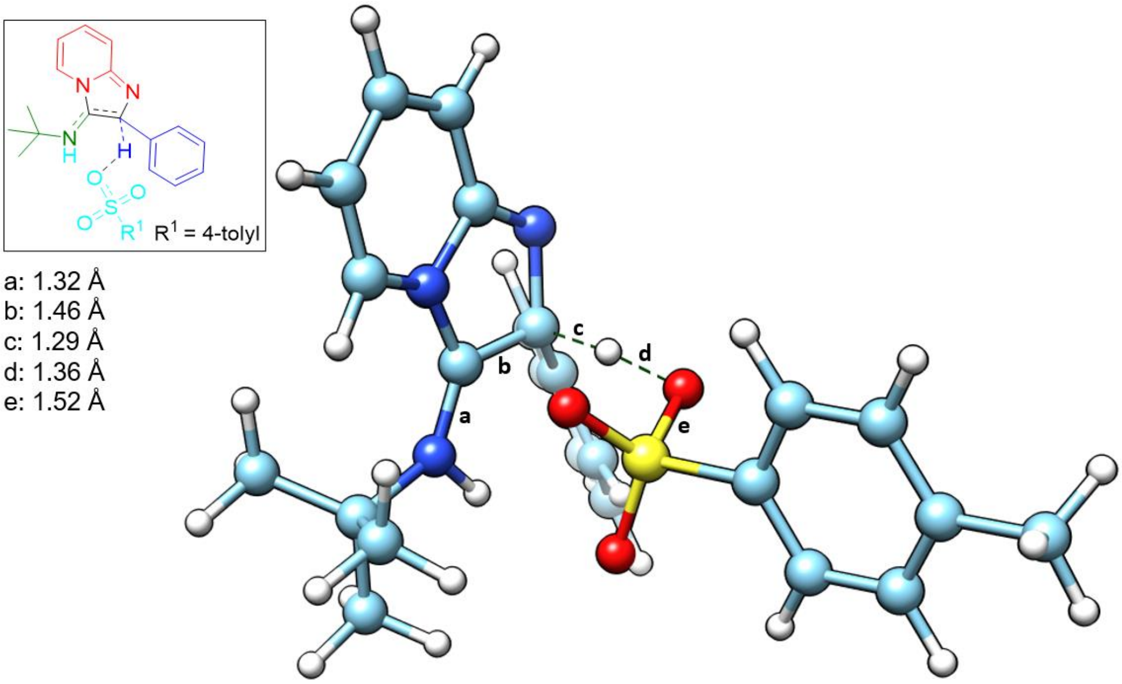

Figure S143. Molecular complex 6.

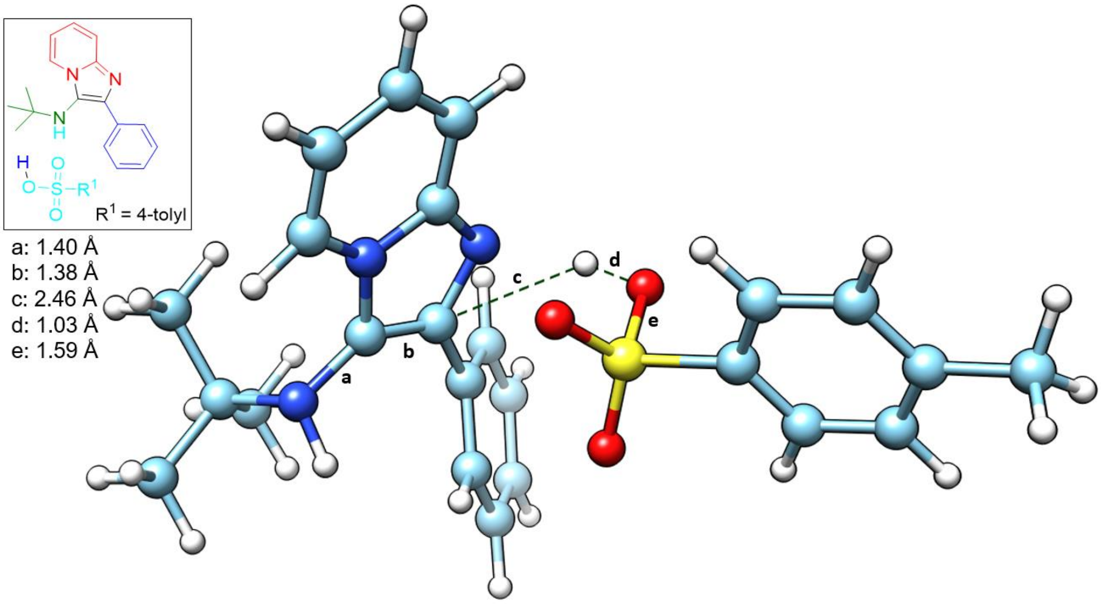

9.9. Proposal I: GBB reaction with a participation of methanol in mechanism, forming a tetrahedral intermediate, catalyzed by *p*-toluenesulfonic acid

9.9.1. Step 1 - Isocyanide nucleophilic attack, forming nitrilium

Figure S144. Molecular complex 1.

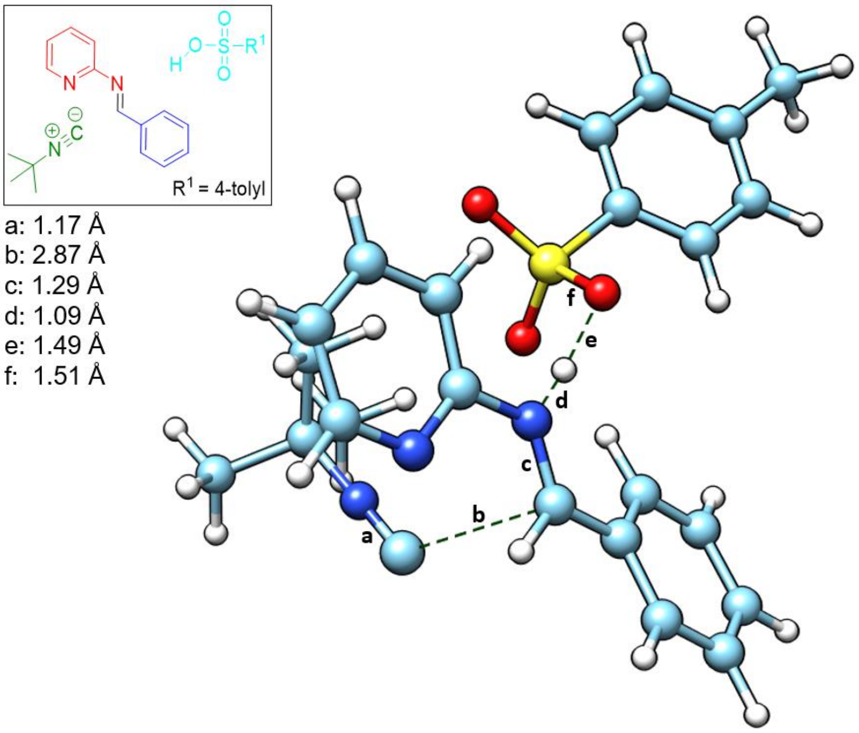

Figure S145. Transition State 1.

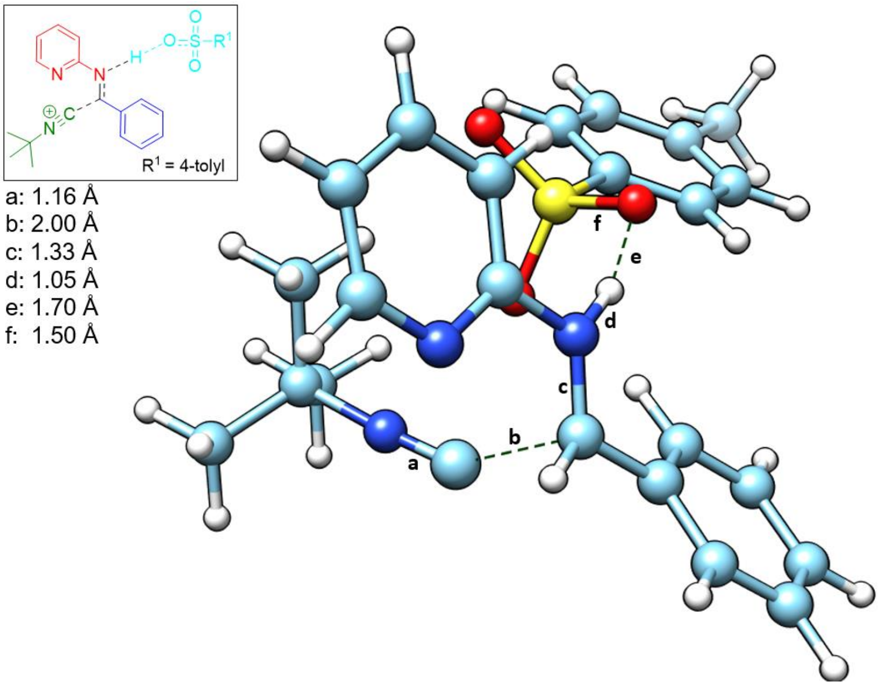

Figure S146. Molecular complex 2.

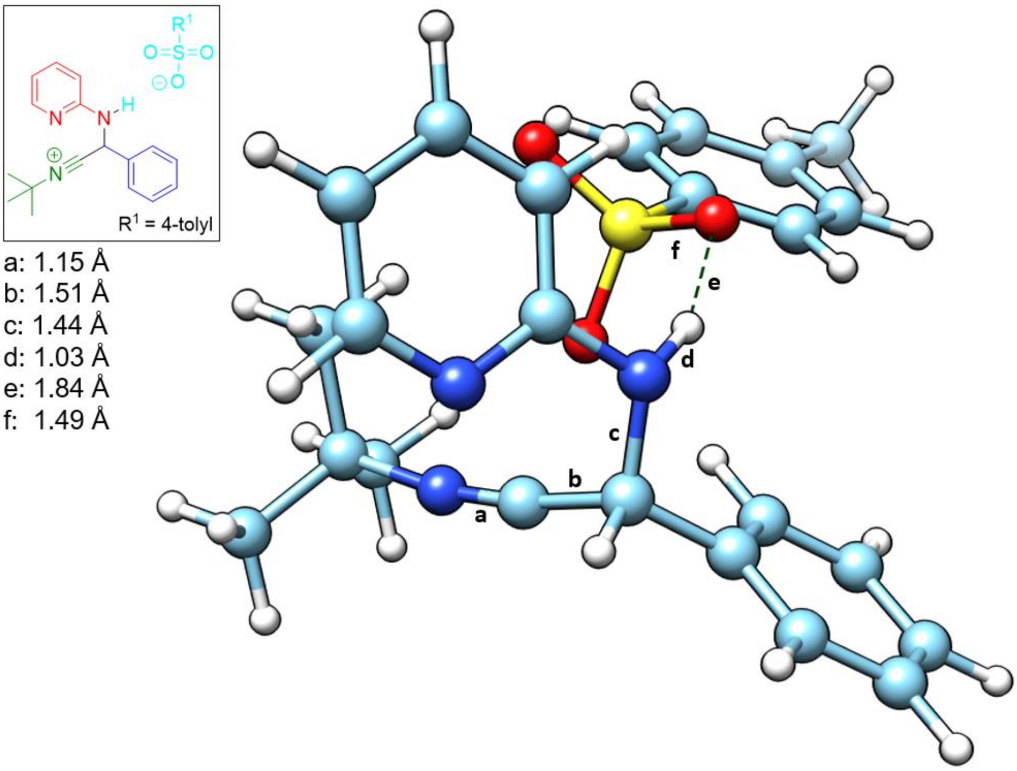

9.9.2. Step 2 – Methanol addition to nitrilium

Figure S147. Molecular complex 3.

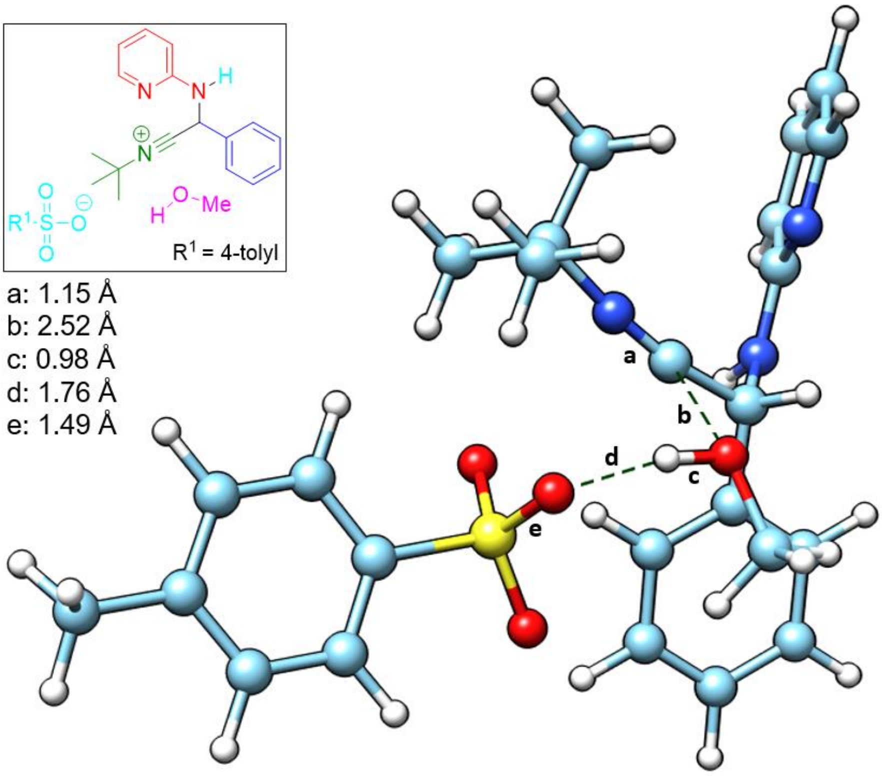

Figure S148. Transition State 2.

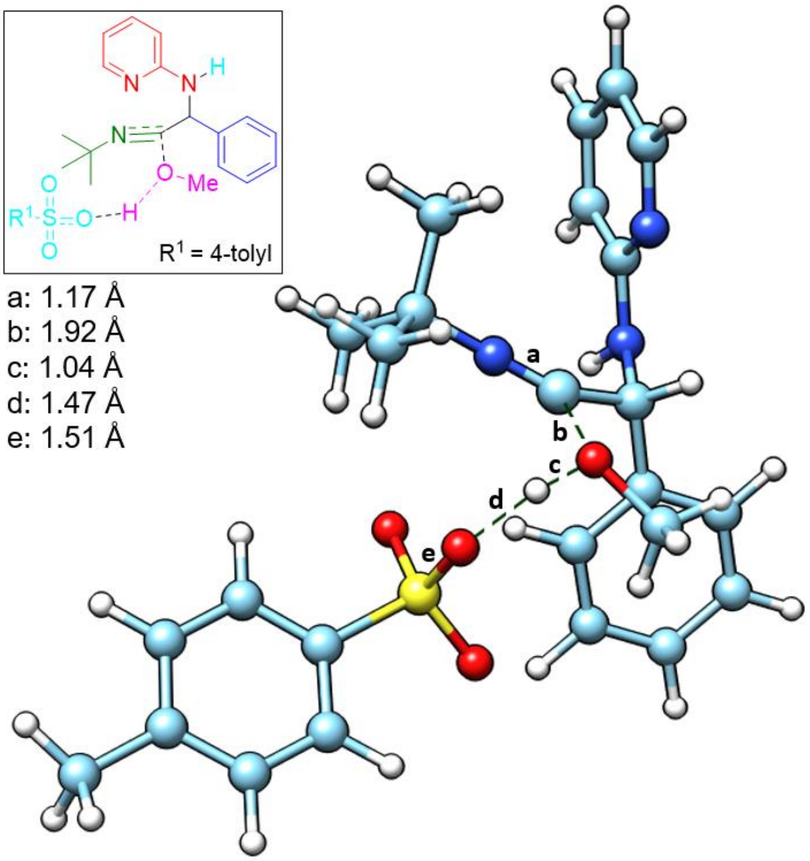

Figure S149. Molecular complex 4.

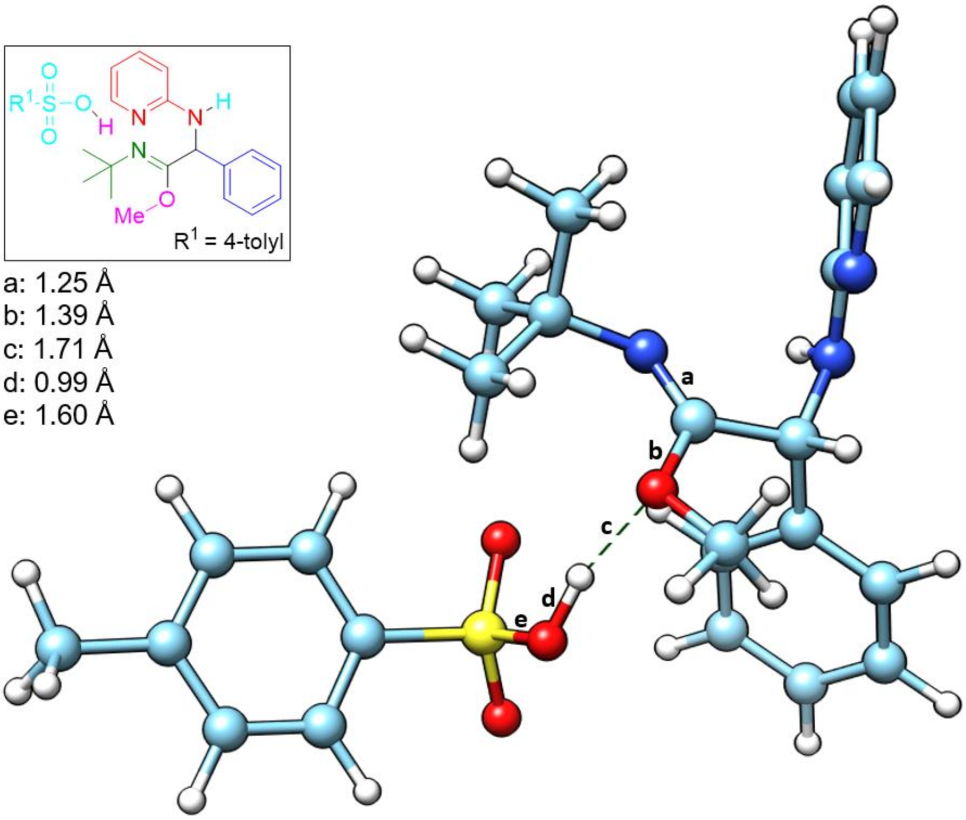

9.9.3. Step 3 – Ring closure, forming a tetrahedral intermediate

Figure S150. Molecular complex 5.

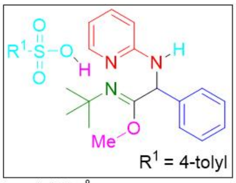

a: 1.53 Å
b: 1.43 Å
c: 1.38 Å
d: 1.34 Å
e: 2.96 Å
f: 1.29 Å
g: 1.05 Å
h: 1.73 Å
i: 1.50 Å

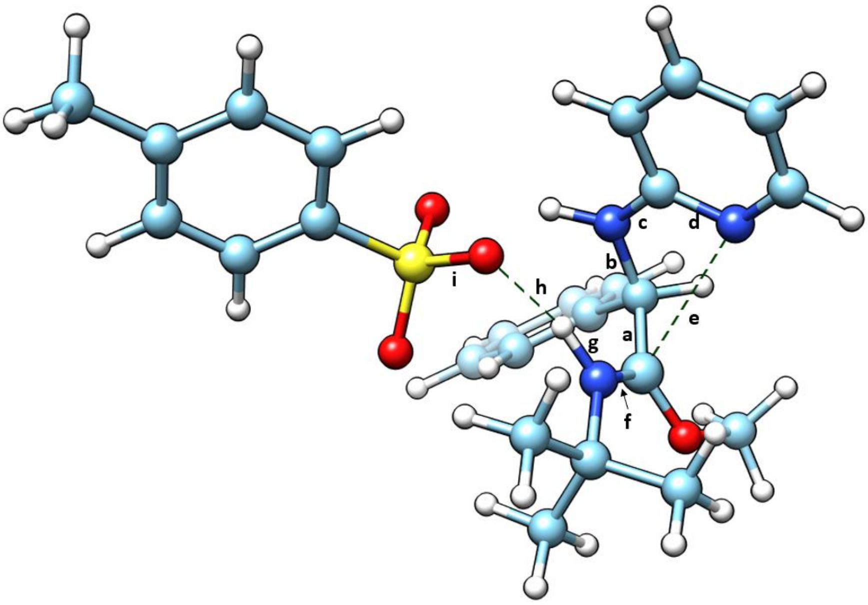

Figure S151. Transition State 3.

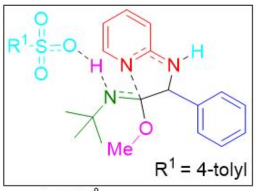

a: 1.56 Å
b: 1.45 Å
c: 1.35 Å
d: 1.34 Å
e: 1.78 Å
f: 1.35 Å
g: 1.03 Å
h: 1.86 Å
i: 1.49 Å

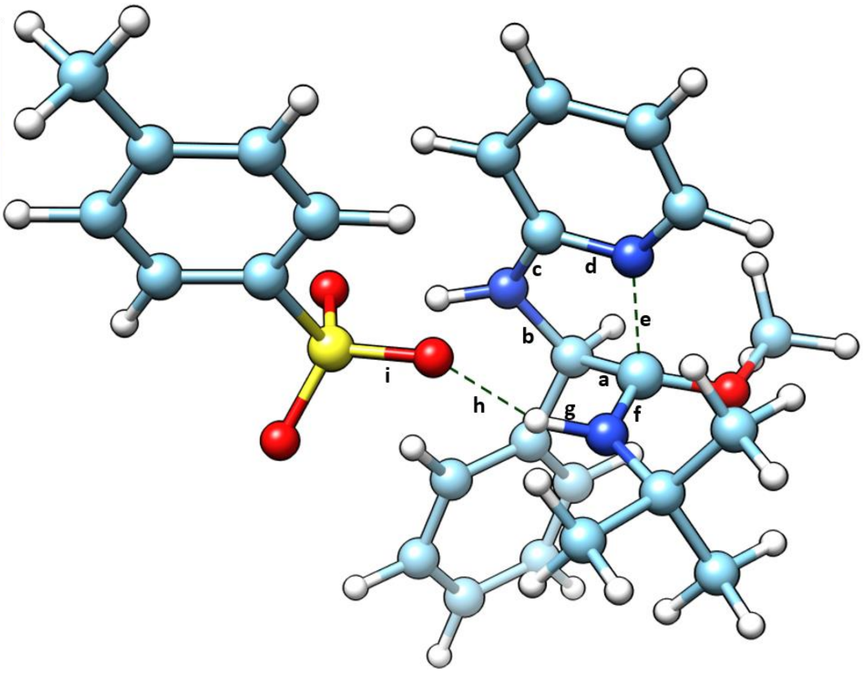

Figure S152. Molecular complex 6.

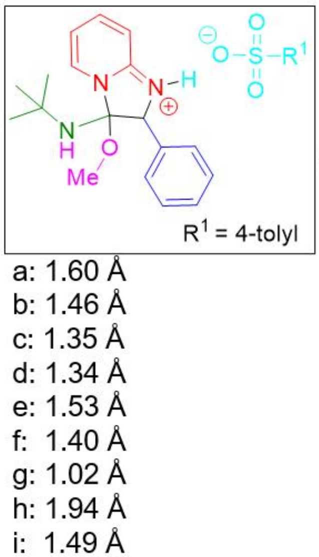

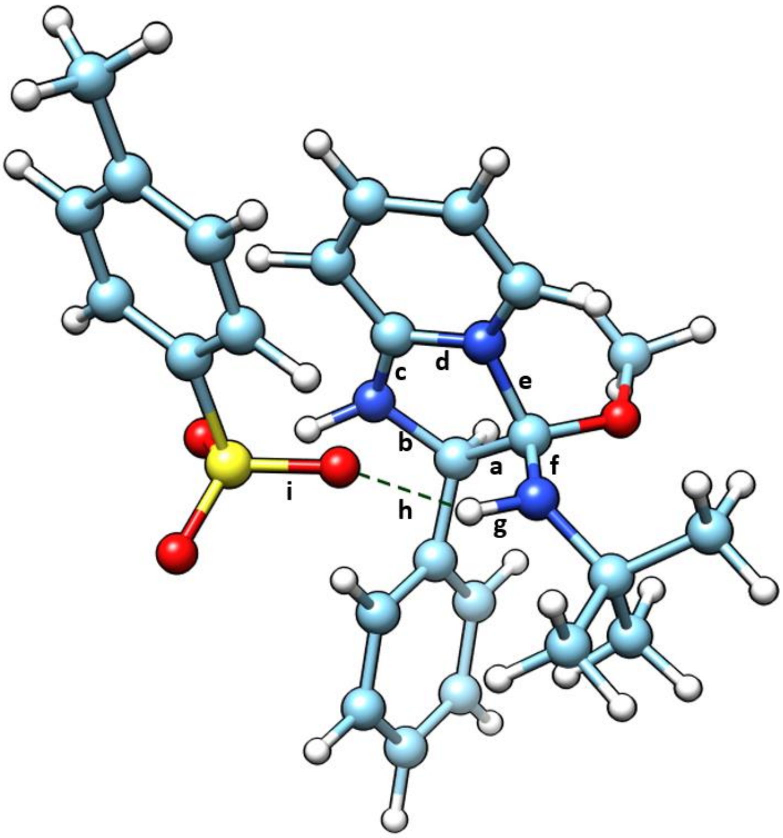

a: 1.60 Å
b: 1.46 Å
c: 1.35 Å
d: 1.34 Å
e: 1.53 Å
f: 1.40 Å
g: 1.02 Å
h: 1.94 Å
i: 1.49 Å

9.9.4. Step 4 – Methanol eliminination

Figure S153. Molecular complex 7.

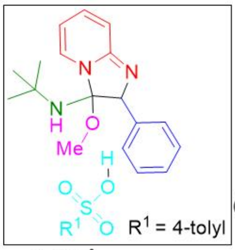

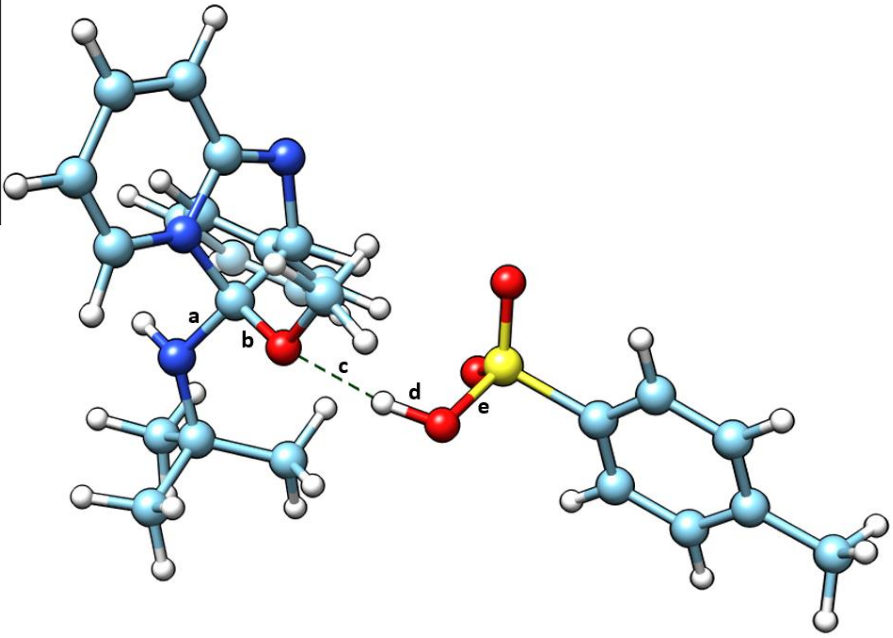

a: 1.43 Å
b: 1.43 Å
c: 1.83 Å
d: 0.99 Å
e: 1.60 Å

Figure S154. Transition State 4.

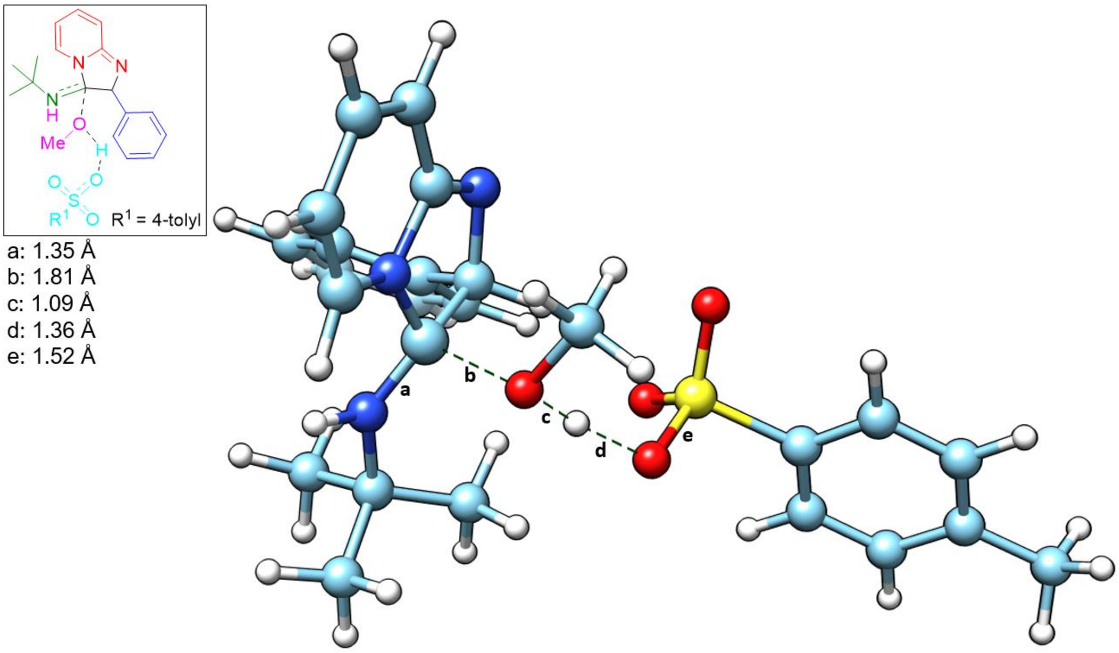

Figure S155. Molecular complex 8.

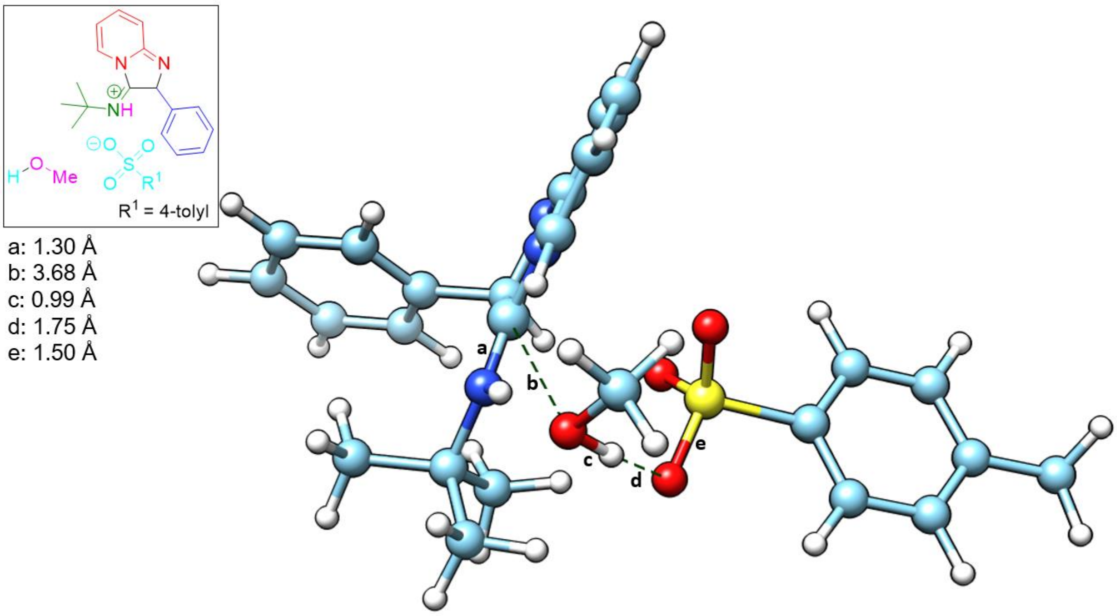

9.9.5. Step 5 – Proton abstraction by tosylate

Figure S156. Molecular complex 9.

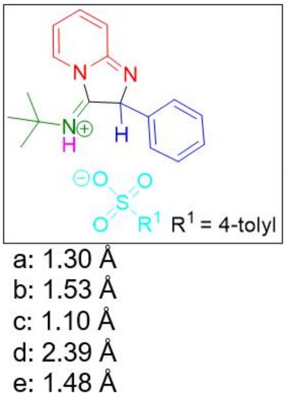

a: 1.30 Å
b: 1.53 Å
c: 1.10 Å
d: 2.39 Å
e: 1.48 Å

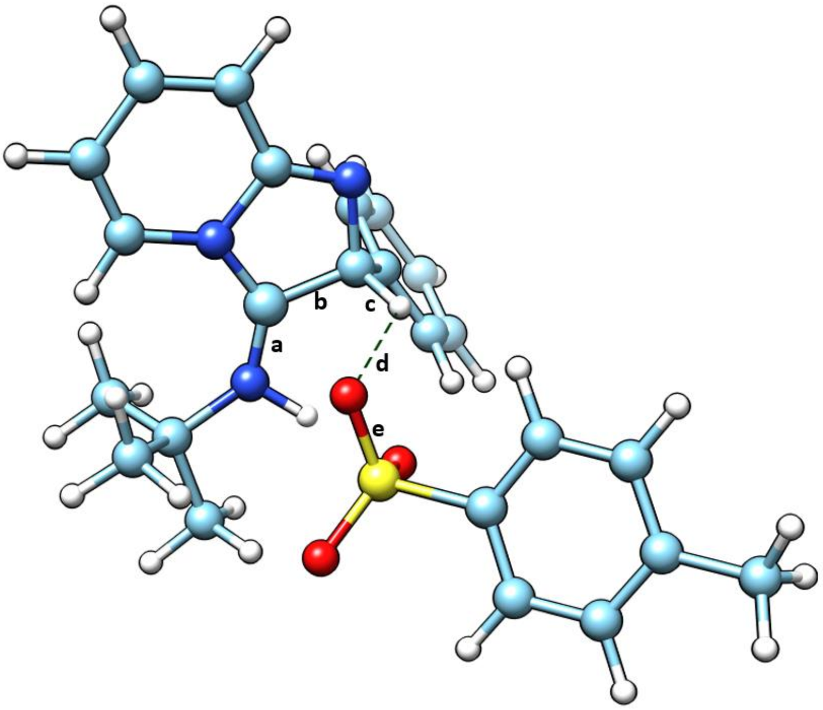

Figure S157. Transition State 5.

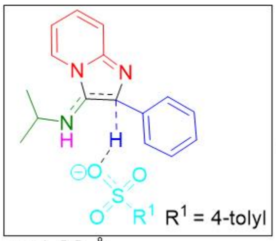

a: 1.32 Å
b: 1.46 Å
c: 1.29 Å
d: 1.36 Å
e: 1.52 Å

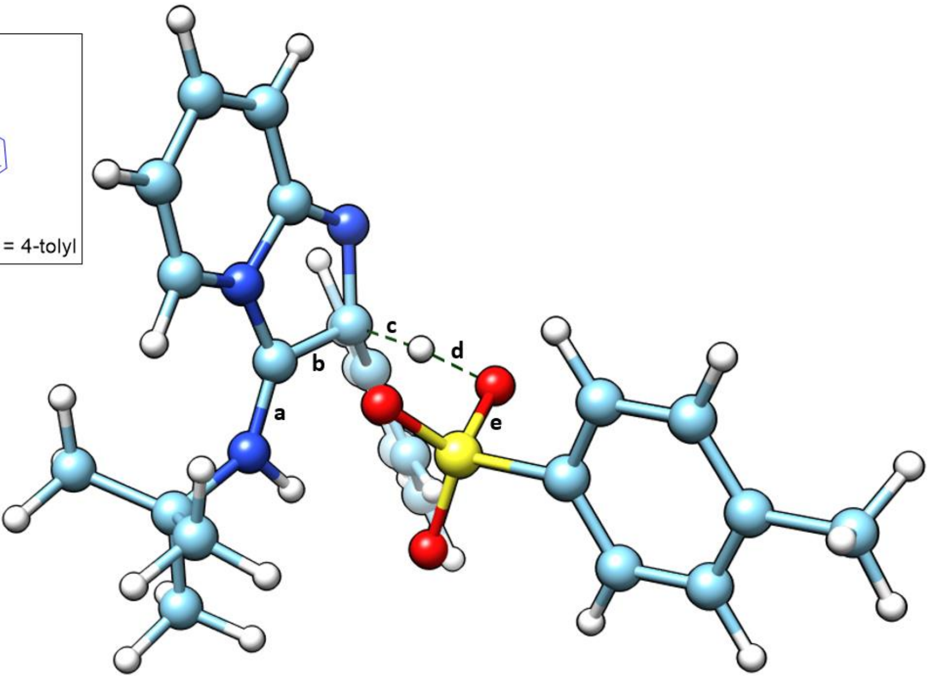

Figure S158. Molecular complex 10.

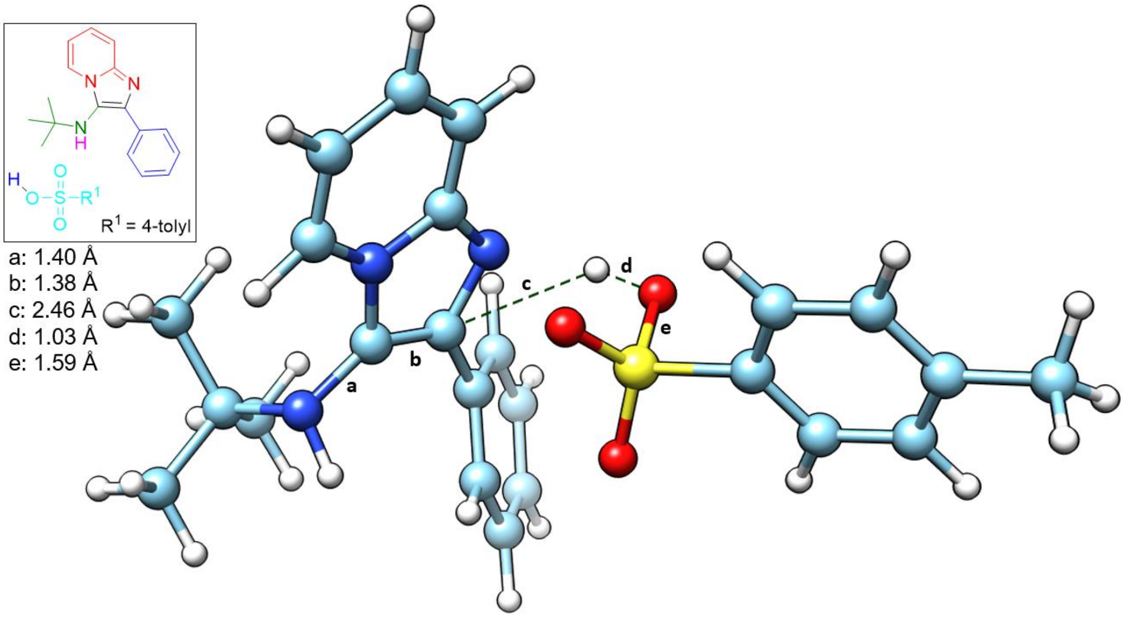

9.10. Proposal J: GBB reaction, methanol acts as a proton shuttle in the third step, catalyzed by *p*-toluenesulfonic acid

9.10.1. Step 1 - Isocyanide nucleophilic attack, forming nitrilium

Figure S159. Molecular complex 1.

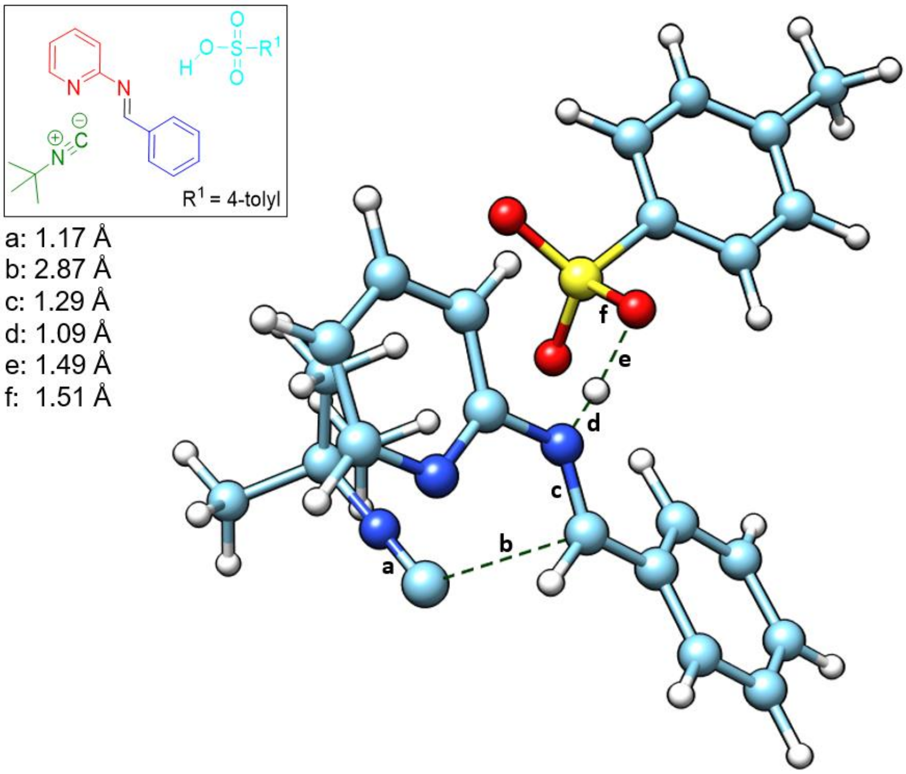

Figure S160. Transition State 1.

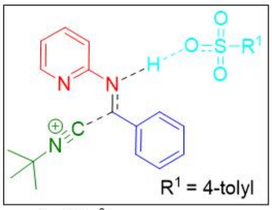

a: 1.16 Å
b: 2.00 Å
c: 1.33 Å
d: 1.05 Å
e: 1.70 Å
f: 1.50 Å

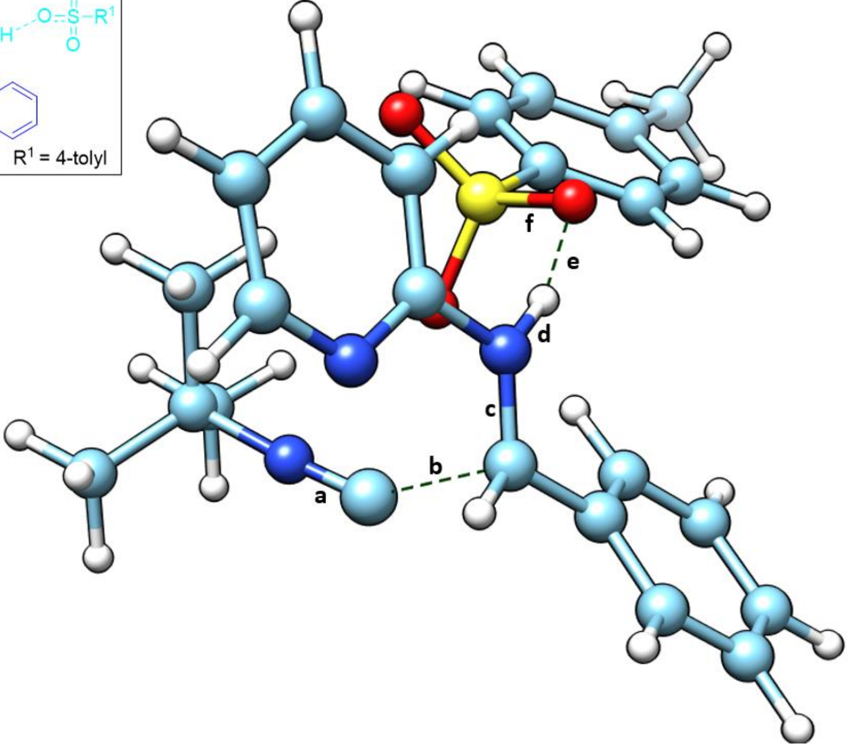

Figure S161. Molecular complex 2.

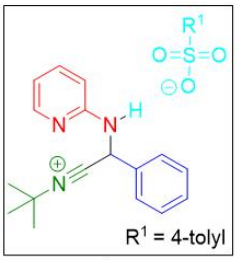

a: 1.15 Å
b: 1.51 Å
c: 1.44 Å
d: 1.03 Å
e: 1.84 Å
f: 1.49 Å

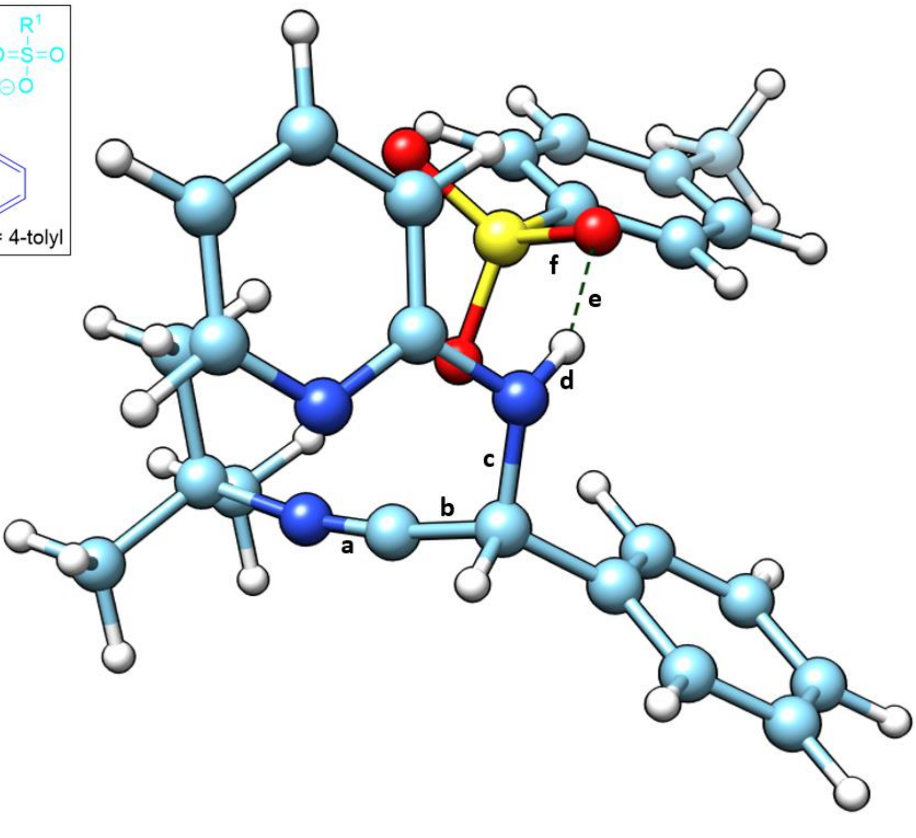

9.10.2. Step 2 – Ring Closure

Figure S162. Molecular complex 3.

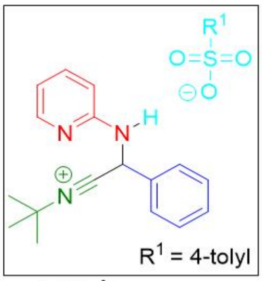

a: 1.15 Å
b: 2.86 Å
c: 1.33 Å
d: 1.39 Å
e: 1.43 Å
f: 1.51 Å
g: 1.03 Å
h: 1.87 Å

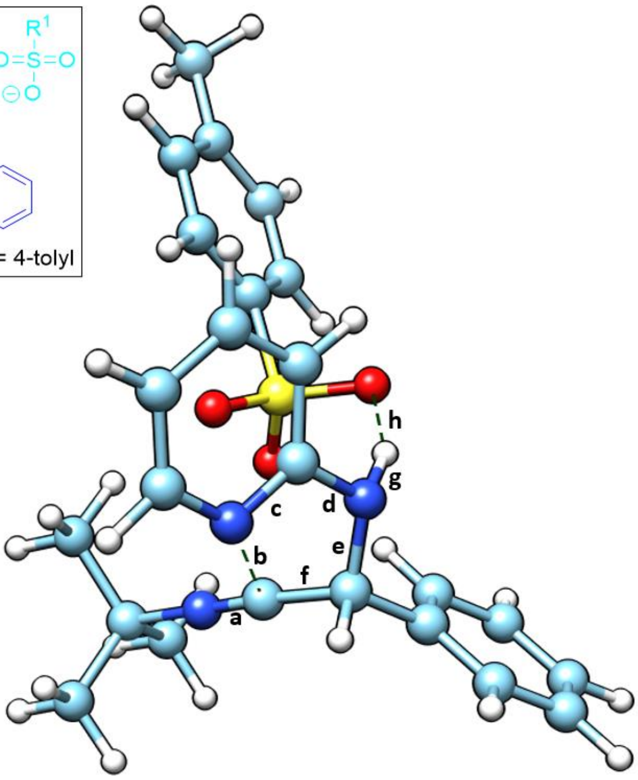

Figure S163. Transition State 2.

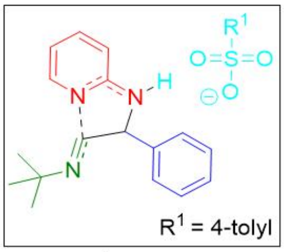

a: 1.17 Å
b: 2.01 Å
c: 1.33 Å
d: 1.39 Å
e: 1.45 Å
f: 1.51 Å
g: 1.03 Å
h: 1.96 Å

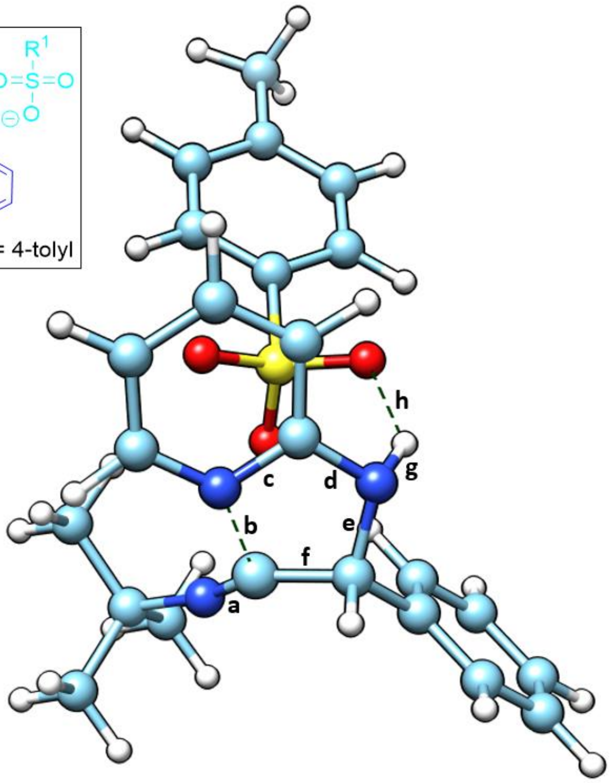

Figure S164. Molecular complex 4.

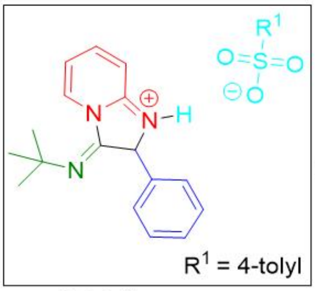

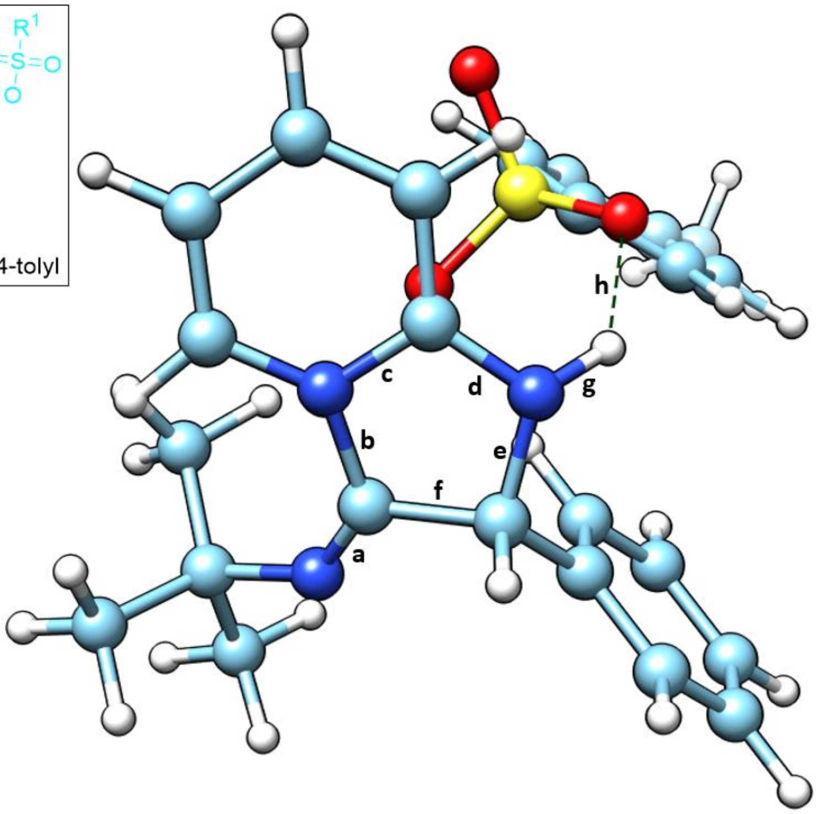

a: 1.24 Å
b: 1.47 Å
c: 1.36 Å
d: 1.34 Å
e: 1.47 Å
f: 1.53 Å
g: 1.03 Å
h: 1.91 Å

9.10.3. Step 3 – Methanol acting as a proton shuttle

Figure S165. Molecular complex 5.

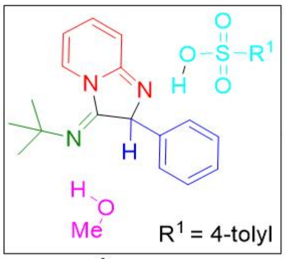

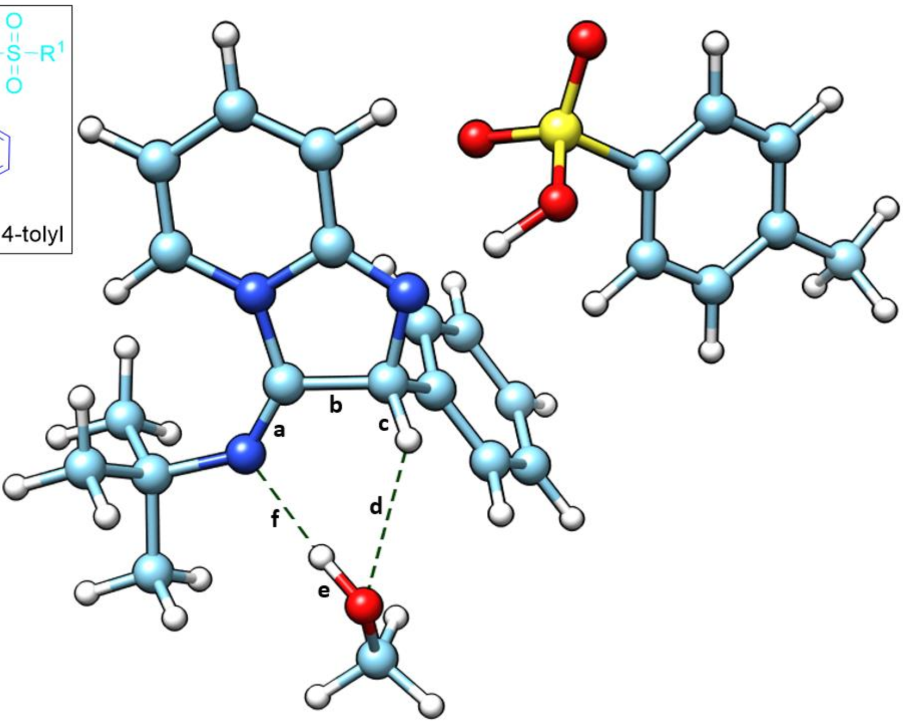

a: 1.26 Å
b: 1.54 Å
c: 1.10 Å
d: 2.63 Å
e: 0.98 Å
f: 1.91 Å

Figure S166. Transition State 3.

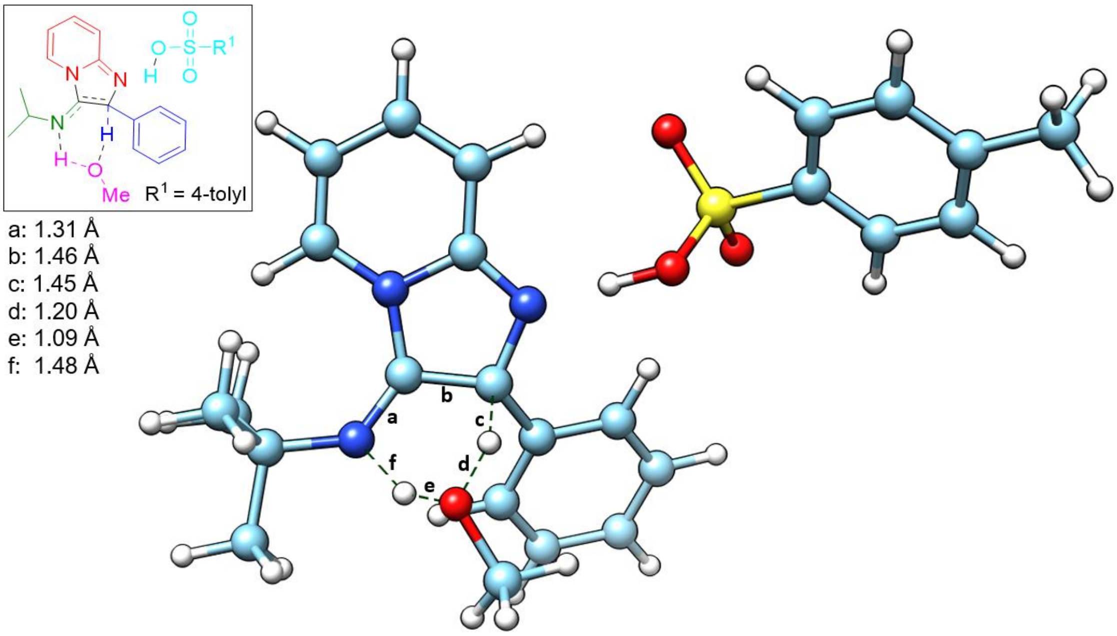

Figure S167. Molecular complex 6.

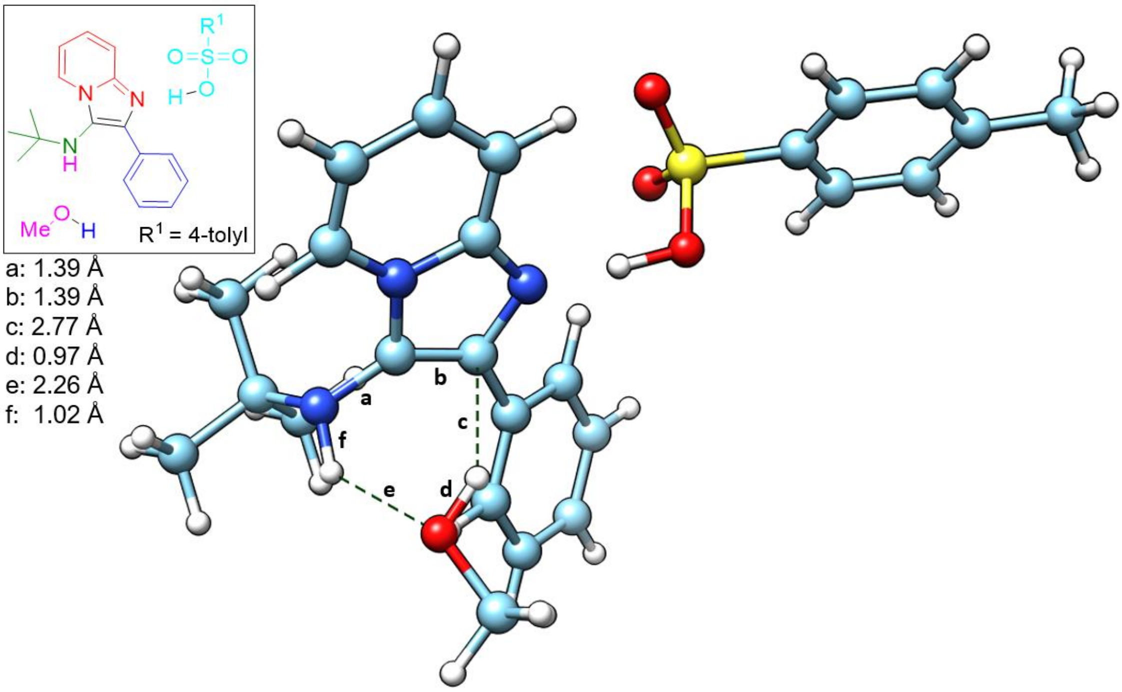

9.11. Proposal K: Methanol addition to imine, catalyzed by *p*-toluenesulfonic acid

9.11.1. Step 1 - Methanol nucleophilic attack to imine

Figure S168. Molecular complex 1.

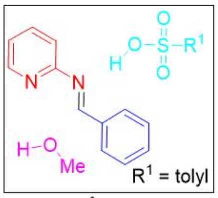

a: 2.62 Å
b: 1.29 Å
c: 1.09 Å
d: 1.50 Å
e: 1.52 Å
f: 1.48 Å
g: 1.89 Å
h: 0.97 Å

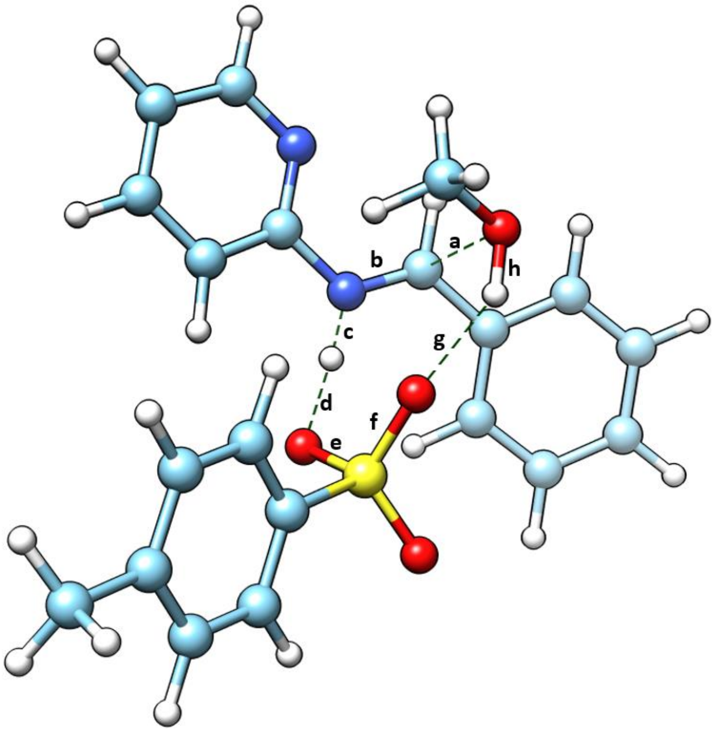

Figure S169. Transition State 1.

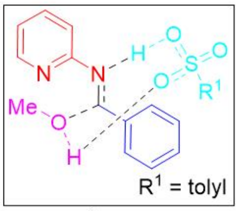

a: 1.97 Å
b: 1.33 Å
c: 1.04 Å
d: 1.69 Å
e: 1.50 Å
f: 1.50 Å
g: 1.53 Å
h: 1.02 Å

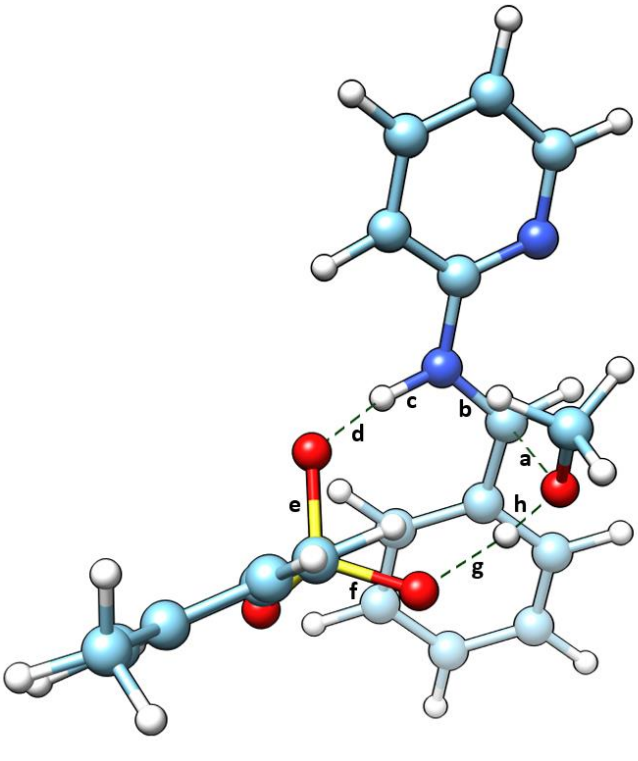

Figure S170. Molecular complex 2.

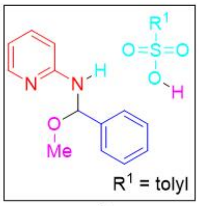

a: 1.45 Å
b: 1.42 Å
c: 1.02 Å
d: 1.99 Å
e: 1.47 Å
f: 1.58 Å
g: 1.02 Å
h: 1.55 Å

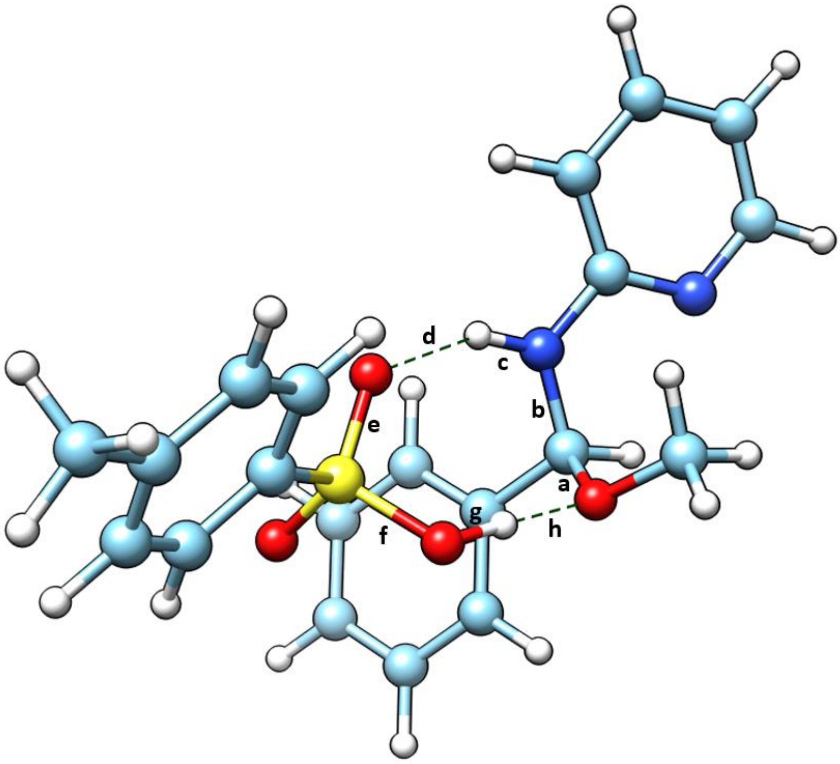

References

(1) Vidyacharan, S.; Shinde, A. H.; Satpathi, B.; Sharada, D. S. A Facile Protocol for the Synthesis of 3-Aminoimidazo-Fused Heterocycles via the Groebke–Blackburn–Bienayme Reaction under Catalyst-Free and Solvent-Free Conditions. *Green Chem.* **2014**, *16* (3), 1168. https://doi.org/10.1039/c3gc42130a.

(2) Budhiraja, M.; Kondabala, R.; Ali, A.; Tyagi, V. First Biocatalytic Groebke-Blackburn-Bienaymé Reaction to Synthesize Imidazo[1,2-a]Pyridine Derivatives Using Lipase Enzyme. *Tetrahedron* **2020**, *76* (47), 131643. https://doi.org/10.1016/j.tet.2020.131643.

(3) Singh, H. K.; Kamal, A.; Kumari, S.; Kumar, D.; Maury, S. K.; Srivastava, V.; Singh, S. Eosin Y-Catalyzed Synthesis of 3-Aminoimidazo[1,2-*a*]Pyridines via the HAT Process under Visible Light through Formation of the C–N Bond. *ACS Omega* **2020**, *5* (46), 29854–29863. https://doi.org/10.1021/acsomega.0c03941.

(4) Adiyala, P. R.; Chandrasekhar, D.; Kapure, J. S.; Reddy, C. N.; Maurya, R. A. Synthesis of α-Amino Amidines through Molecular Iodine-Catalyzed Three-Component Coupling of Isocyanides, Aldehydes and Amines. *Beilstein J. Org. Chem.* **2014**, *10*, 2065–2070. https://doi.org/10.3762/bjoc.10.214.

(5) Santos, G.; Anjos, N.; Gibeli, M.; Silva, G.; Fernandes, P.; Fiorentino, E.; Longo Jr., L. A Comparative Study on the Groebke-Blackburn-Bienaymé Three-Component Reaction Catalyzed by Rare Earth Triflates under Microwave Heating. *J. Braz. Chem. Soc.* **2020**. https://doi.org/10.21577/0103-5053.20200028.

(6) Mani, G. S.; Shaik, S. P.; Tangella, Y.; Bale, S.; Godugu, C.; Kamal, A. A Facile I2 - Catalyzed Synthesis of Imidazo[1,2-*a*]Pyridines via Sp 3 C–H Functionalization of Azaarenes and Evaluation of Anticancer Activity. *Org. Biomol. Chem.* **2017**, *15* (32), 6780–6791. https://doi.org/10.1039/C7OB01384A.

(7) Singh, H. K.; Kamal, A.; Kumari, S.; Maury, S. K.; Kushwaha, A. K.; Srivastava, V.; Singh, S. Visible-Light-Promoted Synthesis of Fusesd Imidazoheterocycle by Eosin Y under Metal-Free and Solvent-Free Conditions. *ChemistrySelect* **2021**, *6* (48), 13982–13991. https://doi.org/10.1002/slct.202103548.

(8) Hussain, M.; Liu, J.; Fu, L.; Hasan, M. Synthesis of Imidazo[1,2-a]Pyridines via the Silver Acetate-catalyzed Groebke-Blackburn-Bienayme Reaction with Ethylene Glycol as a Biodegradable and Sustainable Solvent. *J. Heterocycl. Chem.* **2020**, *57* (3), 955–964. https://doi.org/10.1002/jhet.3746.

(9) Sun, C.; Ji, S.; Liu, Y. A Novel, Simple and Efficient Synthesis of 3-Amino-Benzo[d]Imidazo[2,1-b]Thiazole Derivatives via a Multicomponent Procedure. *J. Chinese Chem. Soc.* **2008**, *55* (2), 292–296. https://doi.org/10.1002/jccs.200800043.

(10) Reutlinger, M.; Rodrigues, T.; Schneider, P.; Schneider, G. Combining On-Chip Synthesis of a Focused Combinatorial Library with Computational Target Prediction Reveals

Imidazopyridine GPCR Ligands. *Angew. Chemie Int. Ed.* **2014**, *53* (2), 582–585. https://doi.org/10.1002/anie.201307786.

(11) Frisch, M. J.; Trucks, G. W.; Schlegel, H. B.; Scuseria, G. E.; Robb, M. A.; Cheeseman, J. R.; Scalmani, G.; Barone, V.; Mennucci, B.; Petersson, G. A.; Nakatsuji, H.; Caricato, M.; Li, X.; Hratchian, H. P.; Izmaylov, A. F.; Bloino, J.; Zheng, G.; Sonnenberg, J. L.; Hada, M.; Ehara, M.; Toyota, K.; Fukuda, R.; Hasegawa, J.; Ishida, M.; Nakajima, T.; Honda, Y.; Kitao, O.; Nakai, H.; Vreven, T.; Montgomery, J. A.; Peralta, J. J. E.; Ogliaro, F.; Bearpark, M.; Heyd, J. J.; Brothers, E.; Kudin, K. N.; Staroverov, V. N.; Keith, T.; Kobayashi, R.; Normand, J.; Raghavachari, K.; Rendell, A.; Burant, J. C.; Iyengar, S. S.; Tomasi, J.; Cossi, M.; Rega, N.; Millam, J. M.; Klene, M.; Knox, J. E.; Cross, J. B.; Bakken, V.; Adamo, C.; Jaramillo, J.; Gomperts, R.; Stratmann, R. E.; Yazyev, O.; Austin, A. J.; Cammi, R.; Pomelli, C.; Ochterski, J. W.; Martin, R. L.; Morokuma, K.; Zakrzewski, V. G.; Voth, G. A.; Salvador, P.; Dannenberg, J. J.; Dapprich, S.; Daniels, A. D.; Farkas, O.; Foresman, J. B.; Ortiz, J. V.; Cioslowski, J.; D. J., F. Gaussian 09, Revision D.01. Gaussian, Inc.: Wallingford CT 2013.

(12) Carvalho, M. H. R.; Ribeiro, J. P. R. S.; De Castro, P. P.; Passos, S. T. A.; Neto, B. A. D.; Dos Santos, H. F.; Amarante, G. W. Solvent Dependent Competitive Mechanisms for the Ugi Multicomponent Reaction: A Joint Theoretical and Experimental Study in the α-Acyl Aminocarboxamides vs α-Amino Amidines Formation. *J. Org. Chem.* **2022**, *87* (16), 11007–11020. https://doi.org/10.1021/acs.joc.2c01272.

(13) Santos, W. A. B.; de Castro, P. P.; Xavier, F. R.; Braga, A. L.; Martins, G. M.; Mendes, S. R. Electrosynthesis of Flavanones via Oxa-Michael Addition Using Sacrificial Electrodes. *Synthesis (Stuttg).* **2023**, *55* (18), 2985–2992. https://doi.org/10.1055/a-2038-9146.
