# Supplementary material for: Solvents’ and Reagents’ Noninnocent Roles in the Groebke–Blackburn–Bienaymé (GBB) Multicomponent Reaction: Experimental and Computational Evidence
Source: ACS Org Inorg Au. 2025 Jun 12;5(4):288–98. doi: 10.1021/acsorginorgau.5c00049 (PMC12332792; doi:10.1021/acsorginorgau.5c00049)
Supplement: Supplementary file 2 [file gg5c00049_si_002.pdf]

Solvents' and Reagents' Noninnocent Roles in the Groebke–Blackburn–Bienaymé (GBB) Multicomponent Reaction: Experimental and Computational Evidence

Marcelo H. R. Carvalho,[‡] Pedro P. De Castro,[†,*] Pedro Beck,[§] Hélio F. Dos Santos,[‡] Fabricio Machado,[§] José R. Correa,[§] Brenno A. D. Neto,[§,*] and Giovanni W. Amarante[‡,*]

[‡] Chemistry Department, Federal University of Juiz de Fora, Campus Martelos, Juiz de Fora, Minas Gerais, 36036-900, Brazil.

[†] Pharmacy Department, Federal University of Juiz de Fora – Campus Governador Valadares, Governador Valadares, Minas Gerais, 35010-180, Brazil.

[§] Laboratory of Medicinal and Technological Chemistry, University of Brasília, Chemistry Institute (IQ-UnB), Campus Universitário Darcy Ribeiro, Brasília, Distrito Federal, 70910-900, Brazil.

* pedro.possa@ufjf.br; brenno.ipi@gmail.com, giovanni.amarante@ufjf.br

Supporting Information - Coordinates of Optimized Stationary Points

1. COORDINATES OF OPTIMIZED STATIONARY POINTS

1.1. Proposal A: Classic GBB reaction

1.1.1. Step 1 - Isocyanide nucleophilic attack, forming nitrilium

- Molecular complex 1

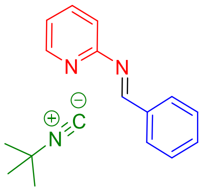

Symbolic Z-matrix:

Charge = 0 Multiplicity = 1

C	-0.33403	-1.2324	0.35932	C	-4.50063	0.42064	-0.97143
C	0.46501	1.36366	2.11397	H	-2.686	0.38412	-2.16475
N	0.92901	1.8087	1.13571	C	-5.06666	-0.01075	0.22679
C	1.51219	2.35791	-0.06581	H	-4.6812	-1.12754	2.03331
N	-1.1241	-0.9633	-0.608	H	-5.08159	1.00077	-1.68181
C	1.62943	3.87337	0.12343	H	-6.09548	0.21593	0.48286
H	2.07139	4.31709	-0.77331	N	-3.00369	-1.07918	0.84665
H	0.64392	4.31705	0.28705	H	-0.6718	-1.20384	1.40078
H	2.26589	4.10525	0.98142	C	1.08063	-1.55965	0.12017
C	0.58853	2.02162	-1.24008	C	1.95365	-1.6404	1.21032
H	0.48478	0.93955	-1.3577	C	1.57098	-1.76816	-1.17646
H	-0.40566	2.44823	-1.07783	C	3.30421	-1.91801	1.00985
H	1.00881	2.44235	-2.15848	H	1.57048	-1.46378	2.21208
C	2.89213	1.72071	-0.25488	C	2.918	-2.04925	-1.37438
H	2.8045	0.63664	-0.36512	H	0.87851	-1.70682	-2.01052
H	3.35697	2.13012	-1.15672	C	3.7872	-2.12217	-0.28199
H	3.53464	1.93705	0.60304	H	3.97815	-1.97189	1.85881
C	-2.46518	-0.6538	-0.29895	H	3.29511	-2.21389	-2.37894
C	-3.18119	0.08879	-1.24646	H	4.83892	-2.34053	-0.4399
C	-4.27409	-0.76037	1.09412				

- Transition state 1

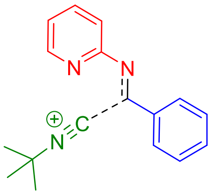

Symbolic Z-matrix:

Charge = 0 Multiplicity = 1

C	-0.98735	-0.58501	-0.55314	C	3.07865	-2.64118	0.71924
C	-0.29978	0.93852	-0.54939	H	1.26958	-2.69768	1.8984
N	0.53241	1.67094	-0.2089	C	3.65837	-2.1608	-0.46895
C	1.72129	2.33182	0.29435	H	3.25382	-1.02647	-2.24916
N	-0.27797	-1.26165	0.39904	H	3.66559	-3.23362	1.41568
C	2.66852	2.51662	-0.89326	H	4.69148	-2.36497	-0.72511
H	3.59521	2.97501	-0.53746	N	1.57049	-1.11048	-1.06643
H	2.89876	1.54665	-1.3412	H	-0.80299	-0.85326	-1.60468
H	2.22142	3.16637	-1.65014	C	-2.46064	-0.39342	-0.26582
C	2.3341	1.40476	1.3513	C	-3.28196	0.19984	-1.2263
H	1.60173	1.16406	2.12649	C	-3.00568	-0.82206	0.94236
H	2.67952	0.47311	0.89464	C	-4.64152	0.37017	-0.98132
H	3.18581	1.91439	1.81155	H	-2.85192	0.5286	-2.17075
C	1.29824	3.67233	0.89672	C	-4.3684	-0.65195	1.1877
H	0.60746	3.52095	1.73006	H	-2.34783	-1.29359	1.66423
H	2.18644	4.19012	1.2679	C	-5.18717	-0.05597	0.23049
H	0.81489	4.30108	0.14459	H	-5.27516	0.82917	-1.73382
C	1.00514	-1.574	0.07448	H	-4.79208	-0.99025	2.1284
C	1.76137	-2.35394	0.99509	H	-6.24777	0.07236	0.42355
C	2.85208	-1.41468	-1.31342				

- Molecular complex 2

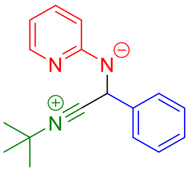

Symbolic Z-matrix:

Charge = 0 Multiplicity = 1

C	-0.92653	-0.59806	-0.58246	N	0.42566	1.64131	-0.17274
C	-0.26468	0.77003	-0.49059	C	1.56299	2.40667	0.31723

N	-0.22198	-1.35551	0.37738
C	2.4926	2.6265	-0.87812
H	3.38319	3.16037	-0.53589
H	2.79242	1.66362	-1.29946
H	1.99988	3.22232	-1.65087
C	2.23518	1.54313	1.39118
H	1.52922	1.29847	2.18906
H	2.61119	0.61214	0.95669
H	3.07203	2.10486	1.81643
C	1.04195	3.72507	0.88662
H	0.35843	3.54463	1.7201
H	1.88949	4.31129	1.25076
H	0.52162	4.30286	0.11844
C	1.06948	-1.59397	0.06637
C	1.84899	-2.39866	0.95502
C	2.9373	-1.29344	-1.28308
C	3.17693	-2.61882	0.68336
H	1.35992	-2.81358	1.82953
C	3.75896	-2.05377	-0.47024
H	3.33665	-0.84114	-2.19115
H	3.77567	-3.22739	1.35585
H	4.80175	-2.20832	-0.72095
N	1.64057	-1.04787	-1.04116
H	-0.77017	-0.89892	-1.63292
C	-2.4114	-0.43946	-0.28775
C	-3.2328	0.22089	-1.20367
C	-2.95402	-0.96082	0.8838
C	-4.5938	0.36561	-0.94904
H	-2.80616	0.61634	-2.12395
C	-4.31897	-0.81808	1.13604
H	-2.29178	-1.47869	1.56871
C	-5.13901	-0.1551	0.22525
H	-5.22849	0.87632	-1.66648
H	-4.74284	-1.2291	2.04718
H	-6.2007	-0.04784	0.42491

1.1.2. Step 2 – Ring closure

- Molecular complex 3

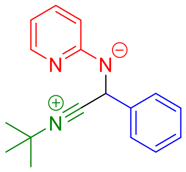

Symbolic Z-matrix:

Charge = 0 Multiplicity = 1

C	-0.92666	-0.59782	-0.58222
C	-0.26477	0.77042	-0.4904
N	0.42551	1.64177	-0.17251
C	1.56348	2.40656	0.3171
N	-0.22212	-1.35518	0.37757
C	2.49285	2.6256	-0.87856
H	3.38402	3.15872	-0.53662
H	2.79171	1.66246	-1.29998
H	2.00037	3.22185	-1.65113
C	2.23545	1.54288	1.39105
H	1.52944	1.29839	2.18895
H	2.61125	0.61179	0.95661
H	3.07242	2.10444	1.81629
C	1.04334	3.72537	0.88637
H	0.36019	3.54549	1.72027
H	1.89133	4.31135	1.24985
H	0.52284	4.30313	0.11828
C	1.06931	-1.59388	0.06649

C	1.84862	-2.39888	0.95502	C	-2.95408	-0.95987	0.88427
C	2.93712	-1.29367	-1.283	C	-4.59405	0.36505	-0.94948
C	3.17651	-2.6193	0.68331	H	-2.80646	0.6151	-2.12461
H	1.35943	-2.81384	1.82944	C	-4.31902	-0.81705	1.13648
C	3.75862	-2.05426	-0.47025	H	-2.29181	-1.47725	1.56953
H	3.33655	-0.84141	-2.19106	C	-5.13917	-0.15482	0.22523
H	3.77513	-3.22812	1.35568	H	-5.2288	0.87515	-1.66729
H	4.80136	-2.20901	-0.72103	H	-4.74281	-1.22744	2.04794
N	1.64045	-1.0478	-1.04099	H	-6.20086	-0.04753	0.42485
C	-2.41151	-0.43931	-0.28767	H	-0.77006	-0.89852	-1.6327
C	-3.23304	0.22027	-1.20403				

- Transition state 2

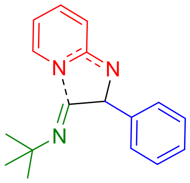

Symbolic Z-matrix:

Charge = 0 Multiplicity = 1

C	-0.75635	-0.86588	-0.66103	C	2.11499	-2.60692	0.75637
C	-0.10472	0.48324	-0.50029	C	3.12232	-0.77949	-1.01258
N	0.17479	1.54263	-0.11452	C	3.46979	-2.52406	0.57773
C	1.07024	2.60681	0.32053	H	1.66079	-3.31032	1.4454
N	-0.07106	-1.75679	0.21613	C	4.01215	-1.58404	-0.33266
C	1.92225	2.99593	-0.88978	H	3.48466	-0.04902	-1.73611
H	2.62921	3.77398	-0.58928	H	4.13073	-3.18265	1.13491
H	2.47964	2.12874	-1.25189	H	5.07866	-1.50416	-0.50525
H	1.29623	3.38345	-1.69778	N	1.78715	-0.83721	-0.86165
C	1.93308	2.01089	1.43801	C	-2.23373	-0.69457	-0.33099
H	1.30942	1.69022	2.27629	C	-3.07739	-0.0503	-1.23796
H	2.49804	1.15036	1.06783	C	-2.74364	-1.17315	0.8741
H	2.63123	2.7757	1.78984	C	-4.42716	0.12159	-0.9415
C	0.22399	3.77686	0.81731	H	-2.67834	0.31043	-2.18431
H	-0.40581	3.47023	1.65604	C	-4.09636	-1.00318	1.16818
H	0.88677	4.57912	1.15202	H	-2.06701	-1.68671	1.54846
H	-0.41492	4.16157	0.01822	C	-4.93833	-0.35515	0.26583
C	1.24977	-1.73135	0.01881	H	-5.07962	0.61849	-1.65269

H	-4.49491	-1.38326	2.10387	H	-0.6352	-1.10274	-1.73365
H	-5.99114	-0.22736	0.49783				

- Molecular complex 4

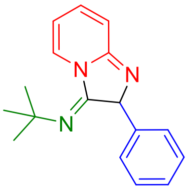

Symbolic Z-matrix:

Charge = 0 Multiplicity = 1

C	-0.65293	-0.66141	-1.19445	C	3.01803	-2.87686	0.42934
C	0.40263	0.24212	-0.52806	H	1.60307	-4.04136	-0.68729
N	0.26888	1.49338	-0.50199	C	3.35036	-1.56907	0.93791
C	1.08285	2.57452	0.05015	H	2.7387	0.47235	1.02688
N	-0.11337	-2.01001	-1.19827	H	3.68272	-3.70935	0.63743
C	2.47887	2.62403	-0.59158	H	4.23968	-1.41143	1.5339
H	3.03697	3.47975	-0.19818	N	1.39358	-0.68984	-0.08296
H	3.07539	1.72595	-0.42739	C	-1.9929	-0.5575	-0.47992
H	2.37481	2.75416	-1.67281	C	-2.74946	0.61269	-0.60329
C	1.1133	2.51128	1.58572	C	-2.47034	-1.60129	0.31229
H	0.08963	2.55928	1.96816	C	-3.96437	0.73709	0.06447
H	1.56272	1.59938	1.98156	H	-2.372	1.42739	-1.21406
H	1.6694	3.36676	1.98235	C	-3.68865	-1.47437	0.98135
C	0.35105	3.86886	-0.33925	H	-1.89107	-2.51563	0.38662
H	-0.66269	3.86136	0.07105	C	-4.4376	-0.30686	0.8609
H	0.88237	4.74702	0.04185	H	-4.54576	1.64822	-0.04007
H	0.27746	3.94511	-1.4277	H	-4.0532	-2.29389	1.59332
C	1.00035	-1.96692	-0.56406	H	-5.38709	-0.21076	1.3785
C	1.89238	-3.07462	-0.29332	H	-0.78305	-0.31178	-2.22562
C	2.53613	-0.52291	0.66748				

1.1.3. Step 3 – 1,3-Intramolecular hydrogen shift

- Molecular complex 5

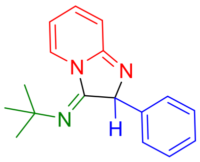

Symbolic Z-matrix:

Charge = 0 Multiplicity = 1

C	-0.65272	-0.66138	-1.19512	C	3.01817	-2.8763	0.42949
C	0.40252	0.24227	-0.52842	H	1.60397	-4.04088	-0.688
N	0.26856	1.49352	-0.50248	C	3.35002	-1.56855	0.93845
C	1.08214	2.57463	0.05021	H	2.73799	0.47274	1.02753
N	-0.1128	-2.00985	-1.19916	H	3.68295	-3.7087	0.63767
C	2.47851	2.62428	-0.59079	H	4.2391	-1.41081	1.53477
H	3.03685	3.47927	-0.19617	N	1.39344	-0.68952	-0.08298
H	3.07445	1.72567	-0.42743	C	-1.99254	-0.55788	-0.48028
H	2.37499	2.75568	-1.67192	C	-2.74981	0.61177	-0.60408
C	1.11174	2.51131	1.58582	C	-2.46903	-1.6014	0.31289
H	0.0878	2.55875	1.96762	C	-3.96449	0.73595	0.06415
H	1.56141	1.59966	1.98191	H	-2.37313	1.42629	-1.21559
H	1.66715	3.36708	1.98276	C	-3.68709	-1.47471	0.98242
C	0.35051	3.86901	-0.3394	H	-1.88924	-2.51539	0.38758
H	-0.66329	3.86164	0.07076	C	-4.43677	-0.3077	0.8615
H	0.88185	4.74715	0.0417	H	-4.54642	1.64669	-0.04078
H	0.27708	3.94519	-1.42786	H	-4.05091	-2.29403	1.5951
C	1.00067	-1.96661	-0.56452	H	-5.38607	-0.21178	1.37947
C	1.89287	-3.07416	-0.29368	H	-0.78303	-0.31155	-2.22616
C	2.53567	-0.5225	0.66793				

- Transition state 3

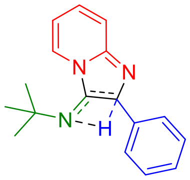

Symbolic Z-matrix:

Charge = 0 Multiplicity = 1

C	-0.55455	-0.69949	-0.72751	C	3.30183	-2.79973	0.39836
C	0.51939	0.14588	-0.2782	H	1.76273	-4.04508	-0.43105
N	0.40146	1.37983	-0.75097	C	3.68835	-1.46004	0.76742
C	0.89515	2.63641	-0.16789	H	3.05223	0.58886	0.86377
N	-0.04068	-2.01628	-0.85279	H	4.00782	-3.60986	0.54663
C	2.27858	2.95345	-0.75412	H	4.67576	-1.26046	1.16412
H	2.63789	3.91818	-0.38106	N	1.54815	-0.68289	0.1296
H	3.0178	2.19067	-0.49452	C	-1.96741	-0.58592	-0.23616
H	2.21723	2.99944	-1.84464	C	-2.4552	0.53621	0.44614
C	0.94046	2.5854	1.3644	C	-2.84405	-1.6479	-0.4917
H	-0.05636	2.39132	1.77224	C	-3.78156	0.59035	0.86509
H	1.60828	1.80083	1.73314	H	-1.79573	1.37381	0.6479
H	1.29503	3.53978	1.76678	C	-4.17198	-1.58497	-0.07671
C	-0.09073	3.72258	-0.61	H	-2.46661	-2.52404	-1.00728
H	-1.09057	3.52033	-0.21431	C	-4.65112	-0.46763	0.60431
H	0.2361	4.70377	-0.25184	H	-4.13543	1.46643	1.40065
H	-0.15522	3.74854	-1.70124	H	-4.83421	-2.42015	-0.28479
C	1.14068	-1.98202	-0.32384	H	-5.68528	-0.42192	0.92995
C	2.08082	-3.05279	-0.13408	H	-0.54035	0.55344	-1.43622
C	2.81907	-0.43619	0.61134				

- Molecular complex 6

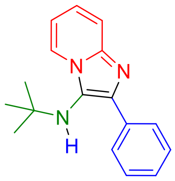

Symbolic Z-matrix:

Charge = 0 Multiplicity = 1

C	-0.29429	-0.91802	-0.1544	C	-1.20806	2.19313	1.02205
C	0.54467	0.14787	-0.46484	H	-2.0473	2.22565	0.31943
N	0.3842	1.50144	-0.79336	H	-1.20178	1.20534	1.49298
C	0.11887	2.47146	0.30585	H	-1.38157	2.94253	1.80152
N	0.41761	-2.03432	0.18082	C	0.07904	3.85121	-0.35036
C	1.26854	2.40438	1.31161	H	-0.73543	3.9111	-1.08188
H	1.08655	3.10768	2.12953	H	-0.09107	4.62709	0.40171
H	1.35716	1.40194	1.74393	H	1.02185	4.05832	-0.86504
H	2.21909	2.66734	0.8382	C	1.69237	-1.69341	0.07077

C	2.87371	-2.44824	0.29009
C	3.04073	0.22116	-0.56328
C	4.09071	-1.8603	0.08227
H	2.76677	-3.47642	0.61484
C	4.17471	-0.50443	-0.35919
H	3.00232	1.24609	-0.91057
H	5.00273	-2.42399	0.24558
H	5.13685	-0.04215	-0.54276
N	1.82492	-0.36939	-0.3366
C	-1.76728	-0.94139	-0.15439
C	-2.51424	-0.24485	-1.11061
C	-2.44149	-1.68557	0.82107
C	-3.90654	-0.26484	-1.07592
H	-2.00544	0.29404	-1.90492
C	-3.83267	-1.70608	0.85549
H	-1.85652	-2.24433	1.54485
C	-4.57032	-0.9899	-0.08766
H	-4.47276	0.27618	-1.82792
H	-4.34392	-2.28251	1.62036
H	-5.65528	-1.00615	-0.05948
H	-0.28016	1.63517	-1.54773

1.2. Proposal B: GBB reaction, aminopyridine acts as a proton shuttle in the third step

1.2.1. Step 1 - Isocyanide nucleophilic attack, forming nitrilium

- Molecular complex 1

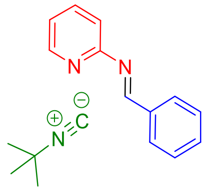

Symbolic Z-matrix:

Charge = 0 Multiplicity = 1

C	-0.33403	-1.2324	0.35932
C	0.46501	1.36366	2.11397
N	0.92901	1.8087	1.13571
C	1.51219	2.35791	-0.06581
N	-1.1241	-0.9633	-0.608
C	1.62943	3.87337	0.12343
H	2.07139	4.31709	-0.77331
H	0.64392	4.31705	0.28705
H	2.26589	4.10525	0.98142
C	0.58853	2.02162	-1.24008
H	0.48478	0.93955	-1.3577
H	-0.40566	2.44823	-1.07783
H	1.00881	2.44235	-2.15848
C	2.89213	1.72071	-0.25488
H	2.8045	0.63664	-0.36512
H	3.35697	2.13012	-1.15672
H	3.53464	1.93705	0.60304
C	-2.46518	-0.6538	-0.29895
C	-3.18119	0.08879	-1.24646
C	-4.27409	-0.76037	1.09412
C	-4.50063	0.42064	-0.97143
H	-2.686	0.38412	-2.16475
C	-5.06666	-0.01075	0.22679
H	-4.6812	-1.12754	2.03331
H	-5.08159	1.00077	-1.68181
H	-6.09548	0.21593	0.48286

N	-3.00369	-1.07918	0.84665	C	2.918	-2.04925	-1.37438
H	-0.6718	-1.20384	1.40078	H	0.87851	-1.70682	-2.01052
C	1.08063	-1.55965	0.12017	C	3.7872	-2.12217	-0.28199
C	1.95365	-1.6404	1.21032	H	3.97815	-1.97189	1.85881
C	1.57098	-1.76816	-1.17646	H	3.29511	-2.21389	-2.37894
C	3.30421	-1.91801	1.00985	H	4.83892	-2.34053	-0.4399
H	1.57048	-1.46378	2.21208				

- Transition state 1

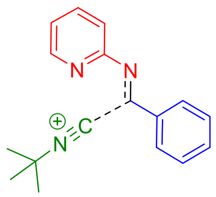

Symbolic Z-matrix:

Charge = 0 Multiplicity = 1

C	-0.98735	-0.58501	-0.55314	C	3.07865	-2.64118	0.71924
C	-0.29978	0.93852	-0.54939	H	1.26958	-2.69768	1.8984
N	0.53241	1.67094	-0.2089	C	3.65837	-2.1608	-0.46895
C	1.72129	2.33182	0.29435	H	3.25382	-1.02647	-2.24916
N	-0.27797	-1.26165	0.39904	H	3.66559	-3.23362	1.41568
C	2.66852	2.51662	-0.89326	H	4.69148	-2.36497	-0.72511
H	3.59521	2.97501	-0.53746	N	1.57049	-1.11048	-1.06643
H	2.89876	1.54665	-1.3412	H	-0.80299	-0.85326	-1.60468
H	2.22142	3.16637	-1.65014	C	-2.46064	-0.39342	-0.26582
C	2.3341	1.40476	1.3513	C	-3.28196	0.19984	-1.2263
H	1.60173	1.16406	2.12649	C	-3.00568	-0.82206	0.94236
H	2.67952	0.47311	0.89464	C	-4.64152	0.37017	-0.98132
H	3.18581	1.91439	1.81155	H	-2.85192	0.5286	-2.17075
C	1.29824	3.67233	0.89672	C	-4.3684	-0.65195	1.1877
H	0.60746	3.52095	1.73006	H	-2.34783	-1.29359	1.66423
H	2.18644	4.19012	1.2679	C	-5.18717	-0.05597	0.23049
H	0.81489	4.30108	0.14459	H	-5.27516	0.82917	-1.73382
C	1.00514	-1.574	0.07448	H	-4.79208	-0.99025	2.1284
C	1.76137	-2.35394	0.99509	H	-6.24777	0.07236	0.42355
C	2.85208	-1.41468	-1.31342				

- Molecular complex 2

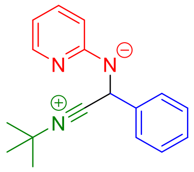

Symbolic Z-matrix:

Charge = 0 Multiplic ity = 1

C	-0.92653	-0.59806	-0.58246	C	3.17693	-2.61882	0.68336
C	-0.26468	0.77003	-0.49059	H	1.35992	-2.81358	1.82953
N	0.42566	1.64131	-0.17274	C	3.75896	-2.05377	-0.47024
C	1.56299	2.40667	0.31723	H	3.33665	-0.84114	-2.19115
N	-0.22198	-1.35551	0.37738	H	3.77567	-3.22739	1.35585
C	2.4926	2.6265	-0.87812	H	4.80175	-2.20832	-0.72095
H	3.38319	3.16037	-0.53589	N	1.64057	-1.04787	-1.04116
H	2.79242	1.66362	-1.29946	H	-0.77017	-0.89892	-1.63292
H	1.99988	3.22232	-1.65087	C	-2.4114	-0.43946	-0.28775
C	2.23518	1.54313	1.39118	C	-3.2328	0.22089	-1.20367
H	1.52922	1.29847	2.18906	C	-2.95402	-0.96082	0.8838
H	2.61119	0.61214	0.95669	C	-4.5938	0.36561	-0.94904
H	3.07203	2.10486	1.81643	H	-2.80616	0.61634	-2.12395
C	1.04195	3.72507	0.88662	C	-4.31897	-0.81808	1.13604
H	0.35843	3.54463	1.7201	H	-2.29178	-1.47869	1.56871
H	1.88949	4.31129	1.25076	C	-5.13901	-0.1551	0.22525
H	0.52162	4.30286	0.11844	H	-5.22849	0.87632	-1.66648
C	1.06948	-1.59397	0.06637	H	-4.74284	-1.2291	2.04718
C	1.84899	-2.39866	0.95502	H	-6.2007	-0.04784	0.42491
C	2.9373	-1.29344	-1.28308				

1.2.2. Step 2 – Ring closure

- Molecular complex 3

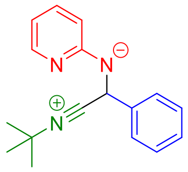

Symbolic Z-matrix:

Charge = 0 Multiplic ity = 1

C	-0.92666	-0.59782	-0.58222
C	-0.26477	0.77042	-0.4904
N	0.42551	1.64177	-0.17251
C	1.56348	2.40656	0.3171
N	-0.22212	-1.35518	0.37757
C	2.49285	2.6256	-0.87856
H	3.38402	3.15872	-0.53662
H	2.79171	1.66246	-1.29998
H	2.00037	3.22185	-1.65113
C	2.23545	1.54288	1.39105
H	1.52944	1.29839	2.18895
H	2.61125	0.61179	0.95661
H	3.07242	2.10444	1.81629
C	1.04334	3.72537	0.88637
H	0.36019	3.54549	1.72027
H	1.89133	4.31135	1.24985
H	0.52284	4.30313	0.11828
C	1.06931	-1.59388	0.06649
C	1.84862	-2.39888	0.95502
C	2.93712	-1.29367	-1.283
C	3.17651	-2.6193	0.68331
H	1.35943	-2.81384	1.82944
C	3.75862	-2.05426	-0.47025
H	3.33655	-0.84141	-2.19106
H	3.77513	-3.22812	1.35568
H	4.80136	-2.20901	-0.72103
N	1.64045	-1.0478	-1.04099
C	-2.41151	-0.43931	-0.28767
C	-3.23304	0.22027	-1.20403
C	-2.95408	-0.95987	0.88427
C	-4.59405	0.36505	-0.94948
H	-2.80646	0.6151	-2.12461
C	-4.31902	-0.81705	1.13648
H	-2.29181	-1.47725	1.56953
C	-5.13917	-0.15482	0.22523
H	-5.2288	0.87515	-1.66729
H	-4.74281	-1.22744	2.04794
H	-6.20086	-0.04753	0.42485
H	-0.77006	-0.89852	-1.6327

- Transition state 2

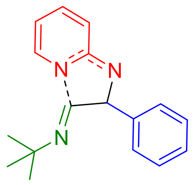

Symbolic Z-matrix:

Charge = 0 Multiplic ity = 1

C	-0.75635	-0.86588	-0.66103
C	-0.10472	0.48324	-0.50029
N	0.17479	1.54263	-0.11452
C	1.07024	2.60681	0.32053
N	-0.07106	-1.75679	0.21613
C	1.92225	2.99593	-0.88978
H	2.62921	3.77398	-0.58928
H	2.47964	2.12874	-1.25189
H	1.29623	3.38345	-1.69778
C	1.93308	2.01089	1.43801
H	1.30942	1.69022	2.27629
H	2.49804	1.15036	1.06783
H	2.63123	2.7757	1.78984
C	0.22399	3.77686	0.81731
H	-0.40581	3.47023	1.65604
H	0.88677	4.57912	1.15202

H	-0.41492	4.16157	0.01822
C	1.24977	-1.73135	0.01881
C	2.11499	-2.60692	0.75637
C	3.12232	-0.77949	-1.01258
C	3.46979	-2.52406	0.57773
H	1.66079	-3.31032	1.4454
C	4.01215	-1.58404	-0.33266
H	3.48466	-0.04902	-1.73611
H	4.13073	-3.18265	1.13491
H	5.07866	-1.50416	-0.50525
N	1.78715	-0.83721	-0.86165
C	-2.23373	-0.69457	-0.33099
C	-3.07739	-0.0503	-1.23796
C	-2.74364	-1.17315	0.8741
C	-4.42716	0.12159	-0.9415
H	-2.67834	0.31043	-2.18431
C	-4.09636	-1.00318	1.16818
H	-2.06701	-1.68671	1.54846
C	-4.93833	-0.35515	0.26583
H	-5.07962	0.61849	-1.65269
H	-4.49491	-1.38326	2.10387
H	-5.99114	-0.22736	0.49783
H	-0.6352	-1.10274	-1.73365

- Molecular complex 4

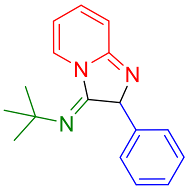

Symbolic Z-matrix:

Charge = 0 Multiplicity = 1

C	-0.65293	-0.66141	-1.19445
C	0.40263	0.24212	-0.52806
N	0.26888	1.49338	-0.50199
C	1.08285	2.57452	0.05015
N	-0.11337	-2.01001	-1.19827
C	2.47887	2.62403	-0.59158
H	3.03697	3.47975	-0.19818
H	3.07539	1.72595	-0.42739
H	2.37481	2.75416	-1.67281
C	1.1133	2.51128	1.58572
H	0.08963	2.55928	1.96816
H	1.56272	1.59938	1.98156
H	1.6694	3.36676	1.98235
C	0.35105	3.86886	-0.33925
H	-0.66269	3.86136	0.07105
H	0.88237	4.74702	0.04185
H	0.27746	3.94511	-1.4277
C	1.00035	-1.96692	-0.56406
C	1.89238	-3.07462	-0.29332
C	2.53613	-0.52291	0.66748
C	3.01803	-2.87686	0.42934
H	1.60307	-4.04136	-0.68729
C	3.35036	-1.56907	0.93791
H	2.7387	0.47235	1.02688
H	3.68272	-3.70935	0.63743
H	4.23968	-1.41143	1.5339
N	1.39358	-0.68984	-0.08296
C	-1.9929	-0.5575	-0.47992
C	-2.74946	0.61269	-0.60329
C	-2.47034	-1.60129	0.31229
C	-3.96437	0.73709	0.06447
H	-2.372	1.42739	-1.21406
C	-3.68865	-1.47437	0.98135
H	-1.89107	-2.51563	0.38662

C	-4.4376	-0.30686	0.8609	H	-5.38709	-0.21076	1.3785
H	-4.54576	1.64822	-0.04007	H	-0.78305	-0.31178	-2.22562
H	-4.0532	-2.29389	1.59332				

1.2.3. Step 3 – Aminopyridine acting as a proton shuttle

- Molecular complex 5

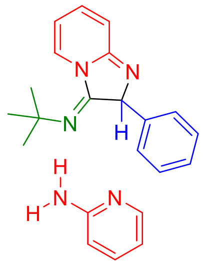

Symbolic Z-matrix:

Charge = 0 Multiplicity = 1

C	0.25104	-0.96677	-0.33058	C	4.85216	-0.86177	-0.77601
C	1.28816	0.14738	-0.15623	H	3.97413	1.01203	-0.27685
N	0.94036	1.31918	0.17055	H	5.42643	-2.91168	-1.28346
C	1.71346	2.54399	0.38959	H	5.85937	-0.46662	-0.8012
N	0.983	-2.18389	-0.66942	N	2.52454	-0.49888	-0.44598
C	2.38344	3.02038	-0.91018	C	-3.0459	0.03085	-2.65472
H	2.92893	3.95199	-0.72818	C	-4.3791	-0.32164	-2.4972
H	3.07473	2.2988	-1.34674	C	-4.99997	0.03324	-1.2934
H	1.60966	3.21758	-1.65752	C	-4.27806	0.70569	-0.32722
C	2.67976	2.38488	1.57623	C	-2.9211	1.00589	-0.58008
H	2.10092	2.18991	2.4843	N	-2.32167	0.6726	-1.73067
H	3.39149	1.5659	1.46646	H	-6.04212	-0.2165	-1.11616
H	3.24305	3.31158	1.72626	H	-2.51621	-0.21975	-3.57169
C	0.68483	3.61502	0.78631	H	-4.91003	-0.84912	-3.28064
H	0.1492	3.30705	1.68985	H	-4.72716	0.99223	0.61856
H	1.18103	4.571	0.98238	N	-2.15943	1.679	0.34448
H	-0.05035	3.75481	-0.01099	H	-2.45202	1.57389	1.3066
C	2.22135	-1.86476	-0.7204	H	-1.15051	1.58137	0.21886
C	3.33372	-2.74087	-1.02747	C	-0.64437	-1.17644	0.87609
C	3.8243	-0.03377	-0.4848	C	-1.91949	-1.71036	0.68247
C	4.59376	-2.25501	-1.05246	C	-0.22069	-0.87933	2.17269
H	3.09282	-3.77742	-1.22995	C	-2.76835	-1.92558	1.76568

H	-2.25565	-1.93687	-0.32709	H	-3.76365	-2.32557	1.5971
C	-1.06634	-1.09679	3.25921	H	-0.72703	-0.85906	4.2629
H	0.76791	-0.45855	2.33639	H	-3.00682	-1.77834	3.9031
C	-2.34534	-1.6152	3.05776	H	-0.38951	-0.67873	-1.1767

- Transition state 3

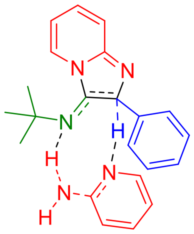

Symbolic Z-matrix:

Charge = 0 Multiplicity = 1

C	-0.00784	0.6882	-0.77736	H	3.83257	-0.36592	0.42357
C	1.11476	-0.1662	-0.38582	H	4.44086	3.88928	0.21036
N	0.99975	-1.44499	-0.18368	H	5.31516	1.5596	0.66715
C	1.99287	-2.4204	0.25288	N	2.15813	0.77349	-0.15042
N	0.40128	2.04485	-0.82336	C	-2.01719	1.71475	1.87899
C	2.45166	-2.17725	1.70568	C	-3.1307	1.75756	2.68639
H	3.17984	-2.93709	2.01108	C	-3.72109	0.52983	3.04092
H	2.89134	-1.19425	1.87527	C	-3.19043	-0.65796	2.59325
H	1.58147	-2.25284	2.36595	C	-2.03733	-0.63139	1.76835
C	3.15065	-2.55199	-0.75698	N	-1.48457	0.55533	1.45287
H	2.75656	-2.95266	-1.69577	H	-4.60621	0.51541	3.66952
H	3.63352	-1.60554	-1.0014	H	-1.50703	2.60641	1.52467
H	3.91308	-3.24394	-0.38236	H	-3.53676	2.70358	3.02018
C	1.26511	-3.77756	0.24897	H	-3.63513	-1.61152	2.85618
H	0.8442	-3.97309	-0.74175	N	-1.44665	-1.74375	1.29436
H	1.95294	-4.58983	0.50662	H	-1.93432	-2.62136	1.37463
H	0.4505	-3.77868	0.98089	H	-0.61416	-1.68821	0.67013
C	1.63214	2.06301	-0.43829	C	-1.12935	0.2832	-1.66479
C	2.49409	3.20309	-0.28252	C	-1.36085	-1.03974	-2.07106
C	3.479	0.63263	0.23492	C	-2.03701	1.27373	-2.07885
C	3.7815	3.03462	0.1024	C	-2.45816	-1.3524	-2.87312
H	2.06463	4.17371	-0.50207	H	-0.66863	-1.81559	-1.76527
C	4.28666	1.71094	0.36422	C	-3.13367	0.95269	-2.87165

H	-1.85319	2.30206	-1.78477	H	-3.81681	1.73812	-3.18218
C	-3.35454	-0.36491	-3.2746	H	-4.20914	-0.61528	-3.89542
H	-2.60892	-2.38157	-3.18728	H	-0.6837	0.61794	0.44227

- Molecular complex 6

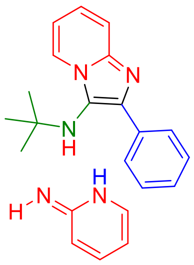

Symbolic Z-matrix:

Charge = 0 Multiplicity = 1

C	0.44059	0.42882	1.06703	H	2.62073	-1.86686	-1.38807
C	1.11136	-0.03752	-0.0646	H	3.13432	-4.26332	2.19334
N	1.27026	0.36201	-1.38925	H	3.59224	-3.90336	-0.25001
C	2.22178	1.44299	-1.72685	N	1.72982	-1.20874	0.36787
N	0.63476	-0.39801	2.14293	C	-2.77593	-1.93008	0.63088
C	2.23836	1.52724	-3.25334	C	-3.93595	-2.5372	0.26231
H	2.93196	2.30491	-3.58698	C	-4.34382	-2.40182	-1.10113
H	2.54457	0.56996	-3.6858	C	-3.60795	-1.68734	-1.99482
H	1.24126	1.77445	-3.63503	C	-2.37521	-1.0285	-1.60214
C	3.6103	1.06062	-1.20894	N	-2.03907	-1.22814	-0.26965
H	3.60063	0.92937	-0.12103	H	-5.26312	-2.87809	-1.42982
H	3.95469	0.12949	-1.6694	H	-2.3672	-1.95945	1.63494
H	4.33155	1.84877	-1.44529	H	-4.51965	-3.09624	0.98043
C	1.81399	2.7994	-1.13703	H	-3.92248	-1.58206	-3.02766
H	1.77113	2.75604	-0.04399	N	-1.58238	-0.29846	-2.32276
H	2.53845	3.57088	-1.42022	H	-1.93254	-0.23789	-3.27567
H	0.82708	3.1005	-1.50157	H	0.38285	0.39051	-1.89624
C	1.41962	-1.37483	1.71062	C	-0.41804	1.62117	1.20423
C	1.94608	-2.50701	2.3863	C	-1.21172	2.08612	0.148
C	2.48997	-2.1023	-0.33896	C	-0.45687	2.28845	2.43503
C	2.72207	-3.39403	1.6925	C	-2.00992	3.21549	0.31837
H	1.71241	-2.62855	3.43742	H	-1.22222	1.5574	-0.80303
C	2.99279	-3.19198	0.30456	C	-1.25359	3.41749	2.59894

H	0.14474	1.90671	3.25381	H	-1.2698	3.93127	3.55527
C	-2.02986	3.88773	1.53926	H	-2.65204	4.76815	1.6673
H	-2.62456	3.56471	-0.50584	H	-1.17698	-0.78546	0.03776

1.3. Proposal C: GBB reaction, methanol addition to nitrilium in the second step, protonating the amine

1.3.1. Step 1 - Isocyanide nucleophilic attack, forming nitrilium

- Molecular complex 1

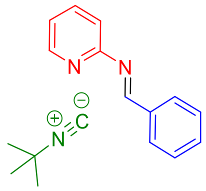

Symbolic Z-matrix:

Charge = 0 Multiplicity = 1

C	-0.33403	-1.2324	0.35932	C	-4.50063	0.42064	-0.97143
C	0.46501	1.36366	2.11397	H	-2.686	0.38412	-2.16475
N	0.92901	1.8087	1.13571	C	-5.06666	-0.01075	0.22679
C	1.51219	2.35791	-0.06581	H	-4.6812	-1.12754	2.03331
N	-1.1241	-0.9633	-0.608	H	-5.08159	1.00077	-1.68181
C	1.62943	3.87337	0.12343	H	-6.09548	0.21593	0.48286
H	2.07139	4.31709	-0.77331	N	-3.00369	-1.07918	0.84665
H	0.64392	4.31705	0.28705	H	-0.6718	-1.20384	1.40078
H	2.26589	4.10525	0.98142	C	1.08063	-1.55965	0.12017
C	0.58853	2.02162	-1.24008	C	1.95365	-1.6404	1.21032
H	0.48478	0.93955	-1.3577	C	1.57098	-1.76816	-1.17646
H	-0.40566	2.44823	-1.07783	C	3.30421	-1.91801	1.00985
H	1.00881	2.44235	-2.15848	H	1.57048	-1.46378	2.21208
C	2.89213	1.72071	-0.25488	C	2.918	-2.04925	-1.37438
H	2.8045	0.63664	-0.36512	H	0.87851	-1.70682	-2.01052
H	3.35697	2.13012	-1.15672	C	3.7872	-2.12217	-0.28199
H	3.53464	1.93705	0.60304	H	3.97815	-1.97189	1.85881
C	-2.46518	-0.6538	-0.29895	H	3.29511	-2.21389	-2.37894
C	-3.18119	0.08879	-1.24646	H	4.83892	-2.34053	-0.4399
C	-4.27409	-0.76037	1.09412				

- Transition state 1

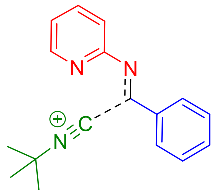

Symbolic Z-matrix:

Charge = 0 Multiplicity = 1

C	-0.98735	-0.58501	-0.55314	C	3.07865	-2.64118	0.71924
C	-0.29978	0.93852	-0.54939	H	1.26958	-2.69768	1.8984
N	0.53241	1.67094	-0.2089	C	3.65837	-2.1608	-0.46895
C	1.72129	2.33182	0.29435	H	3.25382	-1.02647	-2.24916
N	-0.27797	-1.26165	0.39904	H	3.66559	-3.23362	1.41568
C	2.66852	2.51662	-0.89326	H	4.69148	-2.36497	-0.72511
H	3.59521	2.97501	-0.53746	N	1.57049	-1.11048	-1.06643
H	2.89876	1.54665	-1.3412	H	-0.80299	-0.85326	-1.60468
H	2.22142	3.16637	-1.65014	C	-2.46064	-0.39342	-0.26582
C	2.3341	1.40476	1.3513	C	-3.28196	0.19984	-1.2263
H	1.60173	1.16406	2.12649	C	-3.00568	-0.82206	0.94236
H	2.67952	0.47311	0.89464	C	-4.64152	0.37017	-0.98132
H	3.18581	1.91439	1.81155	H	-2.85192	0.5286	-2.17075
C	1.29824	3.67233	0.89672	C	-4.3684	-0.65195	1.1877
H	0.60746	3.52095	1.73006	H	-2.34783	-1.29359	1.66423
H	2.18644	4.19012	1.2679	C	-5.18717	-0.05597	0.23049
H	0.81489	4.30108	0.14459	H	-5.27516	0.82917	-1.73382
C	1.00514	-1.574	0.07448	H	-4.79208	-0.99025	2.1284
C	1.76137	-2.35394	0.99509	H	-6.24777	0.07236	0.42355
C	2.85208	-1.41468	-1.31342				

- Molecular complex 2

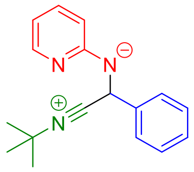

Symbolic Z-matrix:

Charge = 0 Multiplicity = 1

C	-0.92653	-0.59806	-0.58246	C	-0.26468	0.77003	-0.49059

N	0.42566	1.64131	-0.17274	H	1.35992	-2.81358	1.82953
C	1.56299	2.40667	0.31723	C	3.75896	-2.05377	-0.47024
N	-0.22198	-1.35551	0.37738	H	3.33665	-0.84114	-2.19115
C	2.4926	2.6265	-0.87812	H	3.77567	-3.22739	1.35585
H	3.38319	3.16037	-0.53589	H	4.80175	-2.20832	-0.72095
H	2.79242	1.66362	-1.29946	N	1.64057	-1.04787	-1.04116
H	1.99988	3.22232	-1.65087	H	-0.77017	-0.89892	-1.63292
C	2.23518	1.54313	1.39118	C	-2.4114	-0.43946	-0.28775
H	1.52922	1.29847	2.18906	C	-3.2328	0.22089	-1.20367
H	2.61119	0.61214	0.95669	C	-2.95402	-0.96082	0.8838
H	3.07203	2.10486	1.81643	C	-4.5938	0.36561	-0.94904
C	1.04195	3.72507	0.88662	H	-2.80616	0.61634	-2.12395
H	0.35843	3.54463	1.7201	C	-4.31897	-0.81808	1.13604
H	1.88949	4.31129	1.25076	H	-2.29178	-1.47869	1.56871
H	0.52162	4.30286	0.11844	C	-5.13901	-0.1551	0.22525
C	1.06948	-1.59397	0.06637	H	-5.22849	0.87632	-1.66648
C	1.84899	-2.39866	0.95502	H	-4.74284	-1.2291	2.04718
C	2.9373	-1.29344	-1.28308	H	-6.2007	-0.04784	0.42491
C	3.17693	-2.61882	0.68336				

1.3.2. Step 2 – Methanol addition to nitrilium, protonating the amine fragment

- Molecular complex 3

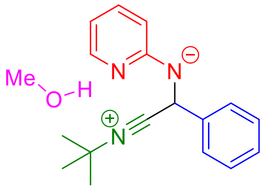

Symbolic Z-matrix:

Charge = 0 Multiplicity = 1

C	-0.74507	-0.57016	1.08957	H	1.70715	-1.70421	-2.99082
C	0.24586	-1.21331	0.14942	C	3.47425	-2.28592	-0.03983
N	1.22929	-1.5074	-0.37512	H	3.48125	-1.78155	0.92997
C	2.54581	-1.53619	-0.99665	H	3.14967	-3.32271	0.08555
N	-0.2159	0.72827	1.34196	H	4.48283	-2.28422	-0.46231
C	2.40896	-2.23979	-2.34638	C	2.96965	-0.07075	-1.151
H	3.3881	-2.25704	-2.83184	H	3.01199	0.40472	-0.1672
H	2.06473	-3.26956	-2.21876	H	3.96104	-0.04611	-1.61322

H	2.26384	0.47315	-1.78498
C	-0.11472	1.52862	0.24375
C	0.38254	2.85311	0.40801
C	-0.29437	1.8694	-2.04065
C	0.52489	3.66011	-0.69757
H	0.63787	3.19048	1.4071
C	0.17668	3.16964	-1.96932
H	-0.57805	1.43937	-3.00039
H	0.90355	4.67248	-0.58601
H	0.26716	3.77792	-2.86156
N	-0.43623	1.05959	-0.98263
O	2.25748	-0.03191	2.11635
H	1.36207	0.38034	1.94426
C	2.79336	0.50846	3.3024
H	2.12765	0.35191	4.16131

H	3.74195	0.00462	3.50968
H	2.99061	1.58503	3.20959
C	-2.1828	-0.72459	0.60228
C	-2.63273	-1.93528	0.07809
C	-3.07319	0.33409	0.75931
C	-3.96525	-2.08745	-0.29957
H	-1.94062	-2.76748	-0.04217
C	-4.40633	0.18212	0.38356
H	-2.70788	1.27154	1.16677
C	-4.85596	-1.0261	-0.14729
H	-4.30635	-3.03216	-0.71159
H	-5.09496	1.01311	0.50077
H	-5.89432	-1.14012	-0.44221
H	-0.62874	-1.16354	2.00883

- Transition state 2

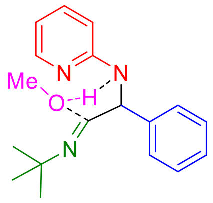

Symbolic Z-matrix:

Charge = 0 Multiplicity = 1

C	-0.41262	0.4438	-1.28652
C	0.91305	0.58021	-0.5759
N	1.66151	1.00244	0.22292
C	2.91977	0.80822	0.93937
N	-0.66066	-0.95808	-1.53647
C	3.08957	1.96283	1.92425
H	4.01457	1.8235	2.4902
H	3.14614	2.91859	1.39567
H	2.25096	1.99687	2.62442
C	4.05315	0.77275	-0.08788
H	3.89967	-0.05584	-0.78305
H	4.10036	1.71286	-0.64558
H	5.00307	0.63234	0.43615

C	2.78717	-0.53634	1.66129
H	2.63134	-1.33705	0.93338
H	3.70385	-0.7287	2.2266
H	1.93805	-0.52003	2.34908
C	-0.89887	-1.71133	-0.40071
C	-1.31134	-3.05688	-0.56655
C	-0.92265	-1.9603	1.89261
C	-1.52058	-3.83675	0.55141
H	-1.45961	-3.43484	-1.57213
C	-1.32987	-3.28283	1.82633
H	-0.76002	-1.48301	2.85787
H	-1.83917	-4.86976	0.4433
H	-1.49353	-3.85869	2.72976

N	-0.70002	-1.18777	0.821
O	1.7491	-0.8962	-1.72157
H	0.6415	-1.19589	-1.81197
C	2.20599	-0.39795	-2.95849
H	1.39215	-0.35067	-3.69645
H	2.62365	0.61385	-2.84352
H	2.99323	-1.04686	-3.35955
C	-1.50316	1.26349	-0.60335
C	-1.24077	2.51977	-0.05544
C	-2.81385	0.78484	-0.62081
C	-2.27436	3.28398	0.48372
H	-0.22598	2.90811	-0.03786
C	-3.84559	1.54947	-0.08185
H	-3.01366	-0.18945	-1.05465
C	-3.58053	2.79996	0.47433
H	-2.05617	4.25758	0.91178
H	-4.86019	1.16348	-0.09426
H	-4.38579	3.39288	0.89649
H	-0.2303	0.90244	-2.27013

- Molecular complex 4

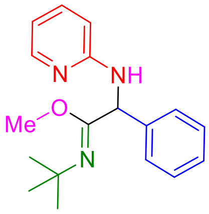

Symbolic Z-matrix:

Charge = 0 Multiplicity = 1

C	-0.14732	0.56484	1.27674
C	-0.5726	-0.79079	0.69681
N	-1.48641	-0.86218	- 0.16323
C	-1.92591	-2.13532	-0.752
N	1.29955	0.64423	1.41577
C	-3.09982	-1.78524	-1.67247
H	-3.47068	-2.67829	-2.1863
H	-3.91804	-1.3474	-1.09199
H	-2.7842	-1.04985	-2.41776
C	-2.40842	-3.13394	0.31102
H	-1.58267	-3.50128	0.92303
H	-3.15161	-2.65966	0.96142
H	-2.88468	-3.99018	- 0.17815
C	-0.78264	-2.72845	-1.58653
H	0.06856	-2.99108	-0.95405
H	-1.13015	-3.62431	- 2.11235
H	-0.44983	-1.995	-2.32773
C	2.20555	0.32377	0.42073
C	3.57808	0.58507	0.62017
C	2.6162	-0.59945	-1.63269
C	4.4646	0.22591	-0.37761
H	3.92051	1.05075	1.53921
C	3.98499	-0.38834	-1.53927
H	2.18709	-1.07027	-2.51466
H	5.52614	0.41757	-0.25263
H	4.64881	-0.68801	-2.34127
N	1.73885	-0.24674	-0.68862
O	0.11566	-1.87403	1.17828
H	1.62125	1.33083	2.08271
C	0.23808	-1.9925	2.58942
H	0.87699	-1.20635	3.00243
H	-0.74959	-1.97137	3.06709

H	0.70001	-2.96337	2.77075	H	0.93954	2.30472	-0.58063
C	-0.79518	1.73947	0.56164	C	-2.05491	3.90585	-0.69219
C	-2.12915	2.0457	0.84193	H	-3.79658	3.34179	0.44633
C	-0.09552	2.53096	-0.34728	H	-0.16845	4.21549	-1.68072
C	-2.75853	3.11861	0.21987	H	-2.5417	4.74547	-1.17845
H	-2.68206	1.42529	1.54341	H	-0.52941	0.57909	2.30878
C	-0.72393	3.60893	-0.97201				

1.4. Proposal D: GBB reaction, methanol addition to nitrilium in the second step, protonating the pyridine

1.4.1. Step 1 - Isocyanide nucleophilic attack, forming nitrilium

- Molecular complex 1

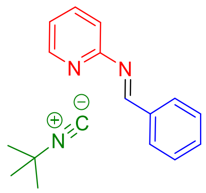

Symbolic Z-matrix:

Charge = 0 Multiplicity = 1

C	-0.33403	-1.2324	0.35932	C	-2.46518	-0.6538	-0.29895
C	0.46501	1.36366	2.11397	C	-3.18119	0.08879	-1.24646
N	0.92901	1.8087	1.13571	C	-4.27409	-0.76037	1.09412
C	1.51219	2.35791	-0.06581	C	-4.50063	0.42064	-0.97143
N	-1.1241	-0.9633	-0.608	H	-2.686	0.38412	-2.16475
C	1.62943	3.87337	0.12343	C	-5.06666	-0.01075	0.22679
H	2.07139	4.31709	-0.77331	H	-4.6812	-1.12754	2.03331
H	0.64392	4.31705	0.28705	H	-5.08159	1.00077	-1.68181
H	2.26589	4.10525	0.98142	H	-6.09548	0.21593	0.48286
C	0.58853	2.02162	-1.24008	N	-3.00369	-1.07918	0.84665
H	0.48478	0.93955	-1.3577	H	-0.6718	-1.20384	1.40078
H	-0.40566	2.44823	-1.07783	C	1.08063	-1.55965	0.12017
H	1.00881	2.44235	-2.15848	C	1.95365	-1.6404	1.21032
C	2.89213	1.72071	-0.25488	C	1.57098	-1.76816	-1.17646
H	2.8045	0.63664	-0.36512	C	3.30421	-1.91801	1.00985
H	3.35697	2.13012	-1.15672	H	1.57048	-1.46378	2.21208
H	3.53464	1.93705	0.60304	C	2.918	-2.04925	-1.37438

H	0.87851	-1.70682	-2.01052	H	3.29511	-2.21389	-2.37894
C	3.7872	-2.12217	-0.28199	H	4.83892	-2.34053	-0.4399
H	3.97815	-1.97189	1.85881				

- Transition state 1

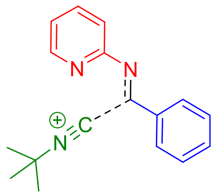

Symbolic Z-matrix:

Charge = 0 Multiplicity = 1

C	-0.98735	-0.58501	-0.55314	C	3.07865	-2.64118	0.71924
C	-0.29978	0.93852	-0.54939	H	1.26958	-2.69768	1.8984
N	0.53241	1.67094	-0.2089	C	3.65837	-2.1608	-0.46895
C	1.72129	2.33182	0.29435	H	3.25382	-1.02647	-2.24916
N	-0.27797	-1.26165	0.39904	H	3.66559	-3.23362	1.41568
C	2.66852	2.51662	-0.89326	H	4.69148	-2.36497	-0.72511
H	3.59521	2.97501	-0.53746	N	1.57049	-1.11048	-1.06643
H	2.89876	1.54665	-1.3412	H	-0.80299	-0.85326	-1.60468
H	2.22142	3.16637	-1.65014	C	-2.46064	-0.39342	-0.26582
C	2.3341	1.40476	1.3513	C	-3.28196	0.19984	-1.2263
H	1.60173	1.16406	2.12649	C	-3.00568	-0.82206	0.94236
H	2.67952	0.47311	0.89464	C	-4.64152	0.37017	-0.98132
H	3.18581	1.91439	1.81155	H	-2.85192	0.5286	-2.17075
C	1.29824	3.67233	0.89672	C	-4.3684	-0.65195	1.1877
H	0.60746	3.52095	1.73006	H	-2.34783	-1.29359	1.66423
H	2.18644	4.19012	1.2679	C	-5.18717	-0.05597	0.23049
H	0.81489	4.30108	0.14459	H	-5.27516	0.82917	-1.73382
C	1.00514	-1.574	0.07448	H	-4.79208	-0.99025	2.1284
C	1.76137	-2.35394	0.99509	H	-6.24777	0.07236	0.42355
C	2.85208	-1.41468	-1.31342				

- Molecular complex 2

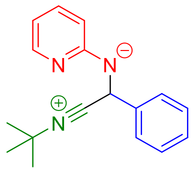

Symbolic Z-matrix:

Charge = 0 Multiplicity = 1

C	-0.92653	-0.59806	-0.58246	C	3.17693	-2.61882	0.68336
C	-0.26468	0.77003	-0.49059	H	1.35992	-2.81358	1.82953
N	0.42566	1.64131	-0.17274	C	3.75896	-2.05377	-0.47024
C	1.56299	2.40667	0.31723	H	3.33665	-0.84114	-2.19115
N	-0.22198	-1.35551	0.37738	H	3.77567	-3.22739	1.35585
C	2.4926	2.6265	-0.87812	H	4.80175	-2.20832	-0.72095
H	3.38319	3.16037	-0.53589	N	1.64057	-1.04787	-1.04116
H	2.79242	1.66362	-1.29946	H	-0.77017	-0.89892	-1.63292
H	1.99988	3.22232	-1.65087	C	-2.4114	-0.43946	-0.28775
C	2.23518	1.54313	1.39118	C	-3.2328	0.22089	-1.20367
H	1.52922	1.29847	2.18906	C	-2.95402	-0.96082	0.8838
H	2.61119	0.61214	0.95669	C	-4.5938	0.36561	-0.94904
H	3.07203	2.10486	1.81643	H	-2.80616	0.61634	-2.12395
C	1.04195	3.72507	0.88662	C	-4.31897	-0.81808	1.13604
H	0.35843	3.54463	1.7201	H	-2.29178	-1.47869	1.56871
H	1.88949	4.31129	1.25076	C	-5.13901	-0.1551	0.22525
H	0.52162	4.30286	0.11844	H	-5.22849	0.87632	-1.66648
C	1.06948	-1.59397	0.06637	H	-4.74284	-1.2291	2.04718
C	1.84899	-2.39866	0.95502	H	-6.2007	-0.04784	0.42491
C	2.9373	-1.29344	-1.28308				

1.4.2. Step 2 – Methanol addition to nitrilium, protonating the pyridine fragment

- Molecular complex 3

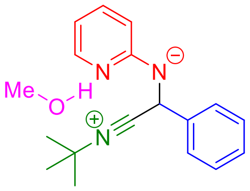

Symbolic Z-matrix:

Charge = 0 Multiplicity = 1

C	0.56816	-0.10469	-1.75103	C	-2.66681	-2.81385	0.5728
C	-0.65012	-0.76348	-1.17203	H	-3.57107	-3.08925	1.1224
N	-1.68343	-1.12538	-0.81715	H	-2.43831	-3.60769	-0.1439
C	-2.92321	-1.48831	-0.14804	H	-1.8343	-2.6935	1.27035
N	0.30245	1.28075	-1.91989	C	-4.00552	-1.61233	-1.22103

H	-4.14978	-0.66171	-1.74048	H	-0.06594	-0.20576	1.1511
H	-3.74666	-2.38385	-1.9509	C	1.04502	-1.08775	2.50117
H	-4.94432	-1.89245	-0.73662	H	1.93003	-0.70475	1.97822
C	-3.2287	-0.36422	0.84662	H	0.89926	-0.50363	3.42048
H	-3.30152	0.60034	0.33709	H	1.23453	-2.12848	2.77973
H	-4.18394	-0.58676	1.33077	C	1.81446	-0.54542	-0.9671
H	-2.4412	-0.31154	1.60185	C	2.04553	-1.89583	-0.70214
C	-0.03296	1.96682	-0.81819	C	2.76368	0.40356	-0.59857
C	-0.23727	3.38205	-0.94544	C	3.22118	-2.29958	-0.07504
C	-0.56276	2.13915	1.45444	H	1.29982	-2.63991	-0.97829
C	-0.59603	4.12659	0.14643	C	3.94282	-0.00059	0.02895
H	-0.09759	3.82088	-1.92695	H	2.57211	1.45013	-0.81233
C	-0.77082	3.49979	1.40143	C	4.17606	-1.34961	0.28918
H	-0.68377	1.5976	2.3938	H	3.39125	-3.35194	0.1302
H	-0.74754	5.19802	0.04565	H	4.67954	0.74352	0.31529
H	-1.05334	4.05472	2.28763	H	5.09441	-1.66052	0.77733
N	-0.20423	1.37899	0.40106	H	0.6441	-0.55809	-2.75117
O	-0.10069	-1.04747	1.67452				

- Transition state 2

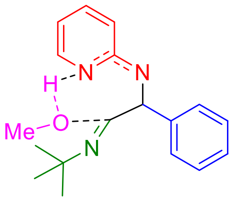

Symbolic Z-matrix:

Charge = 0 Multiplicity = 1

C	0.34563	-0.38931	-1.43767	H	-4.47691	0.20845	-1.84088
C	-0.97315	-0.56981	-0.70156	H	-4.47276	-1.56192	-1.69004
N	-2.14718	-0.57359	-0.79478	H	-5.51031	-0.58344	-0.63195
C	-3.42632	-0.52947	-0.0918	C	-3.47592	0.80431	0.66047
N	0.61712	0.97399	-1.74934	H	-3.38363	1.64215	-0.03584
C	-3.4701	-1.71333	0.87825	H	-4.43625	0.88272	1.17859
H	-4.42876	-1.70408	1.40494	H	-2.66227	0.85204	1.38775
H	-3.3812	-2.65961	0.3366	C	0.75432	1.8768	-0.78745
H	-2.65784	-1.63062	1.60311	C	1.13188	3.21413	-1.17397
C	-4.53991	-0.62281	-1.13406	C	0.72881	2.6208	1.45766

C	1.30559	4.18918	-0.23407
H	1.27335	3.3971	-2.23292
C	1.10389	3.90134	1.14083
H	0.54142	2.32743	2.48948
H	1.5968	5.18982	-0.54191
H	1.23019	4.65208	1.91058
N	0.56115	1.63515	0.54822
O	-0.53045	-0.46776	1.28602
H	0.03014	0.44552	1.04718
C	0.18317	-1.37888	2.10038
H	1.26512	-1.31596	1.93268
H	-0.03016	-1.16976	3.15656
H	-0.13749	-2.40249	1.87828
C	1.45077	-1.22924	-0.8015
C	1.26488	-2.60644	-0.66041
C	2.65805	-0.65757	-0.40978
C	2.25981	-3.40337	-0.10223
H	0.32774	-3.05917	-0.98283
C	3.65622	-1.45409	0.15351
H	2.8124	0.40711	-0.54715
C	3.45896	-2.82406	0.31477
H	2.10281	-4.47224	0.00566
H	4.5918	-0.99993	0.46484
H	4.23815	-3.43996	0.75273
H	0.09407	-0.88106	-2.39061

- Molecular complex 4

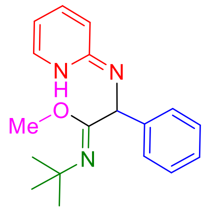

Symbolic Z-matrix:
Charge = 0 Multiplicity = 1

C	0.6308	-0.14776	-1.19854
C	-0.32545	-0.982	-0.32429
N	-1.32432	-1.52069	-0.8678
C	-2.41419	-2.19052	-0.13916
N	0.15791	1.1878	-1.52201
C	-1.91609	-3.35785	0.72339
H	-2.77177	-3.94575	1.07074
H	-1.26923	-4.0172	0.13491
H	-1.36799	-3.00894	1.60007
C	-3.35756	-2.73475	-1.2171
H	-3.70185	-1.92033	-1.86016
H	-2.83446	-3.46294	-1.8443
H	-4.22626	-3.22118	-0.76195
C	-3.1614	-1.15979	0.7185
H	-3.47545	-0.31246	0.10086
H	-4.05387	-1.61846	1.15676
H	-2.52554	-0.78963	1.52671
C	-0.45774	1.97858	-0.71208
C	-0.91875	3.27581	-1.19173
C	-1.45635	2.61077	1.43238
C	-1.60508	4.13347	-0.39903
H	-0.68772	3.4983	-2.22658
C	-1.90453	3.80067	0.96649
H	-1.62095	2.26854	2.44816
H	-1.93876	5.08566	-0.80055
H	-2.46281	4.46864	1.6084
N	-0.74862	1.74871	0.63724
O	-0.12742	-0.95723	1.05814

H	-0.42174	0.88399	1.05951
C	0.90039	-1.78609	1.61857
H	1.8428	-1.24014	1.70201
H	0.54848	-2.07963	2.60996
H	1.04555	-2.68128	1.0071
C	2.05463	-0.16134	-0.66473
C	2.8648	-1.27076	-0.92276
C	2.5623	0.87877	0.11504
C	4.14296	-1.36325	-0.37752
H	2.48156	-2.07387	-1.54928
C	3.84043	0.78899	0.66595
H	1.95876	1.76522	0.28636
C	4.63073	-0.33532	0.42981
H	4.75996	-2.23198	-0.58606
H	4.22235	1.60238	1.27545
H	5.62613	-0.40247	0.85747
H	0.63779	-0.68511	-2.1517

1.5. Proposal E: GBB reaction, methanol acts as a hydrogen bond donor in the first and second steps, and as a proton shuttle in the third step

1.5.1. Step 1 - Isocyanide nucleophilic attack, forming nitrilium (methanol as a hydrogen bond donor)

- Molecular complex 1

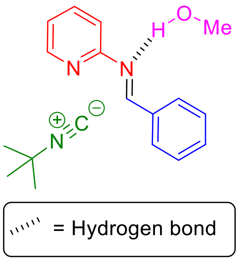

Symbolic Z-matrix:

Charge = 0 Multiplicity = 1

C	-1.1052	-1.23604	-0.64475
C	0.10961	1.38295	-1.57849
N	1.02702	1.77047	-0.965
C	2.17007	2.26661	-0.23525
N	-0.35009	-1.44307	0.36885
C	3.18843	2.77154	-1.262
H	4.06947	3.15456	-0.73919
H	3.49732	1.9592	-1.92534
H	2.76012	3.57575	-1.86627
C	2.74322	1.11634	0.59539
H	1.99869	0.78233	1.32221
H	3.02826	0.27874	-0.04881
H	3.63168	1.46799	1.12921
C	1.69379	3.39792	0.6798
H	0.98388	3.00229	1.40972
H	2.55419	3.81605	1.21077
H	1.21884	4.1923	0.09732
C	0.98446	-1.85377	0.14985
C	1.74562	-2.14788	1.28767
C	2.74246	-2.31243	-1.23717
C	3.06702	-2.53983	1.12085
H	1.28692	-2.05232	2.26582
C	3.58465	-2.62495	-0.17026
H	3.10658	-2.36703	-2.26061

H	3.68471	-2.77197	1.98282	C	-3.16817	-0.98272	0.74492
H	4.61069	-2.92435	-0.35246	C	-4.46613	0.25927	-1.39401
N	1.4729	-1.93585	-1.08974	H	-2.62368	-0.04087	-2.47852
O	-0.36969	1.00865	2.0068	C	-4.4797	-0.54495	0.88764
H	-0.47381	0.13245	1.59763	H	-2.64738	-1.46983	1.56417
C	-1.46897	1.82072	1.63758	C	-5.12791	0.08139	-0.17929
H	-1.27258	2.82751	2.01641	H	-4.96929	0.74842	-2.22181
H	-1.59133	1.87332	0.54764	H	-4.99977	-0.69002	1.82937
H	-2.40748	1.46363	2.07948	H	-6.15074	0.42664	-0.06388
C	-2.49825	-0.80261	-0.47233	H	-0.73277	-1.36044	-1.66532
C	-3.15728	-0.19064	-1.54405				

- Transition state 1

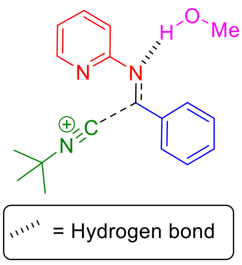

Symbolic Z-matrix:

Charge = 0 Multiplicity = 1

C	-0.79744	-0.85701	-0.79328	H	0.3248	4.05805	0.01416
C	-0.13992	0.71832	-1.05099	C	1.19906	-1.66194	0.06068
N	0.63393	1.52235	-0.73951	C	1.96104	-2.14689	1.15323
C	1.68761	2.38025	-0.22996	C	3.03783	-1.81944	-1.32458
N	-0.10901	-1.30931	0.2883	C	3.28947	-2.46148	0.95946
C	2.42679	2.9614	-1.43698	H	1.47707	-2.24736	2.11889
H	3.23706	3.6019	-1.07901	C	3.85724	-2.29927	-0.31366
H	2.85494	2.16028	-2.04525	H	3.43268	-1.67563	-2.329
H	1.75394	3.56051	-2.0564	H	3.88959	-2.8298	1.78656
C	2.60697	1.50201	0.62768	H	4.89665	-2.53603	-0.50956
H	2.04303	1.07803	1.46189	N	1.74948	-1.49471	-1.1586
H	3.04307	0.69472	0.03166	O	-0.1911	0.73763	2.1899
H	3.41336	2.13028	1.01854	H	-0.22089	-0.08274	1.63951
C	1.0229	3.46858	0.61506	C	-1.49487	1.26176	2.31
H	0.49374	3.00732	1.45216	H	-1.43902	2.15639	2.93739
H	1.79953	4.13323	1.0033	H	-1.92098	1.54602	1.33521

H	-2.18497	0.55262	2.78501	H	-2.32105	-1.81089	1.20484
C	-2.28353	-0.66462	-0.59845	C	-5.04362	-0.37798	-0.26415
C	-3.03578	0.05275	-1.53147	H	-4.98536	0.76216	-2.09366
C	-2.91964	-1.24907	0.4954	H	-4.78618	-1.55746	1.51765
C	-4.4095	0.1987	-1.3662	H	-6.11494	-0.26327	-0.13184
H	-2.53826	0.50762	-2.3858	H	-0.54442	-1.29482	-1.76699
C	-4.29716	-1.10284	0.66155				

- Molecular complex 2

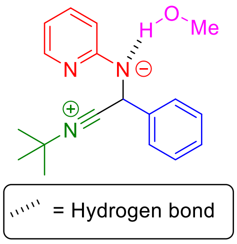

Symbolic Z-matrix:

Charge = 0 Multiplicity = 1

C	-0.68089	-0.74936	-0.90637	H	1.54059	-2.47904	1.94944
C	-0.05647	0.63402	-0.951	C	3.99237	-2.14427	-0.39272
N	0.57169	1.56292	-0.68378	H	3.60622	-1.2872	-2.32529
C	1.50862	2.50645	-0.08859	H	3.97424	-2.93623	1.62646
N	-0.02099	-1.36234	0.19118	H	5.04402	-2.3257	-0.58085
C	2.26381	3.18933	-1.22831	N	1.88245	-1.3015	-1.19973
H	2.98373	3.89099	-0.79937	O	-0.38656	0.59517	2.08112
H	2.8061	2.45181	-1.82578	H	-0.28312	-0.22888	1.53331
H	1.57933	3.7436	-1.876	C	-1.65119	0.58884	2.70668
C	2.44073	1.67271	0.79962	H	-1.65849	1.37901	3.46323
H	1.86172	1.17341	1.58104	H	-2.46484	0.78094	1.99283
H	2.9761	0.92406	0.20779	H	-1.85424	-0.36628	3.2086
H	3.16651	2.34837	1.26243	C	-2.18431	-0.61762	-0.70999
C	0.68515	3.49324	0.74001	C	-2.92489	0.31837	-1.43391
H	0.12412	2.9467	1.50212	C	-2.83288	-1.47374	0.17829
H	1.36957	4.19524	1.2241	C	-4.30476	0.40591	-1.26684
H	-0.00363	4.05727	0.10493	H	-2.42303	0.98702	-2.13122
C	1.29362	-1.63577	-0.02744	C	-4.21541	-1.38975	0.34015
C	2.04774	-2.23687	1.02161	H	-2.23679	-2.18543	0.73936
C	3.18762	-1.56672	-1.35936	C	-4.95348	-0.45015	-0.37709
C	3.3874	-2.48263	0.83246	H	-4.87172	1.14126	-1.829

H	-4.71552	-2.05732	1.03507
H	-6.02861	-0.38287	-0.2437
H	-0.44856	-1.19745	-1.88532

1.5.2. Step 2 – Ring Closure (methanol as a hydrogen bond donor)

- Molecular complex 3

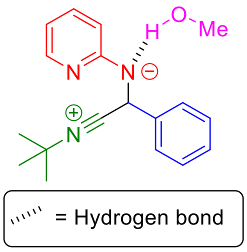

Symbolic Z-matrix:

Charge = 0 Multiplicity = 1

C	-0.72319	-0.71857	-0.93048
C	-0.10586	0.66997	-0.94431
N	0.51246	1.59891	-0.65534
C	1.44776	2.54811	-0.06717
N	-0.04309	-1.35907	0.13695
C	2.10797	3.31728	-1.21105
H	2.824	4.02609	-0.78736
H	2.64272	2.63438	-1.87629
H	1.36569	3.87437	-1.78887
C	2.46072	1.70788	0.72061
H	1.9471	1.15026	1.50857
H	2.98229	1.00799	0.0608
H	3.19095	2.38638	1.17201
C	0.63912	3.45917	0.85743
H	0.13645	2.85306	1.61518
H	1.32644	4.15758	1.34274
H	-0.10048	4.03201	0.29114
C	1.267	-1.62738	-0.11635
C	2.0401	-2.26147	0.8985
C	3.13377	-1.52218	-1.48311
C	3.37488	-2.50541	0.67508
H	1.55052	-2.5326	1.82797
C	3.95573	-2.13179	-0.55126
H	3.53375	-1.21431	-2.44827
H	3.97587	-2.98538	1.44254
H	5.00255	-2.31104	-0.76645
N	1.83301	-1.2584	-1.28908
O	-0.25053	0.49565	2.12547
H	-0.19878	-0.28491	1.50661
C	-1.15069	0.19845	3.16842
H	-1.10383	1.00999	3.90023
H	-2.18523	0.12039	2.80642
H	-0.88851	-0.73734	3.68042
C	-2.22446	-0.58405	-0.71614
C	-2.98895	0.19395	-1.58825
C	-2.84515	-1.26701	0.32644
C	-4.366	0.29741	-1.41522
H	-2.50498	0.72035	-2.40941
C	-4.22616	-1.16413	0.4983
H	-2.23345	-1.87402	0.98515
C	-4.98781	-0.38272	-0.36728
H	-4.95327	0.90477	-2.0967
H	-4.70664	-1.69798	1.31255
H	-6.06162	-0.30396	-0.22949
H	-0.50656	-1.13632	-1.92587

- Transition state 2

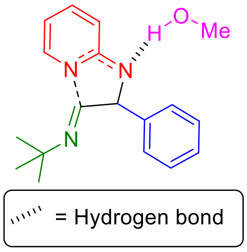

Symbolic Z-matrix:

Charge = 0 Multiplicity = 1

C	-0.49989	-0.92306	-1.05095	H	3.62744	0.24372	-1.90115
C	0.14776	0.43742	-0.90631	H	4.45418	-2.91286	0.90304
N	0.25322	1.52953	-0.52468	H	5.30496	-1.11725	-0.65637
C	0.79106	2.70971	0.12782	N	1.98844	-0.74071	-1.11801
N	0.17143	-1.79403	-0.13087	O	-0.11221	-0.04137	2.08926
C	1.13109	3.7321	-0.95702	H	-0.06237	-0.80648	1.47192
H	1.53557	4.62934	-0.48128	C	-1.09194	-0.29283	3.07354
H	1.88142	3.33171	-1.64407	H	-1.04736	0.52051	3.80332
H	0.23929	4.01054	-1.52493	H	-2.10281	-0.32092	2.64501
C	2.03408	2.25747	0.90079	H	-0.90693	-1.23598	3.60343
H	1.7577	1.48207	1.62093	C	-1.99197	-0.78879	-0.78538
H	2.79148	1.85946	0.22016	C	-2.75348	0.12908	-1.5134
H	2.45055	3.11911	1.43111	C	-2.61588	-1.60763	0.15385
C	-0.29195	3.22512	1.07798	C	-4.12393	0.23883	-1.29667
H	-0.547	2.44174	1.79586	H	-2.27214	0.76321	-2.25573
H	0.08958	4.10001	1.61194	C	-3.99054	-1.49984	0.36832
H	-1.18943	3.51277	0.52344	H	-2.01197	-2.32568	0.69818
C	1.50236	-1.69131	-0.27842	C	-4.74612	-0.57684	-0.35083
C	2.40765	-2.50632	0.47113	H	-4.70616	0.95665	-1.86601
C	3.31529	-0.54573	-1.2186	H	-4.471	-2.14229	1.1001
C	3.75593	-2.30131	0.33837	H	-5.81502	-0.49472	-0.18036
H	1.99554	-3.26028	1.13262	H	-0.32921	-1.20171	-2.10526
C	4.2438	-1.29359	-0.5284				

- Molecular complex 4

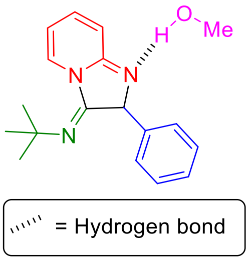

Symbolic Z-matrix:

Charge = 0 Multiplicity = 1

C	-0.53806	-0.56323	-1.56303	H	2.34175	0.08585	1.35994
C	0.53711	0.20389	-0.77058	H	3.17426	-4.09155	0.69682
N	0.59982	1.4613	-0.77822	H	3.56621	-1.96727	2.00926
C	1.61518	2.38199	-0.258	N	1.34126	-0.84405	-0.22529
N	-0.09913	-1.94887	-1.62232	O	-0.39827	-0.07501	2.19302
C	3.04283	1.95455	-0.63719	H	-0.77948	-0.74351	1.60942
H	3.73703	2.76754	-0.40202	C	-1.34064	0.98104	2.30496
H	3.39506	1.06121	-0.12156	H	-0.96635	1.66174	3.07289
H	3.095	1.76566	-1.71401	H	-1.44088	1.52703	1.35849
C	1.43984	2.5992	1.25298	H	-2.32648	0.61237	2.61041
H	1.40267	1.66641	1.81934	C	-1.90198	-0.40309	-0.90894
H	2.25578	3.22206	1.63399	C	-2.60352	0.79665	-1.06583
H	0.49716	3.1218	1.44019	C	-2.44803	-1.41606	-0.11725
C	1.33494	3.71969	-0.96061	C	-3.82975	0.9827	-0.43338
H	0.30124	4.02507	-0.77728	H	-2.17315	1.58646	-1.67521
H	2.00801	4.4997	-0.59013	C	-3.67376	-1.22435	0.52415
H	1.47474	3.6182	-2.04094	H	-1.92188	-2.36291	-0.03239
C	0.92204	-2.04572	-0.84722	C	-4.36669	-0.02588	0.36827
C	1.67963	-3.23705	-0.53181	H	-4.36784	1.91652	-0.56518
C	2.22839	-0.8447	0.82701	H	-4.09023	-2.01882	1.1361
C	2.62001	-3.19363	0.44153	H	-5.32302	0.11985	0.86087
H	1.43145	-4.13972	-1.07677	H	-0.57822	-0.13125	-2.56811
C	2.87908	-1.97949	1.1737				

1.5.3. Step 3 – Methanol acting as a proton shuttle

- Molecular complex 5

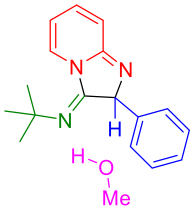

Symbolic Z-matrix:

Charge = 0 Multiplicity = 1

C	0.49088	-0.7172	1.08627	H	-2.77406	0.426	-1.32586
C	-0.41805	0.16031	0.20531	H	-4.50199	-3.15319	0.32614
N	-0.03757	1.29763	-0.19914	H	-4.65293	-1.15065	-1.21033
C	-0.66924	2.2987	-1.06534	N	-1.59628	-0.62165	0.06247
N	-0.31369	-1.84955	1.51557	O	1.70308	2.29942	1.82048
C	-1.97365	2.83316	-0.45178	H	1.29987	2.0006	0.9808
H	-2.39267	3.6134	-1.09517	C	0.65846	2.75679	2.64764
H	-2.73958	2.07287	-0.29619	H	-0.13821	2.00604	2.76135
H	-1.75513	3.27896	0.52353	H	1.07877	2.9643	3.63392
C	-0.8222	1.75981	-2.49697	H	0.20427	3.68385	2.26748
H	-1.42422	0.85316	-2.56858	C	1.72162	-1.14104	0.29898
H	-1.27458	2.52462	-3.13617	C	2.90841	-0.41515	0.41372
H	0.16707	1.51925	-2.89658	C	1.65701	-2.22434	-0.57964
C	0.32252	3.47149	-1.1262	C	4.01911	-0.76857	-0.35189
H	1.29036	3.12577	-1.5001	H	2.96123	0.42163	1.1056
H	-0.0531	4.25809	-1.78805	C	2.76688	-2.57379	-1.3454
H	0.4773	3.89953	-0.13093	H	0.74157	-2.80584	-0.64767
C	-1.43782	-1.7573	0.90963	C	3.95075	-1.84487	-1.2344
C	-2.55366	-2.67656	0.99704	H	4.94078	-0.2035	-0.25232
C	-2.7438	-0.43817	-0.68401	H	2.70994	-3.42001	-2.02331
C	-3.66854	-2.46077	0.26499	H	4.81764	-2.12033	-1.82702
H	-2.4333	-3.52461	1.66021	H	0.81722	-0.12766	1.95102
C	-3.76836	-1.31625	-0.60929				

- Transition state 3

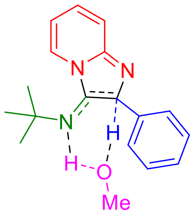

Symbolic Z-matrix:

Charge = 0 Multiplicity = 1

C	-0.69836	0.74232	0.43875	H	3.08051	-0.09344	-1.11035
C	0.44039	-0.0222	-0.0777	H	3.70128	4.08469	-0.26717
N	0.41414	-1.33782	-0.08594	H	4.53708	1.87391	-1.15862
C	1.40328	-2.31534	-0.53987	N	1.45936	0.92597	-0.22759
N	-0.29453	2.08271	0.64097	O	-0.84732	-1.25327	2.04634
C	2.67023	-2.31774	0.33764	H	-0.35331	-1.56244	1.09175
H	3.39734	-3.04155	-0.0456	C	0.13636	-1.29275	3.07322
H	3.1596	-1.34573	0.40699	H	0.97259	-0.6191	2.8401
H	2.40463	-2.61696	1.35684	H	-0.32475	-0.98356	4.01224
C	1.69858	-2.15081	-2.03949	H	0.517	-2.31361	3.17524
H	2.07772	-1.16312	-2.30636	C	-2.10296	0.54469	-0.05355
H	2.42806	-2.89798	-2.3687	C	-2.56849	-0.67327	-0.5679
H	0.77332	-2.29895	-2.6038	C	-2.99974	1.62032	0.03234
C	0.7248	-3.68254	-0.35823	C	-3.88704	-0.80088	-1.00072
H	-0.19232	-3.73227	-0.95198	H	-1.89697	-1.52136	-0.64005
H	1.39258	-4.49103	-0.67185	C	-4.31807	1.48145	-0.39087
H	0.46073	-3.84319	0.69282	H	-2.64327	2.56641	0.42324
C	0.93084	2.16196	0.25232	C	-4.77202	0.27078	-0.91269
C	1.79236	3.31318	0.2431	H	-4.22301	-1.75167	-1.40426
C	2.74923	0.85864	-0.73175	H	-4.99276	2.32938	-0.31743
C	3.05164	3.21643	-0.24446	H	-5.8001	0.16439	-1.24415
H	1.37469	4.23778	0.62378	H	-0.85284	-0.1178	1.55471
C	3.53695	1.95698	-0.75186				

- **Molecular complex 6**

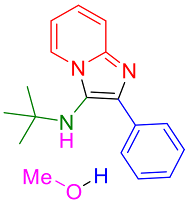

Symbolic Z-matrix:

Charge = 0 Multiplicity = 1

C	0.35587	-0.84138	-0.30128	H	-3.02595	1.17187	0.47683
C	-0.52951	0.18993	0.00544	H	-4.8787	-2.5633	-0.7198
N	-0.43685	1.51187	0.44796	H	-5.10755	-0.2032	0.11212
C	-0.21385	2.6007	-0.53508	N	-1.78701	-0.37937	-0.14069
N	-0.31401	-1.98471	-0.64452	O	0.47427	0.07066	2.90811
C	-1.36405	2.59705	-1.54463	H	0.14101	1.58647	1.2813
H	-1.21092	3.38371	-2.28928	C	-0.7423	-0.65701	3.00893
H	-1.42045	1.63942	-2.07385	H	-0.80245	-1.45281	2.25657
H	-2.32115	2.77896	-1.04695	H	-0.86601	-1.08875	4.00849
C	1.11709	2.45912	-1.28478	H	-1.55312	0.05284	2.82754
H	1.17455	1.49553	-1.80137	C	1.83116	-0.85186	-0.27424
H	1.21846	3.25442	-2.03084	C	2.5838	0.04538	0.49572
H	1.96552	2.52694	-0.5975	C	2.5093	-1.81847	-1.03067
C	-0.22609	3.90411	0.26262	C	3.97605	-0.01306	0.49671
H	0.58193	3.91326	1.00317	H	2.08265	0.79009	1.10411
H	-0.08138	4.76031	-0.40282	C	3.89936	-1.87432	-1.02902
H	-1.17776	4.02279	0.78913	H	1.92432	-2.52289	-1.61232
C	-1.60144	-1.69545	-0.54844	C	4.64028	-0.97058	-0.2673
C	-2.75062	-2.49693	-0.77529	H	4.54119	0.69066	1.10047
C	-3.02454	0.15213	0.11239	H	4.40705	-2.62683	-1.62465
C	-3.99032	-1.96386	-0.55346	H	5.72485	-1.01469	-0.26627
H	-2.60196	-3.51612	-1.11192	H	1.20534	-0.55238	2.82474
C	-4.1283	-0.61986	-0.08946				

1.6. Proposal F: GBB reaction, methanol acts as a proton shuttle in the third step

1.6.1. Step 1 - Isocyanide nucleophilic attack, forming nitrilium

- Molecular complex 1

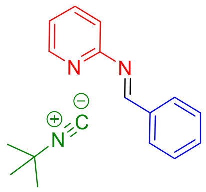

Symbolic Z-matrix:

Charge = 0 Multiplicity = 1

C	-0.33403	-1.2324	0.35932	C	-4.50063	0.42064	-0.97143
C	0.46501	1.36366	2.11397	H	-2.686	0.38412	-2.16475
N	0.92901	1.8087	1.13571	C	-5.06666	-0.01075	0.22679
C	1.51219	2.35791	-0.06581	H	-4.6812	-1.12754	2.03331
N	-1.1241	-0.9633	-0.608	H	-5.08159	1.00077	-1.68181
C	1.62943	3.87337	0.12343	H	-6.09548	0.21593	0.48286
H	2.07139	4.31709	-0.77331	N	-3.00369	-1.07918	0.84665
H	0.64392	4.31705	0.28705	H	-0.6718	-1.20384	1.40078
H	2.26589	4.10525	0.98142	C	1.08063	-1.55965	0.12017
C	0.58853	2.02162	-1.24008	C	1.95365	-1.6404	1.21032
H	0.48478	0.93955	-1.3577	C	1.57098	-1.76816	-1.17646
H	-0.40566	2.44823	-1.07783	C	3.30421	-1.91801	1.00985
H	1.00881	2.44235	-2.15848	H	1.57048	-1.46378	2.21208
C	2.89213	1.72071	-0.25488	C	2.918	-2.04925	-1.37438
H	2.8045	0.63664	-0.36512	H	0.87851	-1.70682	-2.01052
H	3.35697	2.13012	-1.15672	C	3.7872	-2.12217	-0.28199
H	3.53464	1.93705	0.60304	H	3.97815	-1.97189	1.85881
C	-2.46518	-0.6538	-0.29895	H	3.29511	-2.21389	-2.37894
C	-3.18119	0.08879	-1.24646	H	4.83892	-2.34053	-0.4399
C	-4.27409	-0.76037	1.09412				

- Transition state 1

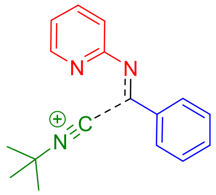

Symbolic Z-matrix:

Charge = 0 Multiplicity = 1

C	-0.98735	-0.58501	-0.55314	C	3.07865	-2.64118	0.71924
C	-0.29978	0.93852	-0.54939	H	1.26958	-2.69768	1.8984
N	0.53241	1.67094	-0.2089	C	3.65837	-2.1608	-0.46895
C	1.72129	2.33182	0.29435	H	3.25382	-1.02647	-2.24916
N	-0.27797	-1.26165	0.39904	H	3.66559	-3.23362	1.41568
C	2.66852	2.51662	-0.89326	H	4.69148	-2.36497	-0.72511
H	3.59521	2.97501	-0.53746	N	1.57049	-1.11048	-1.06643
H	2.89876	1.54665	-1.3412	H	-0.80299	-0.85326	-1.60468
H	2.22142	3.16637	-1.65014	C	-2.46064	-0.39342	-0.26582
C	2.3341	1.40476	1.3513	C	-3.28196	0.19984	-1.2263
H	1.60173	1.16406	2.12649	C	-3.00568	-0.82206	0.94236
H	2.67952	0.47311	0.89464	C	-4.64152	0.37017	-0.98132
H	3.18581	1.91439	1.81155	H	-2.85192	0.5286	-2.17075
C	1.29824	3.67233	0.89672	C	-4.3684	-0.65195	1.1877
H	0.60746	3.52095	1.73006	H	-2.34783	-1.29359	1.66423
H	2.18644	4.19012	1.2679	C	-5.18717	-0.05597	0.23049
H	0.81489	4.30108	0.14459	H	-5.27516	0.82917	-1.73382
C	1.00514	-1.574	0.07448	H	-4.79208	-0.99025	2.1284
C	1.76137	-2.35394	0.99509	H	-6.24777	0.07236	0.42355
C	2.85208	-1.41468	-1.31342				

- Molecular complex 2

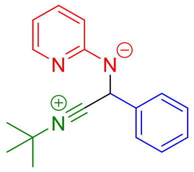

Symbolic Z-matrix:

Charge = 0 Multiplicity = 1

C	-0.92653	-0.59806	-0.58246	N	0.42566	1.64131	-0.17274
C	-0.26468	0.77003	-0.49059	C	1.56299	2.40667	0.31723

N	-0.22198	-1.35551	0.37738	C	3.75896	-2.05377	-0.47024
C	2.4926	2.6265	-0.87812	H	3.33665	-0.84114	-2.19115
H	3.38319	3.16037	-0.53589	H	3.77567	-3.22739	1.35585
H	2.79242	1.66362	-1.29946	H	4.80175	-2.20832	-0.72095
H	1.99988	3.22232	-1.65087	N	1.64057	-1.04787	-1.04116
C	2.23518	1.54313	1.39118	H	-0.77017	-0.89892	-1.63292
H	1.52922	1.29847	2.18906	C	-2.4114	-0.43946	-0.28775
H	2.61119	0.61214	0.95669	C	-3.2328	0.22089	-1.20367
H	3.07203	2.10486	1.81643	C	-2.95402	-0.96082	0.8838
C	1.04195	3.72507	0.88662	C	-4.5938	0.36561	-0.94904
H	0.35843	3.54463	1.7201	H	-2.80616	0.61634	-2.12395
H	1.88949	4.31129	1.25076	C	-4.31897	-0.81808	1.13604
H	0.52162	4.30286	0.11844	H	-2.29178	-1.47869	1.56871
C	1.06948	-1.59397	0.06637	C	-5.13901	-0.1551	0.22525
C	1.84899	-2.39866	0.95502	H	-5.22849	0.87632	-1.66648
C	2.9373	-1.29344	-1.28308	H	-4.74284	-1.2291	2.04718
C	3.17693	-2.61882	0.68336	H	-6.2007	-0.04784	0.42491
H	1.35992	-2.81358	1.82953				

1.6.2. Step 2 – Ring closure

- Molecular complex 3

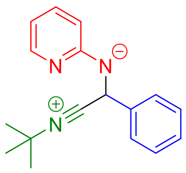

Symbolic Z-matrix:

Charge = 0 Multiplicity = 1

C	-0.92666	-0.59782	-0.58222	C	2.23545	1.54288	1.39105
C	-0.26477	0.77042	-0.4904	H	1.52944	1.29839	2.18895
N	0.42551	1.64177	-0.17251	H	2.61125	0.61179	0.95661
C	1.56348	2.40656	0.3171	H	3.07242	2.10444	1.81629
N	-0.22212	-1.35518	0.37757	C	1.04334	3.72537	0.88637
C	2.49285	2.6256	-0.87856	H	0.36019	3.54549	1.72027
H	3.38402	3.15872	-0.53662	H	1.89133	4.31135	1.24985
H	2.79171	1.66246	-1.29998	H	0.52284	4.30313	0.11828
H	2.00037	3.22185	-1.65113	C	1.06931	-1.59388	0.06649

C	1.84862	-2.39888	0.95502
C	2.93712	-1.29367	-1.283
C	3.17651	-2.6193	0.68331
H	1.35943	-2.81384	1.82944
C	3.75862	-2.05426	-0.47025
H	3.33655	-0.84141	-2.19106
H	3.77513	-3.22812	1.35568
H	4.80136	-2.20901	-0.72103
N	1.64045	-1.0478	-1.04099
C	-2.41151	-0.43931	-0.28767
C	-3.23304	0.22027	-1.20403
C	-2.95408	-0.95987	0.88427
C	-4.59405	0.36505	-0.94948
H	-2.80646	0.6151	-2.12461
C	-4.31902	-0.81705	1.13648
H	-2.29181	-1.47725	1.56953
C	-5.13917	-0.15482	0.22523
H	-5.2288	0.87515	-1.66729
H	-4.74281	-1.22744	2.04794
H	-6.20086	-0.04753	0.42485
H	-0.77006	-0.89852	-1.6327

- Transition state 2

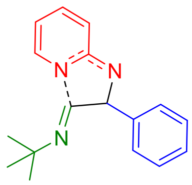

Symbolic Z-matrix:

Charge = 0 Multiplicity = 1

C	-0.75635	-0.86588	-0.66103
C	-0.10472	0.48324	-0.50029
N	0.17479	1.54263	-0.11452
C	1.07024	2.60681	0.32053
N	-0.07106	-1.75679	0.21613
C	1.92225	2.99593	-0.88978
H	2.62921	3.77398	-0.58928
H	2.47964	2.12874	-1.25189
H	1.29623	3.38345	-1.69778
C	1.93308	2.01089	1.43801
H	1.30942	1.69022	2.27629
H	2.49804	1.15036	1.06783
H	2.63123	2.7757	1.78984
C	0.22399	3.77686	0.81731
H	-0.40581	3.47023	1.65604
H	0.88677	4.57912	1.15202
H	-0.41492	4.16157	0.01822
C	1.24977	-1.73135	0.01881
C	2.11499	-2.60692	0.75637
C	3.12232	-0.77949	-1.01258
C	3.46979	-2.52406	0.57773
H	1.66079	-3.31032	1.4454
C	4.01215	-1.58404	-0.33266
H	3.48466	-0.04902	-1.73611
H	4.13073	-3.18265	1.13491
H	5.07866	-1.50416	-0.50525
N	1.78715	-0.83721	-0.86165
C	-2.23373	-0.69457	-0.33099
C	-3.07739	-0.0503	-1.23796
C	-2.74364	-1.17315	0.8741
C	-4.42716	0.12159	-0.9415
H	-2.67834	0.31043	-2.18431
C	-4.09636	-1.00318	1.16818
H	-2.06701	-1.68671	1.54846
C	-4.93833	-0.35515	0.26583
H	-5.07962	0.61849	-1.65269

H	-4.49491	-1.38326	2.10387	H	-0.6352	-1.10274	-1.73365
H	-5.99114	-0.22736	0.49783				

- Molecular complex 4

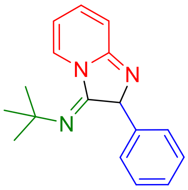

Symbolic Z-matrix:

Charge = 0 Multiplicity = 1

C	-0.65293	-0.66141	-1.19445	C	3.01803	-2.87686	0.42934
C	0.40263	0.24212	-0.52806	H	1.60307	-4.04136	-0.68729
N	0.26888	1.49338	-0.50199	C	3.35036	-1.56907	0.93791
C	1.08285	2.57452	0.05015	H	2.7387	0.47235	1.02688
N	-0.11337	-2.01001	-1.19827	H	3.68272	-3.70935	0.63743
C	2.47887	2.62403	-0.59158	H	4.23968	-1.41143	1.5339
H	3.03697	3.47975	-0.19818	N	1.39358	-0.68984	-0.08296
H	3.07539	1.72595	-0.42739	C	-1.9929	-0.5575	-0.47992
H	2.37481	2.75416	-1.67281	C	-2.74946	0.61269	-0.60329
C	1.1133	2.51128	1.58572	C	-2.47034	-1.60129	0.31229
H	0.08963	2.55928	1.96816	C	-3.96437	0.73709	0.06447
H	1.56272	1.59938	1.98156	H	-2.372	1.42739	-1.21406
H	1.6694	3.36676	1.98235	C	-3.68865	-1.47437	0.98135
C	0.35105	3.86886	-0.33925	H	-1.89107	-2.51563	0.38662
H	-0.66269	3.86136	0.07105	C	-4.4376	-0.30686	0.8609
H	0.88237	4.74702	0.04185	H	-4.54576	1.64822	-0.04007
H	0.27746	3.94511	-1.4277	H	-4.0532	-2.29389	1.59332
C	1.00035	-1.96692	-0.56406	H	-5.38709	-0.21076	1.3785
C	1.89238	-3.07462	-0.29332	H	-0.78305	-0.31178	-2.22562
C	2.53613	-0.52291	0.66748				

1.6.3. Step 3 – Methanol acting as a proton shuttle

- Molecular complex 5

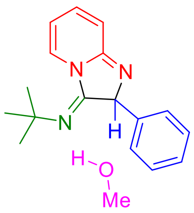

Symbolic Z-matrix:

Charge = 0 Multiplicity = 1

C	0.49088	-0.7172	1.08627	H	-2.77406	0.426	-1.32586
C	-0.41805	0.16031	0.20531	H	-4.50199	-3.15319	0.32614
N	-0.03757	1.29763	-0.19914	H	-4.65293	-1.15065	-1.21033
C	-0.66924	2.2987	-1.06534	N	-1.59628	-0.62165	0.06247
N	-0.31369	-1.84955	1.51557	O	1.70308	2.29942	1.82048
C	-1.97365	2.83316	-0.45178	H	1.29987	2.0006	0.9808
H	-2.39267	3.6134	-1.09517	C	0.65846	2.75679	2.64764
H	-2.73958	2.07287	-0.29619	H	-0.13821	2.00604	2.76135
H	-1.75513	3.27896	0.52353	H	1.07877	2.9643	3.63392
C	-0.8222	1.75981	-2.49697	H	0.20427	3.68385	2.26748
H	-1.42422	0.85316	-2.56858	C	1.72162	-1.14104	0.29898
H	-1.27458	2.52462	-3.13617	C	2.90841	-0.41515	0.41372
H	0.16707	1.51925	-2.89658	C	1.65701	-2.22434	-0.57964
C	0.32252	3.47149	-1.1262	C	4.01911	-0.76857	-0.35189
H	1.29036	3.12577	-1.5001	H	2.96123	0.42163	1.1056
H	-0.0531	4.25809	-1.78805	C	2.76688	-2.57379	-1.3454
H	0.4773	3.89953	-0.13093	H	0.74157	-2.80584	-0.64767
C	-1.43782	-1.7573	0.90963	C	3.95075	-1.84487	-1.2344
C	-2.55366	-2.67656	0.99704	H	4.94078	-0.2035	-0.25232
C	-2.7438	-0.43817	-0.68401	H	2.70994	-3.42001	-2.02331
C	-3.66854	-2.46077	0.26499	H	4.81764	-2.12033	-1.82702
H	-2.4333	-3.52461	1.66021	H	0.81722	-0.12766	1.95102
C	-3.76836	-1.31625	-0.60929				

- Transition state 3

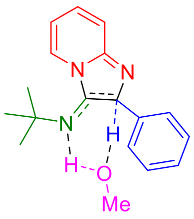

Symbolic Z-matrix:

Charge = 0 Multiplicity = 1

C	-0.69836	0.74232	0.43875	H	3.08051	-0.09344	-1.11035
C	0.44039	-0.0222	-0.0777	H	3.70128	4.08469	-0.26717
N	0.41414	-1.33782	-0.08594	H	4.53708	1.87391	-1.15862
C	1.40328	-2.31534	-0.53987	N	1.45936	0.92597	-0.22759
N	-0.29453	2.08271	0.64097	O	-0.84732	-1.25327	2.04634
C	2.67023	-2.31774	0.33764	H	-0.35331	-1.56244	1.09175
H	3.39734	-3.04155	-0.0456	C	0.13636	-1.29275	3.07322
H	3.1596	-1.34573	0.40699	H	0.97259	-0.6191	2.8401
H	2.40463	-2.61696	1.35684	H	-0.32475	-0.98356	4.01224
C	1.69858	-2.15081	-2.03949	H	0.517	-2.31361	3.17524
H	2.07772	-1.16312	-2.30636	C	-2.10296	0.54469	-0.05355
H	2.42806	-2.89798	-2.3687	C	-2.56849	-0.67327	-0.5679
H	0.77332	-2.29895	-2.6038	C	-2.99974	1.62032	0.03234
C	0.7248	-3.68254	-0.35823	C	-3.88704	-0.80088	-1.00072
H	-0.19232	-3.73227	-0.95198	H	-1.89697	-1.52136	-0.64005
H	1.39258	-4.49103	-0.67185	C	-4.31807	1.48145	-0.39087
H	0.46073	-3.84319	0.69282	H	-2.64327	2.56641	0.42324
C	0.93084	2.16196	0.25232	C	-4.77202	0.27078	-0.91269
C	1.79236	3.31318	0.2431	H	-4.22301	-1.75167	-1.40426
C	2.74923	0.85864	-0.73175	H	-4.99276	2.32938	-0.31743
C	3.05164	3.21643	-0.24446	H	-5.8001	0.16439	-1.24415
H	1.37469	4.23778	0.62378	H	-0.85284	-0.1178	1.55471
C	3.53695	1.95698	-0.75186				

- Molecular complex 6

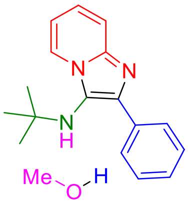

Symbolic Z-matrix:

Charge = 0 Multiplicity = 1

C	0.35587	-0.84138	-0.30128
C	-0.52951	0.18993	0.00544
N	-0.43685	1.51187	0.44796
C	-0.21385	2.6007	-0.53508
N	-0.31401	-1.98471	-0.64452
C	-1.36405	2.59705	-1.54463
H	-1.21092	3.38371	-2.28928
H	-1.42045	1.63942	-2.07385
H	-2.32115	2.77896	-1.04695
C	1.11709	2.45912	-1.28478
H	1.17455	1.49553	-1.80137
H	1.21846	3.25442	-2.03084
H	1.96552	2.52694	-0.5975
C	-0.22609	3.90411	0.26262
H	0.58193	3.91326	1.00317
H	-0.08138	4.76031	-0.40282
H	-1.17776	4.02279	0.78913
C	-1.60144	-1.69545	-0.54844
C	-2.75062	-2.49693	-0.77529
C	-3.02454	0.15213	0.11239
C	-3.99032	-1.96386	-0.55346
H	-2.60196	-3.51612	-1.11192
C	-4.1283	-0.61986	-0.08946
H	-3.02595	1.17187	0.47683
H	-4.8787	-2.5633	-0.7198
H	-5.10755	-0.2032	0.11212
N	-1.78701	-0.37937	-0.14069
O	0.47427	0.07066	2.90811
H	0.14101	1.58647	1.2813
C	-0.7423	-0.65701	3.00893
H	-0.80245	-1.45281	2.25657
H	-0.86601	-1.08875	4.00849
H	-1.55312	0.05284	2.82754
C	1.83116	-0.85186	-0.27424
C	2.5838	0.04538	0.49572
C	2.5093	-1.81847	-1.03067
C	3.97605	-0.01306	0.49671
H	2.08265	0.79009	1.10411
C	3.89936	-1.87432	-1.02902
H	1.92432	-2.52289	-1.61232
C	4.64028	-0.97058	-0.2673
H	4.54119	0.69066	1.10047
H	4.40705	-2.62683	-1.62465
H	5.72485	-1.01469	-0.26627
H	1.20534	-0.55238	2.82474

1.7. Proposal G: GBB reaction, methanol acts as a hydrogen bond donor in the first and second steps, and 2-aminopyridine acts a proton shuttle in the third step

1.7.1. Step 1 - Isocyanide nucleophilic attack, forming nitrilium (methanol as a hydrogen bond donor)

- Molecular complex 1

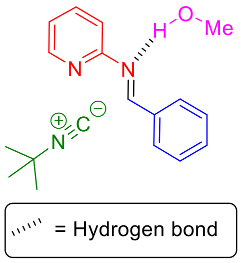

Symbolic Z-matrix:

Charge = 0 Multiplicity = 1

C	-1.1052	-1.23604	-0.64475	C	3.58465	-2.62495	-0.17026
C	0.10961	1.38295	-1.57849	H	3.10658	-2.36703	-2.26061
N	1.02702	1.77047	-0.965	H	3.68471	-2.77197	1.98282
C	2.17007	2.26661	-0.23525	H	4.61069	-2.92435	-0.35246
N	-0.35009	-1.44307	0.36885	N	1.4729	-1.93585	-1.08974
C	3.18843	2.77154	-1.262	O	-0.36969	1.00865	2.0068
H	4.06947	3.15456	-0.73919	H	-0.47381	0.13245	1.59763
H	3.49732	1.9592	-1.92534	C	-1.46897	1.82072	1.63758
H	2.76012	3.57575	-1.86627	H	-1.27258	2.82751	2.01641
C	2.74322	1.11634	0.59539	H	-1.59133	1.87332	0.54764
H	1.99869	0.78233	1.32221	H	-2.40748	1.46363	2.07948
H	3.02826	0.27874	-0.04881	C	-2.49825	-0.80261	-0.47233
H	3.63168	1.46799	1.12921	C	-3.15728	-0.19064	-1.54405
C	1.69379	3.39792	0.6798	C	-3.16817	-0.98272	0.74492
H	0.98388	3.00229	1.40972	C	-4.46613	0.25927	-1.39401
H	2.55419	3.81605	1.21077	H	-2.62368	-0.04087	-2.47852
H	1.21884	4.1923	0.09732	C	-4.4797	-0.54495	0.88764
C	0.98446	-1.85377	0.14985	H	-2.64738	-1.46983	1.56417
C	1.74562	-2.14788	1.28767	C	-5.12791	0.08139	-0.17929
C	2.74246	-2.31243	-1.23717	H	-4.96929	0.74842	-2.22181
C	3.06702	-2.53983	1.12085	H	-4.99977	-0.69002	1.82937
H	1.28692	-2.05232	2.26582	H	-6.15074	0.42664	-0.06388

H	-0.73277	-1.36044	-1.66532

- Transition state 1

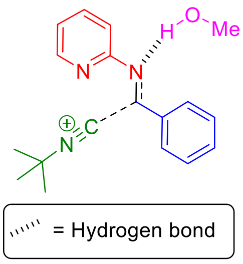

Symbolic Z-matrix:

Charge = 0 Multiplicity = 1

C	-0.79744	-0.85701	-0.79328
C	-0.13992	0.71832	-1.05099
N	0.63393	1.52235	-0.73951
C	1.68761	2.38025	-0.22996
N	-0.10901	-1.30931	0.2883
C	2.42679	2.9614	-1.43698
H	3.23706	3.6019	-1.07901
H	2.85494	2.16028	-2.04525
H	1.75394	3.56051	-2.0564
C	2.60697	1.50201	0.62768
H	2.04303	1.07803	1.46189
H	3.04307	0.69472	0.03166
H	3.41336	2.13028	1.01854
C	1.0229	3.46858	0.61506
H	0.49374	3.00732	1.45216
H	1.79953	4.13323	1.0033
H	0.3248	4.05805	0.01416
C	1.19906	-1.66194	0.06068
C	1.96104	-2.14689	1.15323
C	3.03783	-1.81944	-1.32458
C	3.28947	-2.46148	0.95946
H	1.47707	-2.24736	2.11889
C	3.85724	-2.29927	-0.31366
H	3.43268	-1.67563	-2.329
H	3.88959	-2.8298	1.78656
H	4.89665	-2.53603	-0.50956
N	1.74948	-1.49471	-1.1586
O	-0.1911	0.73763	2.1899
H	-0.22089	-0.08274	1.63951
C	-1.49487	1.26176	2.31
H	-1.43902	2.15639	2.93739
H	-1.92098	1.54602	1.33521
H	-2.18497	0.55262	2.78501
C	-2.28353	-0.66462	-0.59845
C	-3.03578	0.05275	-1.53147
C	-2.91964	-1.24907	0.4954
C	-4.4095	0.1987	-1.3662
H	-2.53826	0.50762	-2.3858
C	-4.29716	-1.10284	0.66155
H	-2.32105	-1.81089	1.20484
C	-5.04362	-0.37798	-0.26415
H	-4.98536	0.76216	-2.09366
H	-4.78618	-1.55746	1.51765
H	-6.11494	-0.26327	-0.13184
H	-0.54442	-1.29482	-1.76699

- Molecular complex 2

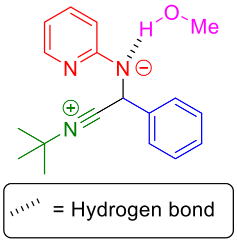

Symbolic Z-matrix:

Charge = 0 Multiplicity = 1

C	-0.68089	-0.74936	-0.90637	H	3.60622	-1.2872	-2.32529
C	-0.05647	0.63402	-0.951	H	3.97424	-2.93623	1.62646
N	0.57169	1.56292	-0.68378	H	5.04402	-2.3257	-0.58085
C	1.50862	2.50645	-0.08859	N	1.88245	-1.3015	-1.19973
N	-0.02099	-1.36234	0.19118	O	-0.38656	0.59517	2.08112
C	2.26381	3.18933	-1.22831	H	-0.28312	-0.22888	1.53331
H	2.98373	3.89099	-0.79937	C	-1.65119	0.58884	2.70668
H	2.8061	2.45181	-1.82578	H	-1.65849	1.37901	3.46323
H	1.57933	3.7436	-1.876	H	-2.46484	0.78094	1.99283
C	2.44073	1.67271	0.79962	H	-1.85424	-0.36628	3.2086
H	1.86172	1.17341	1.58104	C	-2.18431	-0.61762	-0.70999
H	2.9761	0.92406	0.20779	C	-2.92489	0.31837	-1.43391
H	3.16651	2.34837	1.26243	C	-2.83288	-1.47374	0.17829
C	0.68515	3.49324	0.74001	C	-4.30476	0.40591	-1.26684
H	0.12412	2.9467	1.50212	H	-2.42303	0.98702	-2.13122
H	1.36957	4.19524	1.2241	C	-4.21541	-1.38975	0.34015
H	-0.00363	4.05727	0.10493	H	-2.23679	-2.18543	0.73936
C	1.29362	-1.63577	-0.02744	C	-4.95348	-0.45015	-0.37709
C	2.04774	-2.23687	1.02161	H	-4.87172	1.14126	-1.829
C	3.18762	-1.56672	-1.35936	H	-4.71552	-2.05732	1.03507
C	3.3874	-2.48263	0.83246	H	-6.02861	-0.38287	-0.2437
H	1.54059	-2.47904	1.94944	H	-0.44856	-1.19745	-1.88532
C	3.99237	-2.14427	-0.39272				

1.7.2. Step 2 – Ring Closure (methanol as a hydrogen bond donor)

- Molecular complex 3

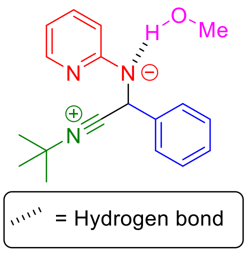

Symbolic Z-matrix:

Charge = 0 Multiplicity = 1

C	-0.72319	-0.71857	-0.93048	H	3.53375	-1.21431	-2.44827
C	-0.10586	0.66997	-0.94431	H	3.97587	-2.98538	1.44254
N	0.51246	1.59891	-0.65534	H	5.00255	-2.31104	-0.76645
C	1.44776	2.54811	-0.06717	N	1.83301	-1.2584	-1.28908
N	-0.04309	-1.35907	0.13695	O	-0.25053	0.49565	2.12547
C	2.10797	3.31728	-1.21105	H	-0.19878	-0.28491	1.50661
H	2.824	4.02609	-0.78736	C	-1.15069	0.19845	3.16842
H	2.64272	2.63438	-1.87629	H	-1.10383	1.00999	3.90023
H	1.36569	3.87437	-1.78887	H	-2.18523	0.12039	2.80642
C	2.46072	1.70788	0.72061	H	-0.88851	-0.73734	3.68042
H	1.9471	1.15026	1.50857	C	-2.22446	-0.58405	-0.71614
H	2.98229	1.00799	0.0608	C	-2.98895	0.19395	-1.58825
H	3.19095	2.38638	1.17201	C	-2.84515	-1.26701	0.32644
C	0.63912	3.45917	0.85743	C	-4.366	0.29741	-1.41522
H	0.13645	2.85306	1.61518	H	-2.50498	0.72035	-2.40941
H	1.32644	4.15758	1.34274	C	-4.22616	-1.16413	0.4983
H	-0.10048	4.03201	0.29114	H	-2.23345	-1.87402	0.98515
C	1.267	-1.62738	-0.11635	C	-4.98781	-0.38272	-0.36728
C	2.0401	-2.26147	0.8985	H	-4.95327	0.90477	-2.0967
C	3.13377	-1.52218	-1.48311	H	-4.70664	-1.69798	1.31255
C	3.37488	-2.50541	0.67508	H	-6.06162	-0.30396	-0.22949
H	1.55052	-2.5326	1.82797	H	-0.50656	-1.13632	-1.92587
C	3.95573	-2.13179	-0.55126				

- Transition state 2

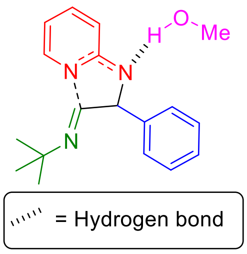

Symbolic Z-matrix:

Charge = 0 Multiplicity = 1

C	-0.49989	-0.92306	-1.05095	H	3.62744	0.24372	-1.90115
C	0.14776	0.43742	-0.90631	H	4.45418	-2.91286	0.90304
N	0.25322	1.52953	-0.52468	H	5.30496	-1.11725	-0.65637
C	0.79106	2.70971	0.12782	N	1.98844	-0.74071	-1.11801
N	0.17143	-1.79403	-0.13087	O	-0.11221	-0.04137	2.08926
C	1.13109	3.7321	-0.95702	H	-0.06237	-0.80648	1.47192
H	1.53557	4.62934	-0.48128	C	-1.09194	-0.29283	3.07354
H	1.88142	3.33171	-1.64407	H	-1.04736	0.52051	3.80332
H	0.23929	4.01054	-1.52493	H	-2.10281	-0.32092	2.64501
C	2.03408	2.25747	0.90079	H	-0.90693	-1.23598	3.60343
H	1.7577	1.48207	1.62093	C	-1.99197	-0.78879	-0.78538
H	2.79148	1.85946	0.22016	C	-2.75348	0.12908	-1.5134
H	2.45055	3.11911	1.43111	C	-2.61588	-1.60763	0.15385
C	-0.29195	3.22512	1.07798	C	-4.12393	0.23883	-1.29667
H	-0.547	2.44174	1.79586	H	-2.27214	0.76321	-2.25573
H	0.08958	4.10001	1.61194	C	-3.99054	-1.49984	0.36832
H	-1.18943	3.51277	0.52344	H	-2.01197	-2.32568	0.69818
C	1.50236	-1.69131	-0.27842	C	-4.74612	-0.57684	-0.35083
C	2.40765	-2.50632	0.47113	H	-4.70616	0.95665	-1.86601
C	3.31529	-0.54573	-1.2186	H	-4.471	-2.14229	1.1001
C	3.75593	-2.30131	0.33837	H	-5.81502	-0.49472	-0.18036
H	1.99554	-3.26028	1.13262	H	-0.32921	-1.20171	-2.10526
C	4.2438	-1.29359	-0.5284				

- Molecular complex 4

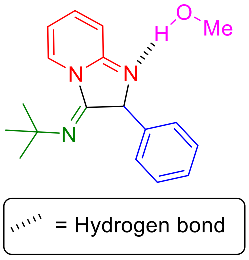

Symbolic Z-matrix:

Charge = 0 Multiplicity = 1

C	-0.53806	-0.56323	-1.56303	H	2.34175	0.08585	1.35994
C	0.53711	0.20389	-0.77058	H	3.17426	-4.09155	0.69682
N	0.59982	1.4613	-0.77822	H	3.56621	-1.96727	2.00926
C	1.61518	2.38199	-0.258	N	1.34126	-0.84405	-0.22529
N	-0.09913	-1.94887	-1.62232	O	-0.39827	-0.07501	2.19302
C	3.04283	1.95455	-0.63719	H	-0.77948	-0.74351	1.60942
H	3.73703	2.76754	-0.40202	C	-1.34064	0.98104	2.30496
H	3.39506	1.06121	-0.12156	H	-0.96635	1.66174	3.07289
H	3.095	1.76566	-1.71401	H	-1.44088	1.52703	1.35849
C	1.43984	2.5992	1.25298	H	-2.32648	0.61237	2.61041
H	1.40267	1.66641	1.81934	C	-1.90198	-0.40309	-0.90894
H	2.25578	3.22206	1.63399	C	-2.60352	0.79665	-1.06583
H	0.49716	3.1218	1.44019	C	-2.44803	-1.41606	-0.11725
C	1.33494	3.71969	-0.96061	C	-3.82975	0.9827	-0.43338
H	0.30124	4.02507	-0.77728	H	-2.17315	1.58646	-1.67521
H	2.00801	4.4997	-0.59013	C	-3.67376	-1.22435	0.52415
H	1.47474	3.6182	-2.04094	H	-1.92188	-2.36291	-0.03239
C	0.92204	-2.04572	-0.84722	C	-4.36669	-0.02588	0.36827
C	1.67963	-3.23705	-0.53181	H	-4.36784	1.91652	-0.56518
C	2.22839	-0.8447	0.82701	H	-4.09023	-2.01882	1.1361
C	2.62001	-3.19363	0.44153	H	-5.32302	0.11985	0.86087
H	1.43145	-4.13972	-1.07677	H	-0.57822	-0.13125	-2.56811
C	2.87908	-1.97949	1.1737				

1.7.3. Step 3 – Aminopyridine acting as a proton shuttle

- Molecular complex 5

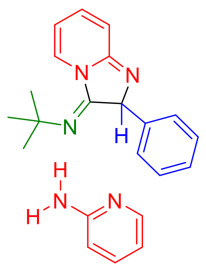

Symbolic Z-matrix:

Charge = 0 Multiplicity = 1

C	0.25104	-0.96677	-0.33058	N	2.52454	-0.49888	-0.44598
C	1.28816	0.14738	-0.15623	C	-3.0459	0.03085	-2.65472
N	0.94036	1.31918	0.17055	C	-4.3791	-0.32164	-2.4972
C	1.71346	2.54399	0.38959	C	-4.99997	0.03324	-1.2934
N	0.983	-2.18389	-0.66942	C	-4.27806	0.70569	-0.32722
C	2.38344	3.02038	-0.91018	C	-2.9211	1.00589	-0.58008
H	2.92893	3.95199	-0.72818	N	-2.32167	0.6726	-1.73067
H	3.07473	2.2988	-1.34674	H	-6.04212	-0.2165	-1.11616
H	1.60966	3.21758	-1.65752	H	-2.51621	-0.21975	-3.57169
C	2.67976	2.38488	1.57623	H	-4.91003	-0.84912	-3.28064
H	2.10092	2.18991	2.4843	H	-4.72716	0.99223	0.61856
H	3.39149	1.5659	1.46646	N	-2.15943	1.679	0.34448
H	3.24305	3.31158	1.72626	H	-2.45202	1.57389	1.3066
C	0.68483	3.61502	0.78631	H	-1.15051	1.58137	0.21886
H	0.1492	3.30705	1.68985	C	-0.64437	-1.17644	0.87609
H	1.18103	4.571	0.98238	C	-1.91949	-1.71036	0.68247
H	-0.05035	3.75481	-0.01099	C	-0.22069	-0.87933	2.17269
C	2.22135	-1.86476	-0.7204	C	-2.76835	-1.92558	1.76568
C	3.33372	-2.74087	-1.02747	H	-2.25565	-1.93687	-0.32709
C	3.8243	-0.03377	-0.4848	C	-1.06634	-1.09679	3.25921
C	4.59376	-2.25501	-1.05246	H	0.76791	-0.45855	2.33639
H	3.09282	-3.77742	-1.22995	C	-2.34534	-1.6152	3.05776
C	4.85216	-0.86177	-0.77601	H	-3.76365	-2.32557	1.5971
H	3.97413	1.01203	-0.27685	H	-0.72703	-0.85906	4.2629
H	5.42643	-2.91168	-1.28346	H	-3.00682	-1.77834	3.9031
H	5.85937	-0.46662	-0.8012	H	-0.38951	-0.67873	-1.1767

- Transition state 3

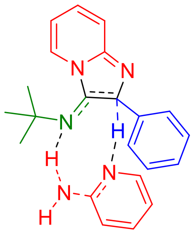

Symbolic Z-matrix:

Charge = 0 Multiplicity = 1

C	-0.00784	0.6882	-0.77736	N	2.15813	0.77349	-0.15042
C	1.11476	-0.1662	-0.38582	C	-2.01719	1.71475	1.87899
N	0.99975	-1.44499	-0.18368	C	-3.1307	1.75756	2.68639
C	1.99287	-2.4204	0.25288	C	-3.72109	0.52983	3.04092
N	0.40128	2.04485	-0.82336	C	-3.19043	-0.65796	2.59325
C	2.45166	-2.17725	1.70568	C	-2.03733	-0.63139	1.76835
H	3.17984	-2.93709	2.01108	N	-1.48457	0.55533	1.45287
H	2.89134	-1.19425	1.87527	H	-4.60621	0.51541	3.66952
H	1.58147	-2.25284	2.36595	H	-1.50703	2.60641	1.52467
C	3.15065	-2.55199	-0.75698	H	-3.53676	2.70358	3.02018
H	2.75656	-2.95266	-1.69577	H	-3.63513	-1.61152	2.85618
H	3.63352	-1.60554	-1.0014	N	-1.44665	-1.74375	1.29436
H	3.91308	-3.24394	-0.38236	H	-1.93432	-2.62136	1.37463
C	1.26511	-3.77756	0.24897	H	-0.61416	-1.68821	0.67013
H	0.8442	-3.97309	-0.74175	C	-1.12935	0.2832	-1.66479
H	1.95294	-4.58983	0.50662	C	-1.36085	-1.03974	-2.07106
H	0.4505	-3.77868	0.98089	C	-2.03701	1.27373	-2.07885
C	1.63214	2.06301	-0.43829	C	-2.45816	-1.3524	-2.87312
C	2.49409	3.20309	-0.28252	H	-0.66863	-1.81559	-1.76527
C	3.479	0.63263	0.23492	C	-3.13367	0.95269	-2.87165
C	3.7815	3.03462	0.1024	H	-1.85319	2.30206	-1.78477
H	2.06463	4.17371	-0.50207	C	-3.35454	-0.36491	-3.2746
C	4.28666	1.71094	0.36422	H	-2.60892	-2.38157	-3.18728
H	3.83257	-0.36592	0.42357	H	-3.81681	1.73812	-3.18218
H	4.44086	3.88928	0.21036	H	-4.20914	-0.61528	-3.89542
H	5.31516	1.5596	0.66715	H	-0.6837	0.61794	0.44227

- Molecular complex 6

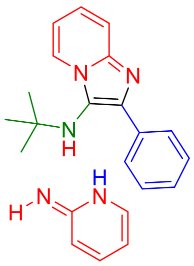

Symbolic Z-matrix:

Charge = 0 Multiplicity = 1

C	0.44059	0.42882	1.06703	N	1.72982	-1.20874	0.36787
C	1.11136	-0.03752	-0.0646	C	-2.77593	-1.93008	0.63088
N	1.27026	0.36201	-1.38925	C	-3.93595	-2.5372	0.26231
C	2.22178	1.44299	-1.72685	C	-4.34382	-2.40182	-1.10113
N	0.63476	-0.39801	2.14293	C	-3.60795	-1.68734	-1.99482
C	2.23836	1.52724	-3.25334	C	-2.37521	-1.0285	-1.60214
H	2.93196	2.30491	-3.58698	N	-2.03907	-1.22814	-0.26965
H	2.54457	0.56996	-3.6858	H	-5.26312	-2.87809	-1.42982
H	1.24126	1.77445	-3.63503	H	-2.3672	-1.95945	1.63494
C	3.6103	1.06062	-1.20894	H	-4.51965	-3.09624	0.98043
H	3.60063	0.92937	-0.12103	H	-3.92248	-1.58206	-3.02766
H	3.95469	0.12949	-1.6694	N	-1.58238	-0.29846	-2.32276
H	4.33155	1.84877	-1.44529	H	-1.93254	-0.23789	-3.27567
C	1.81399	2.7994	-1.13703	H	0.38285	0.39051	-1.89624
H	1.77113	2.75604	-0.04399	C	-0.41804	1.62117	1.20423
H	2.53845	3.57088	-1.42022	C	-1.21172	2.08612	0.148
H	0.82708	3.1005	-1.50157	C	-0.45687	2.28845	2.43503
C	1.41962	-1.37483	1.71062	C	-2.00992	3.21549	0.31837
C	1.94608	-2.50701	2.3863	H	-1.22222	1.5574	-0.80303
C	2.48997	-2.1023	-0.33896	C	-1.25359	3.41749	2.59894
C	2.72207	-3.39403	1.6925	H	0.14474	1.90671	3.25381
H	1.71241	-2.62855	3.43742	C	-2.02986	3.88773	1.53926
C	2.99279	-3.19198	0.30456	H	-2.62456	3.56471	-0.50584
H	2.62073	-1.86686	-1.38807	H	-1.2698	3.93127	3.55527
H	3.13432	-4.26332	2.19334	H	-2.65204	4.76815	1.6673
H	3.59224	-3.90336	-0.25001	H	-1.17698	-0.78546	0.03776

1.8. Proposal H: GBB reaction catalyzed by *p*-toluenesulfonic acid

1.8.1. Step 1 - Isocyanide nucleophilic attack, forming nitrilium

- Molecular complex 1

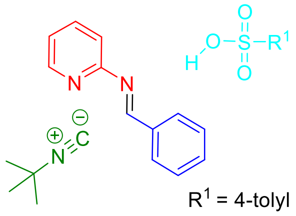

Symbolic Z-matrix:

Charge = 0 Multiplicity = 1

C	0.	0.	0.	C	5.55526	-1.99647	0.5617
H	0.	0.	1.08845	H	5.12671	-2.41768	1.47536
C	2.8086	0.	0.61272	H	6.26645	-2.71395	0.14296
N	3.54481	-0.76189	0.11949	H	6.09095	-1.07755	0.81457
C	4.45867	-1.71562	-0.47011	C	0.19465	-2.37267	0.14801
N	0.04627	-1.1535	-0.57828	C	0.20746	-3.56837	-0.57053
C	-0.06862	1.26436	-0.71022	C	0.49865	-3.40757	2.14623
C	-0.57567	2.37109	-0.01368	C	0.38956	-4.73793	0.15952
C	0.38906	1.40531	-2.02979	H	0.1132	-3.57478	-1.65177
C	-0.67811	3.60339	-0.64699	C	0.53976	-4.66378	1.54166
H	-0.89251	2.25707	1.01932	H	0.60856	-3.30275	3.22209
C	0.30027	2.64714	-2.64689	H	0.4205	-5.69298	-0.35431
H	0.85612	0.57072	-2.54681	H	0.68822	-5.55311	2.14363
C	-0.24166	3.73866	-1.96537	N	0.32853	-2.27549	1.46409
H	-1.08248	4.45715	-0.11392	S	1.1243	-1.98128	-3.79279
H	0.67116	2.76666	-3.65962	O	1.51992	-3.38224	-3.5916
H	-0.30725	4.70392	-2.45799	O	2.15216	-0.98246	-3.43346
C	5.03656	-1.09742	-1.74589	O	-0.17763	-1.64591	-3.09848
H	5.73948	-1.80624	-2.1939	H	-0.01596	-1.27239	-1.6643
H	4.23596	-0.89408	-2.46116	C	0.75823	-1.78223	-5.52502
H	5.57127	-0.17202	-1.51356	C	1.16256	-2.75538	-6.42794
C	3.66461	-2.98383	-0.7931	C	0.90611	-2.56851	-7.78689
H	3.18687	-3.37723	0.1108	C	0.2511	-1.42497	-8.24577
H	2.90585	-2.78308	-1.55419	C	-0.1495	-0.46009	-7.31062
H	4.34562	-3.74278	-1.18994	C	0.10023	-0.63104	-5.95545

H	1.66226	-3.64586	-6.06071
H	1.21804	-3.32735	-8.49945
H	-0.66764	0.43197	-7.65416
H	-0.22256	0.10893	-5.22872
C	-0.03192	-1.22169	-9.71259
H	0.3535	-2.05187	-10.30869
H	-1.10817	-1.14371	-9.89558
H	0.43118	-0.29907	-10.07593

- Transition state 1

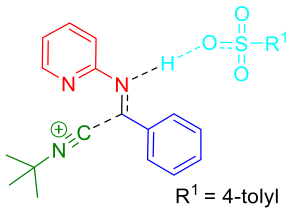

R[1] = 4-tolyl

Symbolic Z-matrix:

Charge = 0 Multiplicity = 1

C	0.	0.	0.
H	0.	0.	1.08566
C	2.0038	0.	-0.04845
N	2.94942	-0.62581	-0.28492
C	4.07621	-1.48251	-0.60352
N	-0.27934	-1.17445	-0.56419
C	-0.39996	1.25058	-0.68466
C	-1.03918	2.23789	0.06794
C	-0.15994	1.44157	-2.04912
C	-1.46935	3.41072	-0.5485
H	-1.20781	2.0859	1.1307
C	-0.58387	2.61983	-2.65481
H	0.38855	0.68814	-2.61104
C	-1.24285	3.59983	-1.91033
H	-1.97465	4.17434	0.0336
H	-0.38996	2.77706	-3.71108
H	-1.5714	4.51604	-2.39102
C	4.75931	-0.91501	-1.84945
H	5.60072	-1.56409	-2.10838
H	4.04797	-0.88718	-2.67748
H	5.14034	0.09175	-1.65544
C	3.51426	-2.88418	-0.86473
H	2.97717	-3.25209	0.01624
H	2.84401	-2.88164	-1.7283
H	4.35163	-3.55861	-1.06855
C	5.00764	-1.47041	0.61163
H	4.49202	-1.85252	1.49711
H	5.86744	-2.11221	0.40218
H	5.36792	-0.45884	0.81735
C	0.02862	-2.39096	0.07886
C	-0.11364	-3.58799	-0.63415
C	0.782	-3.45531	1.94784
C	0.2313	-4.75974	0.02134
H	-0.44786	-3.57621	-1.66613
C	0.68504	-4.70221	1.34075
H	1.13153	-3.36017	2.97266
H	0.15326	-5.70916	-0.49811
H	0.96289	-5.59816	1.88406
N	0.46783	-2.31105	1.33162
S	0.88944	-1.94442	-3.69523
O	1.3692	-3.3322	-3.61167
O	1.78819	-0.96236	-3.03408
O	-0.51713	-1.75688	-3.21298
H	-0.44474	-1.24458	-1.59489
C	0.84204	-1.51598	-5.42777
C	1.71453	-2.14187	-6.31037
C	1.71733	-1.7591	-7.65021
C	0.85598	-0.76255	-8.11728

C	-0.01614	-0.15188	-7.20832
C	-0.02942	-0.52306	-5.86758
H	2.36905	-2.92681	-5.94469
H	2.39622	-2.24608	-8.34554
H	-0.69783	0.61901	-7.55949
H	-0.71992	-0.06822	-5.16331
C	0.83743	-0.37493	-9.57433
H	1.77279	-0.64868	-10.06843
H	0.02151	-0.88257	-10.09982
H	0.68838	0.70148	-9.6951

- Molecular complex 2

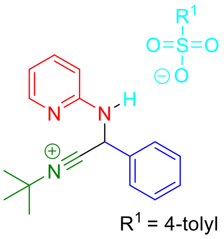

R^1 = 4-tolyl

Symbolic Z-matrix:

Charge = 0 Multiplicity = 1

C	0.	0.	0.
H	0.	0.	1.09508
C	1.46297	0.	-0.3541
N	2.55816	-0.21525	-0.61209
C	3.865	-0.70668	-1.03852
N	-0.54995	-1.23887	-0.47632
C	-0.71828	1.22359	-0.52898
C	-1.5515	1.93506	0.33491
C	-0.58542	1.61462	-1.86344
C	-2.25734	3.041	-0.13288
H	-1.65547	1.61954	1.36968
C	-1.2914	2.72455	-2.32324
H	0.06059	1.04561	-2.53051
C	-2.12557	3.43706	-1.46281
H	-2.90754	3.59098	0.53971
H	-1.18813	3.03029	-3.35965
H	-2.67373	4.29986	-1.82799
C	4.29131	0.11301	-2.25665
H	5.25721	-0.26882	-2.59834
H	3.54923	0.00443	-3.05015
H	4.40615	1.16887	-1.99549
C	3.65682	-2.18531	-1.38842
H	3.29174	-2.73971	-0.51768
H	2.94612	-2.29683	-2.21221
H	4.62524	-2.59771	-1.68711
C	4.8155	-0.52533	0.1462
H	4.46458	-1.08894	1.01483
H	5.79918	-0.90407	-0.14199
H	4.91398	0.5297	0.41549
C	0.04086	-2.41903	-0.02613
C	-0.15862	-3.61525	-0.74185
C	1.34076	-3.47092	1.54012
C	0.4385	-4.76073	-0.25376
H	-0.72751	-3.60108	-1.66532
C	1.19975	-4.70208	0.92172
H	1.9313	-3.36844	2.4477
H	0.32387	-5.69709	-0.79049
H	1.67768	-5.58333	1.3336
N	0.78827	-2.3393	1.07707
S	0.56006	-1.66555	-3.78404
O	1.2868	-2.94402	-3.81379
O	1.32902	-0.58437	-3.09653
O	-0.82716	-1.7409	-3.25057
H	-0.79869	-1.25327	-1.47556
C	0.38178	-1.13389	-5.48143
C	1.28973	-1.57015	-6.43871

C	1.17559	-1.10491	-7.74786	H	-1.54153	0.89262	-7.39769
C	0.16279	-0.21317	-8.11028	H	-1.35379	0.05454	-5.05701
C	-0.74234	0.20638	-7.12777	C	0.02012	0.26231	-9.53417
C	-0.6405	-0.24966	-5.81757	H	0.94987	0.1263	-10.09187
H	2.06275	-2.27718	-6.15435	H	-0.76539	-0.29756	-10.05285
H	1.88126	-1.44516	-8.50149	H	-0.25021	1.3212	-9.572

1.8.2. Step 2 – Ring Closure

- Molecular complex 3

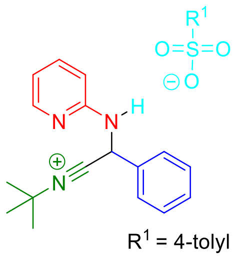

Symbolic Z-matrix:

Charge = 0 Multiplicity = 1

C	2.60538	0.38214	-0.56272	H	-0.1783	1.61848	3.0838
H	3.44825	1.0734	-0.4544	H	-0.89381	0.04006	2.71499
C	2.04663	0.30115	0.83278	C	2.19748	0.6308	4.19738
N	1.68246	0.15837	1.90905	H	2.23376	1.70596	4.0041
C	1.19591	-0.05741	3.26581	H	1.87136	0.47158	5.22809
N	1.67068	0.93291	-1.4981	H	3.19912	0.20861	4.08013
C	3.0999	-0.99743	-0.96229	C	1.15384	-1.57274	3.47807
C	4.34604	-1.09998	-1.58265	H	2.15393	-2.00696	3.38984
C	2.31799	-2.13633	-0.75562	H	0.77663	-1.76324	4.48659
C	4.81451	-2.3421	-2.0023	H	0.48345	-2.02615	2.74434
H	4.94339	-0.20688	-1.74603	C	1.21804	2.2256	-1.24827
C	2.7953	-3.3768	-1.1758	C	0.15019	2.73968	-2.00823
H	1.33765	-2.06213	-0.28671	C	1.4303	4.17741	-0.07388
C	4.03849	-3.48219	-1.79676	C	-0.2589	4.03064	-1.75339
H	5.78216	-2.41886	-2.48753	H	-0.33658	2.10923	-2.74457
H	2.18566	-4.26083	-1.02013	C	0.39627	4.78162	-0.76557
H	4.40257	-4.45155	-2.12253	H	1.9647	4.7143	0.70618
C	-0.19764	0.5642	3.36951	H	-1.08583	4.45883	-2.31164
H	-0.52035	0.47436	4.41086	H	0.10241	5.79973	-0.53866

N	1.84095	2.91828	-0.29792	C	-5.0312	0.38502	-0.31215
S	-1.09135	-0.53884	-0.14512	C	-3.64323	0.49311	-0.21539
O	-0.64258	-1.59328	0.80079	H	-2.84182	-2.80735	-0.31312
O	-0.60005	-0.7806	-1.52822	H	-5.31802	-2.98822	-0.49417
O	-0.7898	0.82936	0.33478	H	-5.64089	1.28485	-0.30439
H	0.92672	0.27712	-1.76891	H	-3.15256	1.45737	-0.12575
C	-2.87134	-0.66132	-0.22082	C	-7.15004	-0.98331	-0.52121
C	-3.46526	-1.91853	-0.3209	H	-7.43716	-1.486	-1.45007
C	-4.84805	-2.01054	-0.418	H	-7.55449	-1.5711	0.30881
C	-5.65071	-0.86141	-0.41617	H	-7.62896	-0.00154	-0.50578

- Transition state 2

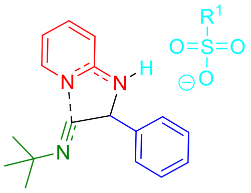

R^1 = 4-tolyl

Symbolic Z-matrix:

Charge = 0 Multiplicity = 1

C	-2.74453	-0.02454	-0.99102	H	-0.18305	-2.10489	3.78899
H	-3.68732	-0.52576	-1.24739	H	-0.73001	-2.89034	2.291
C	-2.28887	-0.73884	0.26404	H	0.29615	-1.4443	2.19503
N	-2.12932	-0.74069	1.41854	C	-2.94132	-1.74244	3.43317
C	-1.74624	-1.03373	2.78816	H	-3.16771	-2.67791	2.91337
N	-1.7862	-0.2821	-2.0487	H	-2.68952	-1.9731	4.47158
C	-2.99727	1.44616	-0.75055	H	-3.82838	-1.10331	3.42297
C	-4.19343	2.00414	-1.20424	C	-1.45091	0.30279	3.4737
C	-2.04541	2.24542	-0.1114	H	-2.33378	0.94825	3.45209
C	-4.44377	3.36297	-1.02581	H	-1.18011	0.10997	4.51584
H	-4.92735	1.3769	-1.70391	H	-0.6207	0.79926	2.96653
C	-2.30367	3.60337	0.0654	C	-1.23591	-1.55448	-1.93324
H	-1.11017	1.81661	0.24696	C	-0.25265	-2.04895	-2.80309
C	-3.49726	4.16336	-0.3889	C	-1.17881	-3.47313	-0.63577
H	-5.37403	3.79303	-1.38288	C	0.25386	-3.30318	-2.54122
H	-1.56367	4.22333	0.56129	H	0.10548	-1.42903	-3.61616
H	-3.68976	5.22232	-0.247	C	-0.21627	-4.04139	-1.44119
C	-0.50492	-1.92736	2.7584	H	-1.57452	-3.9801	0.2399

H	1.03063	-3.71532	-3.17712
H	0.17349	-5.02664	-1.21582
N	-1.68742	-2.2586	-0.89976
S	1.22454	0.12761	-0.11713
O	0.60121	0.78993	1.05784
O	0.77204	0.72758	-1.40107
O	1.09952	-1.34448	-0.06382
H	-1.038	0.42041	-2.08846
C	2.97519	0.48282	-0.02838
C	3.45001	1.70567	-0.49855
C	4.80432	1.99858	-0.38723
C	5.69646	1.08434	0.18798
C	5.19632	-0.13517	0.64887
C	3.8396	-0.4419	0.54363
H	2.7577	2.40463	-0.95754
H	5.18064	2.94999	-0.75598
H	5.8769	-0.85744	1.09253
H	3.44682	-1.39432	0.88556
C	7.16467	1.41424	0.28718
H	7.61908	1.47715	-0.70695
H	7.31794	2.37906	0.77999
H	7.70368	0.65283	0.85591

- Molecular complex 4

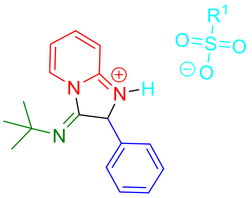

R1 = 4-tolyl

Symbolic Z-matrix:

Charge = 0 Multiplicity = 1

C	-1.92057	1.17194	-1.42525
H	-2.77452	1.61347	-1.95812
C	-2.43777	0.60625	-0.09957
N	-2.50864	1.25313	0.95625
C	-2.73987	0.94735	2.37222
N	-1.50389	-0.03373	-2.15651
C	-0.83954	2.2143	-1.28876
C	-0.96844	3.42686	-1.9651
C	0.29142	1.97236	-0.50272
C	0.0259	4.39944	-1.8628
H	-1.84919	3.61439	-2.57433
C	1.28379	2.94346	-0.40508
H	0.38715	1.02429	0.02265
C	1.15342	4.1571	-1.08233
H	-0.082	5.34099	-2.39181
H	2.16289	2.74549	0.20132
H	1.92988	4.91159	-1.001
C	-2.01717	-0.33474	2.82598
H	-1.80374	-0.25783	3.89615
H	-2.60712	-1.24196	2.68702
H	-1.07167	-0.46034	2.28768
C	-4.24515	0.91689	2.66141
H	-4.76122	0.11578	2.12437
H	-4.40455	0.76359	3.73289
H	-4.70705	1.86583	2.37452
C	-2.12132	2.13949	3.11906
H	-2.56102	3.07514	2.76442
H	-2.29136	2.04517	4.19583
H	-1.04393	2.17557	2.93337
C	-1.97355	-1.12596	-1.53095
C	-1.78954	-2.47387	-1.89632
C	-3.32046	-1.76197	0.32187
C	-2.37594	-3.43339	-1.11511
H	-1.14423	-2.70293	-2.73493

C	-3.18867	-3.07789	-0.00763
H	-3.9232	-1.40243	1.14154
H	-2.21794	-4.4805	-1.34926
H	-3.69664	-3.83114	0.58036
N	-2.69022	-0.81122	-0.42033
S	1.09311	-1.73414	-0.61611
O	0.0606	-1.12565	0.2764
O	0.99478	-1.19305	-2.00401
O	1.12696	-3.20012	-0.53939
H	-0.52602	-0.12998	-2.46435
C	2.66219	-1.13592	0.00198
C	3.28283	-0.05231	-0.60737
C	4.47217	0.44316	-0.07149
C	5.04793	-0.13534	1.06156
C	4.40514	-1.23102	1.65306
C	3.21956	-1.73466	1.1305
H	2.83717	0.38469	-1.49571
H	4.96317	1.28833	-0.54808
H	4.84579	-1.69622	2.53171
H	2.7256	-2.59137	1.57895
C	6.3472	0.38126	1.62644
H	7.16746	-0.31133	1.41055
H	6.60728	1.35189	1.19736
H	6.28845	0.49239	2.71308

1.8.3. Step 3 – Proton abstraction by tosylate

- Molecular complex 5

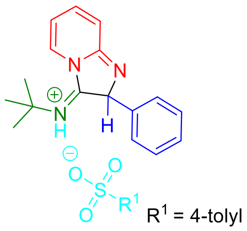

Symbolic Z-matrix:

Charge = 0 Multiplicity = 1

C	1.17114	0.92575	-0.87416
H	0.08047	0.82245	-0.91574
C	1.70934	-0.26081	-0.07593
N	1.34989	-0.45904	1.15321
C	1.68208	-1.4664	2.20094
N	1.74126	0.78887	-2.20012
C	1.55102	2.23949	-0.20282
C	0.64137	2.87848	0.64157
C	2.82373	2.78124	-0.39611
C	1.01204	4.05617	1.29118
H	-0.3452	2.44782	0.79038
C	3.18872	3.95638	0.25586
H	3.5193	2.29522	-1.07499
C	2.28271	4.59543	1.1023
H	0.29999	4.5537	1.94207
H	4.17697	4.37679	0.09757
H	2.56533	5.51415	1.60676
C	3.164	-1.33967	2.57611
H	3.40274	-2.08335	3.34158
H	3.85419	-1.48096	1.74281
H	3.35377	-0.34577	2.99128
C	1.23772	-2.87056	1.766
H	1.65031	-3.19342	0.80982
H	1.54379	-3.58813	2.53377
H	0.14836	-2.89346	1.6734
C	0.83852	-1.05806	3.41637
H	-0.22835	-1.12429	3.18328
H	1.0594	-1.7364	4.24472

H	1.07947	-0.03716	3.72983	H	0.48797	0.09559	1.36767
C	2.50396	-0.23101	-2.17919	C	-3.39132	-0.45295	-0.06172
C	3.30043	-0.76714	-3.26283	C	-4.48428	-0.56087	0.79221
C	3.35769	-2.05552	-0.74711	C	-5.74349	-0.18512	0.33308
C	4.0603	-1.86277	-3.06505	C	-5.92313	0.29775	-0.96765
H	3.24142	-0.24492	-4.20987	C	-4.80891	0.38649	-1.80811
C	4.08828	-2.52281	-1.77554	C	-3.54307	0.01457	-1.36285
H	3.34261	-2.52733	0.21966	H	-4.34037	-0.9529	1.79403
H	4.65725	-2.26733	-3.87539	H	-6.60347	-0.27516	0.99196
H	4.69111	-3.40943	-1.62613	H	-4.93581	0.74344	-2.82699
N	2.56953	-0.91468	-0.90883	H	-2.679	0.06063	-2.01858
S	-1.76602	-0.87923	0.53744	C	-7.28626	0.72988	-1.44581
O	-0.97568	-1.30348	-0.64016	H	-7.46822	1.78088	-1.19656
O	-1.20945	0.39909	1.1123	H	-7.37689	0.62555	-2.52993
O	-1.948	-1.90804	1.57503	H	-8.07603	0.13717	-0.97704

- Transition state 3

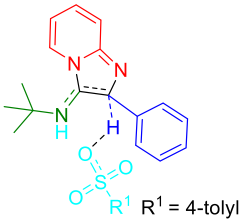

R^1 = 4-tolyl

Symbolic Z-matrix:

Charge = 0 Multiplicity = 1

C	1.71383	0.39783	-1.09387	C	3.18704	4.41895	-0.78043
H	0.42772	0.51076	-1.1362	H	1.4437	4.79496	0.42738
C	1.77757	-0.47509	0.07733	H	4.79711	3.75321	-2.04791
N	1.7001	-0.0307	1.32256	H	3.56596	5.43255	-0.69475
C	1.74744	-0.62265	2.68439	C	3.02461	-1.45508	2.85186
N	2.04751	-0.38617	-2.2332	H	3.08257	-1.81756	3.88236
C	2.2122	1.80502	-1.00397	H	3.07216	-2.32302	2.19247
C	1.50404	2.76161	-0.26511	H	3.90301	-0.83393	2.6536
C	3.39716	2.17328	-1.64957	C	0.46393	-1.38293	3.04033
C	2.00034	4.0598	-0.14544	H	0.22745	-2.20202	2.36277
H	0.54461	2.50267	0.18229	H	0.56485	-1.77778	4.05652
C	3.87871	3.47474	-1.54016	H	-0.38686	-0.69955	2.99612
H	3.92355	1.43288	-2.24388	C	1.83731	0.5977	3.61106

H	0.95309	1.23113	3.48303	O	-1.05786	-1.26882	0.37783
H	1.87406	0.26663	4.65158	H	1.55725	0.97462	1.33735
H	2.7377	1.18466	3.40214	C	-3.22861	0.17876	0.00176
C	2.07263	-1.60683	-1.83148	C	-3.81975	-0.77877	-0.82041
C	2.25842	-2.81212	-2.59481	C	-5.19238	-0.734	-1.027
C	1.71419	-2.98925	0.17042	C	-5.98224	0.25382	-0.42283
C	2.17793	-4.01503	-1.98225	C	-5.36204	1.19858	0.39697
H	2.44419	-2.6969	-3.65585	C	-3.98465	1.16922	0.61397
C	1.87119	-4.10251	-0.57291	H	-3.20502	-1.54694	-1.27901
H	1.42517	-3.00379	1.20721	H	-5.66385	-1.4767	-1.66578
H	2.31181	-4.92902	-2.55056	H	-5.96257	1.9684	0.87389
H	1.72684	-5.06692	-0.10233	H	-3.49463	1.89465	1.25496
N	1.884	-1.73942	-0.41689	C	-7.47197	0.2779	-0.6524
S	-1.46632	0.13952	0.23704	H	-7.92936	1.157	-0.19298
O	-0.91388	0.72125	-1.05777	H	-7.7027	0.29311	-1.72175
O	-1.15743	1.00917	1.38757	H	-7.94594	-0.61199	-0.22603

- Molecular complex 6

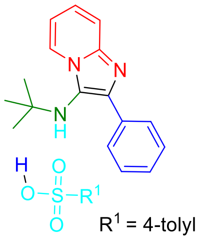

Symbolic Z-matrix:

Charge = 0 Multiplicity = 1

C	-1.57177	0.28102	0.66401	H	-2.02946	1.8264	2.78994
H	0.68056	0.18459	1.6396	C	-2.00661	4.52705	0.71859
C	-2.10541	-0.61927	-0.23917	H	-1.61296	4.4457	-1.39808
N	-2.91563	-0.53229	-1.37594	H	-2.31371	4.2968	2.83876
C	-4.38593	-0.38584	-1.19276	H	-2.13072	5.6054	0.7358
N	-0.85292	-0.36965	1.63891	C	-4.90858	-1.60385	-0.43004
C	-1.69469	1.74791	0.6709	H	-5.98797	-1.51234	-0.27738
C	-1.56505	2.49236	-0.50591	H	-4.71744	-2.52541	-0.98802
C	-1.95011	2.40934	1.87705	H	-4.43669	-1.68529	0.55524
C	-1.72609	3.87485	-0.48173	C	-4.9839	-0.35379	-2.59878
H	-1.2826	1.99264	-1.42742	H	-4.71717	-1.26056	-3.14948
C	-2.10894	3.79235	1.8996	H	-6.07395	-0.27854	-2.54818

H	-4.61441	0.51311	-3.15877	O	1.271	1.27885	-0.97294
C	-4.7561	0.89507	-0.43534	O	1.26215	-1.18269	-0.50477
H	-4.36732	1.78097	-0.94824	H	-2.56442	0.16061	-2.0287
H	-5.84462	0.98793	-0.35959	C	3.54826	0.12847	-0.33876
H	-4.34337	0.88653	0.5781	C	4.22484	-1.07721	-0.18895
C	-0.92774	-1.66862	1.34681	C	5.61389	-1.0798	-0.27231
C	-0.34656	-2.78778	1.98586	C	6.32461	0.10318	-0.50193
C	-1.84573	-3.09869	-0.36339	C	5.61302	1.29936	-0.64825
C	-0.53189	-4.0282	1.43815	C	4.22424	1.3218	-0.57107
H	0.23688	-2.62321	2.88401	H	3.66361	-1.99079	-0.02066
C	-1.28815	-4.18519	0.24066	H	6.15573	-2.01484	-0.15908
H	-2.41262	-3.10967	-1.28552	H	6.15447	2.22432	-0.82725
H	-0.09333	-4.90115	1.90923	H	3.66326	2.2427	-0.69371
H	-1.41686	-5.16252	-0.20759	C	7.82672	0.08617	-0.62315
N	-1.67356	-1.86626	0.2034	H	8.26346	1.01697	-0.25298
S	1.7811	0.14899	-0.21021	H	8.25972	-0.74479	-0.06124
O	1.63176	0.43403	1.34396	H	8.12589	-0.0277	-1.67052

1.9. Proposal I: GBB reaction with a participation of methanol in mechanism, forming a tetrahedral intermediate, catalyzed by *p*-toluenesulfonic acid

1.9.1. Step 1 - Isocyanide nucleophilic attack, forming nitrilium

- Molecular complex 1

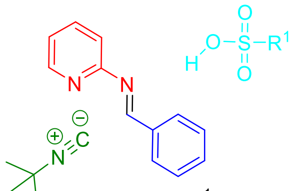

R^1 = 4-tolyl

Symbolic Z-matrix:

Charge = 0 Multiplicity = 1

C	0.	0.	0.	C	-0.06862	1.26436	-0.71022
H	0.	0.	1.08845	C	-0.57567	2.37109	-0.01368
C	2.8086	0.	0.61272	C	0.38906	1.40531	-2.02979
N	3.54481	-0.76189	0.11949	C	-0.67811	3.60339	-0.64699
C	4.45867	-1.71562	-0.47011	H	-0.89251	2.25707	1.01932
N	0.04627	-1.1535	-0.57828	C	0.30027	2.64714	-2.64689

H	0.85612	0.57072	-2.54681
C	-0.24166	3.73866	-1.96537
H	-1.08248	4.45715	-0.11392
H	0.67116	2.76666	-3.65962
H	-0.30725	4.70392	-2.45799
C	5.03656	-1.09742	-1.74589
H	5.73948	-1.80624	-2.1939
H	4.23596	-0.89408	-2.46116
H	5.57127	-0.17202	-1.51356
C	3.66461	-2.98383	-0.7931
H	3.18687	-3.37723	0.1108
H	2.90585	-2.78308	-1.55419
H	4.34562	-3.74278	-1.18994
C	5.55526	-1.99647	0.5617
H	5.12671	-2.41768	1.47536
H	6.26645	-2.71395	0.14296
H	6.09095	-1.07755	0.81457
C	0.19465	-2.37267	0.14801
C	0.20746	-3.56837	-0.57053
C	0.49865	-3.40757	2.14623
C	0.38956	-4.73793	0.15952
H	0.1132	-3.57478	-1.65177
C	0.53976	-4.66378	1.54166
H	0.60856	-3.30275	3.22209
H	0.4205	-5.69298	-0.35431
H	0.68822	-5.55311	2.14363
N	0.32853	-2.27549	1.46409
S	1.1243	-1.98128	-3.79279
O	1.51992	-3.38224	-3.5916
O	2.15216	-0.98246	-3.43346
O	-0.17763	-1.64591	-3.09848
H	-0.01596	-1.27239	-1.6643
C	0.75823	-1.78223	-5.52502
C	1.16256	-2.75538	-6.42794
C	0.90611	-2.56851	-7.78689
C	0.2511	-1.42497	-8.24577
C	-0.1495	-0.46009	-7.31062
C	0.10023	-0.63104	-5.95545
H	1.66226	-3.64586	-6.06071
H	1.21804	-3.32735	-8.49945
H	-0.66764	0.43197	-7.65416
H	-0.22256	0.10893	-5.22872
C	-0.03192	-1.22169	-9.71259
H	0.3535	-2.05187	-10.30869
H	-1.10817	-1.14371	-9.89558
H	0.43118	-0.29907	-10.07593

- Transition state 1

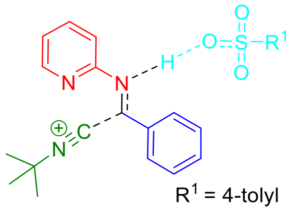

Symbolic Z-matrix:

Charge = 0 Multiplicity = 1

C	0.	0.	0.
H	0.	0.	1.08566
C	2.0038	0.	-0.04845
N	2.94942	-0.62581	-0.28492
C	4.07621	-1.48251	-0.60352
N	-0.27934	-1.17445	-0.56419
C	-0.39996	1.25058	-0.68466
C	-1.03918	2.23789	0.06794
C	-0.15994	1.44157	-2.04912
C	-1.46935	3.41072	-0.5485

H	-1.20781	2.0859	1.1307
C	-0.58387	2.61983	-2.65481
H	0.38855	0.68814	-2.61104
C	-1.24285	3.59983	-1.91033
H	-1.97465	4.17434	0.0336
H	-0.38996	2.77706	-3.71108
H	-1.5714	4.51604	-2.39102
C	4.75931	-0.91501	-1.84945
H	5.60072	-1.56409	-2.10838
H	4.04797	-0.88718	-2.67748
H	5.14034	0.09175	-1.65544
C	3.51426	-2.88418	-0.86473
H	2.97717	-3.25209	0.01624
H	2.84401	-2.88164	-1.7283
H	4.35163	-3.55861	-1.06855
C	5.00764	-1.47041	0.61163
H	4.49202	-1.85252	1.49711
H	5.86744	-2.11221	0.40218
H	5.36792	-0.45884	0.81735
C	0.02862	-2.39096	0.07886
C	-0.11364	-3.58799	-0.63415
C	0.782	-3.45531	1.94784
C	0.2313	-4.75974	0.02134
H	-0.44786	-3.57621	-1.66613
C	0.68504	-4.70221	1.34075
H	1.13153	-3.36017	2.97266
H	0.15326	-5.70916	-0.49811
H	0.96289	-5.59816	1.88406
N	0.46783	-2.31105	1.33162
S	0.88944	-1.94442	-3.69523
O	1.3692	-3.3322	-3.61167
O	1.78819	-0.96236	-3.03408
O	-0.51713	-1.75688	-3.21298
H	-0.44474	-1.24458	-1.59489
C	0.84204	-1.51598	-5.42777
C	1.71453	-2.14187	-6.31037
C	1.71733	-1.7591	-7.65021
C	0.85598	-0.76255	-8.11728
C	-0.01614	-0.15188	-7.20832
C	-0.02942	-0.52306	-5.86758
H	2.36905	-2.92681	-5.94469
H	2.39622	-2.24608	-8.34554
H	-0.69783	0.61901	-7.55949
H	-0.71992	-0.06822	-5.16331
C	0.83743	-0.37493	-9.57433
H	1.77279	-0.64868	-10.06843
H	0.02151	-0.88257	-10.09982
H	0.68838	0.70148	-9.6951

- Molecular complex 2

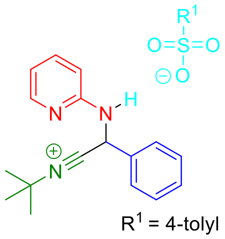

R[1] = 4-tolyl

Symbolic Z-matrix:

Charge = 0 Multiplicity = 1

C	0.	0.	0.
H	0.	0.	1.09508
C	1.46297	0.	-0.3541
N	2.55816	-0.21525	-0.61209
C	3.865	-0.70668	-1.03852
N	-0.54995	-1.23887	-0.47632
C	-0.71828	1.22359	-0.52898
C	-1.5515	1.93506	0.33491

C	-0.58542	1.61462	-1.86344	H	-0.72751	-3.60108	-1.66532
C	-2.25734	3.041	-0.13288	C	1.19975	-4.70208	0.92172
H	-1.65547	1.61954	1.36968	H	1.9313	-3.36844	2.4477
C	-1.2914	2.72455	-2.32324	H	0.32387	-5.69709	-0.79049
H	0.06059	1.04561	-2.53051	H	1.67768	-5.58333	1.3336
C	-2.12557	3.43706	-1.46281	N	0.78827	-2.3393	1.07707
H	-2.90754	3.59098	0.53971	S	0.56006	-1.66555	-3.78404
H	-1.18813	3.03029	-3.35965	O	1.2868	-2.94402	-3.81379
H	-2.67373	4.29986	-1.82799	O	1.32902	-0.58437	-3.09653
C	4.29131	0.11301	-2.25665	O	-0.82716	-1.7409	-3.25057
H	5.25721	-0.26882	-2.59834	H	-0.79869	-1.25327	-1.47556
H	3.54923	0.00443	-3.05015	C	0.38178	-1.13389	-5.48143
H	4.40615	1.16887	-1.99549	C	1.28973	-1.57015	-6.43871
C	3.65682	-2.18531	-1.38842	C	1.17559	-1.10491	-7.74786
H	3.29174	-2.73971	-0.51768	C	0.16279	-0.21317	-8.11028
H	2.94612	-2.29683	-2.21221	C	-0.74234	0.20638	-7.12777
H	4.62524	-2.59771	-1.68711	C	-0.6405	-0.24966	-5.81757
C	4.8155	-0.52533	0.1462	H	2.06275	-2.27718	-6.15435
H	4.46458	-1.08894	1.01483	H	1.88126	-1.44516	-8.50149
H	5.79918	-0.90407	-0.14199	H	-1.54153	0.89262	-7.39769
H	4.91398	0.5297	0.41549	H	-1.35379	0.05454	-5.05701
C	0.04086	-2.41903	-0.02613	C	0.02012	0.26231	-9.53417
C	-0.15862	-3.61525	-0.74185	H	0.94987	0.1263	-10.09187
C	1.34076	-3.47092	1.54012	H	-0.76539	-0.29756	-10.05285
C	0.4385	-4.76073	-0.25376	H	-0.25021	1.3212	-9.572

1.9.2. Step 2 – Methanol addition to nitrilium

- Molecular complex 3

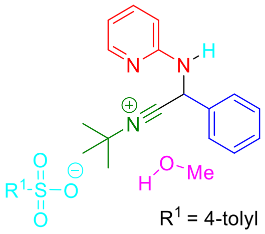

Symbolic Z-matrix:

Charge = 0 Multiplicity = 1

```
C
H 1 B1
C 1 B2 2 A1
N 3 B3 1 A2 2 D1 0
C 4 B4 3 A3 1 D2 0
N 1 B5 3 A4 4 D3 0
C 1 B6 6 A5 3 D4 0
C 7 B7 1 A6 6 D5 0
C 7 B8 1 A7 6 D6 0
C 8 B9 7 A8 1 D7 0
H 8 B10 7 A9 1 D8 0
C 9 B11 7 A10 1 D9 0
H 9 B12 7 A11 1 D10 0
C 12 B13 9 A12 7 D11 0
H 10 B14 8 A13 7 D12 0
H 12 B15 9 A14 7 D13 0
H 14 B16 12 A15 9 D14 0
C 5 B17 4 A16 3 D15 0
H 18 B18 5 A17 4 D16 0
H 18 B19 5 A18 4 D17 0
H 18 B20 5 A19 4 D18 0
C 5 B21 4 A20 3 D19 0
H 22 B22 5 A21 4 D20 0
H 22 B23 5 A22 4 D21 0
H 22 B24 5 A23 4 D22 0
C 5 B25 4 A24 3 D23 0
H 26 B26 5 A25 4 D24 0
H 26 B27 5 A26 4 D25 0
H 26 B28 5 A27 4 D26 0
C 6 B29 1 A28 3 D27 0
C 30 B30 6 A29 1 D28 0
C 30 B31 6 A30 1 D29 0
C 31 B32 30 A31 6 D30 0
H 31 B33 30 A32 6 D31 0
C 32 B34 30 A33 6 D32 0
H 32 B35 30 A34 6 D33 0
H 33 B36 31 A35 30 D34 0
H 35 B37 32 A36 30 D35 0
N 30 B38 6 A37 1 D36 0
S 9 B39 7 A38 1 D37 0
O 40 B40 9 A39 7 D38 0
O 40 B41 9 A40 7 D39 0
O 40 B42 9 A41 7 D40 0
H 6 B43 1 A42 3 D41 0
C 40 B44 9 A43 7 D42 0
C 45 B45 40 A44 9 D43 0
C 46 B46 45 A45 40 D44 0
C 47 B47 46 A46 45 D45 0
C 48 B48 47 A47 46 D46 0
C 45 B49 40 A48 9 D47 0
H 46 B50 45 A49 40 D48 0
H 47 B51 46 A50 45 D49 0
H 49 B52 48 A51 47 D50 0
H 50 B53 45 A52 40 D51 0
C 48 B54 47 A53 46 D52 0
H 55 B55 48 A54 47 D53 0
H 55 B56 48 A55 47 D54 0
H 55 B57 48 A56 47 D55 0
C 42 B58 40 A57 9 D56 0
H 59 B59 42 A58 40 D57 0
H 59 B60 42 A59 40 D58 0
H 59 B61 42 A60 40 D59 0
O 59 B62 42 A61 40 D60 0
H 63 B63 59 A62 42 D61 0
Variables:
B1 1.09679
B2 1.49337
B3 1.14552
B4 1.4587
B5 1.44482
B6 1.51506
B7 1.39912
B8 1.39023
B9 1.39017
```

B10	1.08723	B47	1.40107
B11	1.39603	B48	1.39616
B12	1.0881	B49	1.38855
B13	1.39106	B50	1.08655
B14	1.08547	B51	1.08745
B15	1.08455	B52	1.08699
B16	1.08553	B53	1.0852
B17	1.5292	B54	1.50795
B18	1.09365	B55	1.09261
B19	1.09391	B56	1.09521
B20	1.09149	B57	1.0937
B21	1.53317	B58	3.25009
B22	1.09295	B59	1.09451
B23	1.09424	B60	1.0964
B24	1.09319	B61	1.0963
B25	1.52878	B62	1.42125
B26	1.09368	B63	0.98391
B27	1.09388	A1	102.84166
B28	1.09142	A2	166.41582
B29	1.38614	A3	173.67929
B30	1.40842	A4	108.81896
B31	2.28807	A5	110.63775
B32	1.38178	A6	117.22861
B33	1.08516	A7	122.22946
B34	1.38611	A8	119.66521
B35	1.08731	A9	119.83912
B36	1.08554	A10	119.56417
B37	1.08331	A11	121.37984
B38	1.33211	A12	120.15238
B39	3.62197	A13	119.78523
B40	1.48805	A14	118.36802
B41	1.49314	A15	120.04456
B42	1.4716	A16	108.04461
B43	1.0112	A17	107.79562
B44	1.78815	A18	110.72172
B45	1.39328	A19	110.04828
B46	1.3897	A20	105.36343

A21	110.75435	A56	111.17548
A22	111.19961	A57	102.3915
A23	108.17653	A58	156.48317
A24	107.34496	A59	67.66486
A25	110.59777	A60	93.93345
A26	108.00956	A61	55.83793
A27	110.83846	A62	105.74469
A28	121.42821	D1	99.89658
A29	120.22361	D2	-67.1422
A30	147.80974	D3	-16.58827
A31	117.94379	D4	125.51118
A32	120.44341	D5	64.17809
A33	92.82313	D6	-116.96803
A34	146.45465	D7	178.56257
A35	119.91634	D8	-0.76401
A36	120.66293	D9	-177.63066
A37	116.63579	D10	-0.17137
A38	130.87171	D11	-1.24287
A39	65.60141	D12	-179.56256
A40	103.28033	D13	176.54579
A41	58.56318	D14	179.62505
A42	115.40898	D15	157.91413
A43	151.32177	D16	-179.31469
A44	119.47413	D17	-60.87323
A45	119.47523	D18	60.76757
A46	120.9342	D19	38.31815
A47	118.5112	D20	-60.63906
A48	119.82024	D21	60.49775
A49	119.6588	D22	179.96634
A50	119.71735	D23	-80.88772
A51	119.37802	D24	61.30889
A52	119.10386	D25	-179.72894
A53	120.39378	D26	-59.11131
A54	111.20795	D27	59.86915
A55	110.76335	D28	-172.23715
D29	10.56199	D31	0.90184
D30	-178.58368	D32	177.46605

D33	-2.34594	D49	-179.57586
D34	-179.97581	D50	-179.40265
D35	-179.84467	D51	-2.17641
D36	8.56714	D52	178.92965
D37	-38.71712	D53	163.79047
D38	85.88012	D54	-76.36899
D39	-20.98192	D55	43.13836
D40	-130.8889	D56	-22.36945
D41	-93.90895	D57	50.56202
D42	164.02271	D58	-35.73417
D43	-111.75064	D59	-145.04341
D44	-178.56618	D60	102.39788
D45	0.13345	D61	6.12511
D46	-0.33766	D59	-148.75242
D47	69.60569	D60	98.72806
D48	2.35404	D61	6.03535

- Transition state 2

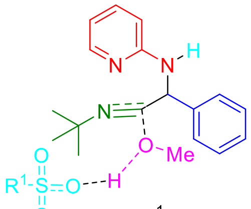

R1 = 4-tolyl

Symbolic Z-matrix:

Charge = 0 Multiplicity = 1

C	0.	0.	0.	H	0.63804	1.65473	-2.12813
H	0.	0.	1.09508	C	-2.19576	3.39656	-1.45338
C	1.45478	0.	-0.38937	H	-3.59154	2.94676	0.13036
N	2.28405	-0.59214	-0.95547	H	-0.60621	3.61365	-2.90134
C	3.48385	-1.22403	-1.46773	H	-2.74724	4.26552	-1.79849
N	-0.60847	-1.22978	-0.45997	C	3.31859	-1.36297	-2.98311
C	-0.75746	1.19598	-0.53632	H	4.20961	-1.85342	-3.38568
C	-1.94904	1.56322	0.09705	H	2.44529	-1.97929	-3.2177
C	-0.29567	1.91694	-1.63377	H	3.20526	-0.37655	-3.43775
C	-2.66955	2.65855	-0.36473	C	3.55012	-2.59884	-0.79048
H	-2.30223	0.99194	0.95227	H	3.62951	-2.49209	0.29489
C	-1.01105	3.02886	-2.08265	H	2.65953	-3.19321	-1.02008

H	4.43357	-3.12742	-1.15934	C	4.42062	3.80055	-3.15236
C	4.70027	-0.36881	-1.11283	C	5.27721	3.02062	-3.92073
H	4.77924	-0.24294	-0.0291	C	6.33659	3.63027	-4.5892
H	5.60105	-0.87317	-1.47555	C	6.54753	5.00907	-4.49774
H	4.62183	0.61834	-1.57053	C	5.66948	5.77051	-3.71773
C	-0.12317	-2.45304	-0.01984	C	4.60653	5.17579	-3.04565
C	-0.4253	-3.63306	-0.72698	H	5.10011	1.95232	-4.00119
C	1.05767	-3.61481	1.55872	H	7.00827	3.02663	-5.19421
C	0.05661	-4.83072	-0.23134	H	5.82024	6.84434	-3.63961
H	-1.02202	-3.59266	-1.63266	H	3.91425	5.76231	-2.44986
C	0.81784	-4.83468	0.94258	C	7.67582	5.67121	-5.24732
H	1.64739	-3.56138	2.4707	H	8.12289	6.47557	-4.65706
H	-0.15594	-5.75811	-0.75429	H	8.46037	4.95282	-5.49718
H	1.21279	-5.75259	1.3611	H	7.31397	6.10978	-6.18343
N	0.6095	-2.44268	1.09391	C	1.41641	2.57149	1.21311
S	3.09396	3.02934	-2.24332	H	0.56764	2.16876	1.76984
O	2.76202	1.77664	-2.95912	H	1.06121	3.26618	0.4452
O	3.69997	2.72086	-0.89526	H	2.08272	3.09302	1.9064
O	2.00112	4.00592	-2.15252	O	2.13726	1.4871	0.62092
H	-0.93431	-1.19779	-1.41783	H	2.83227	1.92162	-0.01138

- Molecular complex 4

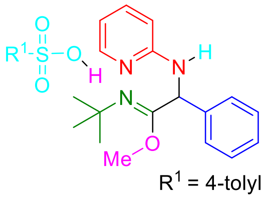

Symbolic Z-matrix:

Charge = 0 Multiplicity = 1

C								
H	1	B1						
C	1	B2	2	A1				
N	3	B3	1	A2	2	D1	0	
C	4	B4	3	A3	1	D2	0	
N	1	B5	3	A4	4	D3	0	
C	1	B6	6	A5	3	D4	0	
C	7	B7	1	A6	6	D5	0	
C	7	B8	1	A7	6	D6	0	
C	8	B9	7	A8	1	D7	0	
H	8	B10	7	A9	1	D8	0	
C	9	B11	7	A10	1	D9	0	
H	9	B12	7	A11	1	D10	0	
C	12	B13	9	A12	7	D11	0	
H	10	B14	8	A13	7	D12	0	
H	12	B15	9	A14	7	D13	0	

H 14 B16 12 A15 9 D14 0
C 5 B17 4 A16 3 D15 0
H 18 B18 5 A17 4 D16 0
H 18 B19 5 A18 4 D17 0
H 18 B20 5 A19 4 D18 0
C 5 B21 4 A20 3 D19 0
H 22 B22 5 A21 4 D20 0
H 22 B23 5 A22 4 D21 0
H 22 B24 5 A23 4 D22 0
C 5 B25 4 A24 3 D23 0
H 26 B26 5 A25 4 D24 0
H 26 B27 5 A26 4 D25 0
H 26 B28 5 A27 4 D26 0
C 6 B29 1 A28 7 D27 0
C 30 B30 6 A29 1 D28 0
C 30 B31 6 A30 1 D29 0
C 31 B32 30 A31 6 D30 0
H 31 B33 30 A32 6 D31 0
C 32 B34 30 A33 6 D32 0
H 32 B35 30 A34 6 D33 0
H 33 B36 31 A35 30 D34 0
H 35 B37 32 A36 30 D35 0
N 30 B38 6 A37 1 D36 0
S 9 B39 7 A38 1 D37 0
O 40 B40 9 A39 7 D38 0
O 40 B41 9 A40 7 D39 0
O 40 B42 9 A41 7 D40 0
H 6 B43 1 A42 7 D41 0
C 40 B44 9 A43 7 D42 0
C 45 B45 40 A44 9 D43 0
C 46 B46 45 A45 40 D44 0
C 47 B47 46 A46 45 D45 0
C 48 B48 47 A47 46 D46 0
C 49 B49 48 A48 47 D47 0
H 46 B50 45 A49 40 D48 0
H 47 B51 46 A50 45 D49 0
H 49 B52 48 A51 47 D50 0
H 50 B53 49 A52 48 D51 0
C 48 B54 47 A53 46 D52 0
H 55 B55 48 A54 47 D53 0
H 55 B56 48 A55 47 D54 0
H 55 B57 48 A56 47 D55 0
C 3 B58 1 A57 6 D56 0
H 59 B59 3 A58 1 D57 0
H 59 B60 3 A59 1 D58 0
H 59 B61 3 A60 1 D59 0
O 3 B62 1 A61 6 D60 0
H 42 B63 40 A62 9 D61 0

Variables:

B1 1.09266
B2 1.5327
B3 1.25238
B4 1.46317
B5 1.44883
B6 1.51847
B7 1.39778
B8 1.39552
B9 1.39219
B10 1.08761
B11 1.39525
B12 1.08692
B13 1.39273
B14 1.08565
B15 1.08553
B16 1.08543
B17 1.53336
B18 1.09546
B19 1.09364
B20 1.09393
B21 1.53625
B22 1.09497
B23 1.09416
B24 1.0951
B25 1.53481

B26	1.09203	B63	0.99353
B27	1.09584	A1	108.10157
B28	1.09292	A2	120.34524
B29	1.3886	A3	130.00291
B30	1.40971	A4	111.34325
B31	2.29043	A5	111.04532
B32	1.38081	A6	119.74538
B33	1.08532	A7	120.82143
B34	1.38651	A8	120.31025
B35	1.0878	A9	119.40647
B36	1.08592	A10	120.3214
B37	1.0835	A11	120.21953
B38	1.33485	A12	119.9976
B39	3.6876	A13	119.86009
B40	1.45793	A14	119.4659
B41	1.5973	A15	120.06495
B42	1.44704	A16	106.16089
B43	1.0135	A17	110.06751
B44	1.77296	A18	110.05045
B45	1.38933	A19	110.24235
B46	1.39405	A20	104.51432
B47	1.39651	A21	110.35474
B48	1.40214	A22	110.15745
B49	1.3883	A23	110.28825
B50	1.08568	A24	117.38606
B51	1.08626	A25	111.9514
B52	1.08677	A26	108.69283
B53	1.08548	A27	111.20915
B54	1.50674	A28	117.46458
B55	1.09451	A29	119.90521
B56	1.09382	A30	148.60599
B57	1.09195	A31	118.46134
B58	2.43106	A32	119.99603
B59	1.09312	A33	93.12718
B60	1.09408	A34	146.47917
B61	1.09016	A35	119.99595
B62	1.38735	A36	120.74487

A37	117.52261	D12	-179.80039
A38	128.64647	D13	177.90119
A39	58.82902	D14	179.46888
A40	72.30251	D15	-131.95257
A41	89.52304	D16	-174.03519
A42	112.50587	D17	-54.22928
A43	161.83977	D18	65.60379
A44	119.53215	D19	111.9347
A45	118.70607	D20	-65.44687
A46	121.07301	D21	53.78084
A47	118.75976	D22	174.31243
A48	121.06685	D23	-9.43322
A49	119.9439	D24	61.90386
A50	119.39793	D25	-178.39372
A51	119.4004	D26	-59.82297
A52	121.30933	D27	-173.87944
A53	121.12482	D28	-159.64948
A54	110.67009	D29	23.66886
A55	110.90394	D30	-178.67702
A56	111.30913	D31	1.63747
A57	87.72493	D32	177.48666
A58	91.35882	D33	-2.69918
A59	98.28882	D34	-179.52083
A60	135.05442	D35	179.66742
A61	115.51623	D36	21.95039
A62	111.48909	D37	-66.17954
D1	130.8705	D38	111.63746
D2	-173.96287	D39	-13.04698
D3	14.61108	D40	-121.01835
D4	124.39857	D41	52.23012
D5	88.24507	D42	63.44423
D6	-91.67813	D43	32.35534
D7	179.54691	D44	-179.42658
D8	-0.51384	D45	0.07318
D9	-178.92727	D46	-0.28681
D10	-0.12611	D47	0.02428
D11	-0.84647	D48	1.33068

D49	-179.67933	D56	-154.37254
D50	-179.54583	D57	75.02027
D51	-178.97672	D58	-36.30461
D52	-179.51924	D59	-164.83684
D53	113.42784	D60	-168.61289
D54	-127.3386	D61	52.98417
D55	-6.74439		

1.9.3. Step 3 – Ring closure, forming a tetrahedral intermediate

- Molecular complex 5

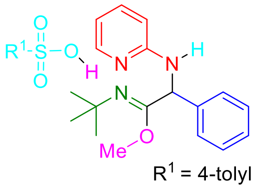

Symbolic Z-matrix:

Charge = 0 Multiplicity = 1

C							
H	1 B1						
C	1 B2	2	A1				
N	3 B3	1	A2	2	D1	0	
C	4 B4	3	A3	1	D2	0	
N	1 B5	3	A4	4	D3	0	
C	1 B6	6	A5	3	D4	0	
C	7 B7	1	A6	6	D5	0	
C	7 B8	1	A7	6	D6	0	
C	8 B9	7	A8	1	D7	0	
H	8 B10	7	A9	1	D8	0	
C	9 B11	7	A10	1	D9	0	
H	9 B12	7	A11	1	D10	0	
C	12 B13	9	A12	7	D11	0	
H	10 B14	8	A13	7	D12	0	
H	12 B15	9	A14	7	D13	0	
H	14 B16	12	A15	9	D14	0	
C	5 B17	4	A16	3	D15	0	
H	18 B18	5	A17	4	D16	0	
H	18 B19	5	A18	4	D17	0	
H	18 B20	5	A19	4	D18	0	
C	5 B21	4	A20	3	D19	0	
H	22 B22	5	A21	4	D20	0	
H	22 B23	5	A22	4	D21	0	
H	22 B24	5	A23	4	D22	0	
C	5 B25	4	A24	3	D23	0	
H	26 B26	5	A25	4	D24	0	
H	26 B27	5	A26	4	D25	0	
H	26 B28	5	A27	4	D26	0	
C	6 B29	1	A28	7	D27	0	
C	30 B30	6	A29	1	D28	0	
C	30 B31	6	A30	1	D29	0	
C	31 B32	30	A31	6	D30	0	
H	31 B33	30	A32	6	D31	0	
C	32 B34	30	A33	6	D32	0	
H	32 B35	30	A34	6	D33	0	
H	33 B36	31	A35	30	D34	0	
H	35 B37	32	A36	30	D35	0	

N	30	B38	6	A37	1	D36	0
S	4	B39	3	A38	1	D37	0
O	40	B40	4	A39	3	D38	0
O	40	B41	4	A40	3	D39	0
O	40	B42	4	A41	3	D40	0
H	6	B43	1	A42	7	D41	0
C	40	B44	4	A43	3	D42	0
C	45	B45	40	A44	4	D43	0
C	46	B46	45	A45	40	D44	0
C	47	B47	46	A46	45	D45	0
C	48	B48	47	A47	46	D46	0
C	49	B49	48	A48	47	D47	0
H	46	B50	45	A49	40	D48	0
H	47	B51	46	A50	45	D49	0
H	49	B52	48	A51	47	D50	0
H	50	B53	49	A52	48	D51	0
C	48	B54	47	A53	46	D52	0
H	55	B55	48	A54	47	D53	0
H	55	B56	48	A55	47	D54	0
H	55	B57	48	A56	47	D55	0
C	3	B58	1	A57	6	D56	0
H	59	B59	3	A58	1	D57	0
H	59	B60	3	A59	1	D58	0
H	59	B61	3	A60	1	D59	0
O	3	B62	1	A61	6	D60	0
H	4	B63	3	A62	1	D61	0

Variables:

B1	1.09522
B2	1.52845
B3	1.29046
B4	1.49705
B5	1.4303
B6	1.52219
B7	1.39764
B8	1.39592
B9	1.39177
B10	1.0878
B11	1.39462
B12	1.08842
B13	1.39272
B14	1.08537
B15	1.08498
B16	1.08548
B17	1.53228
B18	1.09368
B19	1.09051
B20	1.09428
B21	1.53162
B22	1.09423
B23	1.09209
B24	1.09411
B25	1.52985
B26	1.09212
B27	1.09341
B28	1.09374
B29	1.37666
B30	1.41117
B31	2.2923
B32	1.37766
B33	1.08473
B34	1.38343
B35	1.08756
B36	1.08578
B37	1.08354
B38	1.33694
B39	3.43093
B40	1.49693
B41	1.47897
B42	1.48284
B43	1.0239
B44	1.78244
B45	1.38973
B46	1.39355
B47	1.3974

B48	1.40016
B49	1.39064
B50	1.08532
B51	1.08693
B52	1.08723
B53	1.08574
B54	1.50779
B55	1.09335
B56	1.09264
B57	1.09528
B58	2.39863
B59	1.09227
B60	1.09125
B61	1.08806
B62	1.31686
B63	1.04882
A1	105.48757
A2	122.35489
A3	126.55029
A4	112.99788
A5	111.49148
A6	118.85447
A7	121.20346
A8	120.27837
A9	119.57557
A10	119.62842
A11	121.12117
A12	120.42086
A13	119.91467
A14	119.20681
A15	120.07854
A16	109.96976
A17	108.45553
A18	112.36588
A19	110.22927
A20	108.66065
A21	109.47227
A22	111.93049
A23	108.84271
A24	106.4581
A25	110.76405
A26	108.2877
A27	111.24502
A28	119.35623
A29	119.89716
A30	148.2538
A31	117.95028
A32	119.38896
A33	92.79003
A34	146.6221
A35	119.93369
A36	120.63428
A37	117.00145
A38	114.91757
A39	51.45481
A40	71.31035
A41	101.61475
A42	118.2021
A43	148.77341
A44	119.51118
A45	119.14625
A46	121.06173
A47	118.58409
A48	121.01751
A49	119.39437
A50	119.54408
A51	119.36492
A52	121.2976
A53	120.93729
A54	111.14864
A55	111.22021
A56	110.73038
A57	89.57589
A58	95.78778

A59	95.45021
A60	132.67629
A61	120.04918
A62	118.08666
D1	138.55918
D2	174.86133
D3	20.65312
D4	123.74505
D5	99.08353
D6	-78.97647
D7	-178.36475
D8	0.93962
D9	178.13535
D10	0.69973
D11	0.2634
D12	179.78663
D13	-178.96476
D14	-179.94853
D15	59.61337
D16	171.42891
D17	-68.81488
D18	53.01268
D19	-63.97999
D20	-56.99017
D21	64.69644
D22	-175.55284
D23	178.3321
D24	62.13689
D25	-177.99102
D26	-58.32016
D27	-171.03758
D28	-174.47113
D29	7.34858
D30	-178.69471
D31	2.66726
D32	177.8268
D33	-2.22155
D34	-179.70779
D35	-179.96666
D36	5.73243
D37	28.38822
D38	-121.10214
D39	97.90274
D40	-13.67853
D41	30.04295
D42	-170.29085
D43	-108.56483
D44	-177.42311
D45	-0.05935
D46	0.01694
D47	-0.00997
D48	3.19096
D49	-179.96651
D50	-179.84345
D51	-179.08344
D52	-178.65049
D53	-142.24927
D54	-21.5506
D55	98.2404
D56	-157.79156
D57	65.15588
D58	-46.90603
D59	-170.87081
D60	-162.42207
D61	-2.54112

- Transition state 3

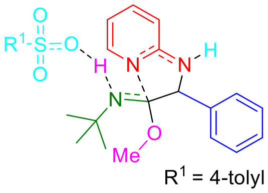

Symbolic Z-matrix:

Charge = 0 Multiplicity = 1

C								
H	1	B1						
C	1	B2	2	A1				
N	3	B3	1	A2	2	D1	0	
C	4	B4	3	A3	1	D2	0	
N	1	B5	3	A4	4	D3	0	
C	1	B6	6	A5	3	D4	0	
C	7	B7	1	A6	6	D5	0	
C	7	B8	1	A7	6	D6	0	
C	8	B9	7	A8	1	D7	0	
H	8	B10	7	A9	1	D8	0	
C	9	B11	7	A10	1	D9	0	
H	9	B12	7	A11	1	D10	0	
C	10	B13	8	A12	7	D11	0	
H	10	B14	8	A13	7	D12	0	
H	12	B15	9	A14	7	D13	0	
H	14	B16	10	A15	8	D14	0	
C	5	B17	4	A16	3	D15	0	
H	18	B18	5	A17	4	D16	0	
H	18	B19	5	A18	4	D17	0	
H	18	B20	5	A19	4	D18	0	
C	5	B21	4	A20	3	D19	0	
H	22	B22	5	A21	4	D20	0	
H	22	B23	5	A22	4	D21	0	
H	22	B24	5	A23	4	D22	0	
C	5	B25	4	A24	3	D23	0	
H	26	B26	5	A25	4	D24	0	
H	26	B27	5	A26	4	D25	0	
H	26	B28	5	A27	4	D26	0	
C	6	B29	1	A28	7	D27	0	
C	30	B30	6	A29	1	D28	0	
C	30	B31	6	A30	1	D29	0	
C	31	B32	30	A31	6	D30	0	
H	31	B33	30	A32	6	D31	0	
C	32	B34	30	A33	6	D32	0	
H	32	B35	30	A34	6	D33	0	
H	33	B36	31	A35	30	D34	0	
H	35	B37	32	A36	30	D35	0	
N	30	B38	6	A37	1	D36	0	
S	6	B39	1	A38	7	D37	0	
O	40	B40	6	A39	1	D38	0	
O	40	B41	6	A40	1	D39	0	
O	40	B42	6	A41	1	D40	0	
H	6	B43	1	A42	7	D41	0	
C	40	B44	6	A43	1	D42	0	
C	45	B45	40	A44	6	D43	0	
C	46	B46	45	A45	40	D44	0	
C	47	B47	46	A46	45	D45	0	
C	48	B48	47	A47	46	D46	0	
C	45	B49	40	A48	6	D47	0	
H	46	B50	45	A49	40	D48	0	
H	47	B51	46	A50	45	D49	0	
H	49	B52	48	A51	47	D50	0	
H	50	B53	45	A52	40	D51	0	
C	48	B54	47	A53	46	D52	0	
H	55	B55	48	A54	47	D53	0	
H	55	B56	48	A55	47	D54	0	
H	55	B57	48	A56	47	D55	0	
C	3	B58	1	A57	6	D56	0	
H	59	B59	3	A58	1	D57	0	

H	59	B60	3	A59	1	D58	0
H	59	B61	3	A60	1	D59	0
O	3	B62	1	A61	6	D60	0
H	4	B63	3	A62	1	D61	0

Variables:

B1	1.09693
B2	1.56344
B3	1.34843
B4	1.4788
B5	1.45136
B6	1.5083
B7	1.39644
B8	1.39687
B9	1.39329
B10	1.08782
B11	1.39235
B12	1.08536
B13	1.39357
B14	1.0854
B15	1.08518
B16	1.08546
B17	1.53232
B18	1.09464
B19	1.09401
B20	1.09494
B21	1.53311
B22	1.09421
B23	1.09138
B24	1.09489
B25	1.53226
B26	1.09484
B27	1.0934
B28	1.09378
B29	1.35007
B30	1.4129
B31	2.34986
B32	1.3733
B33	1.08388
B34	1.37255
B35	1.08589
B36	1.0857
B37	1.0828
B38	1.34184
B39	3.45674
B40	1.49361
B41	1.46671
B42	1.49348
B43	1.02833
B44	1.78683
B45	1.39368
B46	1.38972
B47	1.40129
B48	1.39625
B49	1.38928
B50	1.08572
B51	1.0874
B52	1.08724
B53	1.08586
B54	1.50794
B55	1.09258
B56	1.09421
B57	1.09469
B58	2.39795
B59	1.09423
B60	1.09346
B61	1.08973
B62	1.35426
B63	1.0267
A1	106.18723
A2	114.56138
A3	128.36449
A4	105.99218
A5	115.14381
A6	118.49682

A7	122.08107
A8	120.56161
A9	119.78524
A10	120.0853
A11	121.42048
A12	119.85243
A13	119.85768
A14	118.90815
A15	120.0695
A16	112.25526
A17	109.57447
A18	111.09609
A19	110.38734
A20	110.04103
A21	109.75973
A22	111.67665
A23	109.49287
A24	104.77069
A25	110.37258
A26	109.68797
A27	110.97904
A28	111.82177
A29	126.775
A30	141.58401
A31	117.35239
A32	119.00796
A33	91.58612
A34	145.09132
A35	119.34343
A36	120.29657
A37	112.36068
A38	119.31485
A39	63.10223
A40	121.1937
A41	50.08127
A42	123.58502
A43	130.85621
A44	118.91747
A45	119.25957
A46	121.08998
A47	118.52626
A48	120.22951
A49	119.45081
A50	119.59615
A51	119.35943
A52	119.15485
A53	120.24126
A54	111.25674
A55	111.01736
A56	110.87222
A57	94.45861
A58	89.01516
A59	102.98596
A60	133.39718
A61	118.92487
A62	113.6195
D1	-165.75937
D2	160.47287
D3	79.51284
D4	125.1491
D5	146.84136
D6	-35.85183
D7	176.98496
D8	-3.31163
D9	-177.32035
D10	2.76357
D11	0.44519
D12	179.95006
D13	-179.68896
D14	179.62195
D15	53.72503
D16	175.30066
D17	-65.54611
D18	56.14782

D19	-70.18736	D41	4.52648
D20	-52.13916	D42	148.64708
D21	68.39342	D43	129.54311
D22	-171.30444	D44	-177.58713
D23	172.43194	D45	-0.15507
D24	59.03443	D46	-0.11085
D25	178.68434	D47	-48.40684
D26	-60.40169	D48	3.09724
D27	151.62503	D49	-179.79174
D28	-179.78197	D50	-179.24492
D29	-0.63764	D51	-3.52877
D30	-179.8199	D52	-179.33498
D31	3.04097	D53	-175.35498
D32	179.34393	D54	-54.88497
D33	-1.02617	D55	64.49106
D34	-179.26726	D56	-122.57105
D35	-179.12623	D57	76.3746
D36	-0.55777	D58	-33.84105
D37	53.56839	D59	-166.71404
D38	61.78032	D60	-142.81463
D39	-41.40947	D61	-42.69687
D40	-136.91317		

- Molecular complex 6

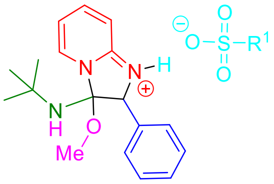

R1 = 4-tolyl

Symbolic Z-matrix:

Charge = 0 Multiplicity = 1

C								
H	1	B1						
C	1	B2	2	A1				
N	3	B3	1	A2	2	D1	0	
C	4	B4	3	A3	1	D2	0	
N	1	B5	3	A4	4	D3	0	
C	1	B6	6	A5	3	D4	0	
C	7	B7	1	A6	6	D5	0	
C	7	B8	1	A7	6	D6	0	
C	8	B9	7	A8	1	D7	0	
H	8	B10	7	A9	1	D8	0	
C	9	B11	7	A10	1	D9	0	

H	9	B12	7	A11	1	D10	0
C	10	B13	8	A12	7	D11	0
H	10	B14	8	A13	7	D12	0
H	12	B15	9	A14	7	D13	0
H	14	B16	10	A15	8	D14	0
C	5	B17	4	A16	3	D15	0
H	18	B18	5	A17	4	D16	0
H	18	B19	5	A18	4	D17	0
H	18	B20	5	A19	4	D18	0
C	5	B21	4	A20	3	D19	0
H	22	B22	5	A21	4	D20	0
H	22	B23	5	A22	4	D21	0
H	22	B24	5	A23	4	D22	0
C	5	B25	4	A24	3	D23	0
H	26	B26	5	A25	4	D24	0
H	26	B27	5	A26	4	D25	0
H	26	B28	5	A27	4	D26	0
C	6	B29	1	A28	7	D27	0
C	30	B30	6	A29	1	D28	0
C	30	B31	6	A30	1	D29	0
C	31	B32	30	A31	6	D30	0
H	31	B33	30	A32	6	D31	0
C	32	B34	30	A33	6	D32	0
H	32	B35	30	A34	6	D33	0
H	33	B36	31	A35	30	D34	0
H	35	B37	32	A36	30	D35	0
N	30	B38	6	A37	1	D36	0
S	31	B39	30	A38	6	D37	0
O	40	B40	31	A39	30	D38	0
O	40	B41	31	A40	30	D39	0
O	40	B42	31	A41	30	D40	0
H	6	B43	1	A42	7	D41	0
C	40	B44	31	A43	30	D42	0
C	45	B45	40	A44	31	D43	0
C	46	B46	45	A45	40	D44	0
C	47	B47	46	A46	45	D45	0
C	48	B48	47	A47	46	D46	0

C	45	B49	40	A48	31	D47	0
H	46	B50	45	A49	40	D48	0
H	47	B51	46	A50	45	D49	0
H	49	B52	48	A51	47	D50	0
H	50	B53	45	A52	40	D51	0
C	48	B54	47	A53	46	D52	0
H	55	B55	48	A54	47	D53	0
H	55	B56	48	A55	47	D54	0
H	55	B57	48	A56	47	D55	0
C	3	B58	1	A57	6	D56	0
H	59	B59	3	A58	1	D57	0
H	59	B60	3	A59	1	D58	0
H	59	B61	3	A60	1	D59	0
O	3	B62	1	A61	6	D60	0
H	4	B63	3	A62	1	D61	0

Variables:

B1	1.09467
B2	1.5967
B3	1.40446
B4	1.47848
B5	1.46355
B6	1.50315
B7	1.39645
B8	1.39795
B9	1.39295
B10	1.08788
B11	1.39299
B12	1.08566
B13	1.39408
B14	1.08535
B15	1.08529
B16	1.0855
B17	1.53583
B18	1.09517
B19	1.09207
B20	1.09476
B21	1.53451

B22	1.09329
B23	1.0936
B24	1.09523
B25	1.53226
B26	1.09538
B27	1.09358
B28	1.09462
B29	1.34564
B30	1.41077
B31	2.37425
B32	1.37144
B33	1.08227
B34	1.36631
B35	1.08481
B36	1.08647
B37	1.08222
B38	1.34374
B39	3.37181
B40	1.49425
B41	1.46271
B42	1.49125
B43	1.02639
B44	1.79543
B45	1.39119
B46	1.39412
B47	1.39905
B48	1.39997
B49	1.39201
B50	1.08604
B51	1.08736
B52	1.08756
B53	1.08582
B54	1.50806
B55	1.09322
B56	1.09309
B57	1.09525
B58	2.41543
B59	1.09589
B60	1.09437
B61	1.09033
B62	1.38555
B63	1.01937
A1	106.8243
A2	119.67277
A3	126.89305
A4	104.68042
A5	114.43998
A6	118.79581
A7	121.69137
A8	120.53858
A9	119.64393
A10	119.95889
A11	120.24702
A12	119.79884
A13	119.93499
A14	119.21786
A15	120.04689
A16	109.78353
A17	110.1498
A18	111.10132
A19	109.76953
A20	114.47671
A21	111.15275
A22	111.45971
A23	109.11276
A24	104.57394
A25	110.5909
A26	110.01311
A27	110.6869
A28	110.52743
A29	128.45967
A30	140.25373
A31	117.64174
A32	119.00584

A33	91.18213	D8	-2.11565
A34	143.1491	D9	-177.47926
A35	119.15533	D10	2.09021
A36	120.13924	D11	0.65851
A37	111.90226	D12	-179.56759
A38	78.86928	D13	-179.78785
A39	69.51828	D14	179.82705
A40	176.66014	D15	80.16443
A41	63.44881	D16	172.37684
A42	121.68075	D17	-67.35765
A43	70.66396	D18	53.06497
A44	119.90448	D19	-43.96429
A45	119.44941	D20	-64.13694
A46	121.04851	D21	57.49507
A47	118.4138	D22	176.6587
A48	119.50953	D23	-163.01029
A49	119.24023	D24	62.78632
A50	119.58629	D25	-177.40882
A51	119.33265	D26	-57.3136
A52	119.25488	D27	140.17669
A53	120.80162	D28	178.7305
A54	111.32703	D29	-5.38261
A55	111.31939	D30	176.2792
A56	110.56121	D31	1.14508
A57	91.9837	D32	-176.24055
A58	87.21542	D33	-0.44281
A59	106.03733	D34	-176.51325
A60	132.85173	D35	178.50698
A61	113.84786	D36	-1.68859
A62	111.38794	D37	71.18026
D1	-149.64723	D38	45.13136
D2	99.76739	D39	-134.68646
D3	96.74093	D40	-83.28898
D4	127.50706	D41	1.65485
D5	133.39294	D42	158.56978
D6	-48.23939	D43	82.82181
D7	177.11587	D44	-178.23759

D45	-0.37899	D54	-29.64739
D46	0.30399	D55	89.85842
D47	-95.73152	D56	-110.4062
D48	0.50646	D57	78.24578
D49	179.68117	D58	-31.35857
D50	179.70802	D59	-167.97538
D51	0.07205	D60	-133.09114
D52	-178.15501	D61	-50.72949
D53	-150.66518		

1.9.4. Step 4 – Methanol elimination

- Molecular complex 7

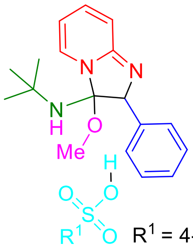

R^1 = 4-tolyl

Symbolic Z-matrix:

Charge = 0 Multiplicity = 1

C	1.5732	-0.06465	-0.96545	H	3.45168	4.91598	-1.89128
H	0.48334	-0.06146	-1.08796	C	0.28511	1.54405	1.92089
C	1.91464	-0.56441	0.50918	H	-0.0527	2.50159	2.32982
N	2.47917	0.31889	1.48493	H	0.12313	1.58003	0.84053
C	1.75524	1.33445	2.29562	H	-0.34821	0.77135	2.36004
N	2.16673	-1.03948	-1.88636	C	2.51205	2.65765	2.11906
C	2.09239	1.3284	-1.2574	H	3.57732	2.52445	2.34274
C	1.21132	2.39839	-1.41756	H	2.41306	3.03203	1.09404
C	3.4687	1.55883	-1.36119	H	2.11915	3.41254	2.80649
C	1.69998	3.68594	-1.64714	C	1.83095	0.88745	3.76114
H	0.13994	2.22022	-1.36507	H	2.87426	0.81254	4.08343
C	3.95823	2.84367	-1.57801	H	1.3111	1.5978	4.41383
H	4.15902	0.72026	-1.28893	H	1.36458	-0.09639	3.87036
C	3.07227	3.91397	-1.7165	C	2.93188	-1.80462	-1.18885
H	1.00379	4.50928	-1.77464	C	3.77349	-2.8954	-1.63206
H	5.02874	3.00994	-1.65062	C	3.65837	-2.2725	1.10247

C	4.49283	-3.60015	-0.72118	O	-1.91086	-0.56117	0.97181
H	3.79234	-3.1153	-2.69255	H	-0.92247	-0.56647	1.04648
C	4.44663	-3.29724	0.6872	C	-4.09807	-0.15726	-0.30697
H	3.53556	-1.94916	2.13111	C	-4.75347	0.9568	0.20925
H	5.12106	-4.41923	-1.05875	C	-6.13283	0.90031	0.37155
H	5.02411	-3.86758	1.4023	C	-6.85457	-0.2475	0.02131
N	2.95044	-1.55058	0.18644	C	-6.16355	-1.34784	-0.49691
C	0.40412	-2.45192	0.48652	C	-4.78278	-1.31296	-0.66626
H	0.18512	-2.3169	-0.57792	H	-4.18775	1.84656	0.4663
H	-0.50165	-2.76952	1.00288	H	-6.66021	1.76107	0.77314
H	1.18305	-3.20667	0.62359	H	-6.71383	-2.24244	-0.77425
O	0.7886	-1.21102	1.10104	H	-4.239	-2.15673	-1.07845
H	3.29861	0.74293	1.0633	C	-8.35344	-0.28119	0.16934
S	-2.3437	-0.10553	-0.50315	H	-8.71761	-1.30506	0.27983
O	-1.92236	-1.12551	-1.4485	H	-8.67751	0.29643	1.03841
O	-1.91355	1.27023	-0.70517	H	-8.83403	0.15084	-0.71476

- Transition state 4

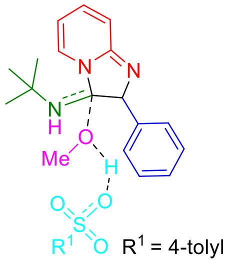

Symbolic Z-matrix:

Charge = 0 Multiplicity = 1

C	1.51561	0.02033	-0.84958	C	4.05683	2.78135	-1.50778
H	0.42781	0.09207	-0.91753	H	4.15923	0.69909	-0.97799
C	1.9553	-0.5154	0.54671	C	3.21997	3.8599	-1.80314
N	2.31978	0.22473	1.60925	H	1.18401	4.51847	-2.0462
C	1.72773	1.42962	2.25921	H	5.13479	2.90958	-1.51737
N	1.99398	-0.98354	-1.80533	H	3.64737	4.82926	-2.04024
C	2.12048	1.37164	-1.1802	C	0.28649	1.6701	1.83427
C	1.28458	2.44198	-1.4968	H	-0.04151	2.62189	2.26319
C	3.50713	1.54082	-1.20236	H	0.16053	1.75019	0.75365
C	1.83766	3.68641	-1.80414	H	-0.38379	0.88881	2.19576
H	0.20568	2.29702	-1.49056	C	2.60476	2.65018	1.95337

H	3.65819	2.43197	2.15897	O	0.52216	-1.48741	1.07454
H	2.51293	2.96032	0.91062	H	2.99578	-0.2278	2.21069
H	2.29546	3.48493	2.59029	S	-2.18314	-0.07262	-0.39285
C	1.76767	1.13567	3.76407	O	-1.78754	-1.04353	-1.42146
H	2.79819	0.99256	4.11325	O	-1.77721	1.32097	-0.63585
H	1.34509	1.97829	4.31727	O	-1.72408	-0.53857	0.98377
H	1.18304	0.2401	3.99528	H	-0.42883	-0.96327	1.05353
C	2.73174	-1.80876	-1.16785	C	-3.95927	-0.09218	-0.27598
C	3.46492	-2.95373	-1.66905	C	-4.62338	1.01993	0.23134
C	3.46722	-2.42913	1.09673	C	-6.00912	0.98222	0.35336
C	4.13984	-3.75012	-0.8081	C	-6.73628	-0.1503	-0.02925
H	3.42887	-3.13497	-2.73625	C	-6.04199	-1.25211	-0.53899
C	4.1367	-3.50071	0.61737	C	-4.65568	-1.23065	-0.66649
H	3.37218	-2.21312	2.15545	H	-4.05433	1.9006	0.51176
H	4.68339	-4.61041	-1.18533	H	-6.53674	1.84683	0.7478
H	4.63921	-4.17235	1.30058	H	-6.59498	-2.13677	-0.84407
N	2.83525	-1.57096	0.22497	H	-4.11043	-2.07528	-1.07545
C	0.23761	-2.71627	0.38283	C	-8.24047	-0.16804	0.07157
H	0.01927	-2.52562	-0.67139	H	-8.61456	-1.18302	0.22739
H	-0.64417	-3.15439	0.85617	H	-8.58899	0.457	0.89764
H	1.08509	-3.39533	0.50078	H	-8.69343	0.21537	-0.84902

- Molecular complex 8

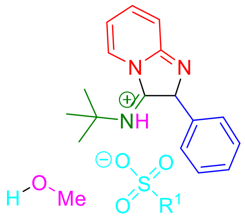

R^1 = 4-tolyl

Symbolic Z-matrix:

Charge = 0 Multiplicity = 1

C	-1.92576	-0.16209	-1.23644	C	-3.3961	-0.53274	-1.31787
H	-1.28024	-1.00104	-1.51092	C	-3.79527	-1.71067	-1.94556
C	-1.50561	0.31114	0.1517	C	-4.36306	0.3315	-0.79762
N	-1.47607	-0.30128	1.30058	C	-5.14834	-2.0408	-2.0247
C	-1.7965	-1.72978	1.63628	H	-3.04696	-2.37231	-2.37359
N	-1.64079	0.95928	-2.12476	C	-5.71277	0.00357	-0.87342

H	-4.05865	1.26443	-0.32695	N	-1.16093	1.61623	-0.00076
C	-6.1075	-1.18931	-1.48161	C	1.10633	1.85356	3.64483
H	-5.45028	-2.9628	-2.51112	H	1.57805	2.29714	2.76035
H	-6.4568	0.67628	-0.45895	H	1.87594	1.61858	4.38824
H	-7.15984	-1.44861	-1.53773	H	0.40732	2.56654	4.09314
C	-1.29705	-2.68438	0.55167	O	0.37284	0.69002	3.2918
H	-1.29807	-3.69566	0.96827	H	-1.02657	0.20852	2.07512
H	-1.9538	-2.69679	-0.32029	S	1.83116	-0.1186	0.27509
H	-0.27805	-2.42441	0.25077	O	1.75644	1.33745	0.52011
C	-3.3044	-1.86473	1.87017	O	0.89155	-0.57323	-0.77639
H	-3.63734	-1.17284	2.65011	O	1.70309	-0.90458	1.54186
H	-3.87936	-1.67596	0.96114	H	0.9678	0.10702	2.76396
H	-3.51691	-2.88511	2.20341	C	3.46932	-0.46977	-0.33995
C	-1.04167	-2.01827	2.93773	C	4.40066	-1.11172	0.46464
H	-1.29594	-1.29385	3.71773	C	5.68373	-1.34524	-0.03274
H	-1.32076	-3.01549	3.287	C	6.04121	-0.94398	-1.32056
H	0.03695	-1.98925	2.76525	C	5.08025	-0.30044	-2.1129
C	-1.21675	1.91832	-1.39404	C	3.79941	-0.06169	-1.63201
C	-0.82214	3.25401	-1.78007	H	4.11212	-1.42908	1.46123
C	-0.77507	2.54285	0.96154	H	6.4159	-1.85084	0.59146
C	-0.46603	4.14422	-0.82867	H	5.34304	0.01227	-3.12067
H	-0.83554	3.48664	-2.83747	H	3.05076	0.42669	-2.2485
C	-0.45657	3.79108	0.57566	C	7.42648	-1.19348	-1.8612
H	-0.73164	2.18929	1.98289	H	7.92384	-0.25111	-2.11153
H	-0.17255	5.14906	-1.11433	H	8.04678	-1.72001	-1.13222
H	-0.15345	4.51348	1.32246	H	7.38817	-1.79714	-2.7733

1.9.5. Step 5 – Proton abstraction by tosylate

- Molecular complex 9

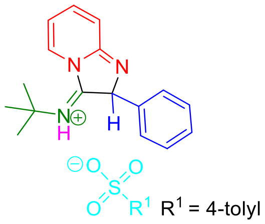

Symbolic Z-matrix:

Charge = 0 Multiplicity = 1

C	1.17114	0.92575	-0.87416	C	2.50396	-0.23101	-2.17919
H	0.08047	0.82245	-0.91574	C	3.30043	-0.76714	-3.26283
C	1.70934	-0.26081	-0.07593	C	3.35769	-2.05552	-0.74711
N	1.34989	-0.45904	1.15321	C	4.0603	-1.86277	-3.06505
C	1.68208	-1.4664	2.20094	H	3.24142	-0.24492	-4.20987
N	1.74126	0.78887	-2.20012	C	4.08828	-2.52281	-1.77554
C	1.55102	2.23949	-0.20282	H	3.34261	-2.52733	0.21966
C	0.64137	2.87848	0.64157	H	4.65725	-2.26733	-3.87539
C	2.82373	2.78124	-0.39611	H	4.69111	-3.40943	-1.62613
C	1.01204	4.05617	1.29118	N	2.56953	-0.91468	-0.90883
H	-0.3452	2.44782	0.79038	S	-1.76602	-0.87923	0.53744
C	3.18872	3.95638	0.25586	O	-0.97568	-1.30348	-0.64016
H	3.5193	2.29522	-1.07499	O	-1.20945	0.39909	1.1123
C	2.28271	4.59543	1.1023	O	-1.948	-1.90804	1.57503
H	0.29999	4.5537	1.94207	H	0.48797	0.09559	1.36767
H	4.17697	4.37679	0.09757	C	-3.39132	-0.45295	-0.06172
H	2.56533	5.51415	1.60676	C	-4.48428	-0.56087	0.79221
C	3.164	-1.33967	2.57611	C	-5.74349	-0.18512	0.33308
H	3.40274	-2.08335	3.34158	C	-5.92313	0.29775	-0.96765
H	3.85419	-1.48096	1.74281	C	-4.80891	0.38649	-1.80811
H	3.35377	-0.34577	2.99128	C	-3.54307	0.01457	-1.36285
C	1.23772	-2.87056	1.766	H	-4.34037	-0.9529	1.79403
H	1.65031	-3.19342	0.80982	H	-6.60347	-0.27516	0.99196
H	1.54379	-3.58813	2.53377	H	-4.93581	0.74344	-2.82699
H	0.14836	-2.89346	1.6734	H	-2.679	0.06063	-2.01858
C	0.83852	-1.05806	3.41637	C	-7.28626	0.72988	-1.44581
H	-0.22835	-1.12429	3.18328	H	-7.46822	1.78088	-1.19656
H	1.0594	-1.7364	4.24472	H	-7.37689	0.62555	-2.52993
H	1.07947	-0.03716	3.72983	H	-8.07603	0.13717	-0.97704

- Transition state 5

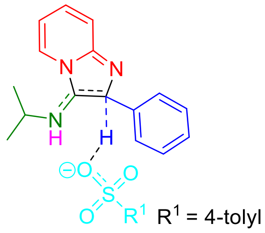

Symbolic Z-matrix:

Charge = 0 Multiplicity = 1

C	1.71383	0.39783	-1.09387	C	2.07263	-1.60683	-1.83148
H	0.42772	0.51076	-1.1362	C	2.25842	-2.81212	-2.59481
C	1.77757	-0.47509	0.07733	C	1.71419	-2.98925	0.17042
N	1.7001	-0.0307	1.32256	C	2.17793	-4.01503	-1.98225
C	1.74744	-0.62265	2.68439	H	2.44419	-2.6969	-3.65585
N	2.04751	-0.38617	-2.2332	C	1.87119	-4.10251	-0.57291
C	2.2122	1.80502	-1.00397	H	1.42517	-3.00379	1.20721
C	1.50404	2.76161	-0.26511	H	2.31181	-4.92902	-2.55056
C	3.39716	2.17328	-1.64957	H	1.72684	-5.06692	-0.10233
C	2.00034	4.0598	-0.14544	N	1.884	-1.73942	-0.41689
H	0.54461	2.50267	0.18229	S	-1.46632	0.13952	0.23704
C	3.87871	3.47474	-1.54016	O	-0.91388	0.72125	-1.05777
H	3.92355	1.43288	-2.24388	O	-1.15743	1.00917	1.38757
C	3.18704	4.41895	-0.78043	O	-1.05786	-1.26882	0.37783
H	1.4437	4.79496	0.42738	H	1.55725	0.97462	1.33735
H	4.79711	3.75321	-2.04791	C	-3.22861	0.17876	0.00176
H	3.56596	5.43255	-0.69475	C	-3.81975	-0.77877	-0.82041
C	3.02461	-1.45508	2.85186	C	-5.19238	-0.734	-1.027
H	3.08257	-1.81756	3.88236	C	-5.98224	0.25382	-0.42283
H	3.07216	-2.32302	2.19247	C	-5.36204	1.19858	0.39697
H	3.90301	-0.83393	2.6536	C	-3.98465	1.16922	0.61397
C	0.46393	-1.38293	3.04033	H	-3.20502	-1.54694	-1.27901
H	0.22745	-2.20202	2.36277	H	-5.66385	-1.4767	-1.66578
H	0.56485	-1.77778	4.05652	H	-5.96257	1.9684	0.87389
H	-0.38686	-0.69955	2.99612	H	-3.49463	1.89465	1.25496
C	1.83731	0.5977	3.61106	C	-7.47197	0.2779	-0.6524
H	0.95309	1.23113	3.48303	H	-7.92936	1.157	-0.19298
H	1.87406	0.26663	4.65158	H	-7.7027	0.29311	-1.72175
H	2.7377	1.18466	3.40214	H	-7.94594	-0.61199	-0.22603

- Molecular complex 10

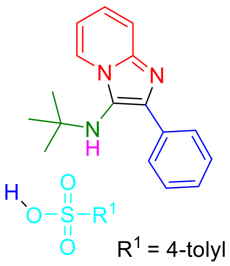

Symbolic Z-matrix:

Charge = 0 Multiplicity = 1

C	-1.57177	0.28102	0.66401	H	-4.34337	0.88653	0.5781
H	0.68056	0.18459	1.6396	C	-0.92774	-1.66862	1.34681
C	-2.10541	-0.61927	-0.23917	C	-0.34656	-2.78778	1.98586
N	-2.91563	-0.53229	-1.37594	C	-1.84573	-3.09869	-0.36339
C	-4.38593	-0.38584	-1.19276	C	-0.53189	-4.0282	1.43815
N	-0.85292	-0.36965	1.63891	H	0.23688	-2.62321	2.88401
C	-1.69469	1.74791	0.6709	C	-1.28815	-4.18519	0.24066
C	-1.56505	2.49236	-0.50591	H	-2.41262	-3.10967	-1.28552
C	-1.95011	2.40934	1.87705	H	-0.09333	-4.90115	1.90923
C	-1.72609	3.87485	-0.48173	H	-1.41686	-5.16252	-0.20759
H	-1.2826	1.99264	-1.42742	N	-1.67356	-1.86626	0.2034
C	-2.10894	3.79235	1.8996	S	1.7811	0.14899	-0.21021
H	-2.02946	1.8264	2.78994	O	1.63176	0.43403	1.34396
C	-2.00661	4.52705	0.71859	O	1.271	1.27885	-0.97294
H	-1.61296	4.4457	-1.39808	O	1.26215	-1.18269	-0.50477
H	-2.31371	4.2968	2.83876	H	-2.56442	0.16061	-2.0287
H	-2.13072	5.6054	0.7358	C	3.54826	0.12847	-0.33876
C	-4.90858	-1.60385	-0.43004	C	4.22484	-1.07721	-0.18895
H	-5.98797	-1.51234	-0.27738	C	5.61389	-1.0798	-0.27231
H	-4.71744	-2.52541	-0.98802	C	6.32461	0.10318	-0.50193
H	-4.43669	-1.68529	0.55524	C	5.61302	1.29936	-0.64825
C	-4.9839	-0.35379	-2.59878	C	4.22424	1.3218	-0.57107
H	-4.71717	-1.26056	-3.14948	H	3.66361	-1.99079	-0.02066
H	-6.07395	-0.27854	-2.54818	H	6.15573	-2.01484	-0.15908
H	-4.61441	0.51311	-3.15877	H	6.15447	2.22432	-0.82725
C	-4.7561	0.89507	-0.43534	H	3.66326	2.2427	-0.69371
H	-4.36732	1.78097	-0.94824	C	7.82672	0.08617	-0.62315
H	-5.84462	0.98793	-0.35959	H	8.26346	1.01697	-0.25298

H	8.25972	-0.74479	-0.06124	H	8.12589	-0.0277	-1.67052

1.10. Proposal J: GBB reaction, methanol acts as a proton shuttle in the third step, catalyzed by *p*-toluenesulfonic acid

1.10.1. Step 1 - Isocyanide nucleophilic attack, forming nitrilium

- Molecular complex 1

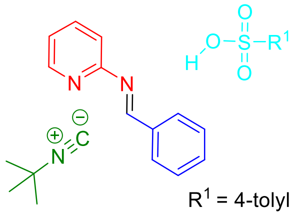

Symbolic Z-matrix:

Charge = 0 Multiplicity = 1

C	0.	0.	0.	H	3.18687	-3.37723	0.1108
H	0.	0.	1.08845	H	2.90585	-2.78308	-1.55419
C	2.8086	0.	0.61272	H	4.34562	-3.74278	-1.18994
N	3.54481	-0.76189	0.11949	C	5.55526	-1.99647	0.5617
C	4.45867	-1.71562	-0.47011	H	5.12671	-2.41768	1.47536
N	0.04627	-1.1535	-0.57828	H	6.26645	-2.71395	0.14296
C	-0.06862	1.26436	-0.71022	H	6.09095	-1.07755	0.81457
C	-0.57567	2.37109	-0.01368	C	0.19465	-2.37267	0.14801
C	0.38906	1.40531	-2.02979	C	0.20746	-3.56837	-0.57053
C	-0.67811	3.60339	-0.64699	C	0.49865	-3.40757	2.14623
H	-0.89251	2.25707	1.01932	C	0.38956	-4.73793	0.15952
C	0.30027	2.64714	-2.64689	H	0.1132	-3.57478	-1.65177
H	0.85612	0.57072	-2.54681	C	0.53976	-4.66378	1.54166
C	-0.24166	3.73866	-1.96537	H	0.60856	-3.30275	3.22209
H	-1.08248	4.45715	-0.11392	H	0.4205	-5.69298	-0.35431
H	0.67116	2.76666	-3.65962	H	0.68822	-5.55311	2.14363
H	-0.30725	4.70392	-2.45799	N	0.32853	-2.27549	1.46409
C	5.03656	-1.09742	-1.74589	S	1.1243	-1.98128	-3.79279
H	5.73948	-1.80624	-2.1939	O	1.51992	-3.38224	-3.5916
H	4.23596	-0.89408	-2.46116	O	2.15216	-0.98246	-3.43346
H	5.57127	-0.17202	-1.51356	O	-0.17763	-1.64591	-3.09848
C	3.66461	-2.98383	-0.7931	H	-0.01596	-1.27239	-1.6643

C	0.75823	-1.78223	-5.52502	H	1.21804	-3.32735	-8.49945
C	1.16256	-2.75538	-6.42794	H	-0.66764	0.43197	-7.65416
C	0.90611	-2.56851	-7.78689	H	-0.22256	0.10893	-5.22872
C	0.2511	-1.42497	-8.24577	C	-0.03192	-1.22169	-9.71259
C	-0.1495	-0.46009	-7.31062	H	0.3535	-2.05187	-10.30869
C	0.10023	-0.63104	-5.95545	H	-1.10817	-1.14371	-9.89558
H	1.66226	-3.64586	-6.06071	H	0.43118	-0.29907	-10.07593

- Transition state 1

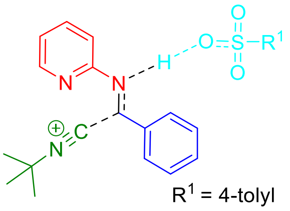

R^1 = 4-tolyl

Symbolic Z-matrix:

Charge = 0 Multiplicity = 1

C	0.	0.	0.	C	3.51426	-2.88418	-0.86473
H	0.	0.	1.08566	H	2.97717	-3.25209	0.01624
C	2.0038	0.	-0.04845	H	2.84401	-2.88164	-1.7283
N	2.94942	-0.62581	-0.28492	H	4.35163	-3.55861	-1.06855
C	4.07621	-1.48251	-0.60352	C	5.00764	-1.47041	0.61163
N	-0.27934	-1.17445	-0.56419	H	4.49202	-1.85252	1.49711
C	-0.39996	1.25058	-0.68466	H	5.86744	-2.11221	0.40218
C	-1.03918	2.23789	0.06794	H	5.36792	-0.45884	0.81735
C	-0.15994	1.44157	-2.04912	C	0.02862	-2.39096	0.07886
C	-1.46935	3.41072	-0.5485	C	-0.11364	-3.58799	-0.63415
H	-1.20781	2.0859	1.1307	C	0.782	-3.45531	1.94784
C	-0.58387	2.61983	-2.65481	C	0.2313	-4.75974	0.02134
H	0.38855	0.68814	-2.61104	H	-0.44786	-3.57621	-1.66613
C	-1.24285	3.59983	-1.91033	C	0.68504	-4.70221	1.34075
H	-1.97465	4.17434	0.0336	H	1.13153	-3.36017	2.97266
H	-0.38996	2.77706	-3.71108	H	0.15326	-5.70916	-0.49811
H	-1.5714	4.51604	-2.39102	H	0.96289	-5.59816	1.88406
C	4.75931	-0.91501	-1.84945	N	0.46783	-2.31105	1.33162
H	5.60072	-1.56409	-2.10838	S	0.88944	-1.94442	-3.69523
H	4.04797	-0.88718	-2.67748	O	1.3692	-3.3322	-3.61167
H	5.14034	0.09175	-1.65544	O	1.78819	-0.96236	-3.03408

O	-0.51713	-1.75688	-3.21298	H	2.36905	-2.92681	-5.94469
H	-0.44474	-1.24458	-1.59489	H	2.39622	-2.24608	-8.34554
C	0.84204	-1.51598	-5.42777	H	-0.69783	0.61901	-7.55949
C	1.71453	-2.14187	-6.31037	H	-0.71992	-0.06822	-5.16331
C	1.71733	-1.7591	-7.65021	C	0.83743	-0.37493	-9.57433
C	0.85598	-0.76255	-8.11728	H	1.77279	-0.64868	-10.06843
C	-0.01614	-0.15188	-7.20832	H	0.02151	-0.88257	-10.09982
C	-0.02942	-0.52306	-5.86758	H	0.68838	0.70148	-9.6951

- Molecular complex 2

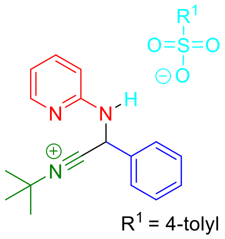

R1 = 4-tolyl

Symbolic Z-matrix:

Charge = 0 Multiplic ity = 1

C	0.	0.	0.	H	4.40615	1.16887	-1.99549
H	0.	0.	1.09508	C	3.65682	-2.18531	-1.38842
C	1.46297	0.	-0.3541	H	3.29174	-2.73971	-0.51768
N	2.55816	-0.21525	-0.61209	H	2.94612	-2.29683	-2.21221
C	3.865	-0.70668	-1.03852	H	4.62524	-2.59771	-1.68711
N	-0.54995	-1.23887	-0.47632	C	4.8155	-0.52533	0.1462
C	-0.71828	1.22359	-0.52898	H	4.46458	-1.08894	1.01483
C	-1.5515	1.93506	0.33491	H	5.79918	-0.90407	-0.14199
C	-0.58542	1.61462	-1.86344	H	4.91398	0.5297	0.41549
C	-2.25734	3.041	-0.13288	C	0.04086	-2.41903	-0.02613
H	-1.65547	1.61954	1.36968	C	-0.15862	-3.61525	-0.74185
C	-1.2914	2.72455	-2.32324	C	1.34076	-3.47092	1.54012
H	0.06059	1.04561	-2.53051	C	0.4385	-4.76073	-0.25376
C	-2.12557	3.43706	-1.46281	H	-0.72751	-3.60108	-1.66532
H	-2.90754	3.59098	0.53971	C	1.19975	-4.70208	0.92172
H	-1.18813	3.03029	-3.35965	H	1.9313	-3.36844	2.4477
H	-2.67373	4.29986	-1.82799	H	0.32387	-5.69709	-0.79049
C	4.29131	0.11301	-2.25665	H	1.67768	-5.58333	1.3336
H	5.25721	-0.26882	-2.59834	N	0.78827	-2.3393	1.07707
H	3.54923	0.00443	-3.05015	S	0.56006	-1.66555	-3.78404

O	1.2868	-2.94402	-3.81379	C	-0.6405	-0.24966	-5.81757
O	1.32902	-0.58437	-3.09653	H	2.06275	-2.27718	-6.15435
O	-0.82716	-1.7409	-3.25057	H	1.88126	-1.44516	-8.50149
H	-0.79869	-1.25327	-1.47556	H	-1.54153	0.89262	-7.39769
C	0.38178	-1.13389	-5.48143	H	-1.35379	0.05454	-5.05701
C	1.28973	-1.57015	-6.43871	C	0.02012	0.26231	-9.53417
C	1.17559	-1.10491	-7.74786	H	0.94987	0.1263	-10.09187
C	0.16279	-0.21317	-8.11028	H	-0.76539	-0.29756	-10.05285
C	-0.74234	0.20638	-7.12777	H	-0.25021	1.3212	-9.572

1.10.2. Step 2 – Ring Closure

- Molecular complex 3

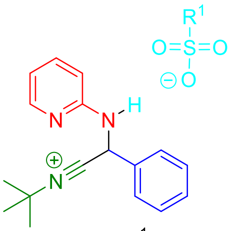

R1 = 4-tolyl

Symbolic Z-matrix:

Charge = 0 Multiplicity = 1

C	2.60538	0.38214	-0.56272	H	4.40257	-4.45155	-2.12253
H	3.44825	1.0734	-0.4544	C	-0.19764	0.5642	3.36951
C	2.04663	0.30115	0.83278	H	-0.52035	0.47436	4.41086
N	1.68246	0.15837	1.90905	H	-0.1783	1.61848	3.0838
C	1.19591	-0.05741	3.26581	H	-0.89381	0.04006	2.71499
N	1.67068	0.93291	-1.4981	C	2.19748	0.6308	4.19738
C	3.0999	-0.99743	-0.96229	H	2.23376	1.70596	4.0041
C	4.34604	-1.09998	-1.58265	H	1.87136	0.47158	5.22809
C	2.31799	-2.13633	-0.75562	H	3.19912	0.20861	4.08013
C	4.81451	-2.3421	-2.0023	C	1.15384	-1.57274	3.47807
H	4.94339	-0.20688	-1.74603	H	2.15393	-2.00696	3.38984
C	2.7953	-3.3768	-1.1758	H	0.77663	-1.76324	4.48659
H	1.33765	-2.06213	-0.28671	H	0.48345	-2.02615	2.74434
C	4.03849	-3.48219	-1.79676	C	1.21804	2.2256	-1.24827
H	5.78216	-2.41886	-2.48753	C	0.15019	2.73968	-2.00823
H	2.18566	-4.26083	-1.02013	C	1.4303	4.17741	-0.07388

C	-0.2589	4.03064	-1.75339
H	-0.33658	2.10923	-2.74457
C	0.39627	4.78162	-0.76557
H	1.9647	4.7143	0.70618
H	-1.08583	4.45883	-2.31164
H	0.10241	5.79973	-0.53866
N	1.84095	2.91828	-0.29792
S	-1.09135	-0.53884	-0.14512
O	-0.64258	-1.59328	0.80079
O	-0.60005	-0.7806	-1.52822
O	-0.7898	0.82936	0.33478
H	0.92672	0.27712	-1.76891
C	-2.87134	-0.66132	-0.22082
C	-3.46526	-1.91853	-0.3209
C	-4.84805	-2.01054	-0.418
C	-5.65071	-0.86141	-0.41617
C	-5.0312	0.38502	-0.31215
C	-3.64323	0.49311	-0.21539
H	-2.84182	-2.80735	-0.31312
H	-5.31802	-2.98822	-0.49417
H	-5.64089	1.28485	-0.30439
H	-3.15256	1.45737	-0.12575
C	-7.15004	-0.98331	-0.52121
H	-7.43716	-1.486	-1.45007
H	-7.55449	-1.5711	0.30881
H	-7.62896	-0.00154	-0.50578

- Transition state 2

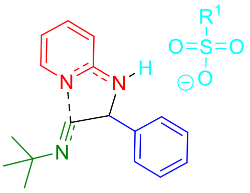

R[1] = 4-tolyl

Symbolic Z-matrix:

Charge = 0 Multiplicity = 1

C	-2.74453	-0.02454	-0.99102
H	-3.68732	-0.52576	-1.24739
C	-2.28887	-0.73884	0.26404
N	-2.12932	-0.74069	1.41854
C	-1.74624	-1.03373	2.78816
N	-1.7862	-0.2821	-2.0487
C	-2.99727	1.44616	-0.75055
C	-4.19343	2.00414	-1.20424
C	-2.04541	2.24542	-0.1114
C	-4.44377	3.36297	-1.02581
H	-4.92735	1.3769	-1.70391
C	-2.30367	3.60337	0.0654
H	-1.11017	1.81661	0.24696
C	-3.49726	4.16336	-0.3889
H	-5.37403	3.79303	-1.38288
H	-1.56367	4.22333	0.56129
H	-3.68976	5.22232	-0.247
C	-0.50492	-1.92736	2.7584
H	-0.18305	-2.10489	3.78899
H	-0.73001	-2.89034	2.291
H	0.29615	-1.4443	2.19503
C	-2.94132	-1.74244	3.43317
H	-3.16771	-2.67791	2.91337
H	-2.68952	-1.9731	4.47158
H	-3.82838	-1.10331	3.42297
C	-1.45091	0.30279	3.4737
H	-2.33378	0.94825	3.45209
H	-1.18011	0.10997	4.51584
H	-0.6207	0.79926	2.96653
C	-1.23591	-1.55448	-1.93324

C	-0.25265	-2.04895	-2.80309	C	2.97519	0.48282	-0.02838
C	-1.17881	-3.47313	-0.63577	C	3.45001	1.70567	-0.49855
C	0.25386	-3.30318	-2.54122	C	4.80432	1.99858	-0.38723
H	0.10548	-1.42903	-3.61616	C	5.69646	1.08434	0.18798
C	-0.21627	-4.04139	-1.44119	C	5.19632	-0.13517	0.64887
H	-1.57452	-3.9801	0.2399	C	3.8396	-0.4419	0.54363
H	1.03063	-3.71532	-3.17712	H	2.7577	2.40463	-0.95754
H	0.17349	-5.02664	-1.21582	H	5.18064	2.94999	-0.75598
N	-1.68742	-2.2586	-0.89976	H	5.8769	-0.85744	1.09253
S	1.22454	0.12761	-0.11713	H	3.44682	-1.39432	0.88556
O	0.60121	0.78993	1.05784	C	7.16467	1.41424	0.28718
O	0.77204	0.72758	-1.40107	H	7.61908	1.47715	-0.70695
O	1.09952	-1.34448	-0.06382	H	7.31794	2.37906	0.77999
H	-1.038	0.42041	-2.08846	H	7.70368	0.65283	0.85591

- Molecular complex 4

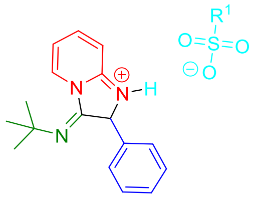

Symbolic Z-matrix:

Charge = 0 Multiplicity = 1

C	-1.92057	1.17194	-1.42525	H	-0.082	5.34099	-2.39181
H	-2.77452	1.61347	-1.95812	H	2.16289	2.74549	0.20132
C	-2.43777	0.60625	-0.09957	H	1.92988	4.91159	-1.001
N	-2.50864	1.25313	0.95625	C	-2.01717	-0.33474	2.82598
C	-2.73987	0.94735	2.37222	H	-1.80374	-0.25783	3.89615
N	-1.50389	-0.03373	-2.15651	H	-2.60712	-1.24196	2.68702
C	-0.83954	2.2143	-1.28876	H	-1.07167	-0.46034	2.28768
C	-0.96844	3.42686	-1.9651	C	-4.24515	0.91689	2.66141
C	0.29142	1.97236	-0.50272	H	-4.76122	0.11578	2.12437
C	0.0259	4.39944	-1.8628	H	-4.40455	0.76359	3.73289
H	-1.84919	3.61439	-2.57433	H	-4.70705	1.86583	2.37452
C	1.28379	2.94346	-0.40508	C	-2.12132	2.13949	3.11906
H	0.38715	1.02429	0.02265	H	-2.56102	3.07514	2.76442
C	1.15342	4.1571	-1.08233	H	-2.29136	2.04517	4.19583

H	-1.04393	2.17557	2.93337	H	-0.52602	-0.12998	-2.46435
C	-1.97355	-1.12596	-1.53095	C	2.66219	-1.13592	0.00198
C	-1.78954	-2.47387	-1.89632	C	3.28283	-0.05231	-0.60737
C	-3.32046	-1.76197	0.32187	C	4.47217	0.44316	-0.07149
C	-2.37594	-3.43339	-1.11511	C	5.04793	-0.13534	1.06156
H	-1.14423	-2.70293	-2.73493	C	4.40514	-1.23102	1.65306
C	-3.18867	-3.07789	-0.00763	C	3.21956	-1.73466	1.1305
H	-3.9232	-1.40243	1.14154	H	2.83717	0.38469	-1.49571
H	-2.21794	-4.4805	-1.34926	H	4.96317	1.28833	-0.54808
H	-3.69664	-3.83114	0.58036	H	4.84579	-1.69622	2.53171
N	-2.69022	-0.81122	-0.42033	H	2.7256	-2.59137	1.57895
S	1.09311	-1.73414	-0.61611	C	6.3472	0.38126	1.62644
O	0.0606	-1.12565	0.2764	H	7.16746	-0.31133	1.41055
O	0.99478	-1.19305	-2.00401	H	6.60728	1.35189	1.19736
O	1.12696	-3.20012	-0.53939	H	6.28845	0.49239	2.71308

1.10.3. Step 3 – Methanol acting as a proton shuttle

- Molecular complex 5

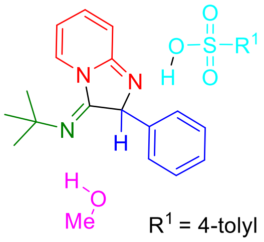

Symbolic Z-matrix:

Charge = 0 Multiplicity = 1

C	1.23656	0.18407	0.9695	C	-0.61044	3.28138	-0.27265
H	1.45144	0.66929	1.92757	H	0.48035	2.74148	1.51686
C	2.56271	-0.20454	0.30029	C	-1.0356	2.8627	-1.5316
N	3.4739	0.64809	0.11895	H	-1.08574	1.23183	-2.94314
C	4.80472	0.58399	-0.49641	H	-0.85281	4.27874	0.08234
N	0.52546	-1.07089	1.16533	H	-1.61574	3.53099	-2.1607
C	0.44	1.13613	0.09259	C	4.69148	0.32024	-2.00612
C	0.00018	0.71293	-1.16584	H	5.6889	0.32067	-2.45639
C	0.12977	2.41953	0.5401	H	4.20355	-0.62204	-2.25847
C	-0.73128	1.57465	-1.9765	H	4.10581	1.11972	-2.46882
H	0.18372	-0.30754	-1.49274	C	5.72181	-0.39898	0.24818

H	5.36582	-1.42955	0.25003	C	-3.82343	-0.58828	0.12895
H	6.71861	-0.3845	-0.20332	C	-3.35562	0.72469	0.14603
H	5.81604	-0.08385	1.29135	C	-4.25917	1.76225	-0.03881
C	5.39982	1.99015	-0.32034	C	-5.62278	1.50694	-0.2407
H	5.47908	2.24153	0.7421	C	-6.06138	0.18175	-0.25166
H	6.39644	2.04448	-0.76874	C	-5.16811	-0.87344	-0.06589
H	4.75698	2.73239	-0.80381	H	-2.30139	0.93351	0.30686
C	0.8111	-3.3942	0.45545	H	-3.89945	2.78839	-0.02493
C	3.22012	-2.49071	-0.66138	H	-7.11582	-0.03151	-0.40346
C	1.62428	-4.24578	-0.21223	H	-5.50118	-1.90604	-0.06309
H	-0.13752	-3.6949	0.88444	C	-6.58742	2.64989	-0.42861
C	2.86025	-3.78777	-0.78896	H	-7.59298	2.28715	-0.65266
H	4.12924	-2.09571	-1.08082	H	-6.64261	3.26412	0.4757
H	1.3386	-5.28602	-0.32671	H	-6.26862	3.29994	-1.24895
H	3.50576	-4.46528	-1.33245	C	1.19342	-2.0122	0.58409
N	2.41285	-1.60118	0.02083	C	3.01154	4.10098	1.36081
S	-2.67379	-1.93208	0.31364	H	4.0306	4.40055	1.0797
O	-1.71233	-1.89422	-0.78901	H	2.64291	4.81026	2.10588
O	-1.91928	-1.56203	1.6372	H	2.36916	4.16609	0.47115
O	-3.44207	-3.14794	0.52093	O	2.97958	2.81537	1.94104
H	-0.89891	-1.29526	1.45928	H	3.18263	2.15448	1.25075

- Transition state 3

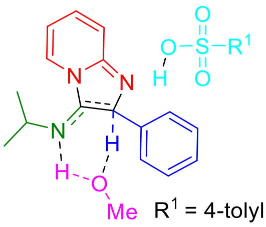

Symbolic Z-matrix:

Charge = 0 Multiplicity = 1

C	1.5449	0.38703	0.05333	C	0.251	2.26093	-0.99223
H	1.7709	0.80296	1.4246	C	2.62642	2.55984	-0.70777
C	2.76362	-0.42186	0.05964	C	0.16029	3.5396	-1.53356
N	3.88305	0.05489	0.54056	H	-0.64741	1.65753	-0.93031
C	5.20934	-0.54304	0.71101	C	2.52653	3.83762	-1.25575
N	0.45762	-0.48155	-0.22117	H	3.59581	2.18186	-0.40102
C	1.48763	1.75332	-0.5629	C	1.2938	4.34112	-1.66392

H	-0.80855	3.89971	-1.86583	O	-2.79139	0.73917	-1.84556
H	3.42412	4.43968	-1.36515	O	-1.97754	-0.12038	0.3298
H	1.21886	5.33774	-2.08703	O	-2.7717	-1.72502	-1.36929
C	5.84283	-0.90291	-0.64205	H	-0.9315	-0.26026	0.05713
H	6.83218	-1.34665	-0.49094	C	-4.51709	-0.21322	-0.10595
H	5.2461	-1.59516	-1.2378	C	-5.14632	1.0261	-0.06518
H	5.96051	0.00702	-1.2378	C	-6.37926	1.13266	0.57173
C	5.19391	-1.70837	1.71722	C	-6.98462	0.01835	1.16203
H	4.57294	-2.55057	1.41129	C	-6.32638	-1.21518	1.10299
H	6.21175	-2.07844	1.87835	C	-5.09389	-1.34041	0.47006
H	4.80548	-1.35115	2.67618	H	-4.67474	1.88245	-0.53624
C	6.06529	0.57545	1.32887	H	-6.8814	2.09563	0.60929
H	5.66118	0.87364	2.3025	H	-6.78769	-2.0886	1.55615
H	7.09606	0.23894	1.47573	H	-4.58288	-2.2958	0.40898
H	6.07212	1.45136	0.67322	C	-8.33616	0.13572	1.81874
C	0.21468	-2.87158	-0.74227	H	-8.44196	-0.58129	2.63661
C	3.01976	-2.89804	-0.53904	H	-8.49578	1.14003	2.21859
C	0.91146	-4.01466	-0.95866	H	-9.13363	-0.0651	1.09541
H	-0.86406	-2.79387	-0.84412	C	0.93274	-1.67288	-0.42239
C	2.34638	-4.02559	-0.86161	C	2.42624	2.59462	2.52419
H	4.09292	-2.8617	-0.46813	H	3.28857	3.1072	2.08922
H	0.38946	-4.92683	-1.22639	H	2.3982	2.75338	3.60333
H	2.90723	-4.93188	-1.05271	H	1.51648	2.98795	2.06334
N	2.33405	-1.71818	-0.29896	O	2.51899	1.18609	2.28614
S	-2.93096	-0.3523	-0.8891	H	3.33564	0.87436	1.63825

- Molecular complex 6

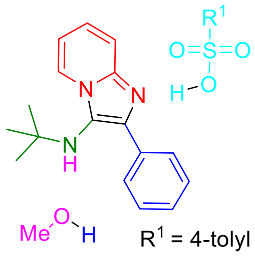

Symbolic Z-matrix:

Charge = 0 Multiplicity = 1

C	1.58447	0.22388	0.09565	C	2.75372	-0.52	0.16024
H	2.66154	0.30769	2.65089	N	4.12101	-0.25503	0.11516

C	4.77895 -0.02142 -1.19534			H	4.13826 -2.84048 0.47646		
N	0.49374 -0.60443 0.1761			H	0.49089 -5.19014 0.54868		
C	1.39692 1.67719 -0.0601			H	2.99575 -5.08741 0.66169		
C	0.29702 2.14536 -0.79206			N	2.32495 -1.84455 0.29414		
C	2.29 2.59667 0.50364			S	-2.73627 -0.58707 -0.78272		
C	0.10307 3.51413 -0.95723			O	-2.38529 0.23682 -1.93528		
H	-0.40035 1.44289 -1.24184			O	-1.96649 -0.07994 0.48346		
C	2.09018 3.96412 0.33295			O	-2.58263 -2.03687 -0.89239		
H	3.1335 2.23789 1.08577			H	-0.90938 -0.2665 0.35749		
C	0.99693 4.42686 -0.3985			C	-4.41209 -0.23189 -0.32245		
H	-0.74945 3.8647 -1.53023			C	-4.95501 1.00865 -0.63923		
H	2.78727 4.66886 0.7764			C	-6.26062 1.28958 -0.24849		
H	0.84217 5.49319 -0.53056			C	-7.023 0.34707 0.44999		
C	4.52172 -1.22886 -2.09879			C	-6.44839 -0.89234 0.75241		
H	4.99641 -1.0736 -3.07189			C	-5.14448 -1.1906 0.36959		
H	4.93366 -2.14208 -1.65893			H	-4.36023 1.72962 -1.19072		
H	3.44943 -1.37387 -2.2705			H	-6.69655 2.25501 -0.49088		
C	6.27368 0.10154 -0.90082			H	-7.03125 -1.63355 1.29264		
H	6.64494 -0.80524 -0.41432			H	-4.69524 -2.15347 0.59001		
H	6.83277 0.25983 -1.82754			C	-8.44778 0.64779 0.83871		
H	6.47018 0.95346 -0.23987			H	-8.72864 0.11783 1.75217		
C	4.27544 1.25094 -1.88742			H	-8.59538 1.71804 1.00246		
H	4.45399 2.13203 -1.26306			H	-9.13645 0.33412 0.04701		
H	4.79445 1.39342 -2.84114			C	0.94754 -1.85289 0.27832		
H	3.20148 1.19178 -2.09016			C	3.28459 1.70615 3.87575		
C	0.25361 -3.08414 0.3632			H	4.25856 2.13516 4.11563		
C	3.06626 -2.98748 0.44125			H	2.85797 1.27688 4.78859		
C	0.99146 -4.22991 0.49097			H	2.62555 2.50252 3.50872		
H	-0.82909 -3.07026 0.28927			O	3.50568 0.71346 2.88548		
C	2.41623 -4.18018 0.54313			H	4.41284 0.39398 0.84109		

1.11. Proposal K: Methanol addition to imine, catalyzed by *p*-toluenesulfonic acid

1.11.1. Step 1 – Methanol nucleophilic attack to imine

- Molecular complex 1

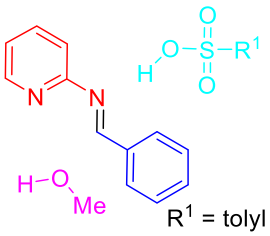

Symbolic Z-matrix:

Charge = 0 Multiplicity = 1

C	2.71872	0.59884	-0.32181	O	-0.11967	-0.77692	1.03329
H	3.34068	1.38153	0.10447	O	-0.58232	-2.82334	-0.33177
N	1.58452	0.96927	-0.82206	O	-0.38263	-0.60028	-1.39227
C	3.20274	-0.76723	-0.30786	H	0.82535	0.25111	-1.1214
C	2.56981	-1.8134	-0.99886	C	-2.51531	-1.08827	0.03893
C	4.33739	-1.02721	0.47714	C	-2.94908	0.04131	0.72525
C	3.06419	-3.10561	-0.88402	C	-4.31543	0.28999	0.82954
H	1.69147	-1.6303	-1.60959	C	-5.25089	-0.57727	0.25614
C	4.83113	-2.32055	0.57873	C	-4.78622	-1.70614	-0.42764
H	4.80024	-0.21394	1.02832	C	-3.42404	-1.96938	-0.53846
C	4.19122	-3.35872	-0.10043	H	-2.21941	0.69951	1.18816
H	2.55678	-3.91574	-1.39569	H	-4.66211	1.16624	1.37141
H	5.70129	-2.52342	1.19364	H	-5.50249	-2.39101	-0.87444
H	4.56929	-4.37264	-0.01333	H	-3.05679	-2.8511	-1.05391
C	1.12291	2.31279	-0.76445	C	-6.73026	-0.32509	0.4005
C	-0.21723	2.5502	-1.07621	H	-7.14564	-0.92292	1.21871
C	1.54359	4.49651	-0.30977	H	-6.93388	0.7259	0.61942
C	-0.66559	3.8617	-0.97679	H	-7.26778	-0.59612	-0.51206
H	-0.86152	1.72523	-1.36558	O	2.03586	0.50344	2.20379
C	0.22565	4.85787	-0.58274	H	1.4504	-0.22634	1.93402
H	2.27362	5.24271	-0.0083	C	1.19772	1.5695	2.60598
H	-1.70014	4.09996	-1.2017	H	0.72614	1.36654	3.57521
H	-0.08593	5.89207	-0.49184	H	1.81816	2.4649	2.69577
N	1.99301	3.24378	-0.39963	H	0.40603	1.75535	1.86754
S	-0.76766	-1.38508	-0.15252				

- Transition state 1

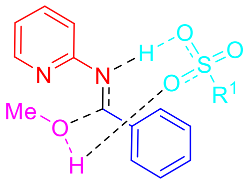

R[1] = tolyl

Symbolic Z-matrix:

Charge = 0 Multiplicity = 1

C	2.64809	-0.29518	0.30617	O	-0.68774	-1.16785	0.97636
H	3.50418	0.05342	0.87672	O	-1.33345	-2.04372	-1.263
N	2.12597	0.59359	-0.53039	O	-0.48522	0.27115	-1.00962
C	2.52358	-1.74035	0.05703	H	1.20286	0.36525	-0.95324
C	1.80422	-2.24858	-1.02804	C	-2.88189	-0.27106	-0.11097
C	3.13505	-2.61511	0.96439	C	-3.11493	0.65549	0.90363
C	1.68078	-3.62459	-1.19029	C	-4.40014	1.1474	1.09137
H	1.31689	-1.58819	-1.7382	C	-5.46062	0.72386	0.27836
C	3.02557	-3.98785	0.78792	C	-5.19957	-0.2066	-0.72864
H	3.67537	-2.2093	1.81506	C	-3.91353	-0.70894	-0.92983
C	2.29274	-4.49213	-0.28865	H	-2.29653	0.97203	1.54312
H	1.08755	-4.00994	-2.01191	H	-4.59046	1.86729	1.88367
H	3.498	-4.66453	1.49219	H	-6.01243	-0.54814	-1.36383
H	2.19341	-5.56516	-0.41888	H	-3.70329	-1.44045	-1.70319
C	2.45189	1.96552	-0.51081	C	-6.85212	1.25676	0.508
C	1.60436	2.85156	-1.18592	H	-7.24735	0.91128	1.4687
C	3.86297	3.62775	0.13802	H	-6.85574	2.35069	0.5249
C	1.9482	4.19476	-1.17529	H	-7.53675	0.92613	-0.27627
H	0.70739	2.47947	-1.67201	O	1.49868	-0.25936	1.90012
C	3.1023	4.59988	-0.50217	H	0.66925	-0.73783	1.55015
H	4.76818	3.89591	0.67624	C	1.0705	1.05777	2.22825
H	1.32008	4.91999	-1.68257	H	0.41737	1.01465	3.10464
H	3.40488	5.64003	-0.47191	H	1.95395	1.65782	2.457
N	3.55097	2.32774	0.13799	H	0.52098	1.50528	1.39087
S	-1.22745	-0.8743	-0.39066				

- Molecular complex 2

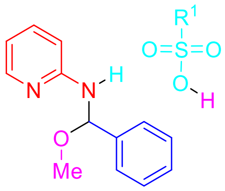

R[1] = tolyl

Symbolic Z-matrix:

Charge = 0 Multiplicity = 1

C	-2.25101	0.7262	0.69337	C	3.06908	-0.36936	-0.13287
H	-3.26106	0.87057	1.09018	C	3.12036	-1.75895	-0.04884
N	-2.31245	-0.29789	-0.29403	C	4.36156	-2.37985	0.00339
C	-1.67362	2.0217	0.14926	C	5.54594	-1.63089	-0.02914
C	-1.7145	2.30507	-1.21637	C	5.45943	-0.23929	-0.11584
C	-1.1153	2.95664	1.02654	C	4.2234	0.40219	-0.16921
C	-1.1903	3.50135	-1.70115	H	2.20023	-2.33443	-0.03582
H	-2.14865	1.58384	-1.90058	H	4.41718	-3.46336	0.0671
C	-0.59099	4.15	0.53936	H	6.37	0.35236	-0.14444
H	-1.07624	2.73924	2.08895	H	4.1456	1.48172	-0.24608
C	-0.62511	4.42461	-0.82621	C	6.8827	-2.32549	0.00572
H	-1.21474	3.70443	-2.76705	H	6.90921	-3.08601	0.79098
H	-0.14649	4.86178	1.22775	H	7.08087	-2.82741	-0.94686
H	-0.20525	5.35041	-1.20666	H	7.69328	-1.61658	0.18743
C	-3.30123	-1.25794	-0.35707	O	-1.40615	0.3535	1.80891
C	-3.17027	-2.31807	-1.28046	H	0.11138	0.41677	1.5009
C	-5.3261	-2.05445	0.35829	C	-1.84196	-0.80394	2.51397
C	-4.1814	-3.25612	-1.34982	H	-1.17635	-0.91568	3.37061
H	-2.29198	-2.37738	-1.91556	H	-2.8746	-0.66938	2.84924
C	-5.29801	-3.13188	-0.5146	H	-1.7817	-1.69316	1.87804
H	-6.16902	-1.91158	1.03063				
H	-4.10609	-4.08167	-2.05124				
H	-6.11284	-3.84532	-0.54102				
N	-4.35707	-1.13571	0.45056				
S	1.49381	0.43953	-0.18386				
O	1.12225	0.42449	1.35549				
O	1.68462	1.79798	-0.65102				
O	0.5453	-0.42956	-0.88638				
H	-1.44129	-0.47699	-0.78636				
